# Sestrin2-Mediated Autophagy Contributes to Drug Resistance *via* Endoplasmic Reticulum Stress in Human Osteosarcoma

**DOI:** 10.3389/fcell.2021.722960

**Published:** 2021-09-27

**Authors:** Zhen Tang, Xinghui Wei, Tian Li, Wei Wang, Hao Wu, Hui Dong, Yichao Liu, Feilong Wei, Lei Shi, Xiaokang Li, Zheng Guo, Xin Xiao

**Affiliations:** ^1^Department of Orthopaedics, Xijing Hospital, Fourth Military Medical University, Xi’an, China; ^2^School of Basic Medicine, Fourth Military Medical University, Xi’an, China; ^3^State Key Laboratory of Cancer Biology, Department of Immunology, Fourth Military Medical University, Xi’an, China; ^4^Department of Orthopaedics, Tangdu Hospital, Fourth Military Medical University, Xi’an, China

**Keywords:** Sestrin2, apoptosis, autophagy, drug resistance, endoplasmic reticulum stress

## Abstract

One contributor to the high mortality of osteosarcoma is its reduced sensitivity to chemotherapy, but the mechanism involved is unclear. Improving the sensitivity of osteosarcoma to chemotherapy is urgently needed to improve patient survival. We found that chemotherapy triggered apoptosis of human osteosarcoma cells *in vitro* and *in vivo*; this was accompanied by increased Sestrin2 expression. Importantly, autophagy was also enhanced with increased Sestrin2 expression. Based on this observation, we explored the potential role of Sestrin2 in autophagy of osteosarcoma. We found that Sestrin2 inhibited osteosarcoma cell apoptosis by promoting autophagy via inhibition of endoplasmic reticulum stress, and this process is closely related to the PERK-eIF2α-CHOP pathway. In addition, our study showed that low Sestrin2 expression can effectively reduce autophagy of human osteosarcoma cells after chemotherapy, increase p-mTOR expression, decrease Bcl-2 expression, promote osteosarcoma cell apoptosis, and slow down tumour progression in NU/NU mice. Sestrin2 activates autophagy by inhibiting mTOR via the PERK-eIF2α-CHOP pathway and inhibits apoptosis via Bcl-2. Therefore, our results explain one underlying mechanism of increasing the sensitivity of osteosarcoma to chemotherapy and suggest that Sestrin2 is a promising gene target.

## Introduction

Neoplasms remain the primary killer worldwide (Burns et al., 2020; Pantziarka et al., 2021). Osteosarcoma is a highly aggressive tumour occurring in long bones and is one of the most common primary malignancies in adolescents and young adults (Liu et al., 2020; Pan et al., 2021). Regardless of improvements in treatment options, including surgery with adjuvant chemotherapy, the recurrence rate of this malignant disease is still as high as 30–40%, and the 10-year survival rate is as low as 20–30% due to drug-resistant metastasis (Yen et al., 2018; Chen J. et al., 2019). Therefore, to develop more effective and comprehensive treatments for clinical application, it is necessary to identify the underlying mechanisms relating to the proliferation, drug resistance and recurrence of osteosarcoma (Gu et al., 2018; Liu et al., 2021).

Autophagy plays a key role in numerous physiological and pathological processes, such as development and growth, as well as various diseases, including cancer (Dodson et al., 2013; Li et al., 2020a,b). Autophagy is considered an intracellular degradation process that is usually triggered by a variety of stress factors, such as nutrient deficiency, hypoxia, intracellular reactive oxygen species (ROS), oxidative stress and chemical drugs (Huang et al., 2018; Mrakovcic and Frohlich, 2019). In autophagy, abnormally expressed or folded cytoplasmic proteins and disrupted organelles are selectively transferred to lysosomes for degradation (Wang W. et al., 2018; Liu et al., 2019). Programmed cell death caused by autophagy plays an important role in various malignant tumours, but there is considerable controversy about the precise role of autophagy in tumour occurrence and progression, especially regarding the regulation of chemoresistance in malignant tumours. Some studies have suggested that autophagy can induce programmed death of tumour cells and thus inhibit tumorigenesis and metastasis (Mrakovcic and Frohlich, 2019). However, accumulating evidence has indicated that autophagy may protect cancer cells from further damage by removing damaged organelles, increasing their metabolism and recycling misfolded macromolecules. Autophagy is also believed to inhibit the exposure of cancer cells to accumulated damage and reduce apoptosis, and enhance the viability of cancer cells, thereby promoting tumour proliferation, drug resistance and metastasis (Wang et al., 2019). However, the relationship between autophagy and apoptosis, and the underlying mechanism of autophagy in drug resistance of osteosarcoma remains unclear (Hattori et al., 2021).

The acquisition of drug resistance is a very complex process mediated by gene mutations, changes in gene expression, selective splicing, post-translational protein modification, etc (Aleksakhina et al., 2019; Khan et al., 2021; Patra et al., 2021; Peixoto da Silva et al., 2021). Sestrins (SESNs) are highly conserved chaperones that play an important role in cell survival (Crisol et al., 2018). They are responsible for many cytoprotective mechanisms, especially under stress conditions (Dai et al., 2018). The expression of SESNs is upregulated in a wide range of tumours in response to cell stress and is closely associated with resistance to chemotherapy (Crisol et al., 2018; Sanchez-Alvarez et al., 2019). Previous studies have reported that the SESN family of proteins are considered as emerging targets for pharmacological intervention (Sanchez-Alvarez et al., 2019). Importantly, it has been reported that SESNs can regulate autophagy (Dai et al., 2018). There are correlations between the expression and activity of SESNs with unfolded protein responses from mitochondria and endoplasmic reticulum (ER), and eIF2AK2/PKR kinase centralised on the phosphorylation of the eIF2α translation initiation factor (Ye et al., 2015; Kimball et al., 2016). Endoplasmic reticulum stress-inducing drugs like bortezomib and nelfinavir resulted in the upregulation of Sestrin2 (SESN2) along with ER stress markers in breast, ovarian and cervical adenocarcinoma cancer cell lines (Bruning et al., 2013). Sestrin2, a member of the SESN family, was found to be one of the most important cellular stress proteins and to be involved in tumour progression and cancer cell invasion (Lear et al., 2019; Zhu et al., 2020). Jia-Hau Yen et al demonstrated that TIIA-mediated autophagy occurred in a SESN2-dependent but not Beclin-1-dependent manner in human osteosarcoma 143B cells, suggesting that SESN2 is a potential molecular target for cancer therapy (Yen et al., 2018; Jeong et al., 2019). However, little is known about the role of SESN2-dependent autophagy in drug resistance of human osteosarcoma. Studies should focus on determining the role of SESN2 in osteosarcoma cells with a particular focus on autophagy and its potential effects on drug resistance, and the underlying molecular mechanism of chemotherapy-driven SESN2-mediated autophagy in osteosarcoma also needs to be explored.

In this study, we found that SESN2 expression was upregulated in osteosarcoma cells during chemotherapy; thus, we further investigated the effects of SESN2 on the proliferation, apoptosis, drug resistance of osteosarcoma cells. In addition, we explored the potential mechanism by which SESN2 promotes drug resistance in osteosarcoma cells through increased autophagy. SESN2 activates autophagy and inhibits apoptosis through the PERK-eIF2α-CHOP pathway and Bcl-2. Our findings provide a novel therapeutic target for the treatment of osteosarcoma.

## Materials and Methods

### Cell Culture and Reagents

The human osteosarcoma cell lines MG-63, HOS and 143B from the Cell Bank of Chinese Academy of Medical Sciences (Shanghai, China) were grown in Eagle’s Minimum Essential medium (Gibco, Los Angeles, CA) containing 10% foetal bovine serum (Gibco) and antibiotics at 37°C under 5% CO_2_. Cisplatin (Cis), doxorubicin (Dox), methotrexate (Mtx), ZVAD-FMK, rapamycin (Rap), and 3-methyladenine (3-MA), 4-Phenylbutyric acid (4-PBA) were purchased from Sigma Aldrich (St. Louis, Missouri, United States).

### Cell Transfection

MG-63, HOS and 143B cells were seeded into 12-well plates and incubated at 37°C for 24 h until the cells reached approximately 50-60% confluence. A plasmid carrying the SESN2 gene and control/SESN2-specific shRNAs was purchased from Hanbio Co., Ltd. (Shanghai, China). Subsequently, the cells were transfected using a plasmid carrying the SESN2 gene according to the manufacturer’s instructions. The sequence of shSESN2 was 5′-GGTCCACGTGAACTTGCTGC-3′. Western blot analysis was used to detect the protein expression level of SESN2 in osteosarcoma cell lines subjected to lentiviral transduction.

### Cell Viability Assay

Osteosarcoma cells were seeded into 96-well plates. After incubation for 24 h, cells were treated with cisplatin (Cis, 20 μmol/L), doxorubicin (Dox, 0.2 μg/mL) or methotrexate (Mtx, 50 μmol/L) at the indicated concentrations for 24 h, 48 h, and 72 h. Cell viability was determined with a Cell Counting Kit-8 Assay Kit (Beyotime, Shanghai, China) and Caspase-3 colorimetric assay kit (Solarbio, Beijing, China) was used according to the manufacturer’s instructions.

### Colony Formation Assay

MG-63 and HOS cells in the logarithmic growth phase were seeded into a 10-cm dish at a density of 200 cells per millilitre. After treatment with Cis (20 μmol/L) and Dox (0.2 μg/mL) for 2 weeks, cell clones were fixed with 4% paraformaldehyde for 15 min. Subsequently, crystal violet solution was added to stain the cell clones for 20 min before they were photographed.

### Apoptosis Analysis

After osteosarcoma cells were seeded into 6-well plates and incubated overnight, they were treated with Cis (20 μmol/L), Dox (0.2 μg/mL), or Mtx (50 μmol/L) for 24 h. Apoptotic cells were determined using an Annexin V-PE Apoptosis Detection kit (BD, Shanghai, China) by flow cytometry according to the manufacturer’s instructions.

### Western Blot Analysis

Cells were washed with PBS and lysed in radioimmunoprecipitation assay (RIPA) buffer, and then a BCA kit (Thermo Fisher Scientific, Shanghai, China) was used to determine the protein concentrations. Equal amounts of proteins were resolved by SDS-PAGE and transferred to activated PVDF membranes. The membranes were blocked with 5% non-fat dry milk and incubated with primary antibodies overnight at 4°C. The dilution ratio of primary antibodies against Sestrin2 (#8487, CST, United States), PARP (#9532, CST, United States), cleaved PARP (#5625, CST, United States), Beclin-1 (ab207612, Abcam, United States), LC3 (ab192890, Abcam, United States), P62 (ab109012, Abcam, United States), mTOR (ab2732, Abcam, United States), phospho-mTOR (ab131538, Abcam, United States), Bcl-2 (ab59348, Abcam, United States), PERK (ab65142, Abcam, United States), phospho-eIF2α (ab131505, Abcam, United States) and CHOP (ab11419, Abcam, United States) was 1:1000, and that of antibody against GAPDH (#5174, CST, United States) was 1:2000. After incubation with HRP-conjugated secondary antibodies (1:3000 dilution) for 40 min at room temperature, the protein bands were visualised with Amersham Imager 600.

### Quantitative Real-Time Polymerase Chain Reaction

Total RNA was isolated from cells by using TRIzol (Sigma, Ventura, United States) according to a procedure modified from a previously described protocol. Quantitative real-time PCR (qRT-PCR) experiments were performed using SYBR Green reagents (Takara Bio Inc., Shiga, Japan) with specific primers. The cycle threshold (Ct) values were collected and normalised to the level of respective. The ΔΔ Ct method was adopted as our previous study. The primers were listed as follows: SESN2 (F) 5′- GACCATGGCTACTCGCTGAT -3′,(R) 5′- GCT GCC TGG AAC TTC TCA TC -3′; GAPDH (F) 5′-CCACAGTCCATGCCATCAC-3′, (R) 5′-TCCACCACCCTG TTGCTGTA-3′.

### Immunofluorescence

To detect the formation of autolysosomes, cells were seeded into 24-well plates at 5 × 10^4^ cells per well. After treatment with the indicated chemotherapeutic drugs for 24 h, cells were fixed in 4% paraformaldehyde for 20 min and incubated with a primary antibody against LC3 (ab192890, Abcam, United States) at 4°C overnight. The cells were washed with PBS three times for 3 min each time, followed by incubation with secondary antibodies for 1 h at room temperature. DAPI was incubated with cells for 3 min to stain nuclei. The samples were sealed in and photographed under a fluorescence microscope within 24 h of antibody treatment.

### Measurement of Autophagic Flux

To analyse autophagic flux, HOS cells at a density of 50–70% were transiently transfected with GFP-RFP-LC3 (Hanbio Co. LTD) according to the manufacturer’s instructions. They were divided into groups according to experimental requirements and treated with chemotherapy drugs or rapamycin (100 nmol/L) for the corresponding time. The expression levels of GFP and mRFP were observed by confocal fluorescence microscopy.

### Transmission Electron Microscopy

Transmission electron microscopy (TEM) was performed to monitor autolysosome formation in cells treated with chemical drugs for 24 h. After the cells were centrifuged at 2000 × g for 15 min and the supernatant was removed, 1.5 mL of glutaraldehyde was added immediately and incubated overnight to fix cells in the pellet. Then, 1% osmium tetroxide was added and fixed at 4°C for 30 min. Next, a graded series of acetone was applied to the sample for 10 min per concentration before 1 mL of pure embedding agent was added and left overnight at room temperature. The cell mass was carefully inserted into the module at the bottom of the hole, and the embedding medium hole filling module was continued. When the sample hardened after 2 h at 60°C, the embedding blocks were sliced at a thickness of 50∼70 nm for follow-up observation.

### Intracellular Ca^2+^ Measurement

The levels of cytosolic Ca^2+^ were detected using Fura-Red-AM (CAS 149732-62-7, AAT Bioquest, United States). After treatment with Cis (20 μmol/L) and Dox (0.2 μg/mL) for 24 h, HOS cells were treated and harvested according to the manufacturer’s instructions. The fluorescence intensities of Fura Red alone and bound to Ca^2+^ were detected by flow cytometry.

### *In vivo* Subcutaneous Tumour Model

All animal work was carried out following the guidelines of the Ethics Committee of the Fourth Military Medical University and was performed according to the institutional guidelines for the care and use of laboratory animals. NU/NU mice (the Fourth Military Medical University, Shaanxi, China) were randomly assigned and used to investigate the effect of SESN2 on tumour formation *in vivo*. A total of 5 × 10^7^ 143B cells (sh-NC/sh- SESN2) suspended in serum-free medium were subcutaneously implanted into the flanks of mice. Tumour size was measured every 3 days from day 7 until day 28. The tumours were removed, weighed at day 28. NU/NU mice were intraperitoneally injected with chemotherapeutic drugs twice a week. The results were counted by three pathologists who were blind to the characteristics of each group. The tumour volume (mm^3^) was calculated using the following formula: length × width^2^/2.

## Results

### Sestrin2 Expression Is Increased in Response to Chemotherapy Drugs

**FIGURE 1:**
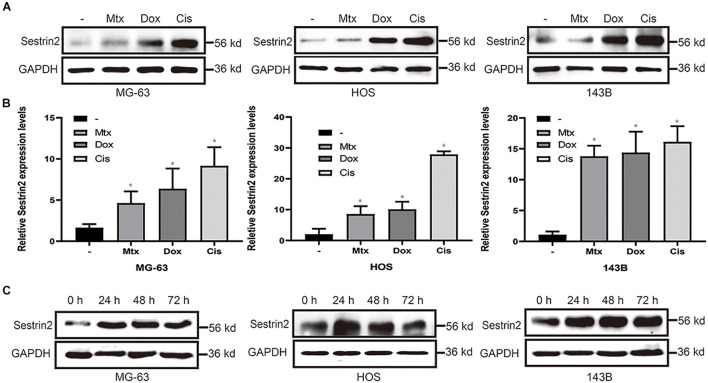
Chemotherapeutic agents increased the expression of SESN2 in osteosarcoma cells **(A,B)**. After treatment with Cis (20 μmol/L), Dox (0.2 μg/mL) or Mtx (50 μmol/L), the expression level of SESN2 in osteosarcoma cells was detected by western blot **(A)** (*n* = 3), and the SESN2 mRNA level was detected by quantitative real-time PCR **(B)** (*n* = 3). Western blotting was used to detect SESN2 expression in MG-63, HOS, and 143B cells after Cis (20 μmol/L) treatment for 24 to 72 h **(C)** (*n* = 3). The data are presented as the mean ± SD. *p < 0.05 vs. the – group.

To analyse the expression of SESN2 in osteosarcoma cells after chemotherapy, we detected the expression of SESN2 in HOS, MG63 and 143B cells treated with Cis (20 μmol/L), Dox (0.2 μg/mL) or Mtx (50 μmol/L). These chemotherapeutic agents significantly increased the expression of SESN2 in all three cell lines (Figure 1A) at the mRNA and protein level (Figure 1B). More importantly, the increases in SESN2 expression were time dependent. We detected SESN2 expression at 24, 48, and 72 h after treatment and found that it was highest at 24 h, and there was no significant decrease at 48 and 72 h (Figure 1C). Therefore, we can conclude that SESN2 expression increases in a time-dependent manner after chemotherapeutic treatment.

### Sestrin2 Reduces the Sensitivity of Osteosarcoma Cells to Chemotherapeutic Agents by Inhibiting Apoptosis to Confer Drug Resistance

**FIGURE 2:**
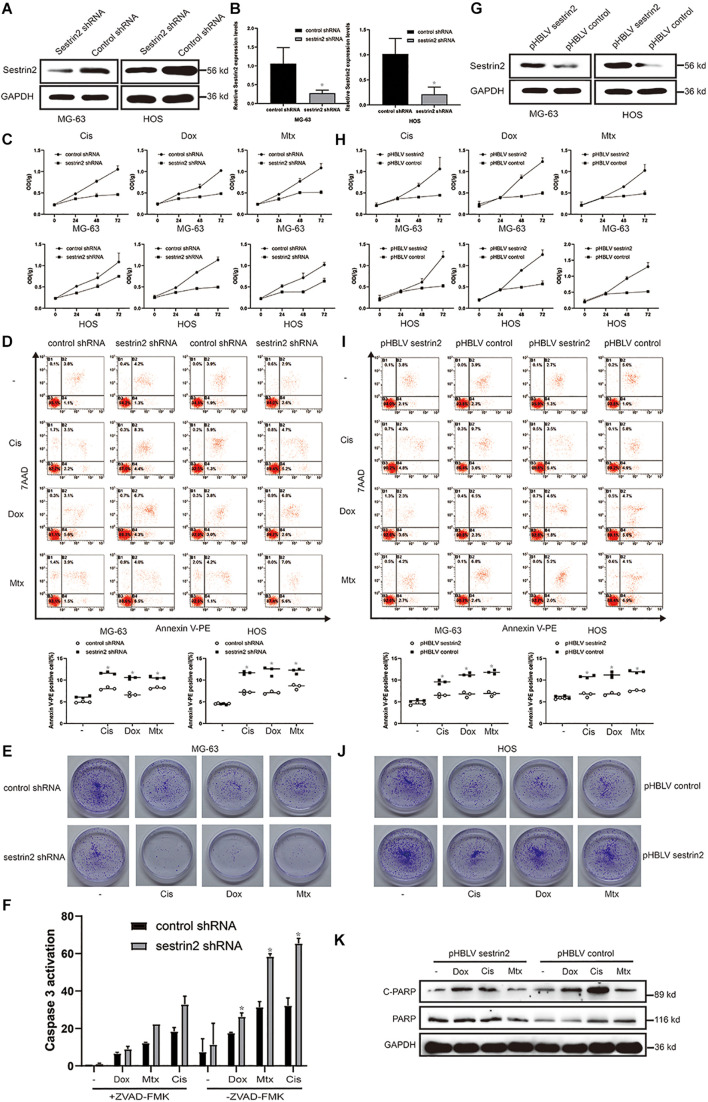
SESN2 confers drug resistance to target cells by reducing their sensitivity to chemotherapeutic agents. MG-63 and HOS cells were transfected with control shRNA or SESN2 shRNA, and the knockdown effect was detected by western blot (*n* = 3) and quantitative real-time PCR (*n* = 3) **(A,B)**. MG-63 and HOS cells with SESN2 knockdown were treated with Cis (20 μmol/L), Dox (0.2 μg/mL), or Mtx (50 μmol/L) for 24, 48, and 72 h. Cell activity was detected by CCK-8 **(C)** (*n* = 3). After treatment for 24 h, apoptotic cells were detected by flow cytometry **(D)** (*n* = 3). SESN2 knockdown significantly reduced the number of colony-forming MG-63 cells **(E)** (*n* = 3). After MG-63 cells were treated with Cis (20 μmol/L), Dox (0.2 μg/mL) or Mtx (50 μmol/L) for 24 h, the apoptosis of cells transfected with control shRNA or SESN2 shRNA and cultured in the presence or absence of ZVAD-FMK (20 μmol/L) was detected using the Caspase 3 kit **(F)** (*n* = 3). The data are presented as the mean ± SD. *p < 0.05 vs. the Control shRNA group. The expression of SESN2 after transfection of a plasmid carrying the SESN2 gene was evaluated by western blot **(G)** (*n* = 3). MG-63 and HOS cells overexpressing SESN2 as well as their corredsponding control cells were treated with Cis (20 μmol/L), Dox (0.2 μg/mL), or Mtx (50 μmol/L). Cell viability and apoptosis were detected by CCK-8 **(H)** (*n* = 3) and flow cytometry **(I)** (*n* = 3), respectively. SESN2 increases the number of colony-forming units of HOS cells **(J)** (*n* = 3). Cleaved and total PARP in SESN2-overexpressing and control HOS cells were analysed by western blot **(K)** (*n* = 3). The data are presented as the mean ± SD. *p < 0.05 vs. the pHBLV control group.

To further explore the underlying role of SESN2 in the drug resistance of osteosarcoma cells, we reduced SESN2 expression via lentivirus transduction with a plasmid containing shRNA targeting SESN2. And we found that SESN2 expression was significantly reduced at the protein and mRNA level after lentivirus transfection (Figures 2A,B). The inhibition of osteosarcoma cell proliferation upon SESN2 knockdown in cells treated with chemotherapeutic drugs was measured by the CCK-8 kit and colony formation assay (Figures 2C,E). Flow cytometry confirmed that SESN2 knockdown increased the sensitivity of osteosarcoma cells to chemotherapeutic agents and increased their apoptosis rates compared with those of the control shRNA-treated group (Figure 2D). After chemotherapy, the apoptotic protein caspase 3 was significantly activated in the SESN2-knockdown group, which was reversed by treatment with the apoptotic inhibitor ZVAD-FMK (20 μmol/L) (Figure 2F).

Sestrin2-overexpressing osteosarcoma cells were constructed using a lentiviral plasmid, and the increased expression was detected by western blot (Figure 2G). Cell proliferation curves showed that after chemotherapy treatment, compared to the control cells, cells in the SESN2 overexpression group proliferated faster and showed reduced sensitivity to chemotherapy drugs (Figures 2H,J). Increased SESN2 expression reduced apoptosis in MG-63 cells and HOS cells, as indicated by the decrease in the number of Annexin V-PE-positive cells (Figure 2I). Similarly, western blot analysis showed that cleaved PARP expression was lower in the SESN2 overexpression group than in the control group, suggesting that SESN2 exerts an antiapoptotic effect (Figure 2K). These results indicate that SESN2 reduces the sensitivity of osteosarcoma cells to chemotherapeutic agents by reducing the apoptosis of osteosarcoma cells and thus leads to drug resistance.

### Sestrin2 Reduces the Sensitivity of Osteosarcoma Cells to Chemotherapies by Promoting Autophagy

**FIGURE 3:**
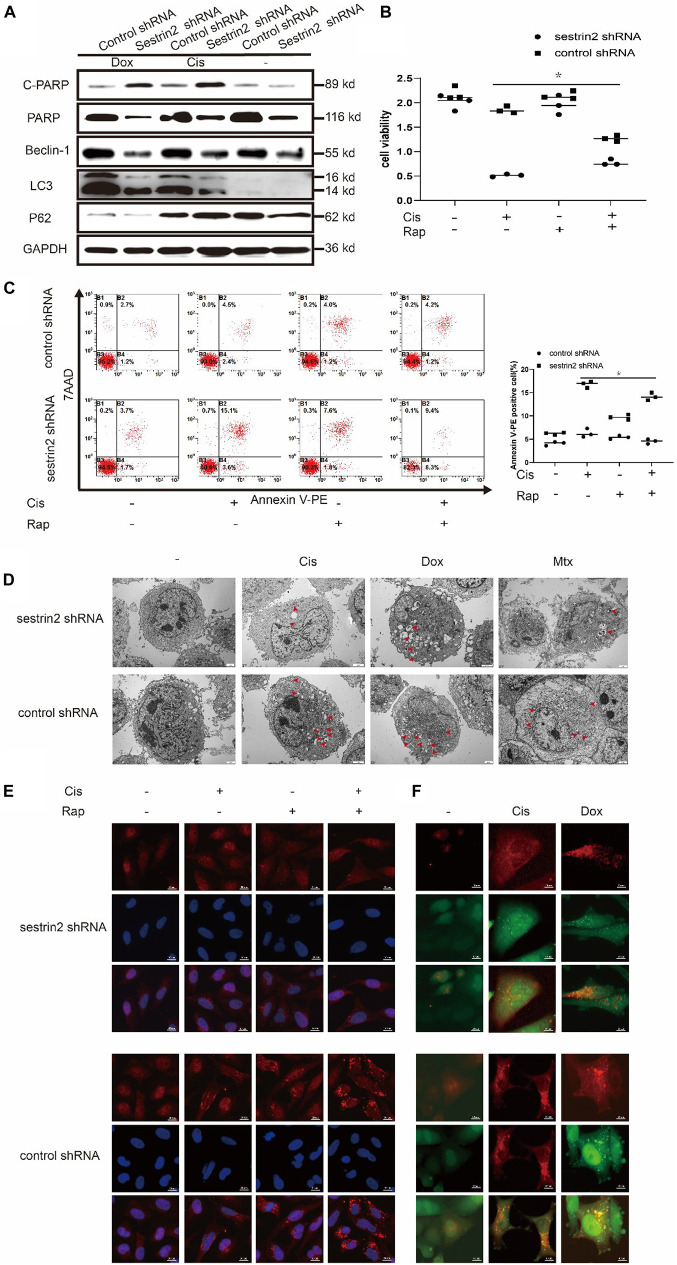
Knockdown of SESN2 resulted in inhibited autophagy and increased apoptosis of osteosarcoma cells treated with chemotherapy. After treatment with Cis (20 μmol/L), Dox (0.2 μg/mL), or Mtx (50 μmol/L) for 24 h, SESN2-knockdown and control cells were subjected to western blot to detect the expression of cleaved and total PARP, LC3, and P62 expression levels **(A)** (*n* = 3). SESN2-knockdown HOS cells were treated with Cis (20 μmol/L) for 24 h with or without rapamycin (100 nmol/L) for 6 h. Proliferation was analysed by CCK-8 assay **(B)** (*n* = 3), apoptosis was assessed by Annexin V-PE/PI staining **(C)** (*n* = 3), and LC3 puncta formation was analysed by immunofluorescence **(E)** (*n* = 3, scale bar = 20 μm). Intracellular autophagosomes were observed by TEM **(D)** (*n* = 3, scale bar = 2 μm), and autophagic flux was monitored by fluorescence microscopy in HOS cells with transient expression of GFP-RFP-LC3 in HOS cells **(F)** (*n* = 3, scale bar = 10 μm). The data are presented as the mean ± SD. *p < 0.05 vs. the Control shRNA group.

Previous reports have described the relationship between autophagy and apoptosis. Some studies have shown that autophagy protects cells under stress conditions, but others have proposed the opposite view: that autophagy can promote apoptosis. After treatment with Cis (20 μmol/L) and Dox (0.2 μg/mL), LC3 expression was significantly decreased in the SESN2 knockdown group compared to the control group, whereas P62 expression showed an upward trend. Cleaved PARP expression, which reflects the level of apoptosis, was increased upon knockdown of SESN2 (Figure 3A). Cell viability assays showed that the decrease in the cell proliferation rate induced by SESN2 knockdown after Cis (20 μmol/L) and Dox (0.2 μg/mL) treatment was reversed by treatment with the autophagy inducer rapamycin (100 nmol/L) (Figure 3B). The flow cytometry results showed that the number of Annexin V-PE-positive cells was increased by SESN2 knockdown after treatment with Cis (20 μmol/L) and Dox (0.2 μg/mL); this effect was reversed by rapamycin (100 nmol/L) (Figure 3C). Ultrastructural analysis of autophagosomes using TEM also showed that the number of intracellular autophagosomes increased significantly after SESN2 knockdown (Figure 3D). Similarly, autophagy flow was blocked in shRNA-transfected cells after chemotherapeutic drug treatment compared with control cells treated with the same drug (Figure 3F). As the immunofluorescence results show in Figure 3E, we observed that after chemotherapy treatment, knocking down SESN2 significantly reduced the recruitment of LC3 to the autophagy membrane even after treatment with rapamycin (100 nmol/L). These results suggest that SESN2 plays an important role in the inhibition of apoptosis by regulating autophagy after chemotherapeutic treatment.

**FIGURE 4:**
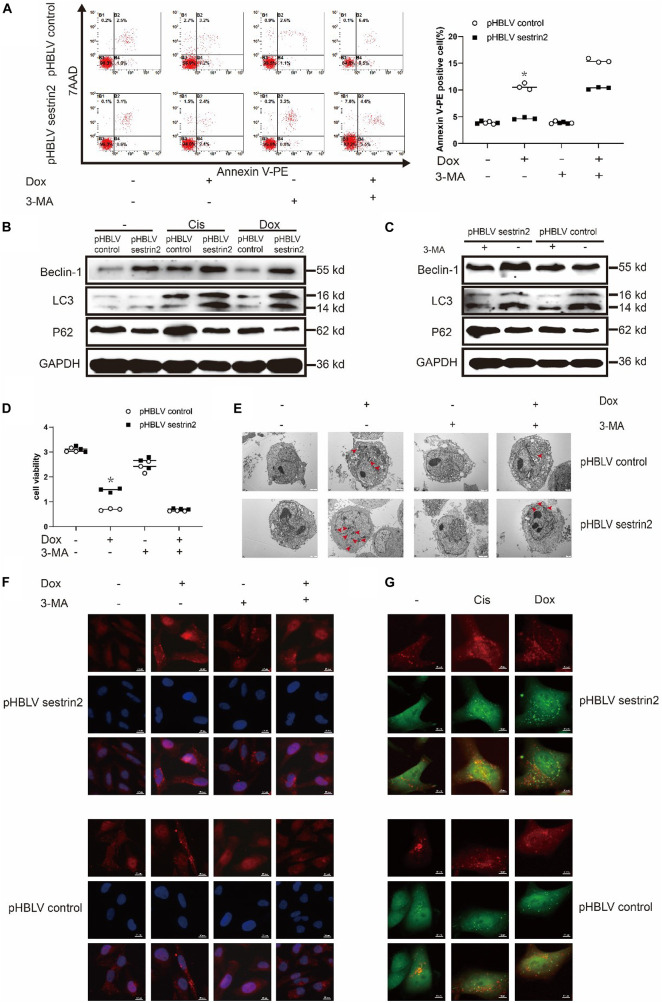
SESN2 regulates autophagy and reduces the sensitivity of osteosarcoma cells to chemotherapy. In the presence or absence of 3-MA (5 mM), SESN2-overexpressing and control HOS cells were treated with Dox (0.2 μg/mL) for 24 h, and apoptosis was analysed by flow cytometry **(A)** (*n* = 3). After treatment with Cis (20 μmol/L) or Dox (0.2 μg/mL), the expression levels of LC3 and P62 in SESN2-overexpressing and control HOS cells were detected by western blot **(B)** (*n* = 3). The expression levels of LC3 and P62 in SESN2-overexpressing and control HOS cells treated with 3-MA (5 mM) were detected by western blot **(C)** (*n* = 3). In the presence or absence of 3-MA (5 mM), SESN2-overexpressing and control HOS cells were treated with Dox (0.2 μg/mL) for 24 h, cell activity was detected by CCK-8 **(D)** (*n* = 3), intracellular autophagosomes were observed by TEM **(E)** (*n* = 3, scale bar = 2 μm), and intracellular LC3 puncta formation was analysed by immunofluorescence **(F)** (*n* = 3, scale bar = 20 μm). After treatment with Cis (20 μmol/L) or Dox (0.2 μg/mL), autophagosome formation in HOS cells with ectopic SESN2 expression was monitored by immunofluorescence through transfection with RFP-GFP-LC3 lentivirus after upregulating SESN2 **(G)** (*n* = 3, scale bar = 10 μm). The data are presented as the mean ± SD. *p < 0.05 vs. the pHBLV control group.

Annexin V-PE staining revealed that the apoptosis of SESN2-overexpressing osteosarcoma cells treated with Dox (0.2 μg/mL) and 3-MA (3-methyladenine, 5 mM) was significantly higher than that in cells treated with Dox (0.2 μg/mL) alone and was close to the apoptosis of the control cells (Figure 4A). Western blot analysis showed that LC3 accumulation was significantly increased in SESN2-overexpressing cells treated with Cis (20 μmol/L) and Dox (0.2 μg/mL) (Figure 4B). In addition, we treated HOS cells transduced with control or SESN2-overexpressing pHBLV with 3-MA (5 mM) and found that autophagy enhancement due to SESN2 overexpression was inhibited by 3-MA (5 mM) (Figure 4C). To investigate the role of SESN2 in autophagy-mediated apoptosis, we treated SESN2-overexpressing and control cells with 3-MA (5 mM) and chemotherapeutic agents and observed that apoptosis of the SESN2-overexpressing HOS cells was increased and that the protective effect of SESN2 on osteosarcoma cells was decreased after 3-MA (5 mM) treatment (Figure 4D). The submicroscopic structure of autophagosomes was observed by TEM. The results indicated that the number and size of autophagosomes in the SESN2 overexpression group were significantly increased after Dox (0.2 μg/mL) treatment, but this chemotherapy-induced autophagy was inhibited by 3-MA (5 mM) (Figure 4E). The same results were confirmed by immunofluorescence, which showed that the accumulation of LC3 in HOS cells induced by chemotherapeutic drugs in the SESN2 overexpression group was inhibited by 3-MA (5 mM) (Figure 4F). In addition, we observed autophagic flux through mRFP-GFP-LC3 adenovirus transfection and found that the SESN2-mediated autophagic flux of HOS cells was significantly enhanced after treatment with Cis (20 μmol/L) and Dox (0.2 μg/mL) (Figure 4G). These experimental results indicate that SESN2-mediated autophagy reduces the sensitivity of osteosarcoma cells to chemotherapy drugs, leading to the occurrence of drug resistance.

### Sestrin2 Promotes Autophagy and Inhibits Apoptosis of Osteosarcoma Cells Through Endoplasmic Reticulum Stress

**FIGURE 5:**
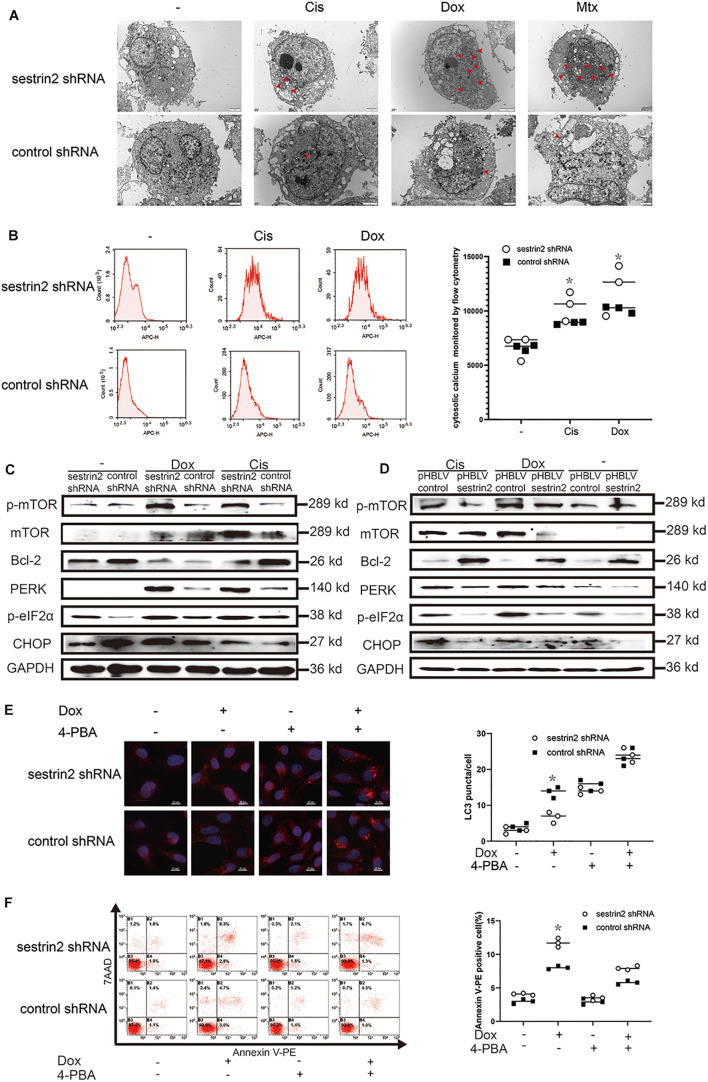
SESN2 promotes autophagy and inhibits apoptosis through endoplasmic reticulum (ER) stress. After cells were treated with Cis (20 μmol/L), Dox (0.2 μg/mL), or Mtx (50 μmol/L) for 24 h, ER stress in SESN2-knockdown HOS cells was observed by TEM **(A)** (*n* = 3, scale bar = 2 μm). After cells were treated with Cis (20 μmol/L) and Dox (0.2 μg/mL) for 24 h, intracellular calcium in SESN2-knockdown and control HOS cells was monitored by flow cytometry **(B)** (*n* = 3). The protein expression levels of genes involved in apoptosis-, autophagy- and ER stress-related pathways in HOS cells with upregulated and downregulated SESN2 expression were detected by western blot **(C,D)** (*n* = 3). Twenty-four hours after Dox (0.2 μg/mL) treatment, the LC3 puncta in HOS cells from the SESN2-knockdown group and the control group were detected by immunofluorescence, and apoptosis was analysed by flow cytometry in the presence and absence of 4-PBA (5 mM) **(E,F)** (*n* = 3, scale bar = 20 μm). The data are presented as the mean ± SD. *p < 0.05 vs. the Control shRNA group.

We have previously shown that Cis (20 μmol/L), Dox (0.2 μg/mL), and Mtx (50 μmol/L) inhibit the growth of human osteosarcoma cells, but the underlying mechanisms have not been investigated. In addition, electron microscopy revealed that these three chemotherapeutic agents induced osteosarcoma cell vacuolation (Figure 5A). Compared with cells in the control group, HOS cells in the SESN2 knockdown group were more exposed to expanded cytoplasmic vacuolation groups after chemotherapeutic treatment. The expansion of cytoplasmic vacuolation is considered to be an expanded endoplasmic cavity, indicating increased endoplasmic stress. To further explain this phenomenon, we evaluated chemotherapy-treated HOS cells using flow cytometry. Calcium homeostasis is an important function of the ER, and calcium dysregulation leads to ER stress. In this study, Fura-Red-AM was used to indicate cytoplasmic calcium. The results showed that the cytoplasmic calcium level was significantly increased in the chemotherapy-treated HOS cells, and the Ca^2+^ content in the SESN2-knockdown group was significantly higher than that in the control group (Figure 5B). We further demonstrated that the ER stress pathway PERK-eIF2α-CHOP was significantly activated by chemotherapy treatment, and western blot results also demonstrated that low SESN2 expression led to activation of PERK-eIF2α-CHOP. Moreover, upregulated SESN2 expression decreased the expression of ER stress-related proteins in osteosarcoma cells treated with chemotherapy drugs (Figures 5C,D). These interesting findings strongly suggest that chemotherapeutic agents can induce ER stress in human osteosarcoma cell lines. To investigate whether osteosarcoma cells inhibit apoptosis by regulating ER stress to promote autophagy, we treated osteosarcoma cells with chemotherapeutic agents and showed that these treated cells had significant LC3 accumulation compared with that in cells with SESN2 knockdown, and this gap was reduced by 5mM of 4-phenylbutyric acid (4-PBA) (Sigma Aldrich, St. Louis, Missouri, United States) (Figure 5E). We then used flow cytometry to investigate the relationship between ER stress and apoptosis and found that 4-PBA (5 mM) reduced chemotherapy-induced apoptosis in osteosarcoma cells with SESN2 knockdown (Figure 5F).

### Mouse Xenograft Models Demonstrate That Inhibition of Sestrin2 Reduces Autophagy to Increase Apoptosis and Thus Increases the Sensitivity of Osteosarcoma to Chemotherapeutic Agents

**FIGURE 6:**
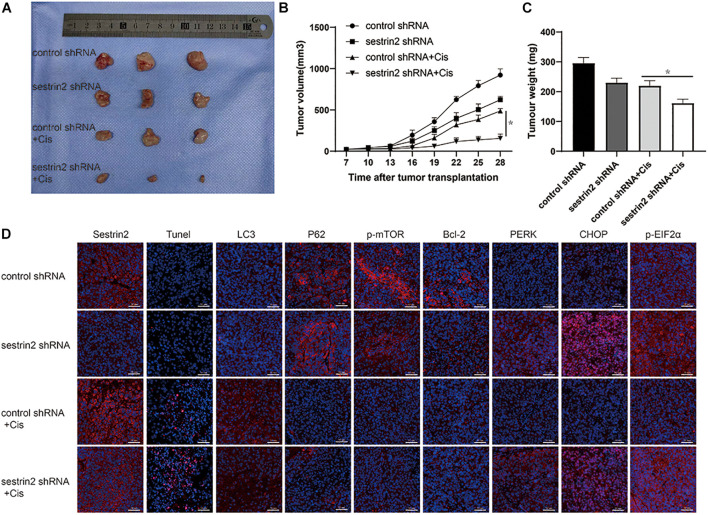
Suppression of SESN2 increases sensitivity to chemotherapy *in vivo*. NU/NU nude mice were subcutaneously injected with SESN2-knockdown or control 143B cells. After 7 days, the mice were intraperitoneally injected with Cis (20 μmol/L) until they were killed on day 28. Tumour volume was measured on day 28 **(A)** (*n* = 3). Tumour growth curves were measured from days 7 to 28 **(B)** (*n* = 3). Tumour weight was measured on day 28 **(C)** (*n* = 3). Immunofluorescence staining of TUNEL, SESN2 expression, LC3 expression, P62 expression, p-mTOR expression, Bcl-2 expression, PERK expression, CHOP expression and p-eIF2α expression after tumour sample injection **(D)** (*n* = 3, scale bar = 50 μm). The data are presented as the mean ± SD. *p < 0.05 vs. the Control shRNA + Cis group.

To investigate the relationship between SESN2 and chemotherapeutic sensitivity and the potential mechanism *in vivo*, 143B cells transfected with SESN2 shRNA or control shRNA were subcutaneously injected into NU/NU nude mice, and tumour growth was observed periodically. The nude mice were intraperitoneally injected with chemotherapeutic agents twice every seven days, starting from day 8 until day 28. And tumour size was measured every 3 days until day 28. We found that SESN2 knockdown significantly slowed tumour growth in response to chemotherapeutic agents (Figure 6A); the tumour volume and weight are significantly decreased in the SESN2 shRNA + Cis group compared with those in the control shRNA + Cis group (Figures 6B,C). TUNEL staining showed that SESN2 knockdown induced more apoptosis in osteosarcoma cells after chemotherapy treatment compared to that in control cells subjected to chemotherapy treatment (Figure 6D). We also found that compared to control cells, SESN2-knockdown cells exhibited decreased autophagy and activated ER stress in the presence of chemotherapeutic agents (Figure 6D). These results also confirm that SESN2 regulates osteosarcoma cell apoptosis through autophagy, thereby mediating their drug resistance.

## Discussion

Osteosarcoma is a malignant tumour with a very high mortality rate in adolescents, and available effective treatments are very limited. Neoadjuvant chemotherapy can prolong the survival of patients, but the resistance of osteosarcoma to chemotherapy drugs is still a major problem today (Li and Ma, 2021; Su et al., 2021). Cis, Dox, and Mtx are the current routine chemotherapy drugs for osteosarcoma in clinical practice, and the decreased sensitivity of osteosarcoma to these drugs leads to the occurrence of drug resistance, which is the main challenge to improve the overall survival of patients (Wang Y. et al., 2018; Xiao et al., 2018; Chen R. et al., 2019). In this paper, the occurrence of drug resistance in osteosarcoma treated with Cis, Dox, and Mtx aroused our interest in the potential molecular mechanism. Autophagy is one of many mechanisms involved in the development of drug resistance in tumours and is widely considered a cellular response under stress conditions. However, it has two sides—autophagy can protect cells from apoptosis caused by external stress conditions, but excessive autophagy can induce cell death. In a study of the mechanism of drug resistance in osteosarcoma, we found that chemotherapy drug treatment stimulated autophagy to protect cells and reduce apoptosis, thus leading to drug resistance (Niu et al., 2019).

Sestrin2 has been identified as a protein that induces DNA damage and oxidative stress by inhibiting mammalian targets of rapamycin complex 1 while accelerating autophagy (Wang et al., 2017; Jeong et al., 2019). It has been reported that SESN2 can cooperate with P62 to promote autophagy-driven degradation (Hua et al., 2018). We found an association between SESN2 and autophagy pathways and observed that SESN2 inhibited the ER stress signalling pathway PERK-eIF2α-CHOP following treatment with chemotherapy, thereby upregulating autophagy in osteosarcoma cells, which was accompanied by a slight decrease in P62. Consistent with these findings, we provided a schematic diagram of signalling axis regulation that plays a role in chemotherapy drugs-induced SESN2-dependent apoptosis in osteosarcoma cells.

It has been reported that many antineoplastic drugs activate various mechanisms of cell death and regulate ER homeostasis by inducing undeveloped protein response (UPR)-mediated ER stress in cancer cells (Chiu et al., 2015; Cheng et al., 2018). Endoplasmic reticulum homeostasis is regulated by the maintenance of intracellular calcium balance (Cheng et al., 2018). Depending on the severity of stress, cancer cells decide to resume protein unfolding or activate the cell death mechanism through autophagy or apoptosis. ER stress is a double-edged sword that plays an important role in regulating the pro-death or pro-survival signals of solid tumour cells (White-Gilbertson et al., 2013). In this paper, we found that failure to resolve ER stress activates two interrelated biological processes: autophagy and apoptosis. Inhibition of endoplasmic reticulum stress in the presence of chemotherapeutic agents reduced PERK-mediated phosphorylation of eIF2α, thus further inhibiting the activation of CHOP related transcription factors. On the one hand, this leaded to the occurrence of autophagy, which degraded the accumulation of misfolded proteins, played a protective role in osteosarcoma cells and reduced the apoptosis of osteosarcoma cells. This process is accompanied by an increase in mTOR phosphorylation. On the other hand, the inhibition of endoplasmic reticulum stress accompanied by the increase of Bcl-2 lead to the inhibition of osteosarcoma cell apoptosis. Therefore, we found that the mechanism of drug resistance in osteosarcoma cells is bidirectional.

Consistent with recent data, the present study exerts synergistic antitumour effects by activating ER stress-mediated autophagy and apoptosis. Subsequent analysis showed that chemotherapy treatment of SESN2-knockdown osteosarcoma cells not only caused intracytoplasmic calcium accumulation and ER swelling and vacuolisation but also enhanced PERK-mediated phosphorylation of eIF2α (Liu et al., 2019). Through observation, we found that SESN2 leads to a decrease in p-mTOR protein levels by reducing CHOP expression, which may be related to chemotherapy-mediated autophagy. This suggestion was further substantiated by the data, revealing that autophagy is associated with dysregulated response to ER stress. The correlation between the expression of mTOR and SESN2 was determined by SESN2 knockout. We found that low SESN2 expression led to upregulation of p-mTOR expression, autophagy inactivation and downregulation of Bcl-2. We suggest that chemotherapeutic agents induce autophagy in a SESN2-dependent manner by downregulating mTOR phosphorylation, which further promotes the inhibition of chemotherapeutic drug-induced apoptosis of osteosarcoma cells. The ER stress inhibitor 4-PBA reduced the apoptosis of osteosarcoma cells after chemotherapeutic treatment to reduce the sensitivity of osteosarcoma cells to chemotherapy drugs. In addition, treatment with 4-PBA showed that chemotherapy-induced autophagy and apoptosis operated via the PERK-eIF2α-CHOP axis of the ER stress response.

## Conclusion

**FIGURE 7:**
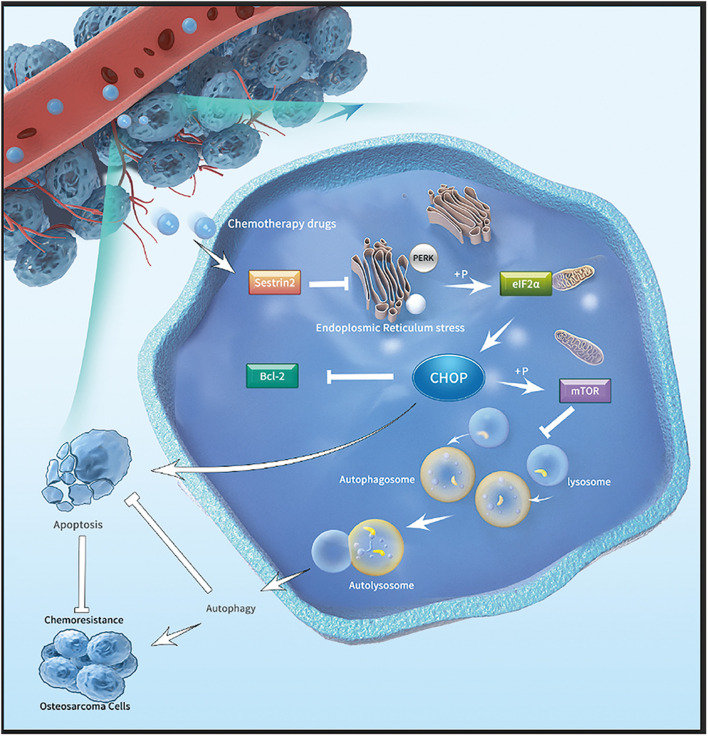
Mechanistic model depicting the effects of SESN2 on the drug resistance of osteosarcoma. After chemotherapy drugs are applied to osteosarcoma cells, SESN2 expression is increased, and the phosphorylation of eIF2α by PERK during ER stress is inhibited. Finally, the expression of CHOP is suppressed, and apoptosis and autophagy are regulated by Bcl-2 and mTOR, respectively, thus causing drug resistance in osteosarcoma cells.

In summary, our data show that SESN2 expression in osteosarcoma cell lines is increased in response to chemotherapeutic agents. SESN2 regulated autophagy and reduced apoptosis via the PERK-eIF2α-CHOP signalling pathway of ER stress and eventually led to drug resistance. In addition, knockdown of SESN2 decreased autophagy and increased apoptosis, which corresponds to increased sensitivity of osteosarcoma cells to chemotherapeutic agents (Figure 7). Therefore, our results suggest that SESN2 is a novel therapeutic target for drug resistance in osteosarcoma.

## Data Availability Statement

The raw data supporting the conclusions of this article will be made available by the authors, without undue reservation.

## Ethics Statement

The animal study was reviewed and approved by Ethics Committee of the Fourth Military Medical University.

## Author Contributions

ZT, XX, and ZG studied concept and design. ZT, XW, HW, HD, YL, WW, XX, XL, and LS acquired data. ZT and XW analyzed data. ZT and XX drafted the manuscript. ZT, XW, HW, HD, and YL provided acquisition, analysis and interpretation of data, and statistical analysis. ZT and FW made substantial revision to this manuscript. All authors read and approved the final manuscript.

## Conflict of Interest

The authors declare that the research was conducted in the absence of any commercial or financial relationships that could be construed as a potential conflict of interest.

## Publisher’s Note

All claims expressed in this article are solely those of the authors and do not necessarily represent those of their affiliated organizations, or those of the publisher, the editors and the reviewers. Any product that may be evaluated in this article, or claim that may be made by its manufacturer, is not guaranteed or endorsed by the publisher.

## Supplementary Material

Supplementary Figure 1 After MG-63 cells were treated with Cis (20 μmol/L), Dox (0.2 μg/mL) or Mtx (50 μmol/L) for 24 h, the apoptosis of cells transfected with pHBLV control or pHBLV sestrin2 and cultured in the presence or absence of ZVAD-FMK (20 μmol/L) was detected using the Caspase 3 kit (F) (*n* = 3).

Supplementary Figure 2 After MG-63 cells were treated with Cis (20 μmol/L), Dox (0.2 μg/mL) or Mtx (50 μmol/L) for 24 h, the expression of Cleaved-Caspase 3 transfected with pHBLV control or pHBLV sestrin2 and sestrin2 shRNA or control shRNA, and cultured in the presence or absence of ZVAD-FMK (20 μmol/L) was detected by western blot (F) (*n* = 3).

Supplementary Figure 3 MG-63 and HOS cells were transfected with pHBLV control or pHBLV sestrin2, and the transfection effect was detected by quantitative real-time PCR (*n* = 3).

Supplementary Figure 4 SESN2-knockdown HOS cells were treated with Cis (20 μmol/L) for 24 h with or without rapamycin (100 nmol/L) for 6 h. Proliferation was analysed by CCK-8 assay (*n* = 3).

Supplementary Figure 5 The red arrows are autophagosomes in Figures 3D, 4E, 5A.

## Abbreviations

SESN2: Sestrin2
ER: endoplasmic reticulum
ROS: reactive oxygen species
Cis: cisplatin
Dox: doxorubicin
Mtx: methotrexate
RIPA: radioimmunoprecipitation assay
TEM: Transmission electron microscopy
CCK8: Cell Counting Kit-8.

## References

[B1] Aleksakhina S. N. Kashyap A. Imyanitov E. N. (2019). Mechanisms of acquired tumor drug resistance. *Biochim. Biophys. Acta Rev. Cancer* 1872:188310. 10.1016/j.bbcan.2019.188310 31442474

[B2] Bruning A. Rahmeh M. Friese K. (2013). Nelfinavir and bortezomib inhibit mTOR activity via ATF4-mediated sestrin-2 regulation. *Mol. Oncol.* 7 1012–1018. 10.1016/j.molonc.2013.07.010 23916134 PMC5528439

[B3] Burns J. Wilding C. P. Jones R. L. Huang P. H. (2020). Proteomic research in sarcomas–current status and future opportunities. *Semin Cancer Biol.* 61 56–70. 10.1016/j.semcancer.2019.11.003 31722230 PMC7083238

[B4] Chen J. Liu G. Wu Y. Ma J. Wu H. Xie Z. (2019). CircMYO10 promotes osteosarcoma progression by regulating miR-370-3p/RUVBL1 axis to enhance the transcriptional activity of beta-catenin/LEF1 complex via effects on chromatin remodeling. *Mol. Cancer* 18:150. 10.1186/s12943-019-1076-1 31665067 PMC6819556

[B5] Chen R. Wang G. Zheng Y. Hua Y. Cai Z. (2019). Drug resistance-related microRNAs in osteosarcoma: translating basic evidence into therapeutic strategies. *J. Cell. Mol. Med.* 23 2280–2292. 10.1111/jcmm.14064 30724027 PMC6433687

[B6] Cheng X. Feng H. Wu H. Jin Z. Shen X. Kuang J. (2018). Targeting autophagy enhances apatinib-induced apoptosis via endoplasmic reticulum stress for human colorectal cancer. *Cancer Lett.* 431 105–114. 10.1016/j.canlet.2018.05.046 29859300

[B7] Chiu H. W. Tseng Y. C. Hsu Y. H. Lin Y. F. Foo N. P. Guo H. R. (2015). Arsenic trioxide induces programmed cell death through stimulation of ER stress and inhibition of the ubiquitin-proteasome system in human sarcoma cells. *Cancer Lett.* 356(2 Pt B) 762–772. 10.1016/j.canlet.2014.10.025 25449439

[B8] Crisol B. M. Lenhare L. Gaspar R. S. Gaspar R. C. Munoz V. R. da Silva A. S. R. (2018). The role of physical exercise on Sestrin1 and 2 accumulations in the skeletal muscle of mice. *Life Sci.* 194 98–103. 10.1016/j.lfs.2017.12.023 29273527

[B9] Dai J. Huang Q. Niu K. Wang B. Li Y. Dai C. (2018). Sestrin 2 confers primary resistance to sorafenib by simultaneously activating AKT and AMPK in hepatocellular carcinoma. *Cancer Med.* 7 5691–5703. 10.1002/cam4.1826 30311444 PMC6247041

[B10] Dodson M. Darley-Usmar V. Zhang J. (2013). Cellular metabolic and autophagic pathways: traffic control by redox signaling. *Free Radic. Biol. Med.* 63 207–221. 10.1016/j.freeradbiomed.2013.05.014 23702245 PMC3729625

[B11] Gu Z. Hou Z. Zheng L. Wang X. Wu L. Zhang C. (2018). LncRNA DICER1-AS1 promotes the proliferation, invasion and autophagy of osteosarcoma cells via miR-30b/ATG5. *Biomed. Pharmacother.* 104 110–118. 10.1016/j.biopha.2018.04.193 29772430

[B12] Hattori T. Takahashi Y. Chen L. Tang Z. Wills C. A. Liang X. (2021). Targeting the ESCRT-III component CHMP2A for noncanonical Caspase-8 activation on autophagosomal membranes. *Cell Death Differ.* 28 657–670. 10.1038/s41418-020-00610-0 32807832 PMC7862398

[B13] Hua X. Xu J. Deng X. Xu J. Li J. Zhu D. Q. (2018). New compound ChlA-F induces autophagy-dependent anti-cancer effect via upregulating Sestrin-2 in human bladder cancer. *Cancer Lett.* 436 38–51. 10.1016/j.canlet.2018.08.013 30118841 PMC6245652

[B14] Huang K. Chen Y. Zhang R. Wu Y. Ma Y. Fang X. (2018). Honokiol induces apoptosis and autophagy via the ROS/ERK1/2 signaling pathway in human osteosarcoma cells in vitro and in vivo. *Cell Death Dis.* 9:157. 10.1038/s41419-017-0166-5 29410403 PMC5833587

[B15] Jeong S. Kim D. Y. Kang S. H. Yun H. K. Kim J. L. Kim B. R. (2019). Docosahexaenoic acid enhances oxaliplatin-induced autophagic cell death via the ER Stress/Sesn2 pathway in colorectal cancer. *Cancers (Basel)* 11:982. 10.3390/cancers11070982 31337142 PMC6678695

[B16] Khan M. A. Vikramdeo K. S. Sudan S. K. Singh S. Wilhite A. Dasgupta S. (2021). Platinum-resistant ovarian cancer: from drug resistance mechanisms to liquid biopsy-based biomarkers for disease management. *Semin Cancer Biol.* 10.1016/j.semcancer.2021.08.005 34418576 PMC8665066

[B17] Kimball S. R. Gordon B. S. Moyer J. E. Dennis M. D. Jefferson L. S. (2016). Leucine induced dephosphorylation of Sestrin2 promotes mTORC1 activation. *Cell Signal.* 28 896–906. 10.1016/j.cellsig.2016.03.008 27010498 PMC4899281

[B18] Lear T. B. Lockwood K. C. Ouyang Y. Evankovich J. W. Larsen M. B. Lin B. (2019). The ring-type E3 ligase RNF186 ubiquitinates Sestrin-2 and thereby controls nutrient sensing. *J. Biol. Chem.* 294 16527–16534. 10.1074/jbc.AC119.010671 31586034 PMC6851335

[B19] Li M. Ma W. (2021). miR-26a reverses multidrug resistance in osteosarcoma by targeting MCL1. *Front. Cell. Dev. Biol.* 9:645381. 10.3389/fcell.2021.645381 33816494 PMC8012539

[B20] Li T. Mu N. Yin Y. Yu L. Ma H. (2020a). Targeting AMP-activated protein kinase in aging-related cardiovascular diseases. *Aging Dis.* 11 967–977. 10.14336/AD.2019.0901 32765957 PMC7390518

[B21] Li T. Yin Y. Mu N. Wang Y. Liu M. Chen M. (2020b). Metformin-enhanced cardiac AMP-activated protein kinase/atrogin-1 pathways inhibit charged multivesicular body protein 2B accumulation in ischemia-reperfusion injury. *Front. Cell. Dev. Biol.* 8:621509. 10.3389/fcell.2020.621509 33614629 PMC7892907

[B22] Liu S. Lin H. Wang D. Li Q. Luo H. Li G. (2019). PCDH17 increases the sensitivity of colorectal cancer to 5-fluorouracil treatment by inducing apoptosis and autophagic cell death. *Signal. Transduct. Target Ther.* 4:53. 10.1038/s41392-019-0087-0 31815010 PMC6882894

[B23] Liu W. Jiang D. Gong F. Huang Y. Luo Y. Rong Y. (2020). miR-210-5p promotes epithelial-mesenchymal transition by inhibiting PIK3R5 thereby activating oncogenic autophagy in osteosarcoma cells. *Cell Death Dis.* 11:93. 10.1038/s41419-020-2270-1 32024814 PMC7002725

[B24] Liu Z. Wang H. Hu C. Wu C. Wang J. Hu F. (2021). Targeting autophagy enhances atezolizumab-induced mitochondria-related apoptosis in osteosarcoma. *Cell Death Dis.* 12:164. 10.1038/s41419-021-03449-6 33558476 PMC7870858

[B25] Mrakovcic M. Frohlich L. F. (2019). Molecular determinants of cancer therapy resistance to HDAC inhibitor-induced autophagy. *Cancers (Basel)* 12:109. 10.3390/cancers12010109 31906235 PMC7016854

[B26] Niu J. Yan T. Guo W. Wang W. Zhao Z. (2019). Insight into the role of autophagy in osteosarcoma and its therapeutic implication. *Front. Oncol.* 9:1232. 10.3389/fonc.2019.01232 31803616 PMC6873391

[B27] Pan Z. Cheng D. D. Wei X. J. Li S. J. Guo H. Yang Q. C. (2021). Chitooligosaccharides inhibit tumor progression and induce autophagy through the activation of the p53/mTOR pathway in osteosarcoma. *Carbohydr. Polym.* 258:117596. 10.1016/j.carbpol.2020.117596 33593530

[B28] Pantziarka P. Verbaanderd C. Huys I. Bouche G. Meheus L. (2021). Repurposing drugs in oncology: from candidate selection to clinical adoption. *Semin Cancer Biol.* 68 186–191. 10.1016/j.semcancer.2020.01.008 31982510

[B29] Patra S. Pradhan B. Nayak R. Behera C. Rout L. Jena M. (2021). Chemotherapeutic efficacy of curcumin and resveratrol against cancer: chemoprevention, chemoprotection, drug synergism and clinical pharmacokinetics. *Semin Cancer Biol.* 73 310–320. 10.1016/j.semcancer.2020.10.010 33152486

[B30] Peixoto da Silva S. Caires H. R. Bergantim R. Guimaraes J. E. Vasconcelos M. H. (2021). miRNAs mediated drug resistance in hematological malignancies. *Semin Cancer Biol.* 10.1016/j.semcancer.2021.03.014 33757848

[B31] Sanchez-Alvarez M. Strippoli R. Donadelli M. Bazhin A. V. Cordani M. (2019). Sestrins as a therapeutic bridge between ROS and autophagy in cancer. *Cancers (Basel)* 11:1415. 10.3390/cancers11101415 31546746 PMC6827145

[B32] Su X. Zhang X. Liu W. Yang X. An N. Yang F. (2021). Advances in the application of nanotechnology in reducing cardiotoxicity induced by cancer chemotherapy. *Semin Cancer Biol.* 10.1016/j.semcancer.2021.08.003 34375726

[B33] Wang M. Xu Y. Liu J. Ye J. Yuan W. Jiang H. (2017). Recent insights into the biological functions of sestrins in health and disease. *Cell Physiol. Biochem.* 43 1731–1741. 10.1159/000484060 29050006

[B34] Wang W. Chen D. Zhu K. (2018). SOX2OT variant 7 contributes to the synergistic interaction between EGCG and Doxorubicin to kill osteosarcoma via autophagy and stemness inhibition. *J. Exp. Clin. Cancer Res.* 37:37. 10.1186/s13046-018-0689-3 29475441 PMC6389193

[B35] Wang Y. Deng X. Yu C. Zhao G. Zhou J. Zhang G. (2018). Synergistic inhibitory effects of capsaicin combined with cisplatin on human osteosarcoma in culture and in xenografts. *J. Exp. Clin. Cancer Res.* 37:251. 10.1186/s13046-018-0922-0 30326933 PMC6192127

[B36] Wang Z. Yin F. Xu J. Zhang T. Wang G. Mao M. (2019). CYT997(Lexibulin) induces apoptosis and autophagy through the activation of mutually reinforced ER stress and ROS in osteosarcoma. *J. Exp. Clin. Cancer Res.* 38:44. 10.1186/s13046-019-1047-9 30704503 PMC6357486

[B37] White-Gilbertson S. Hua Y. Liu B. (2013). The role of endoplasmic reticulum stress in maintaining and targeting multiple myeloma: a double-edged sword of adaptation and apoptosis. *Front. Genet.* 4:109. 10.3389/fgene.2013.00109 23781234 PMC3678081

[B38] Xiao X. Wang W. Li Y. Yang D. Li X. Shen C. (2018). HSP90AA1-mediated autophagy promotes drug resistance in osteosarcoma. *J. Exp. Clin. Cancer Res.* 37:201. 10.1186/s13046-018-0880-6 30153855 PMC6114771

[B39] Ye J. Palm W. Peng M. King B. Lindsten T. Li M. O. (2015). GCN2 sustains mTORC1 suppression upon amino acid deprivation by inducing Sestrin2. *Genes Dev.* 29 2331–2336. 10.1101/gad.269324.115 26543160 PMC4691887

[B40] Yen J. H. Huang S. T. Huang H. S. Fong Y. C. Wu Y. Y. Chiang J. H. (2018). HGK-sestrin 2 signaling-mediated autophagy contributes to antitumor efficacy of Tanshinone IIA in human osteosarcoma cells. *Cell Death Dis.* 9:1003. 10.1038/s41419-018-1016-9 30258193 PMC6158215

[B41] Zhu G. Xu P. Guo S. Yi X. Wang H. Yang Y. (2020). Metastatic melanoma cells rely on Sestrin2 to acquire anoikis resistance via detoxifying intracellular ROS. *J. Investig. Dermatol.* 140 666–675.e2. 10.1016/j.jid.2019.07.720 31476315

