# Corrigendum: Sestrin2-mediated autophagy contributes to drug resistance *via* endoplasmic reticulum stress in human osteosarcoma

**DOI:** 10.3389/fcell.2022.882270

**Published:** 2022-10-11

**Authors:** Zhen Tang, Xinghui Wei, Tian Li, Wei Wang, Hao Wu, Hui Dong, Yichao Liu, Feilong Wei, Lei Shi, Xiaokang Li, Zheng Guo, Xin Xiao

**Affiliations:** ^1^ Department of Orthopaedics, Xijing Hospital, Fourth Military Medical University, Xi’an, China; ^2^ School of Basic Medicine, Fourth Military Medical University, Xi’an, China; ^3^ State Key Laboratory of Cancer Biology, Department of Immunology, Fourth Military Medical University, Xi’an, China; ^4^ Department of Orthopaedics, Tangdu Hospital, Fourth Military Medical University, Xi’an, China

**Keywords:** Sestrin2, apoptosis, autophagy, drug resistance, endoplasmic reticulum stress

In the original article, there was a mistake in Figures 3D,E, 4E as published. The transmission electron microscopy shown in Figures 3D, 4E, and immunofluorescence in Figure 3E were mistakenly used.

**FIGURE 3:**
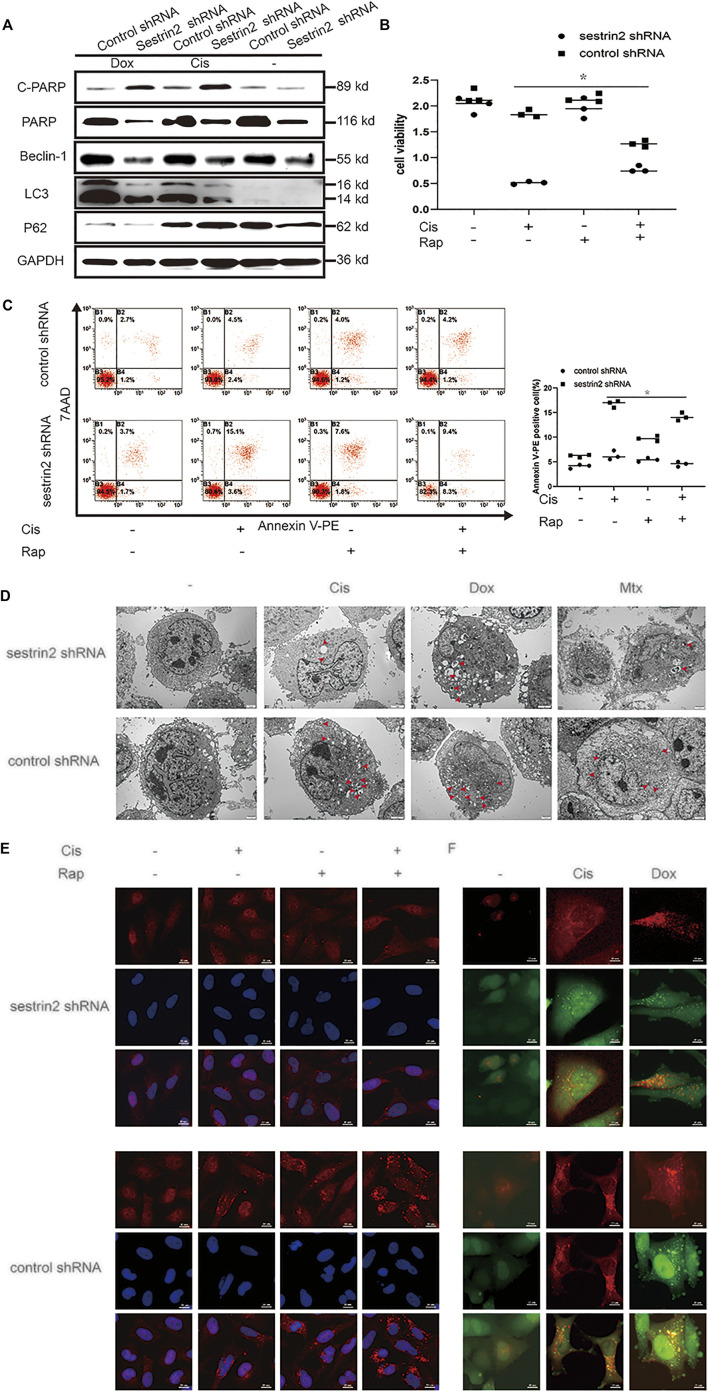
Knockdown of SESN2 resulted in inhibited autophagy and increased apoptosis of osteosarcoma cells treated with chemotherapy. After treatment with Cis (20 μmol/L), Dox (0.2 μg/ml), or Mtx (50 μmol/L) for 24 h, SESN2-knockdown and control cells were subjected to western blot to detect the expression of cleaved and total PARP, LC3, and P62 expression levels **(A)** (*n* = 3). SESN2-knockdown HOS cells were treated with Cis (20 μmol/L) for 24 h with or without rapamycin (100 nmol/L) for 6 h. Proliferation was analysed by CCK-8 assay **(B)** (*n* = 3), apoptosis was assessed by Annexin V-PE/PI staining **(C)** (*n* = 3), and LC3 puncta formation was analysed by immunofluorescence **(E)** (*n* = 3, scale bar = 20 µm). Intracellular autophagosomes were observed by TEM **(D)** (*n* = 3, scale bar = 2 µm), and autophagic flux was monitored by fluorescence microscopy in HOS cells with transient expression of GFP-RFP-LC3 in HOS cells **(F)** (*n* = 3, scale bar = 10 µm). The data are presented as the mean ± SD. *p < 0.05 vs. the Control shRNA group.

**FIGURE 4:**
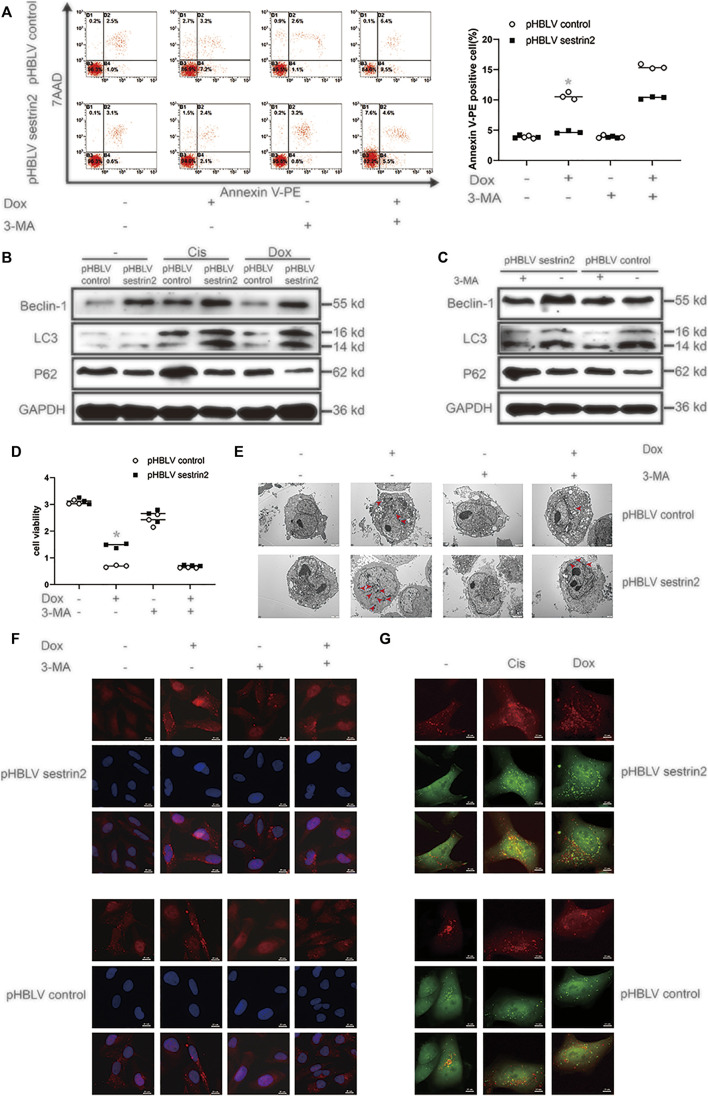
SESN2 regulates autophagy and reduces the sensitivity of osteosarcoma cells to chemotherapy. In the presence or absence of 3-MA (5 mM), SESN2-overexpressing and control HOS cells were treated with Dox (0.2 μg/ml) for 24 h, and apoptosis was analysed by flow cytometry **(A)** (*n* = 3). After treatment with Cis (20 μmol/L) or Dox (0.2 μg/ml), the expression levels of LC3 and P62 in SESN2-overexpressing and control HOS cells were detected by western blot **(B)** (*n* = 3). The expression levels of LC3 and P62 in SESN2-overexpressing and control HOS cells treated with 3-MA (5 mM) were detected by western blot **(C)** (*n* = 3). In the presence or absence of 3-MA (5 mM), SESN2-overexpressing and control HOS cells were treated with Dox (0.2 μg/ml) for 24 h, cell activity was detected by CCK-8 **(D)** (*n* = 3), intracellular autophagosomes were observed by TEM **(E)** (*n* = 3, scale bar = 2 µm), and intracellular LC3 puncta formation was analysed by immunofluorescence **(F)** (*n* = 3, scale bar = 20 µm). After treatment with Cis (20 μmol/L) or Dox (0.2 μg/ml), autophagosome formation in HOS cells with ectopic SESN2 expression was monitored by immunofluorescence through transfection with RFP-GFP-LC3 lentivirus after upregulating SESN2 **(G)** (*n* = 3, scale bar = 10 µm). The data are presented as the mean ± SD. *p < 0.05 vs. the pHBLV control group.

The red arrows in the figure legends of Figures 3, 4 are autophagosomes.

The authors apologize for this error and state that this does not change the scientific conclusions of the article in any way. The original article has been updated.

