# Supplementary material for: Sestrin2-Mediated Autophagy Contributes to Drug Resistance via Endoplasmic Reticulum Stress in Human Osteosarcoma
Source: Front Cell Dev Biol. 2021 Sep 27;9:722960. doi: 10.3389/fcell.2021.722960 (PMC8502982; doi:10.3389/fcell.2021.722960)
Supplement: Supplementary file 1 [file Data_Sheet_2.ZIP › Raw data of immunofluorescence in vitro/Raw data of immunofluorescence in vitro.pptx]

## Slide 1
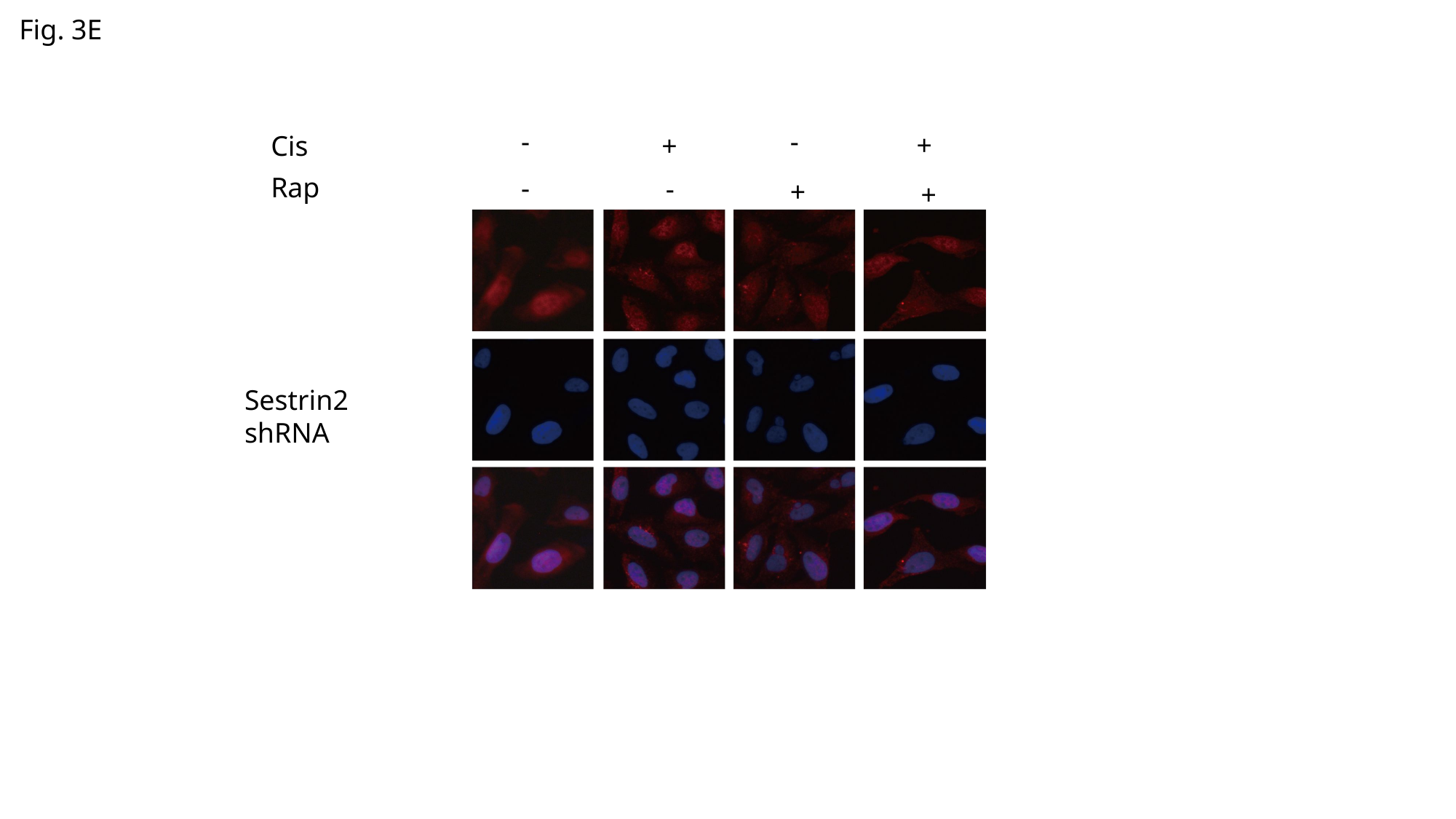

Fig. 3E
-
-
+
+
Cis
Rap
-
-
+
+
Sestrin2 shRNA

## Slide 2
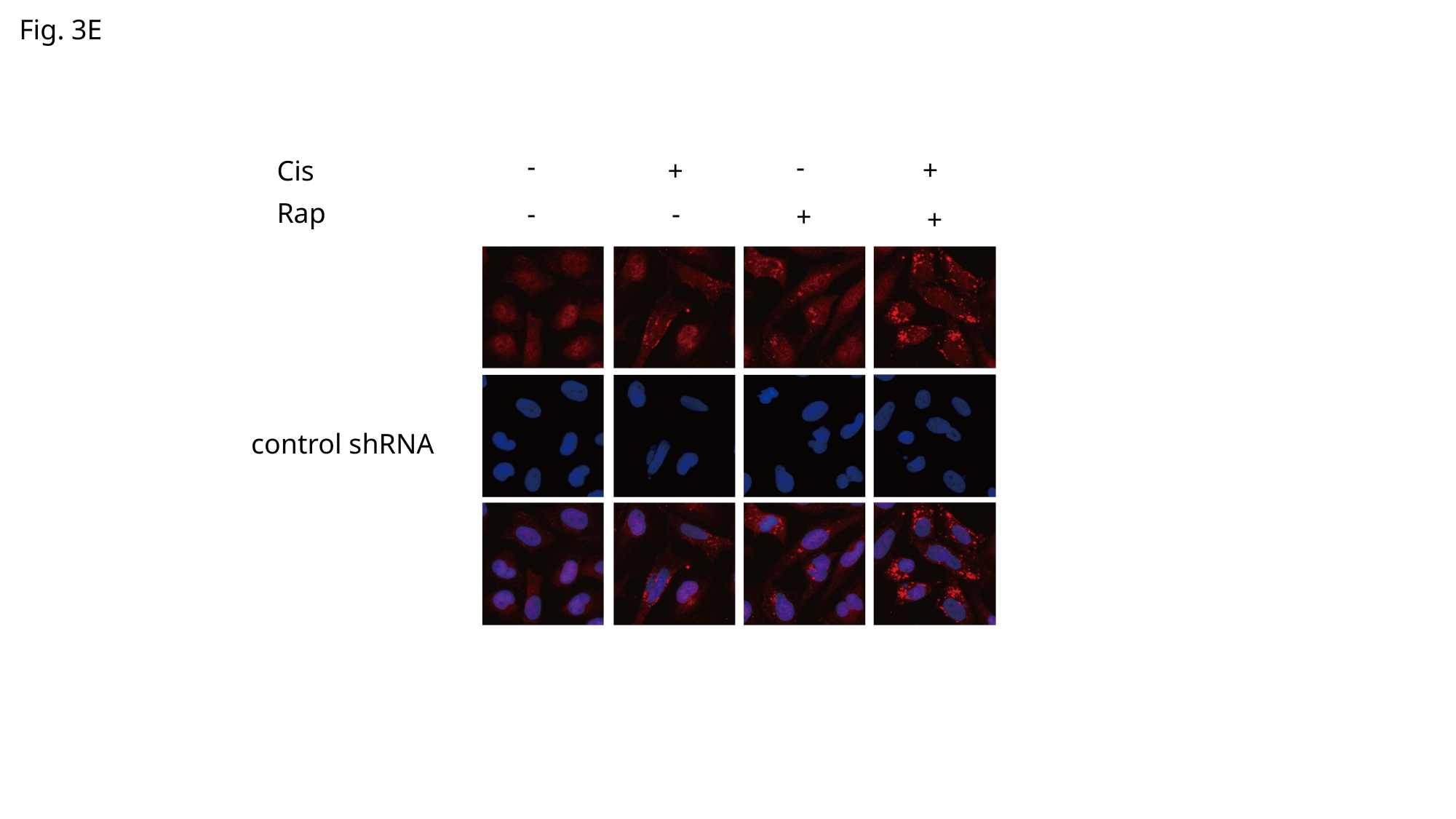

Fig. 3E
-
-
+
+
Cis
Rap
-
-
+
+
control shRNA

## Slide 3
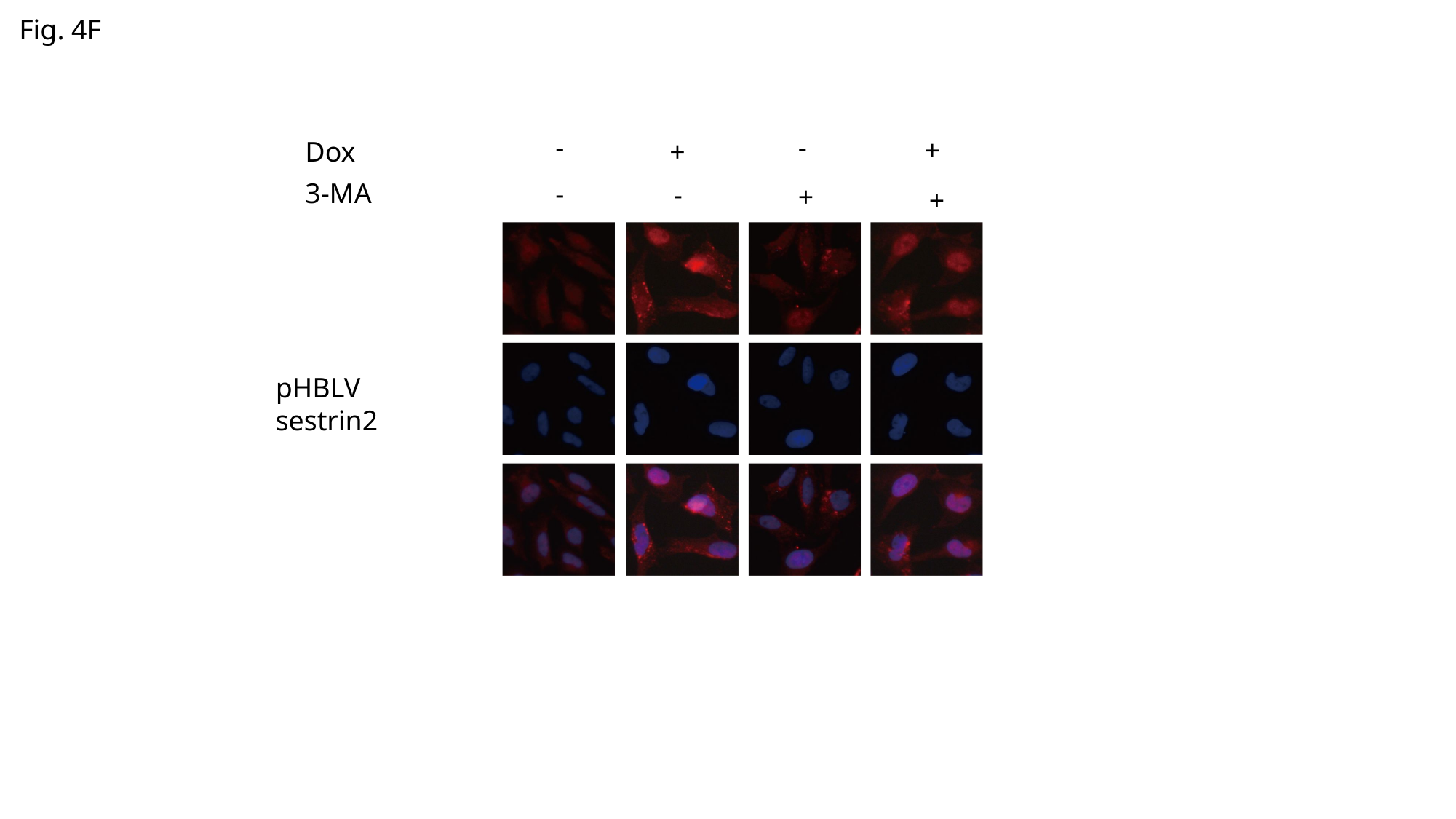

Fig. 4F
-
-
+
+
Dox
3-MA
-
-
+
+
pHBLV sestrin2

## Slide 4
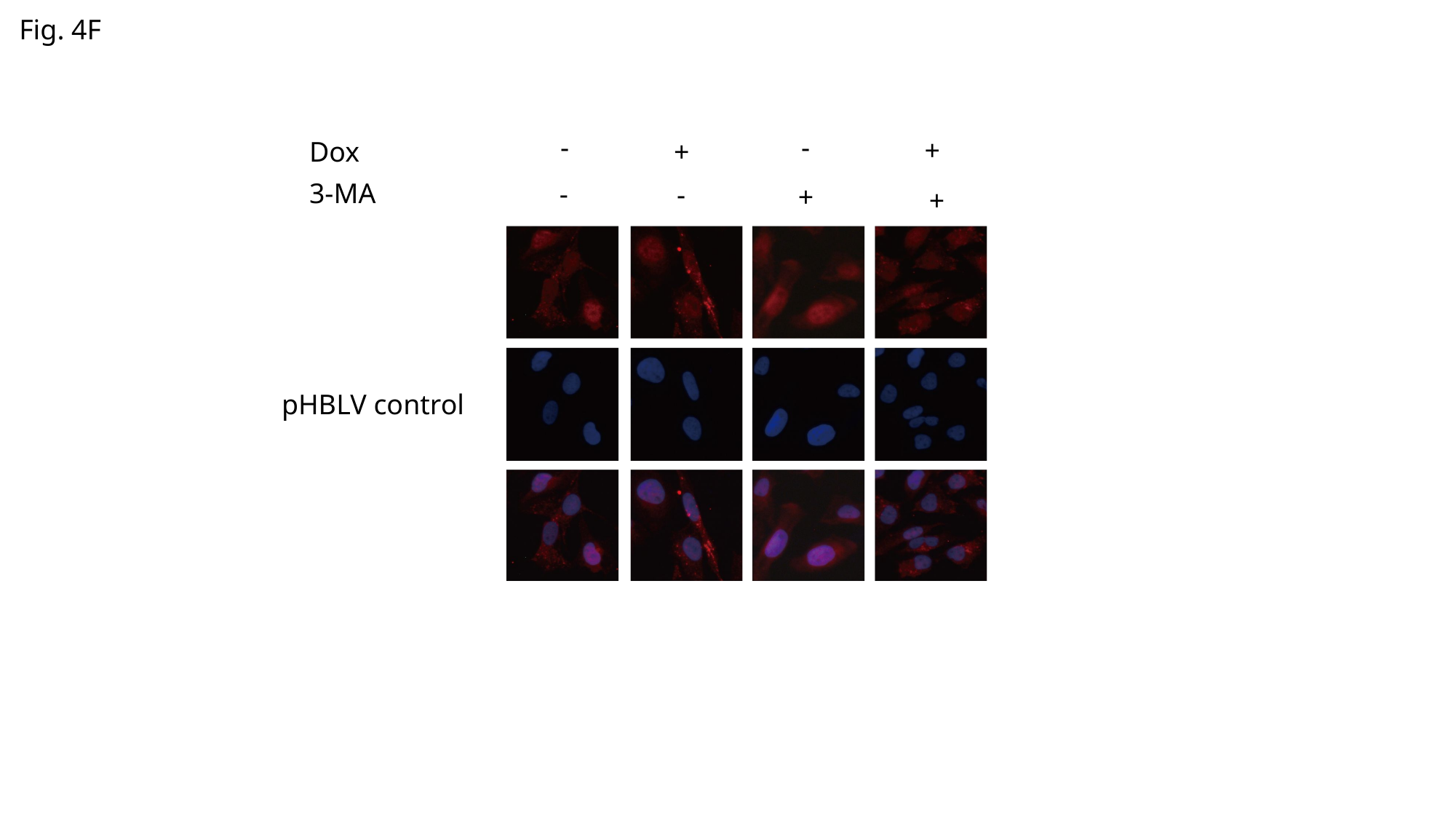

Fig. 4F
-
-
+
+
Dox
3-MA
-
-
+
+
pHBLV control

## Slide 5
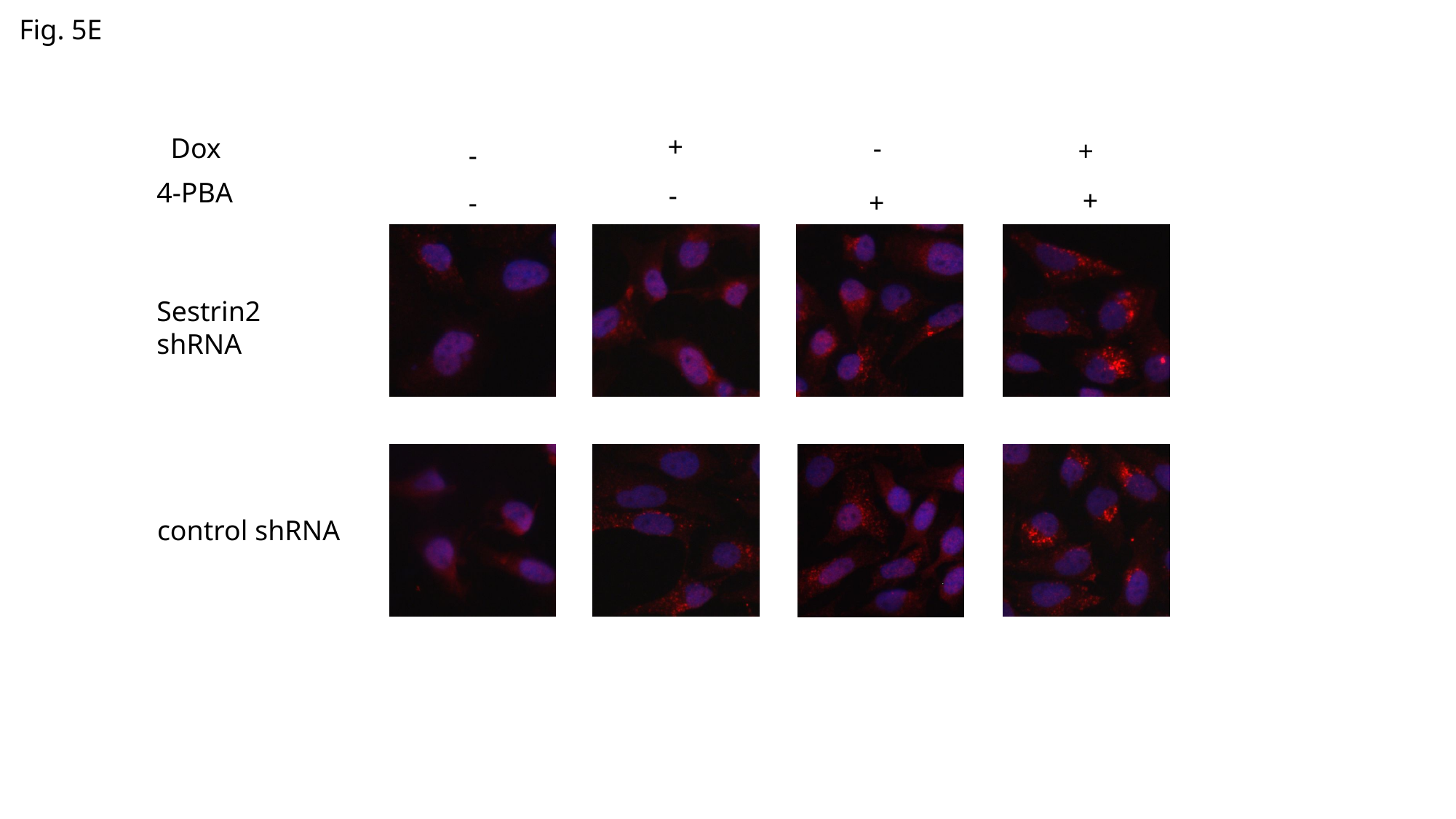

Fig. 5E
+
Dox
-
+
-
4-PBA
-
+
-
+
Sestrin2 shRNA
control shRNA
