# Supplementary material for: Sestrin2-Mediated Autophagy Contributes to Drug Resistance via Endoplasmic Reticulum Stress in Human Osteosarcoma
Source: Front Cell Dev Biol. 2021 Sep 27;9:722960. doi: 10.3389/fcell.2021.722960 (PMC8502982; doi:10.3389/fcell.2021.722960)
Supplement: Supplementary file 2 [file Data_Sheet_3.ZIP › Raw data of quantitative real-time PCR/Raw data of quantitative real-time PCR.pptx]

## Slide 1
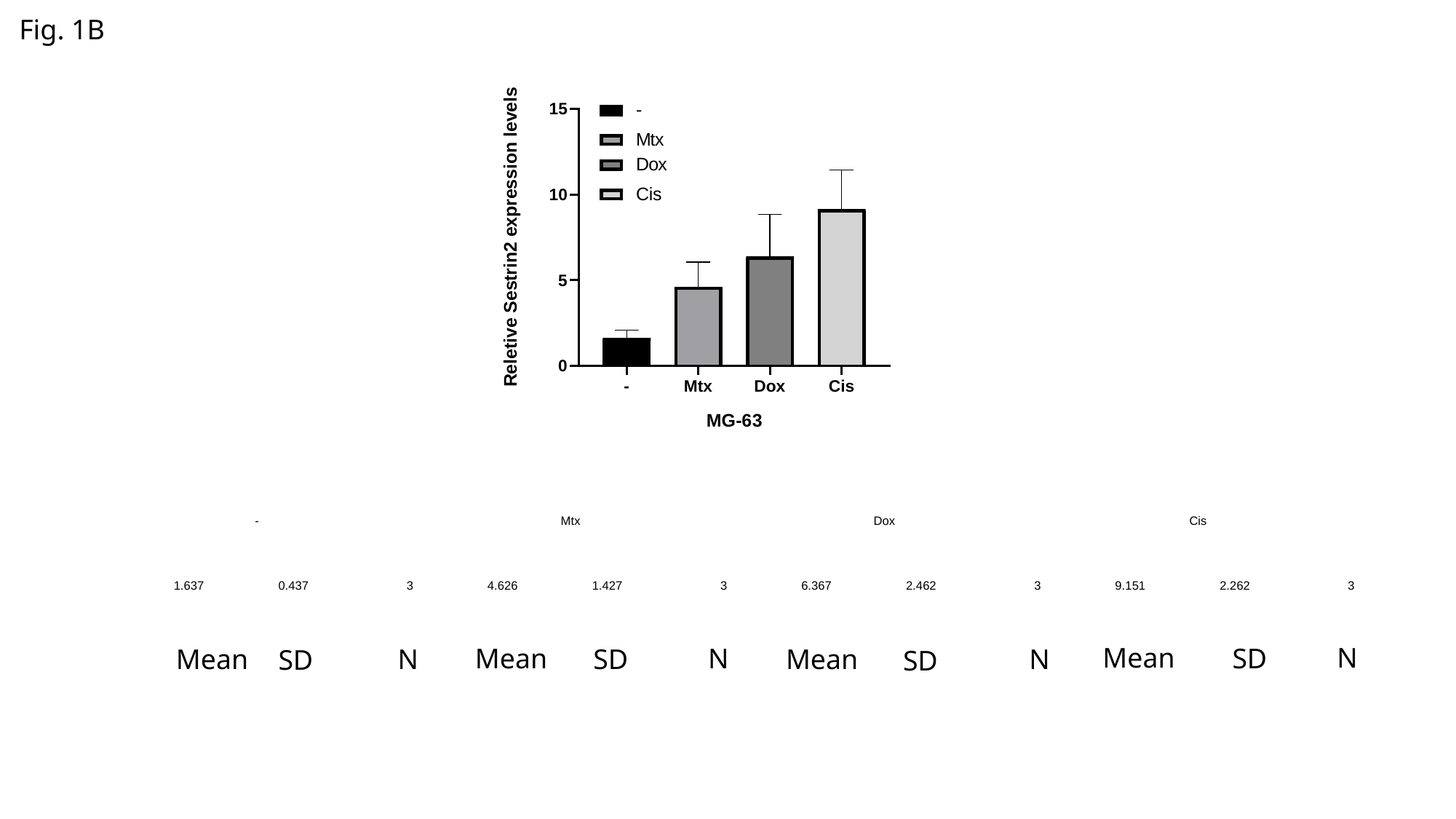

Fig. 1B
| - | | | Mtx | | | Dox | | | Cis | | |
| --- | --- | --- | --- | --- | --- | --- | --- | --- | --- | --- | --- |
| 1.637 | 0.437 | 3 | 4.626 | 1.427 | 3 | 6.367 | 2.462 | 3 | 9.151 | 2.262 | 3 |
Mean
N
Mean
N
SD
Mean
N
SD
Mean
N
SD
SD

## Slide 2
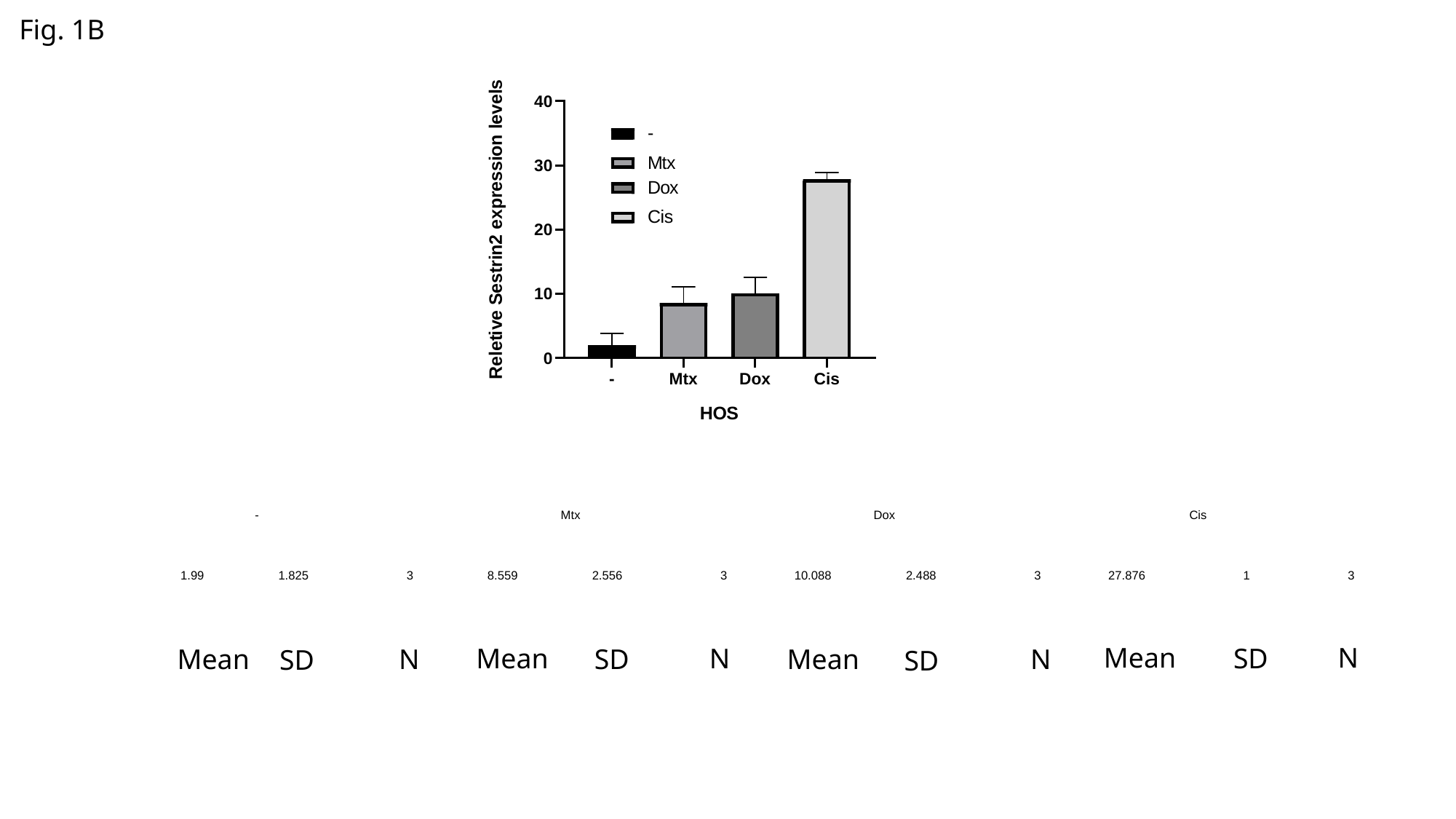

Fig. 1B
| - | | | Mtx | | | Dox | | | Cis | | |
| --- | --- | --- | --- | --- | --- | --- | --- | --- | --- | --- | --- |
| 1.99 | 1.825 | 3 | 8.559 | 2.556 | 3 | 10.088 | 2.488 | 3 | 27.876 | 1 | 3 |
Mean
N
Mean
N
SD
Mean
N
SD
Mean
N
SD
SD

## Slide 3
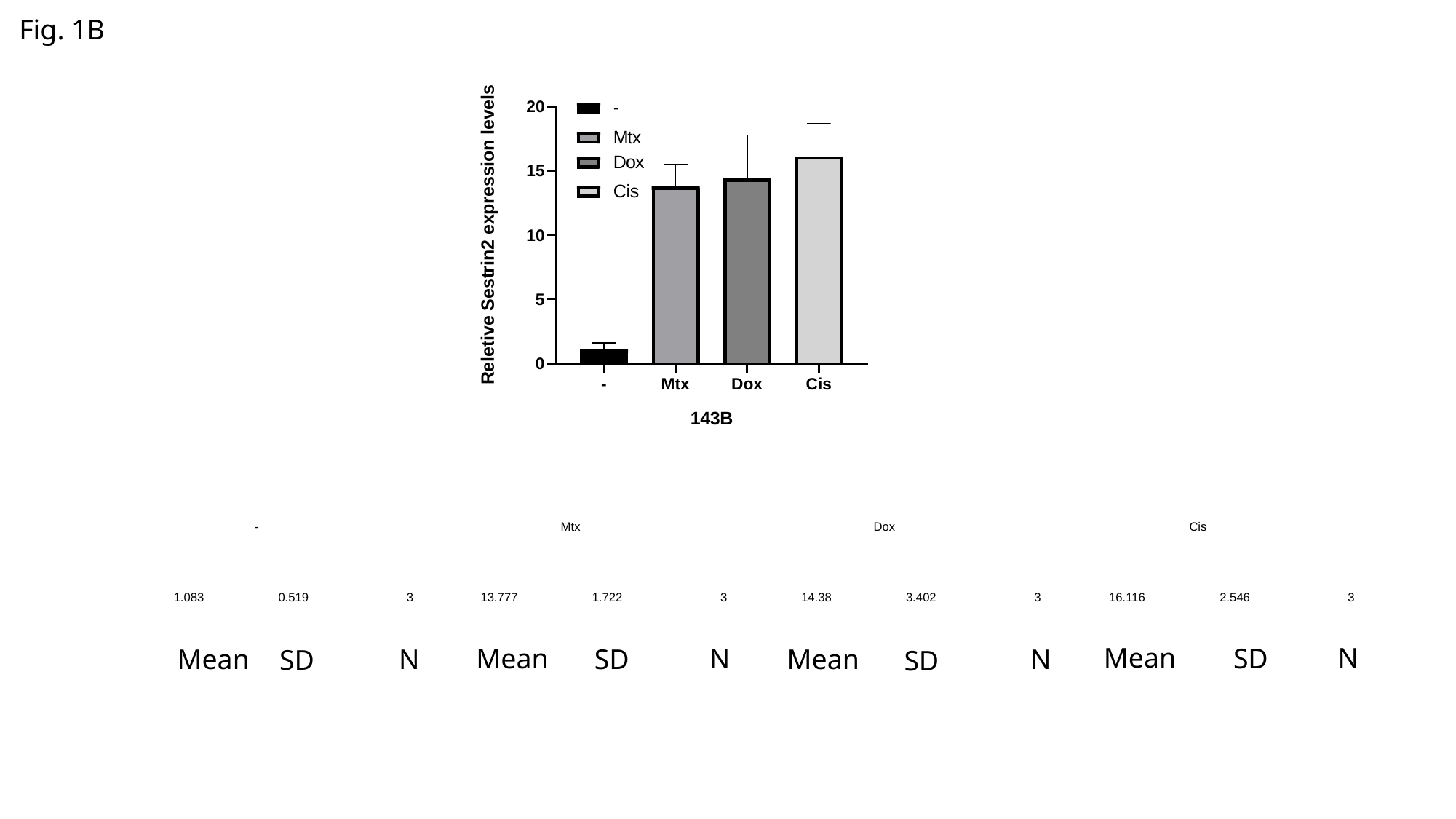

Fig. 1B
| - | | | Mtx | | | Dox | | | Cis | | |
| --- | --- | --- | --- | --- | --- | --- | --- | --- | --- | --- | --- |
| 1.083 | 0.519 | 3 | 13.777 | 1.722 | 3 | 14.38 | 3.402 | 3 | 16.116 | 2.546 | 3 |
Mean
N
Mean
N
SD
Mean
N
SD
Mean
N
SD
SD

## Slide 4
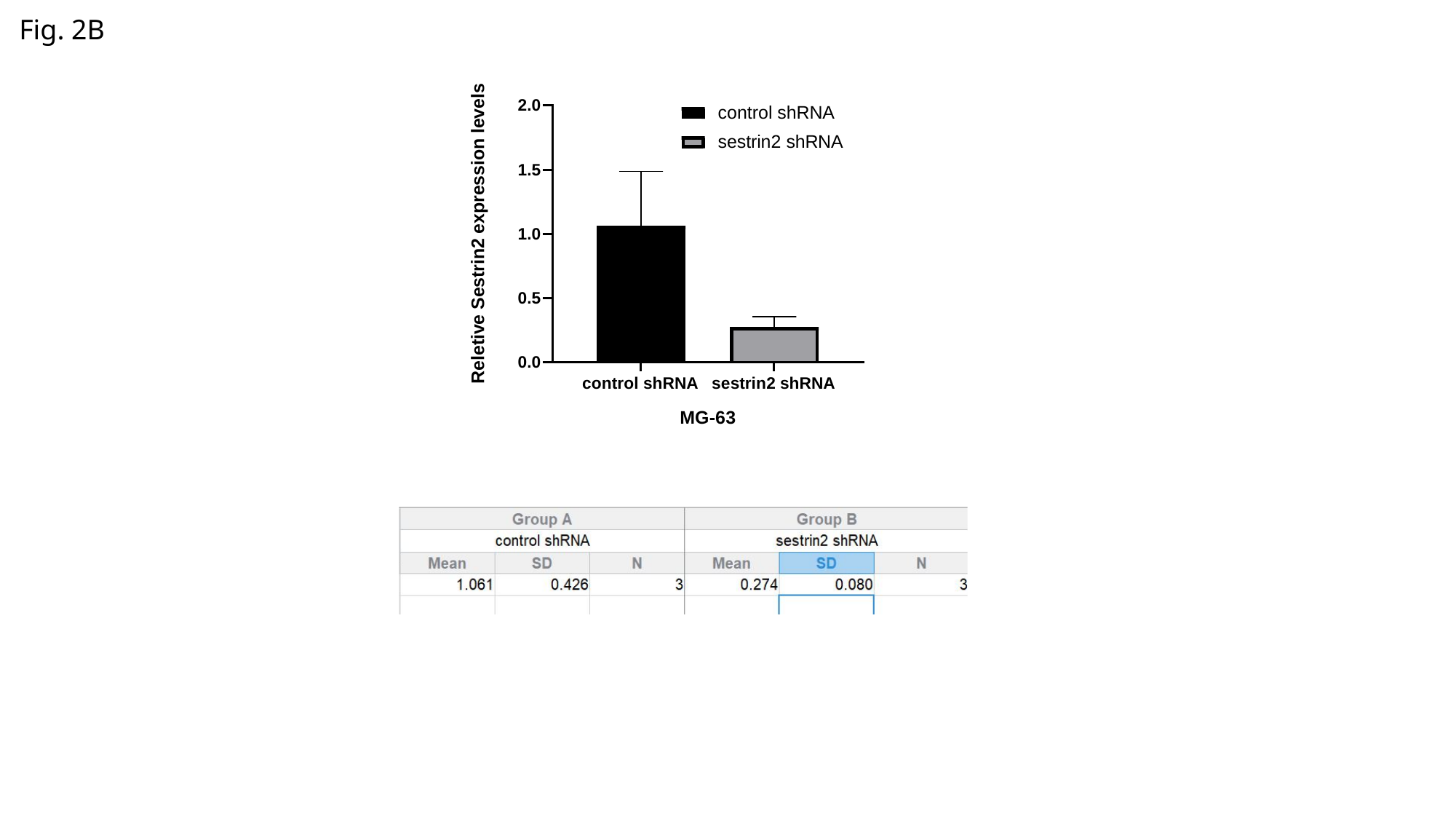

Fig. 2B

## Slide 5
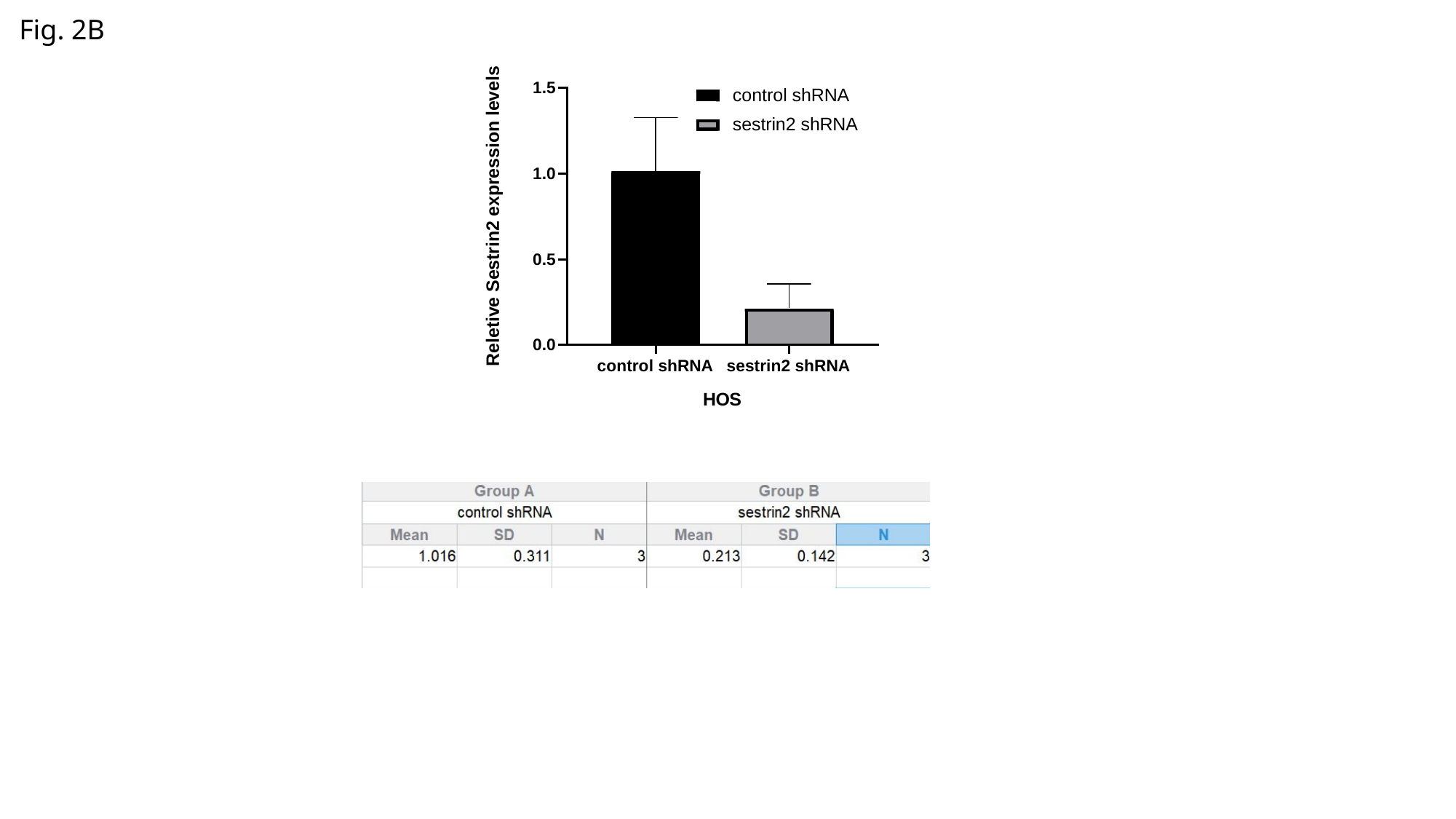

Fig. 2B
