# Supplementary material for: Sestrin2-Mediated Autophagy Contributes to Drug Resistance via Endoplasmic Reticulum Stress in Human Osteosarcoma
Source: Front Cell Dev Biol. 2021 Sep 27;9:722960. doi: 10.3389/fcell.2021.722960 (PMC8502982; doi:10.3389/fcell.2021.722960)
Supplement: Supplementary file 3 [file Data_Sheet_4.ZIP › Raw data of autophagic flux/Raw data of autophagic flux.pptx]

## Slide 1
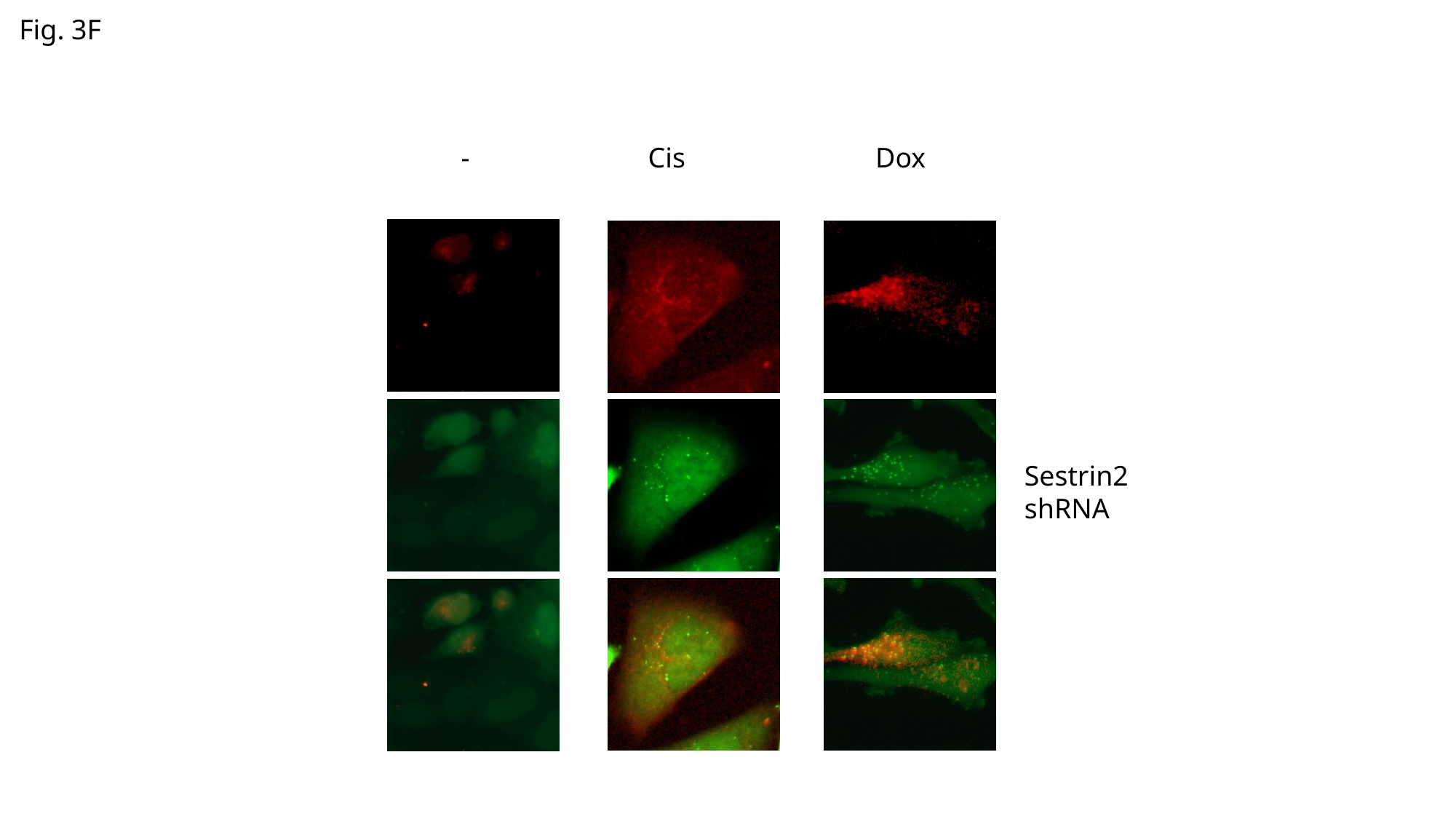

Fig. 3F
-
Cis
Dox
Sestrin2 shRNA

## Slide 2
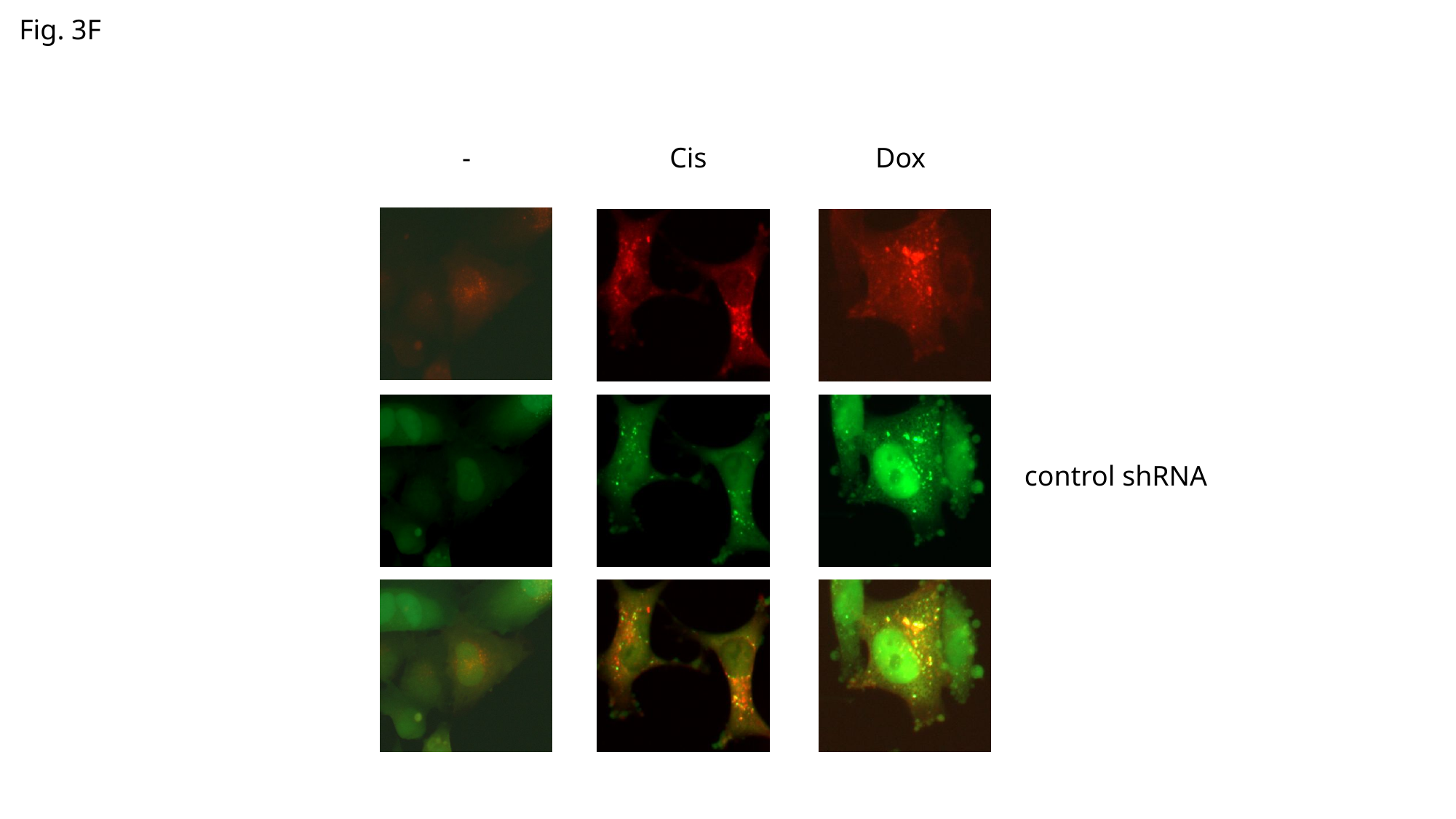

Fig. 3F
-
Cis
Dox
control shRNA

## Slide 3
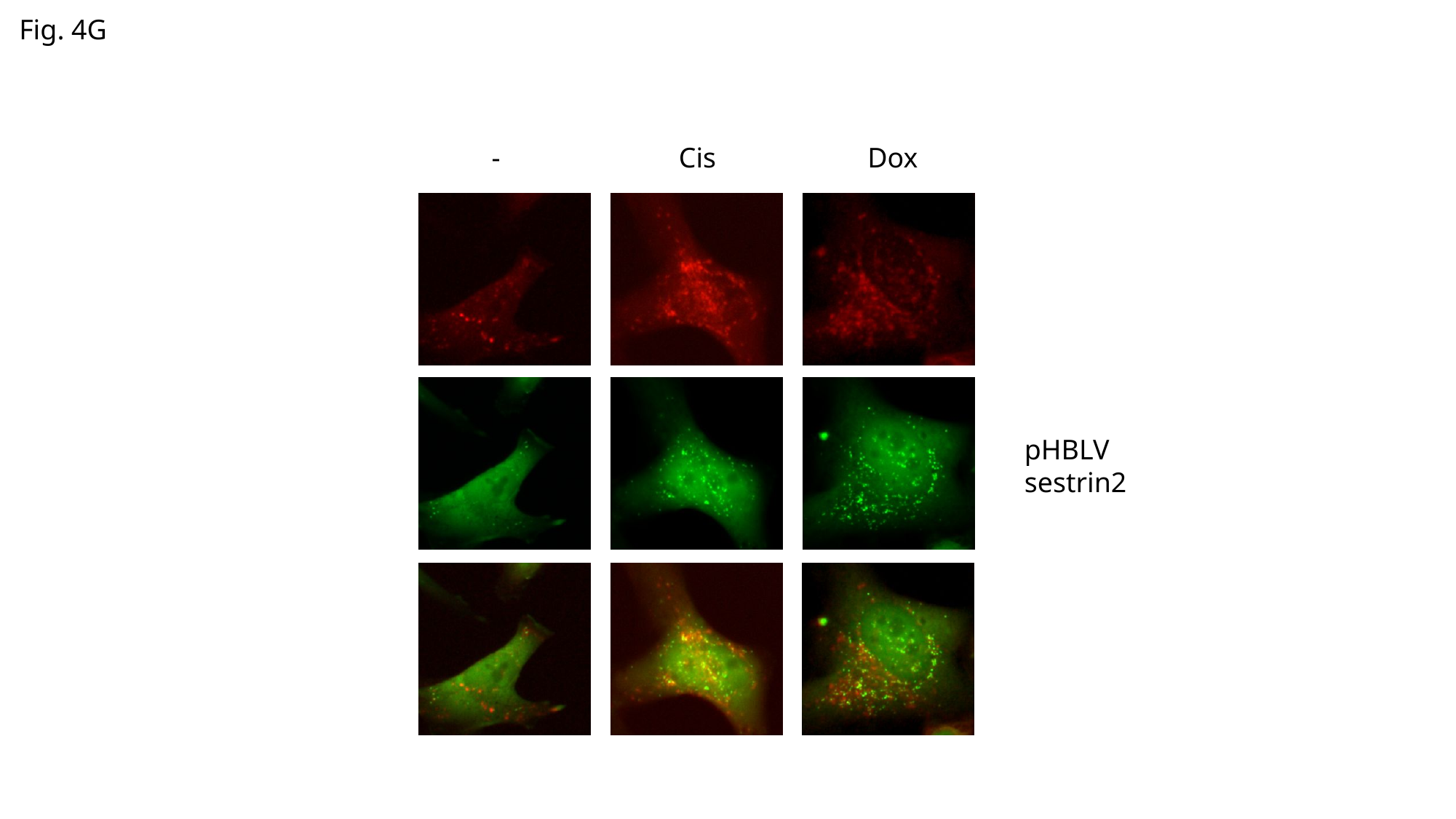

Fig. 4G
-
Cis
Dox
pHBLV sestrin2

## Slide 4
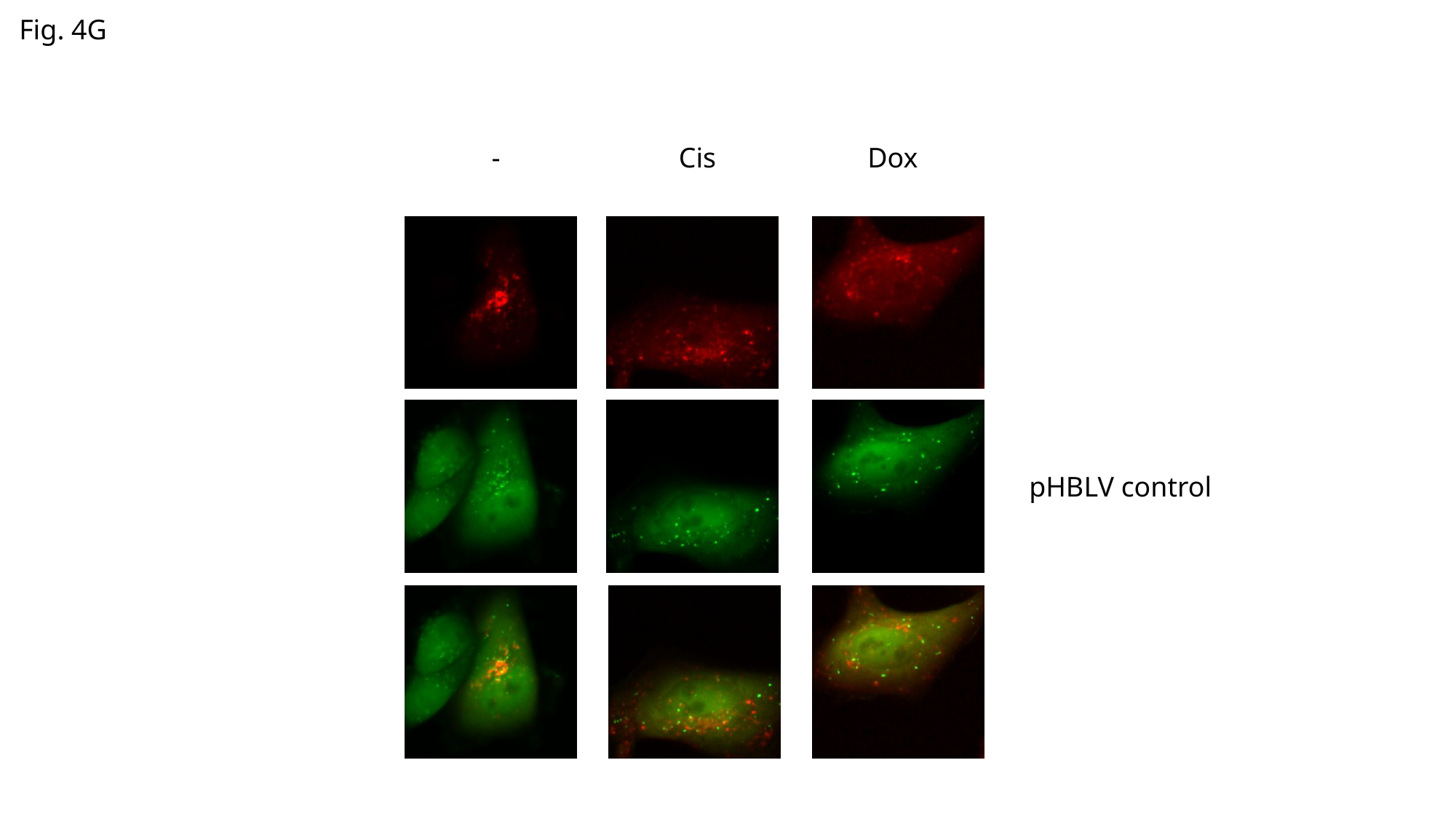

Fig. 4G
-
Cis
Dox
pHBLV control
