# Supplementary figures and images for: Sestrin2-Mediated Autophagy Contributes to Drug Resistance via Endoplasmic Reticulum Stress in Human Osteosarcoma
Source: Front Cell Dev Biol. 2021 Sep 27;9:722960. doi: 10.3389/fcell.2021.722960 (PMC8502982; doi:10.3389/fcell.2021.722960)

## Slide 1
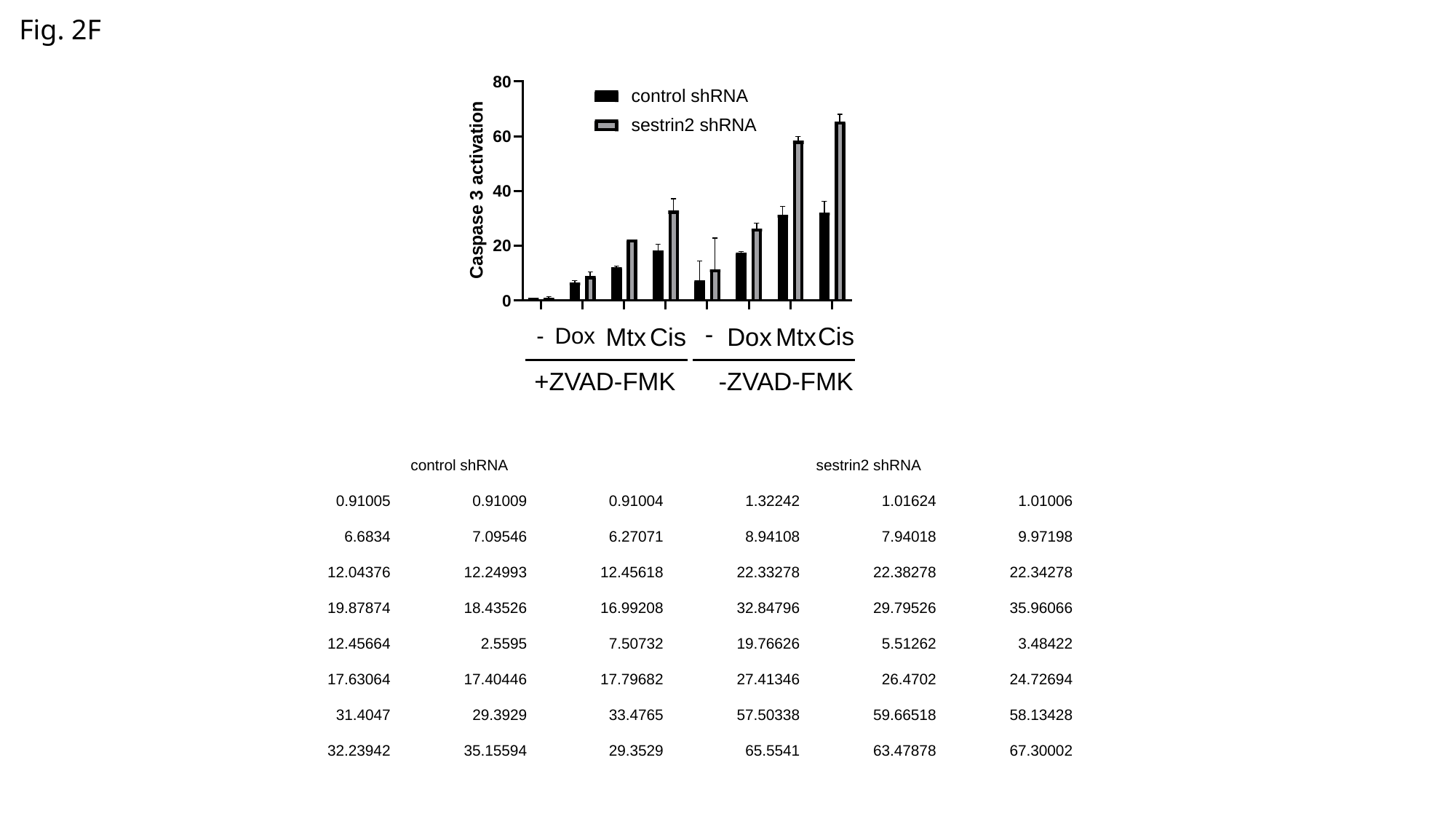

Fig. 2F
-
Cis
Mtx
Cis
Dox
Mtx
-
Dox
+ZVAD-FMK
-ZVAD-FMK
| control shRNA | | | sestrin2 shRNA | | |
| --- | --- | --- | --- | --- | --- |
| 0.91005 | 0.91009 | 0.91004 | 1.32242 | 1.01624 | 1.01006 |
| 6.6834 | 7.09546 | 6.27071 | 8.94108 | 7.94018 | 9.97198 |
| 12.04376 | 12.24993 | 12.45618 | 22.33278 | 22.38278 | 22.34278 |
| 19.87874 | 18.43526 | 16.99208 | 32.84796 | 29.79526 | 35.96066 |
| 12.45664 | 2.5595 | 7.50732 | 19.76626 | 5.51262 | 3.48422 |
| 17.63064 | 17.40446 | 17.79682 | 27.41346 | 26.4702 | 24.72694 |
| 31.4047 | 29.3929 | 33.4765 | 57.50338 | 59.66518 | 58.13428 |
| 32.23942 | 35.15594 | 29.3529 | 65.5541 | 63.47878 | 67.30002 |

Supplement: Supplementary file 4 [file Data_Sheet_5.ZIP › Raw data of caspase-3/Raw data of caspase-3.pptx]

## Slide 1
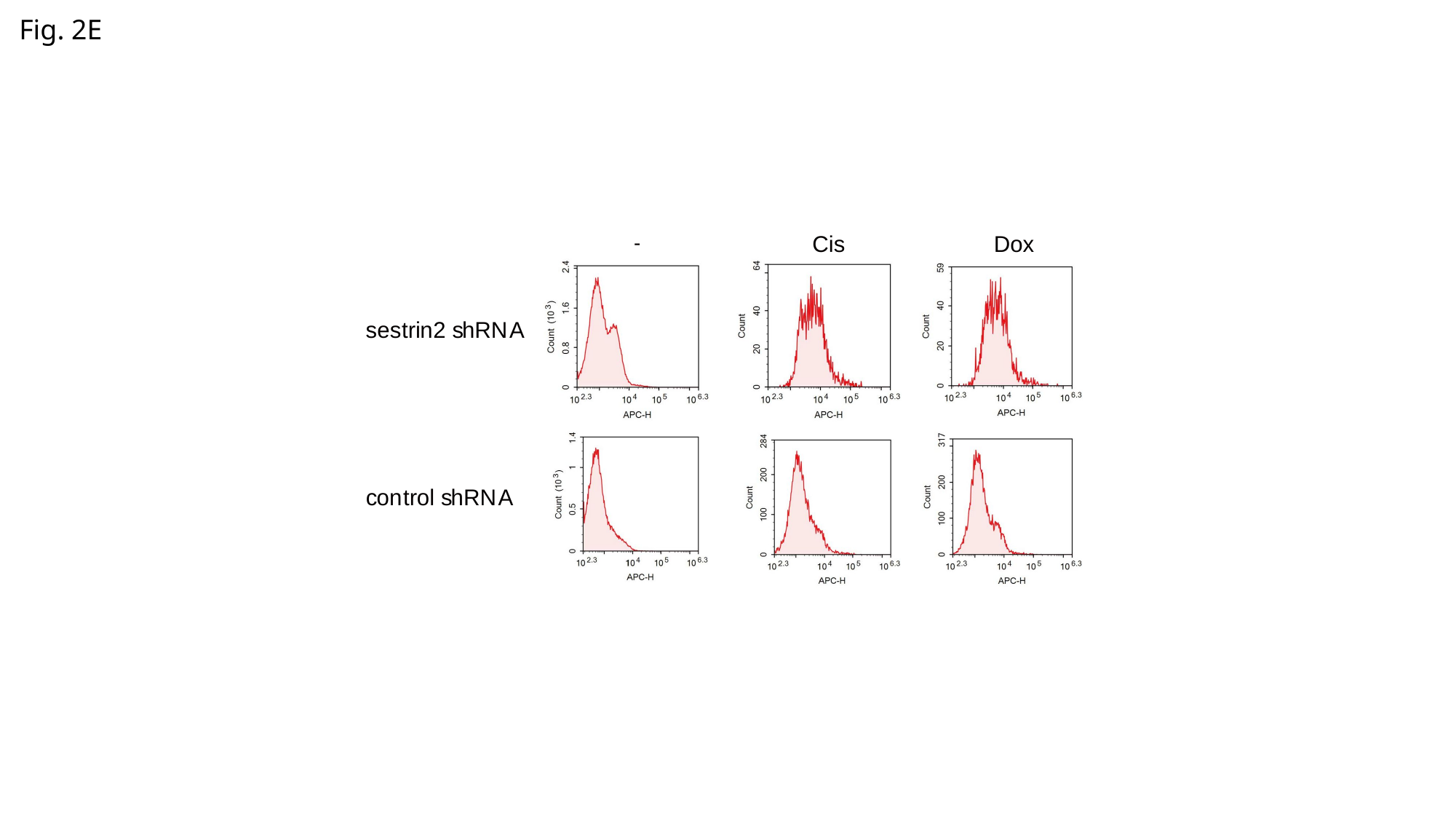

Fig. 2E
Cis
Dox
-
ses
tri
n
2
s
hRN
A
c
on
tr
o
l
s
hRN
A

Supplement: Supplementary file 9 [file Data_Sheet_10.ZIP › Raw data of intracellular Ca2+ measurement/Raw data of intracellular Ca2+ measurement.pptx]

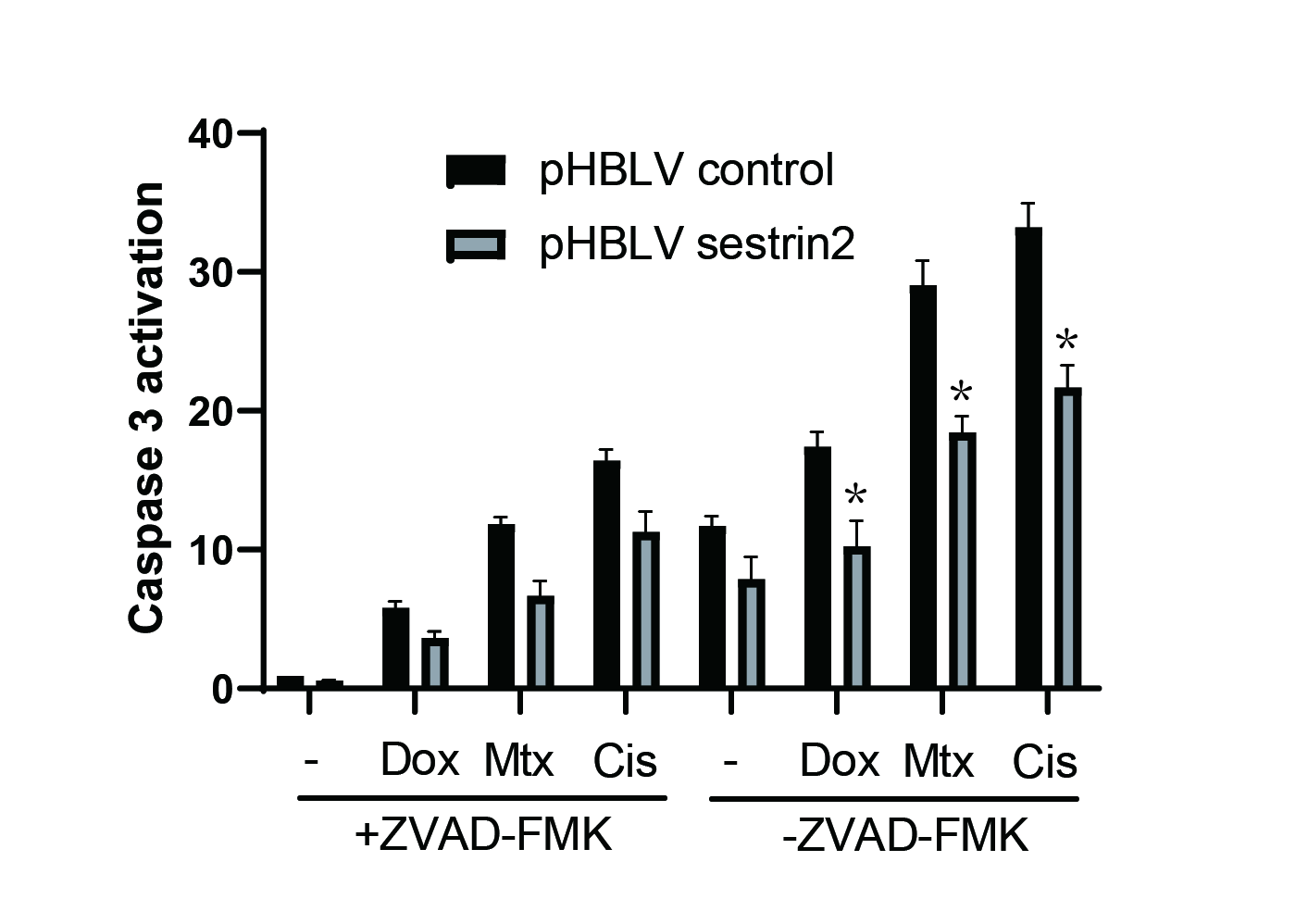

Supplement: Supplementary Figure 1 — After MG-63 cells were treated with Cis (20 μmol/L), Dox (0.2 μg/mL) or Mtx (50 μmol/L) for 24 h, the apoptosis of cells transfected with pHBLV control or pHBLV sestrin2 and cultured in the presence or absence of ZVAD-FMK (20 μmol/L) was detected using the Caspase 3 kit (F) (n = 3). [file Image_1.TIF]

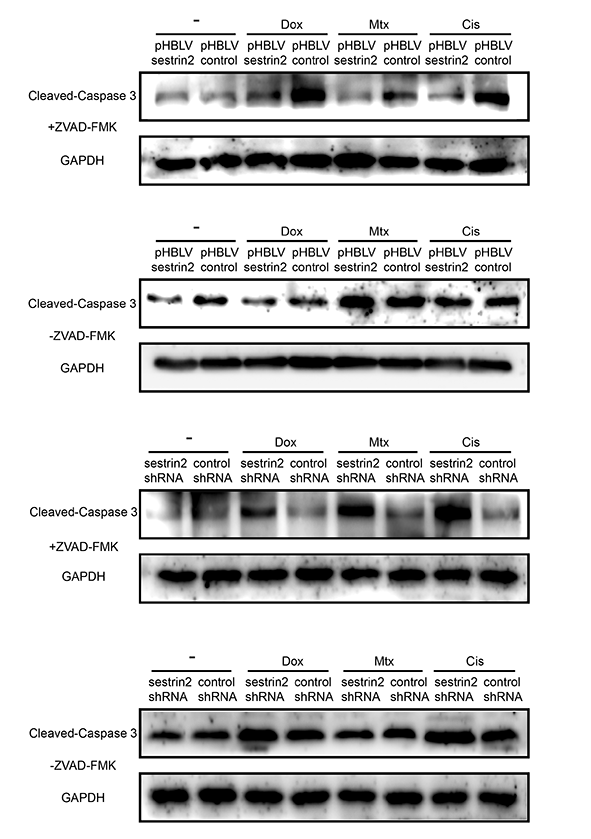

Supplement: Supplementary Figure 2 — After MG-63 cells were treated with Cis (20 μmol/L), Dox (0.2 μg/mL) or Mtx (50 μmol/L) for 24 h, the expression of Cleaved-Caspase 3 transfected with pHBLV control or pHBLV sestrin2 and sestrin2 shRNA or control shRNA, and cultured in the presence or absence of ZVAD-FMK (20 μmol/L) was detected by western blot (F) (n = 3). [file Image_2.TIF]

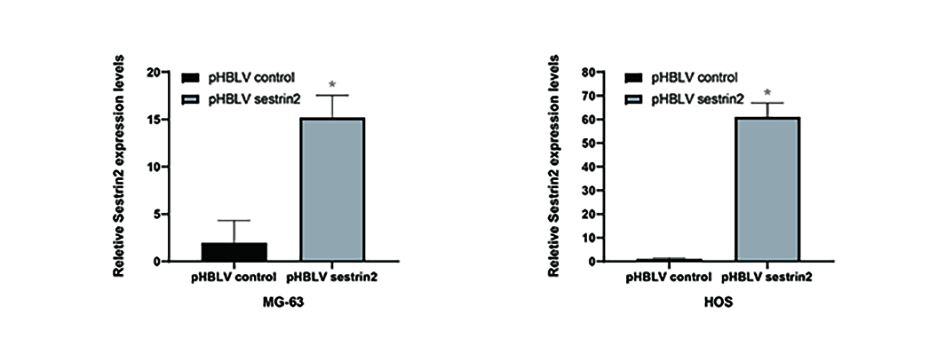

Supplement: Supplementary Figure 3 — MG-63 and HOS cells were transfected with pHBLV control or pHBLV sestrin2, and the transfection effect was detected by quantitative real-time PCR (n = 3). [file Image_3.TIF]

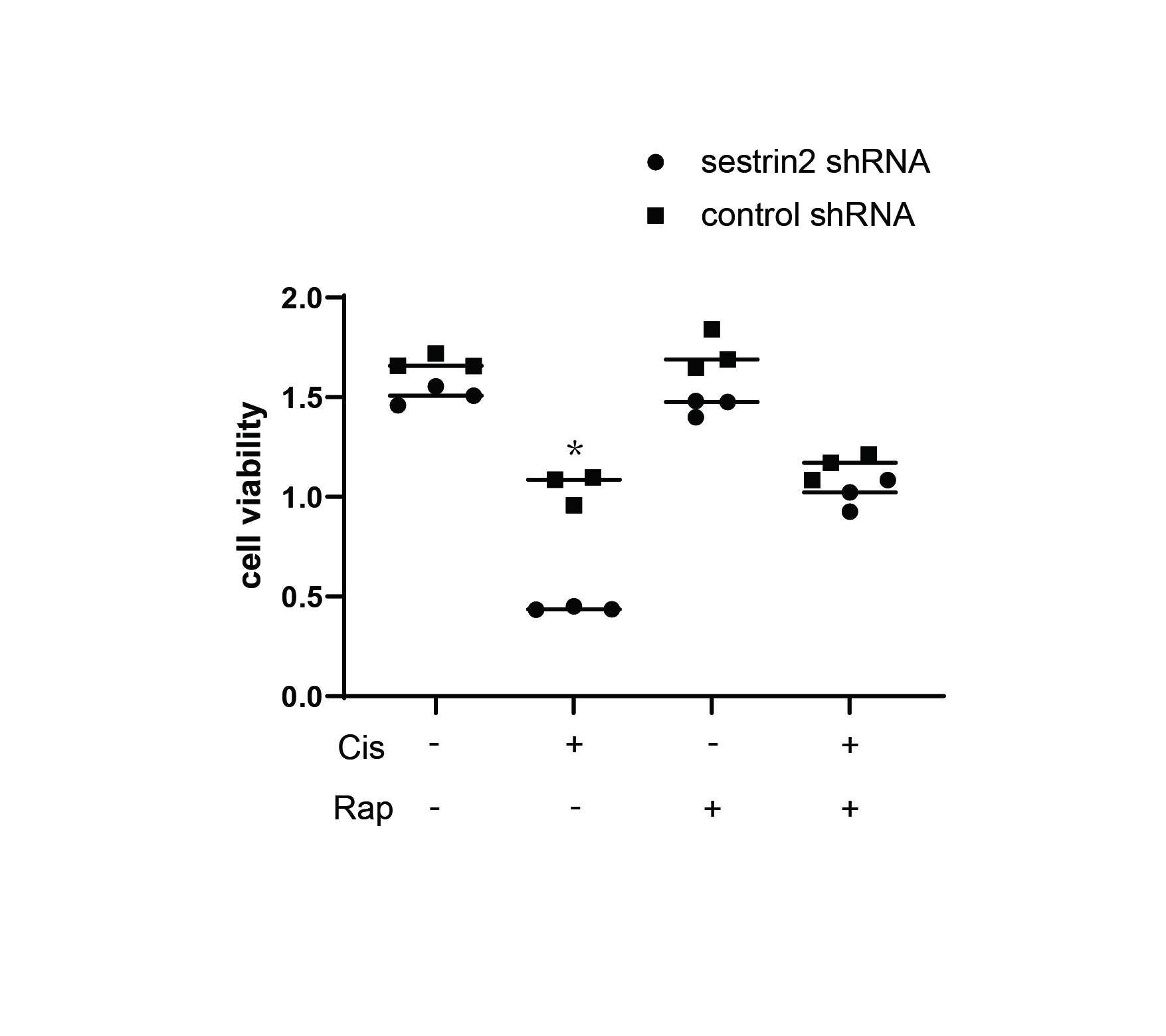

Supplement: Supplementary Figure 4 — SESN2-knockdown HOS cells were treated with Cis (20 μmol/L) for 24 h with or without rapamycin (100 nmol/L) for 6 h. Proliferation was analysed by CCK-8 assay (n = 3). [file Image_4.TIF]

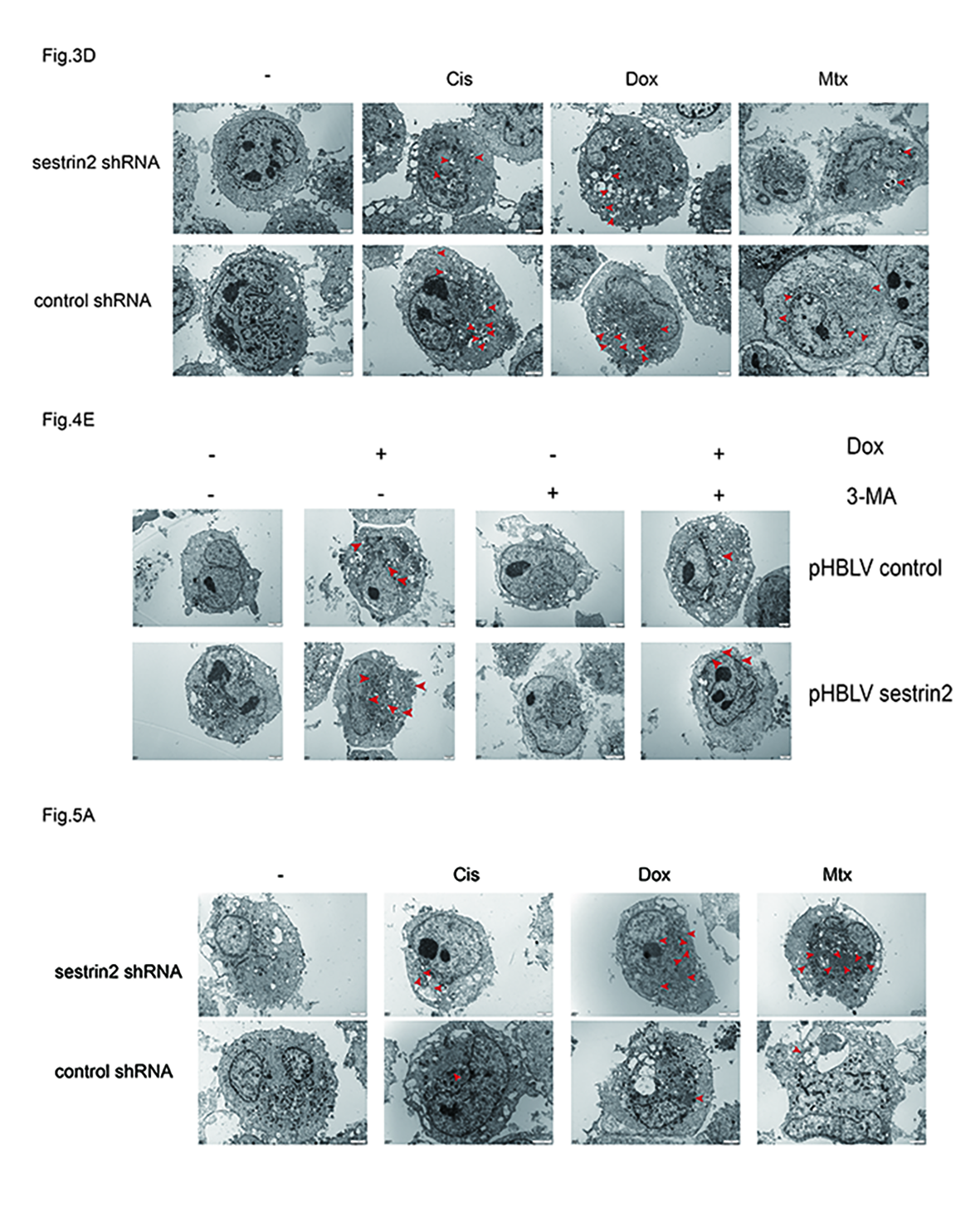

Supplement: Supplementary Figure 5 — The red arrows are autophagosomes in Figures 3D, 4E, 5A. [file Image_5.TIF]
