# Supplementary material for: Sestrin2-Mediated Autophagy Contributes to Drug Resistance via Endoplasmic Reticulum Stress in Human Osteosarcoma
Source: Front Cell Dev Biol. 2021 Sep 27;9:722960. doi: 10.3389/fcell.2021.722960 (PMC8502982; doi:10.3389/fcell.2021.722960)
Supplement: Supplementary file 5 [file Data_Sheet_6.ZIP › Raw data of cell viability assay/Raw data of cell viability assay.pptx]

## Slide 1
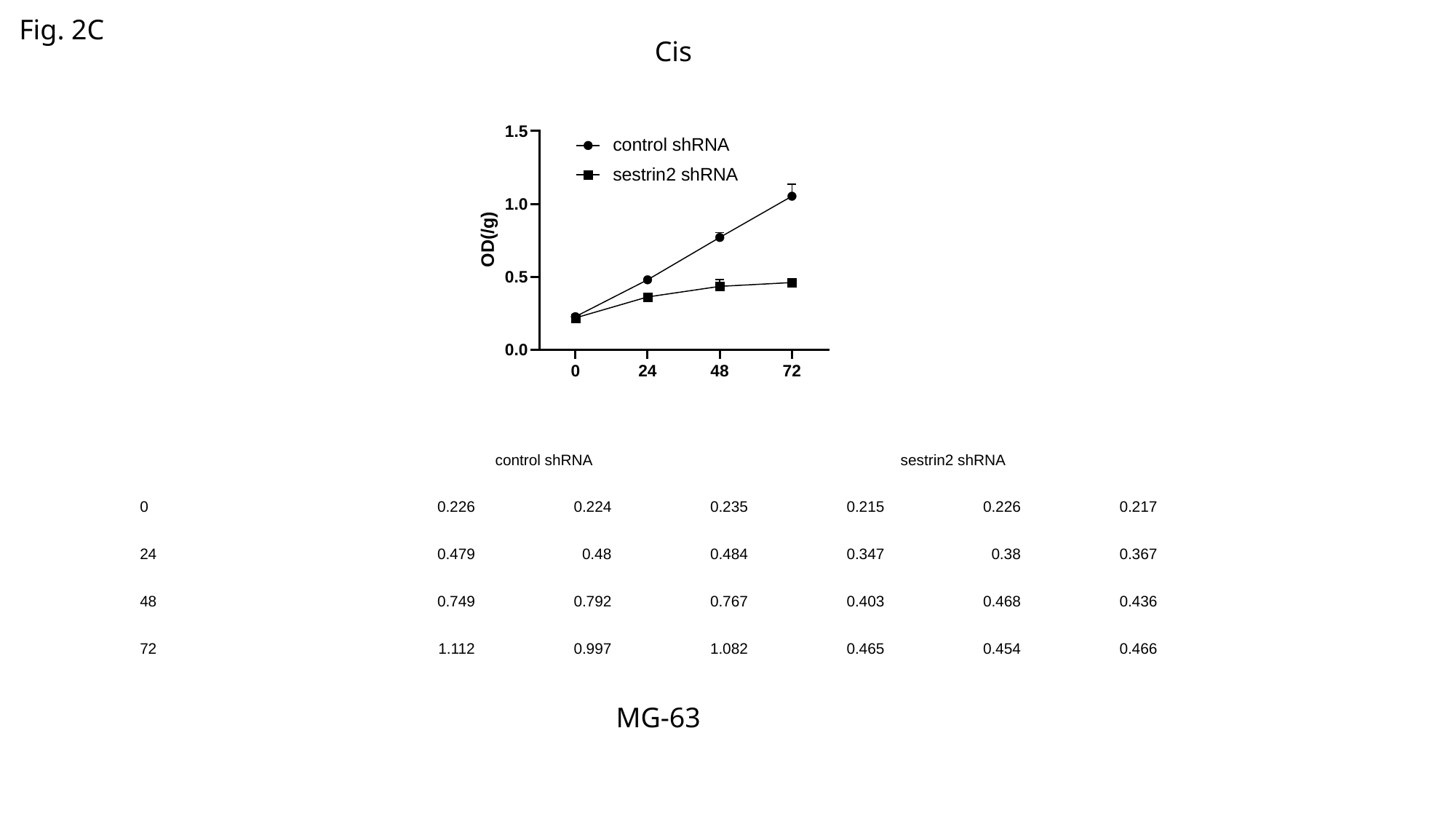

Fig. 2C
Cis
| | | control shRNA | | | sestrin2 shRNA | | |
| --- | --- | --- | --- | --- | --- | --- | --- |
| 0 | | 0.226 | 0.224 | 0.235 | 0.215 | 0.226 | 0.217 |
| 24 | | 0.479 | 0.48 | 0.484 | 0.347 | 0.38 | 0.367 |
| 48 | | 0.749 | 0.792 | 0.767 | 0.403 | 0.468 | 0.436 |
| 72 | | 1.112 | 0.997 | 1.082 | 0.465 | 0.454 | 0.466 |
MG-63

## Slide 2
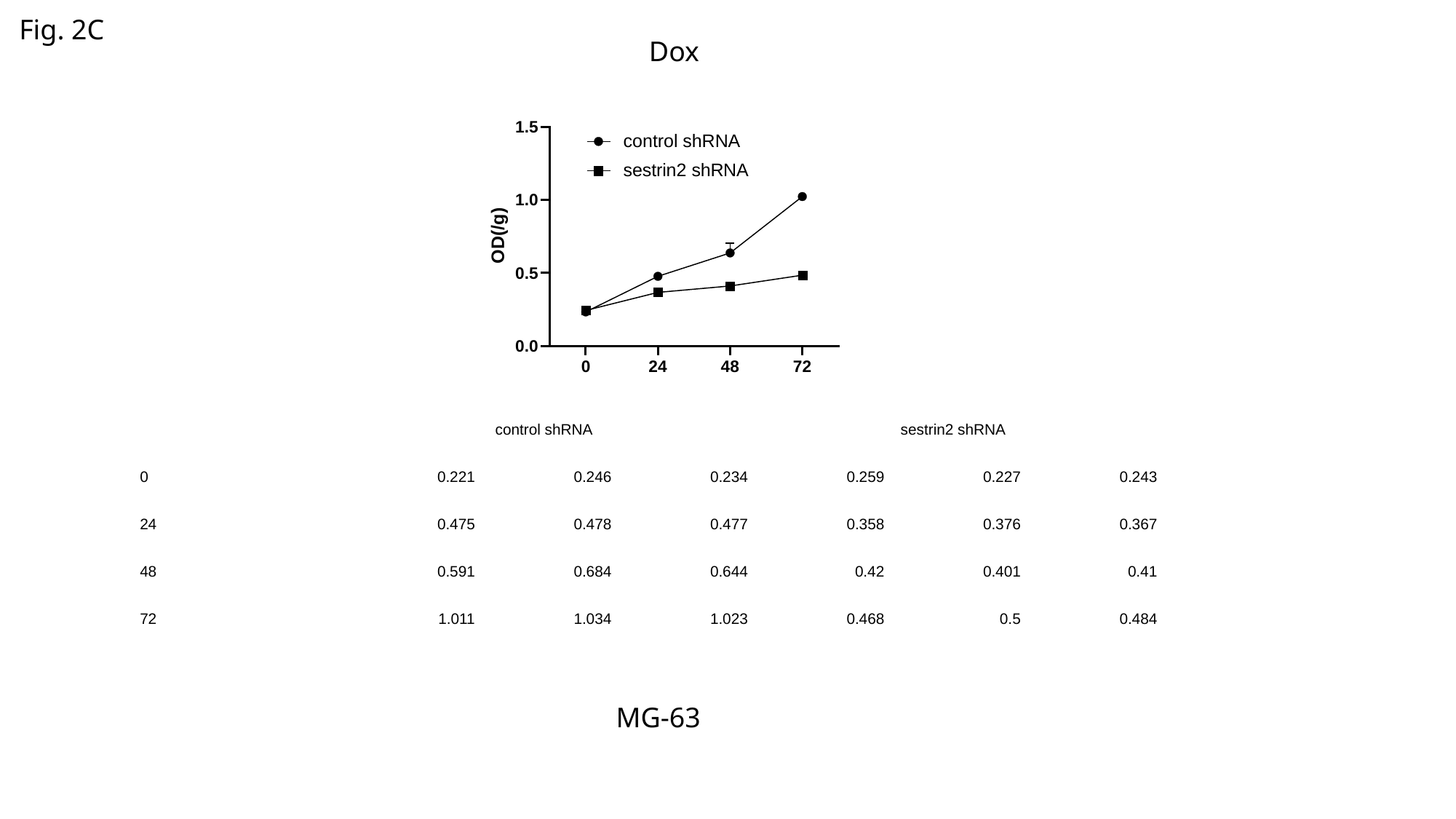

Fig. 2C
Dox
| | | control shRNA | | | sestrin2 shRNA | | |
| --- | --- | --- | --- | --- | --- | --- | --- |
| 0 | | 0.221 | 0.246 | 0.234 | 0.259 | 0.227 | 0.243 |
| 24 | | 0.475 | 0.478 | 0.477 | 0.358 | 0.376 | 0.367 |
| 48 | | 0.591 | 0.684 | 0.644 | 0.42 | 0.401 | 0.41 |
| 72 | | 1.011 | 1.034 | 1.023 | 0.468 | 0.5 | 0.484 |
MG-63

## Slide 3
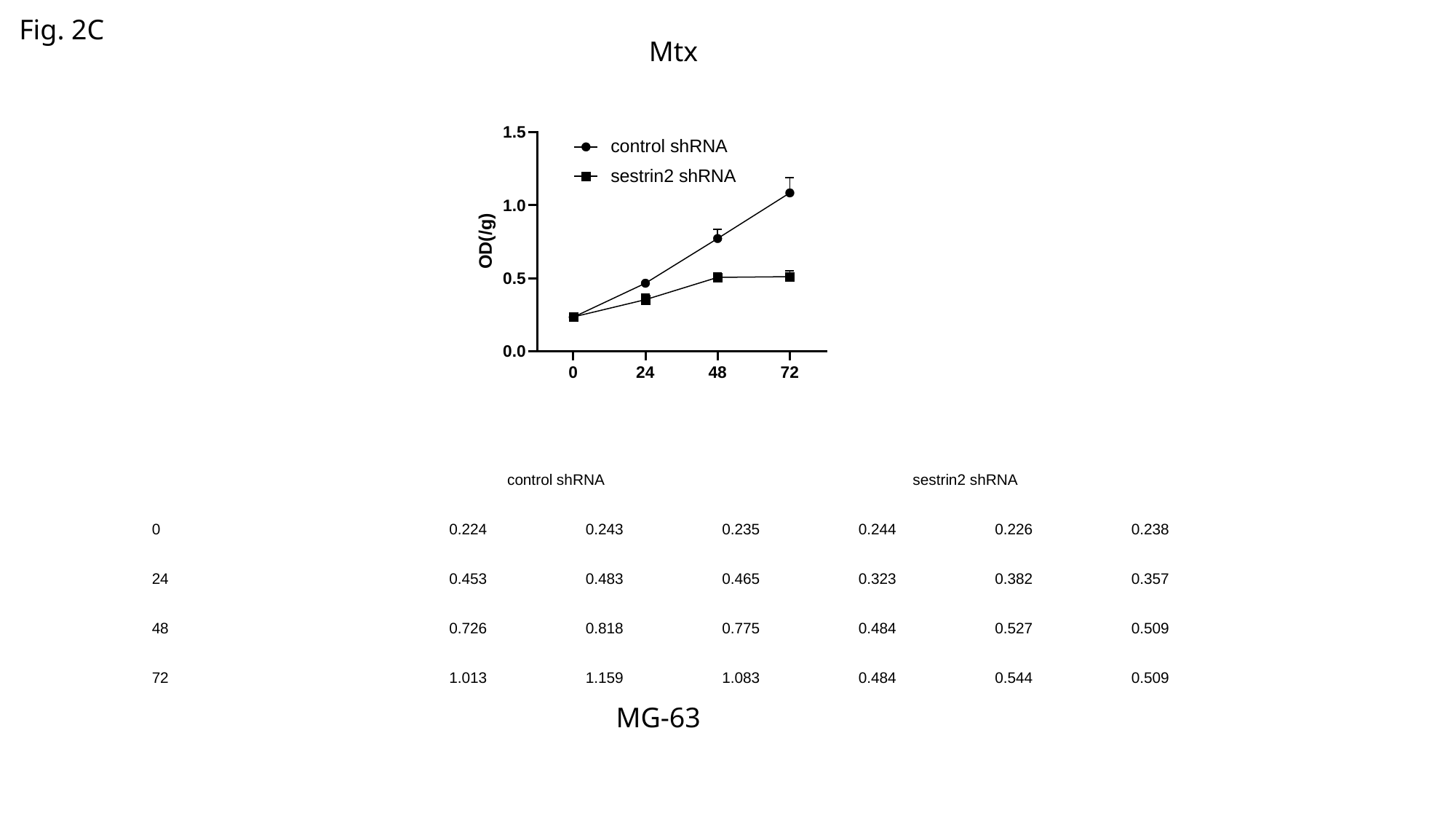

Fig. 2C
Mtx
| | | control shRNA | | | sestrin2 shRNA | | |
| --- | --- | --- | --- | --- | --- | --- | --- |
| 0 | | 0.224 | 0.243 | 0.235 | 0.244 | 0.226 | 0.238 |
| 24 | | 0.453 | 0.483 | 0.465 | 0.323 | 0.382 | 0.357 |
| 48 | | 0.726 | 0.818 | 0.775 | 0.484 | 0.527 | 0.509 |
| 72 | | 1.013 | 1.159 | 1.083 | 0.484 | 0.544 | 0.509 |
MG-63

## Slide 4
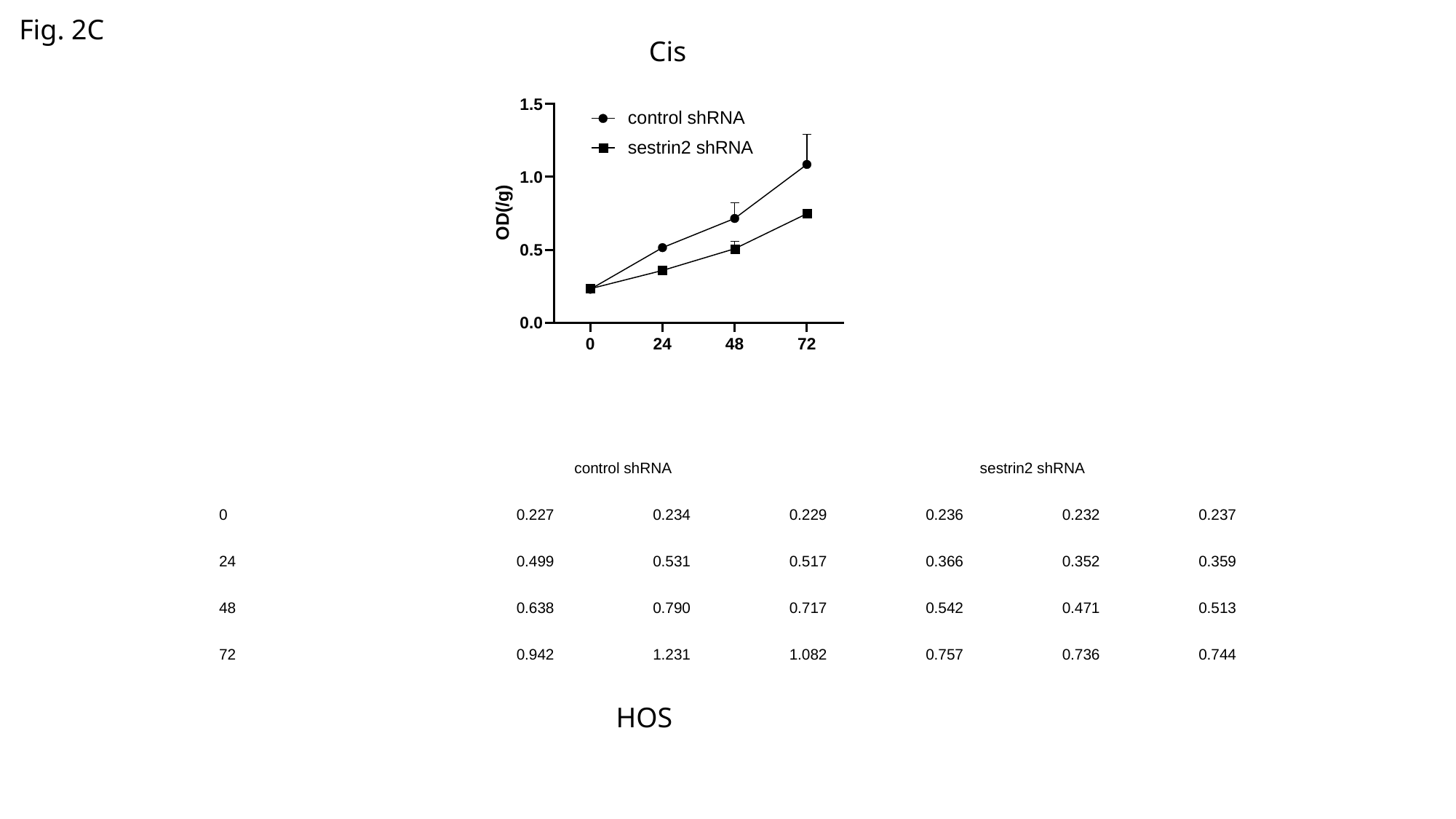

Fig. 2C
Cis
| | | control shRNA | | | sestrin2 shRNA | | |
| --- | --- | --- | --- | --- | --- | --- | --- |
| 0 | | 0.227 | 0.234 | 0.229 | 0.236 | 0.232 | 0.237 |
| 24 | | 0.499 | 0.531 | 0.517 | 0.366 | 0.352 | 0.359 |
| 48 | | 0.638 | 0.790 | 0.717 | 0.542 | 0.471 | 0.513 |
| 72 | | 0.942 | 1.231 | 1.082 | 0.757 | 0.736 | 0.744 |
HOS

## Slide 5
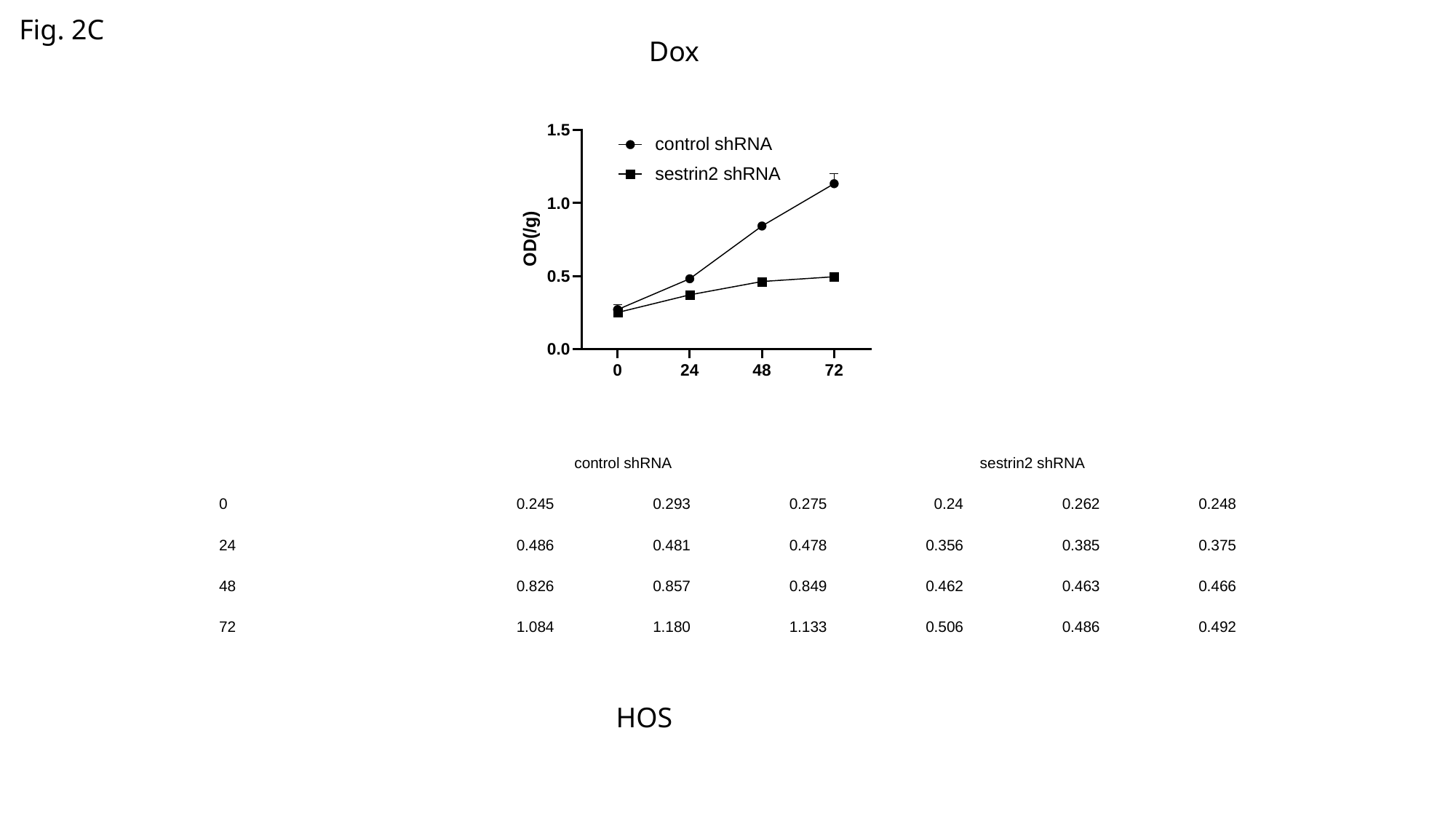

Fig. 2C
Dox
| | | control shRNA | | | sestrin2 shRNA | | |
| --- | --- | --- | --- | --- | --- | --- | --- |
| 0 | | 0.245 | 0.293 | 0.275 | 0.24 | 0.262 | 0.248 |
| 24 | | 0.486 | 0.481 | 0.478 | 0.356 | 0.385 | 0.375 |
| 48 | | 0.826 | 0.857 | 0.849 | 0.462 | 0.463 | 0.466 |
| 72 | | 1.084 | 1.180 | 1.133 | 0.506 | 0.486 | 0.492 |
HOS

## Slide 6
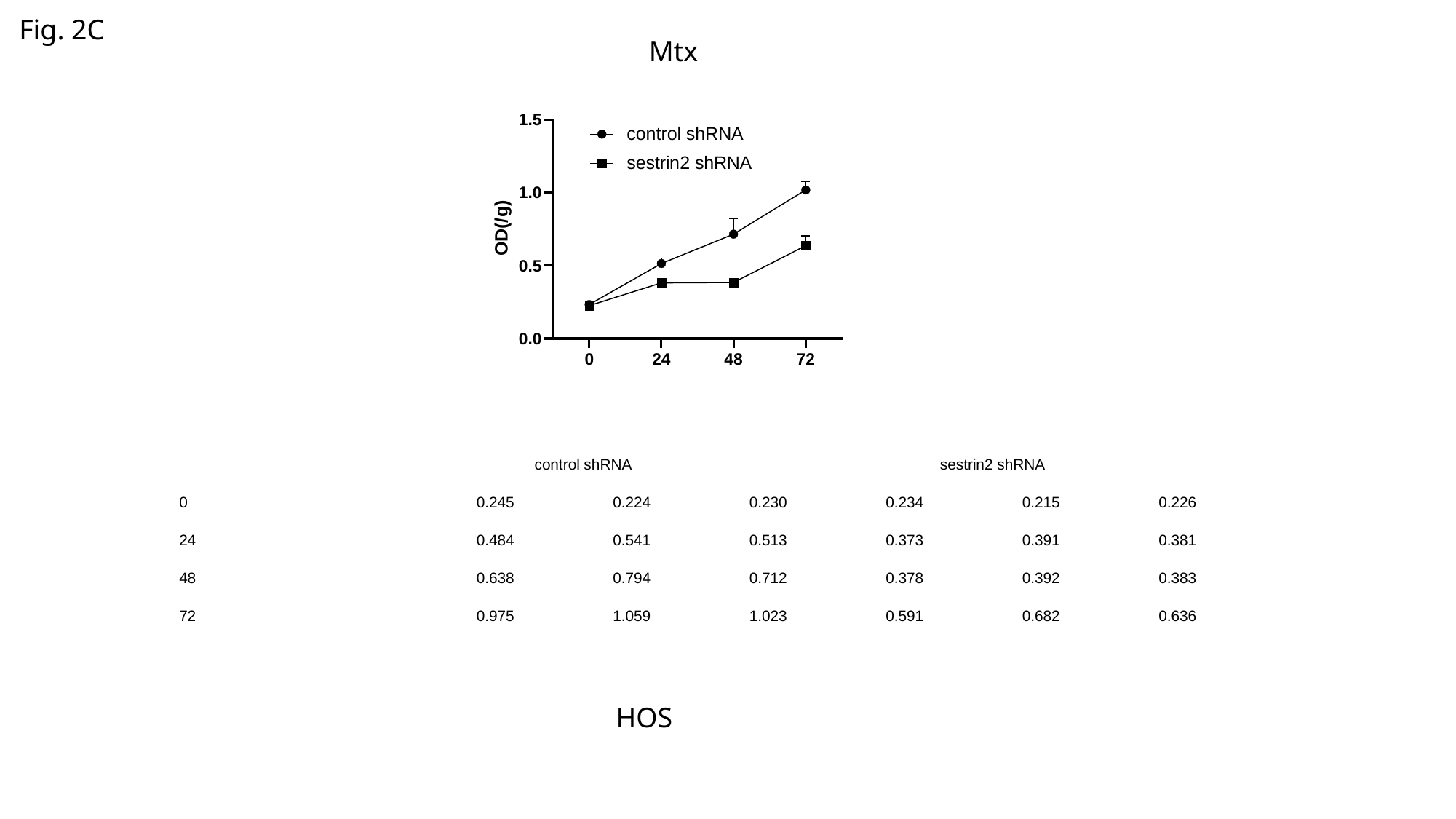

Fig. 2C
Mtx
| | | control shRNA | | | sestrin2 shRNA | | |
| --- | --- | --- | --- | --- | --- | --- | --- |
| 0 | | 0.245 | 0.224 | 0.230 | 0.234 | 0.215 | 0.226 |
| 24 | | 0.484 | 0.541 | 0.513 | 0.373 | 0.391 | 0.381 |
| 48 | | 0.638 | 0.794 | 0.712 | 0.378 | 0.392 | 0.383 |
| 72 | | 0.975 | 1.059 | 1.023 | 0.591 | 0.682 | 0.636 |
HOS

## Slide 7
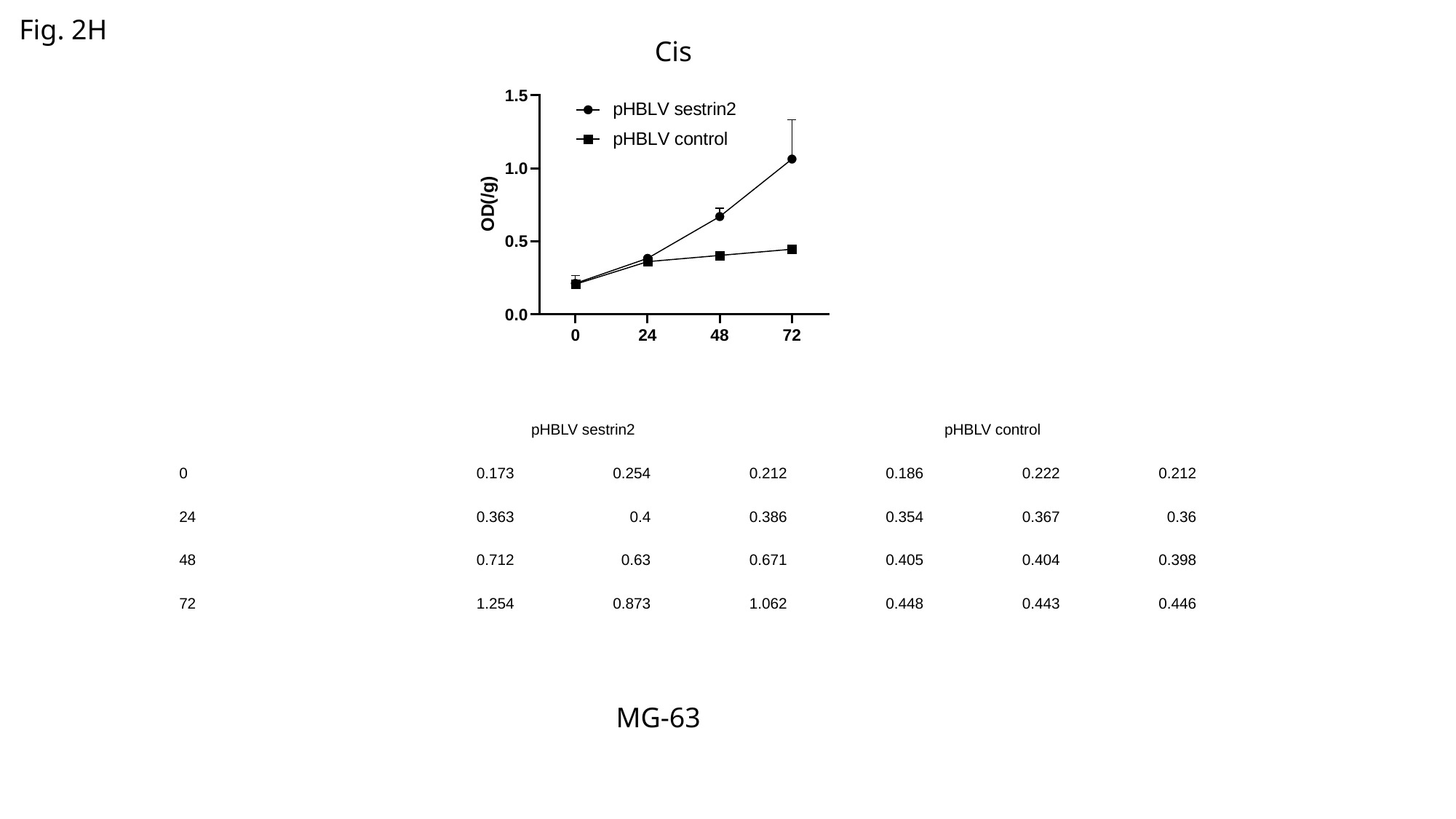

Fig. 2H
Cis
| | | pHBLV sestrin2 | | | pHBLV control | | |
| --- | --- | --- | --- | --- | --- | --- | --- |
| 0 | | 0.173 | 0.254 | 0.212 | 0.186 | 0.222 | 0.212 |
| 24 | | 0.363 | 0.4 | 0.386 | 0.354 | 0.367 | 0.36 |
| 48 | | 0.712 | 0.63 | 0.671 | 0.405 | 0.404 | 0.398 |
| 72 | | 1.254 | 0.873 | 1.062 | 0.448 | 0.443 | 0.446 |
MG-63

## Slide 8
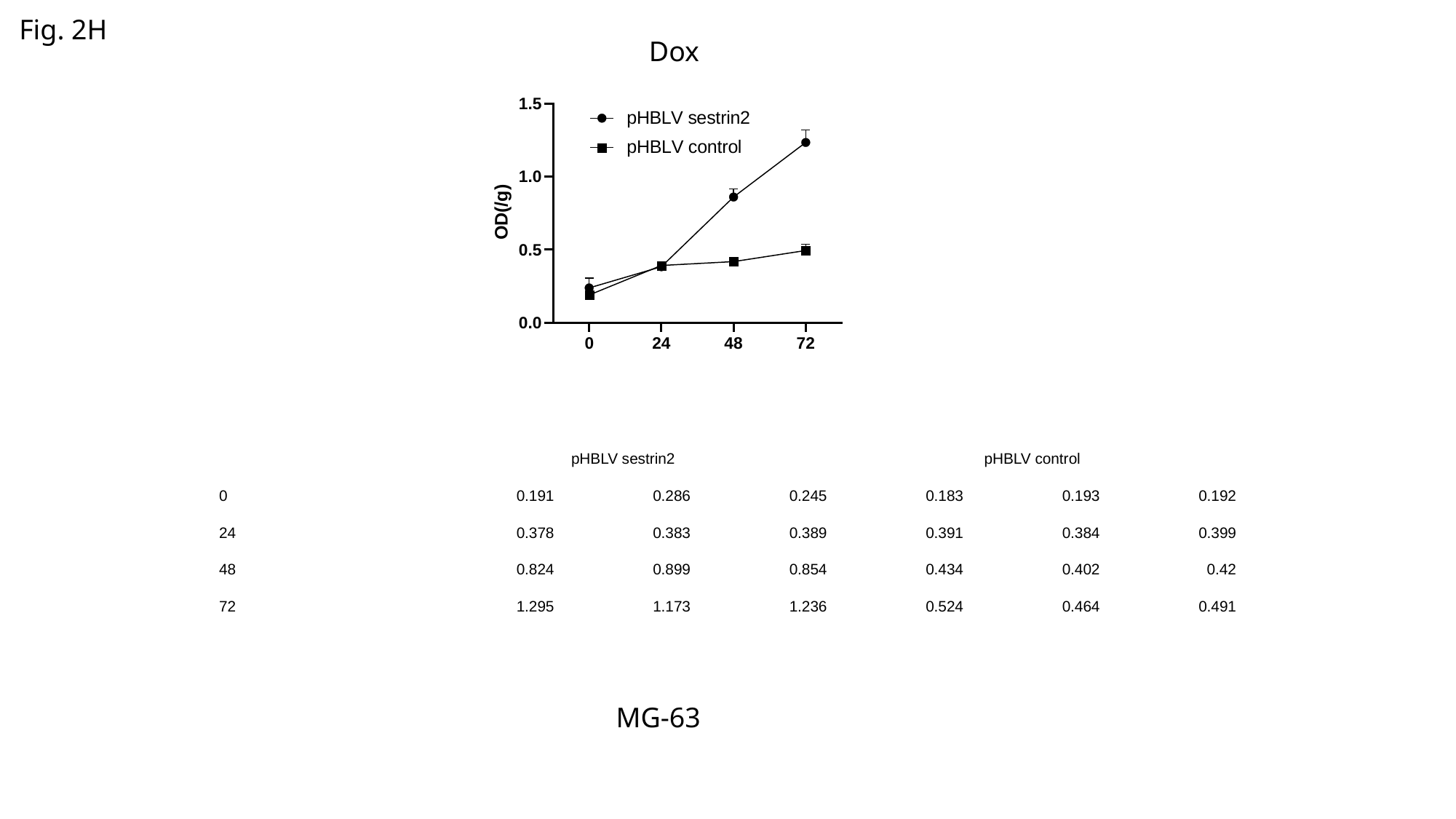

Fig. 2H
Dox
| | | pHBLV sestrin2 | | | pHBLV control | | |
| --- | --- | --- | --- | --- | --- | --- | --- |
| 0 | | 0.191 | 0.286 | 0.245 | 0.183 | 0.193 | 0.192 |
| 24 | | 0.378 | 0.383 | 0.389 | 0.391 | 0.384 | 0.399 |
| 48 | | 0.824 | 0.899 | 0.854 | 0.434 | 0.402 | 0.42 |
| 72 | | 1.295 | 1.173 | 1.236 | 0.524 | 0.464 | 0.491 |
MG-63

## Slide 9
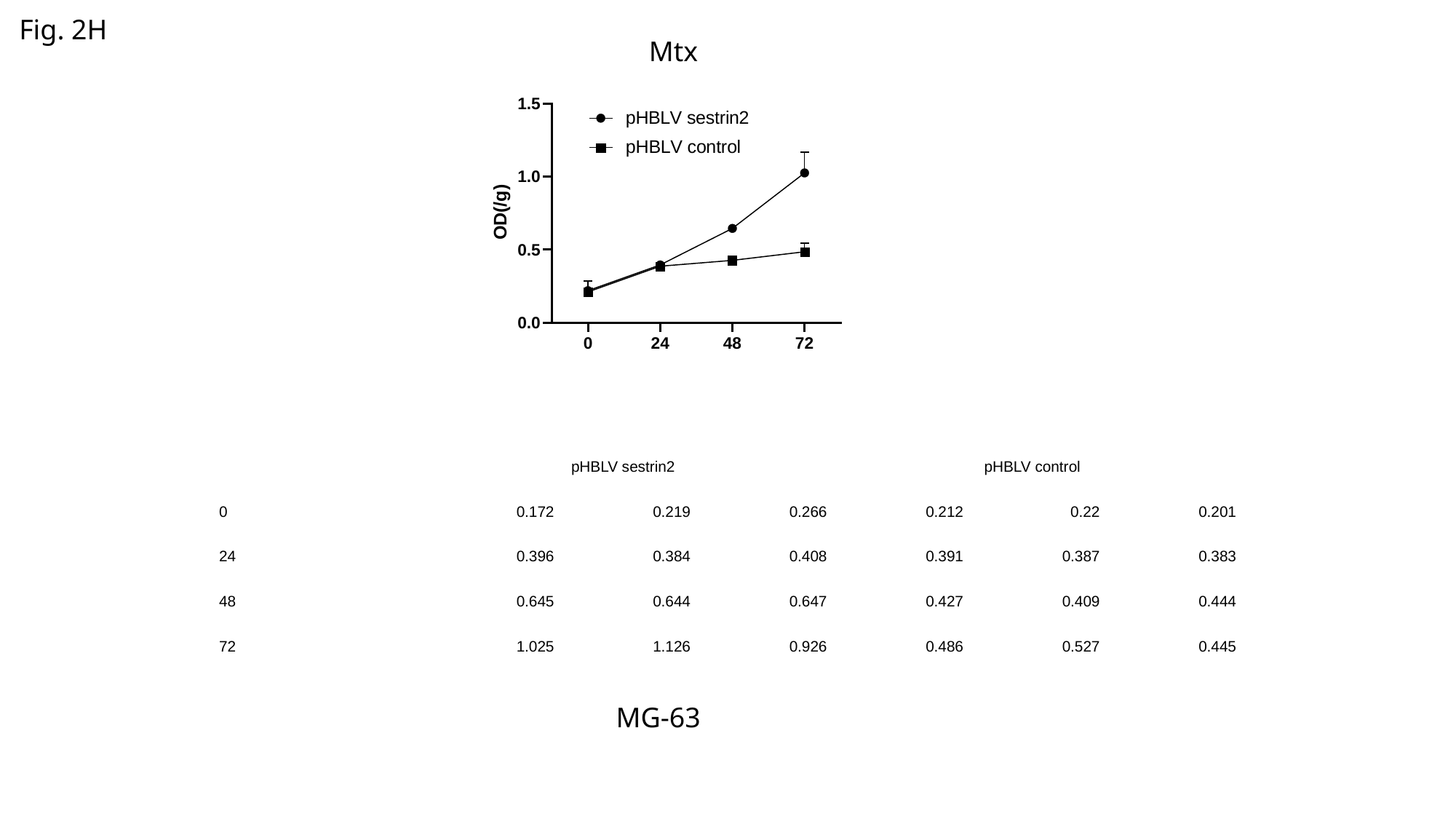

Fig. 2H
Mtx
| | | pHBLV sestrin2 | | | pHBLV control | | |
| --- | --- | --- | --- | --- | --- | --- | --- |
| 0 | | 0.172 | 0.219 | 0.266 | 0.212 | 0.22 | 0.201 |
| 24 | | 0.396 | 0.384 | 0.408 | 0.391 | 0.387 | 0.383 |
| 48 | | 0.645 | 0.644 | 0.647 | 0.427 | 0.409 | 0.444 |
| 72 | | 1.025 | 1.126 | 0.926 | 0.486 | 0.527 | 0.445 |
MG-63

## Slide 10
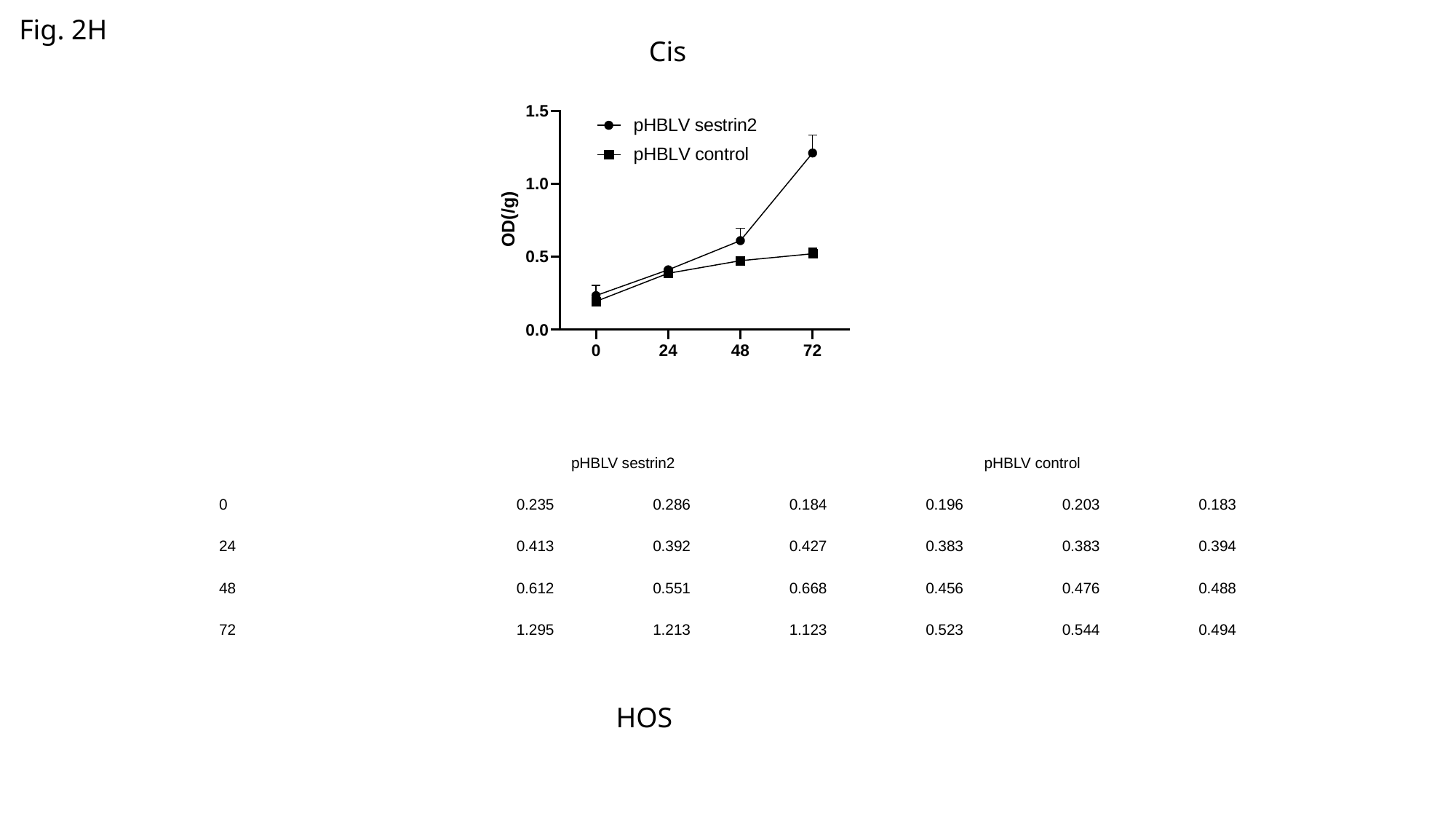

Fig. 2H
Cis
| | | pHBLV sestrin2 | | | pHBLV control | | |
| --- | --- | --- | --- | --- | --- | --- | --- |
| 0 | | 0.235 | 0.286 | 0.184 | 0.196 | 0.203 | 0.183 |
| 24 | | 0.413 | 0.392 | 0.427 | 0.383 | 0.383 | 0.394 |
| 48 | | 0.612 | 0.551 | 0.668 | 0.456 | 0.476 | 0.488 |
| 72 | | 1.295 | 1.213 | 1.123 | 0.523 | 0.544 | 0.494 |
HOS

## Slide 11
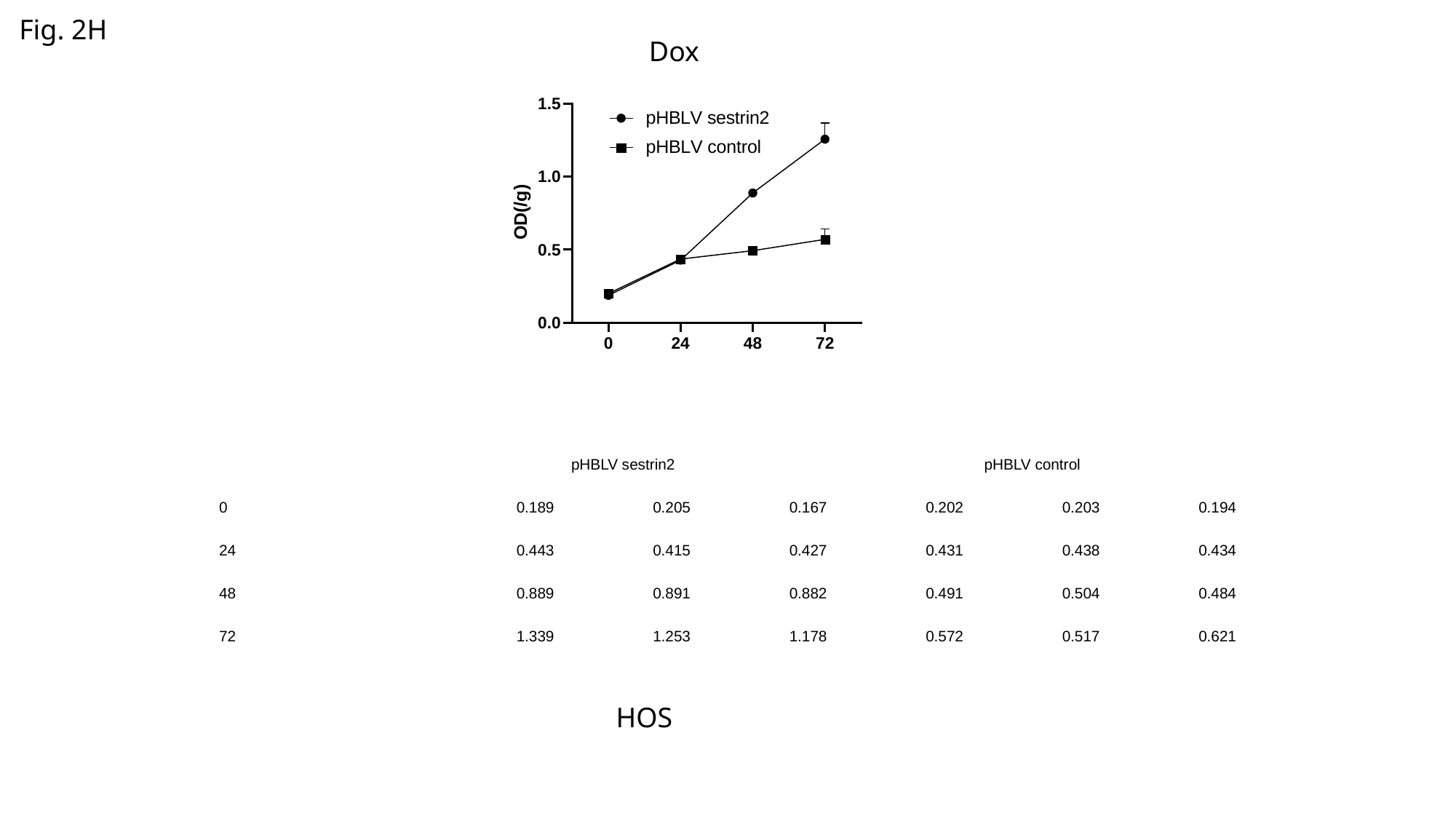

Fig. 2H
Dox
| | | pHBLV sestrin2 | | | pHBLV control | | |
| --- | --- | --- | --- | --- | --- | --- | --- |
| 0 | | 0.189 | 0.205 | 0.167 | 0.202 | 0.203 | 0.194 |
| 24 | | 0.443 | 0.415 | 0.427 | 0.431 | 0.438 | 0.434 |
| 48 | | 0.889 | 0.891 | 0.882 | 0.491 | 0.504 | 0.484 |
| 72 | | 1.339 | 1.253 | 1.178 | 0.572 | 0.517 | 0.621 |
HOS

## Slide 12
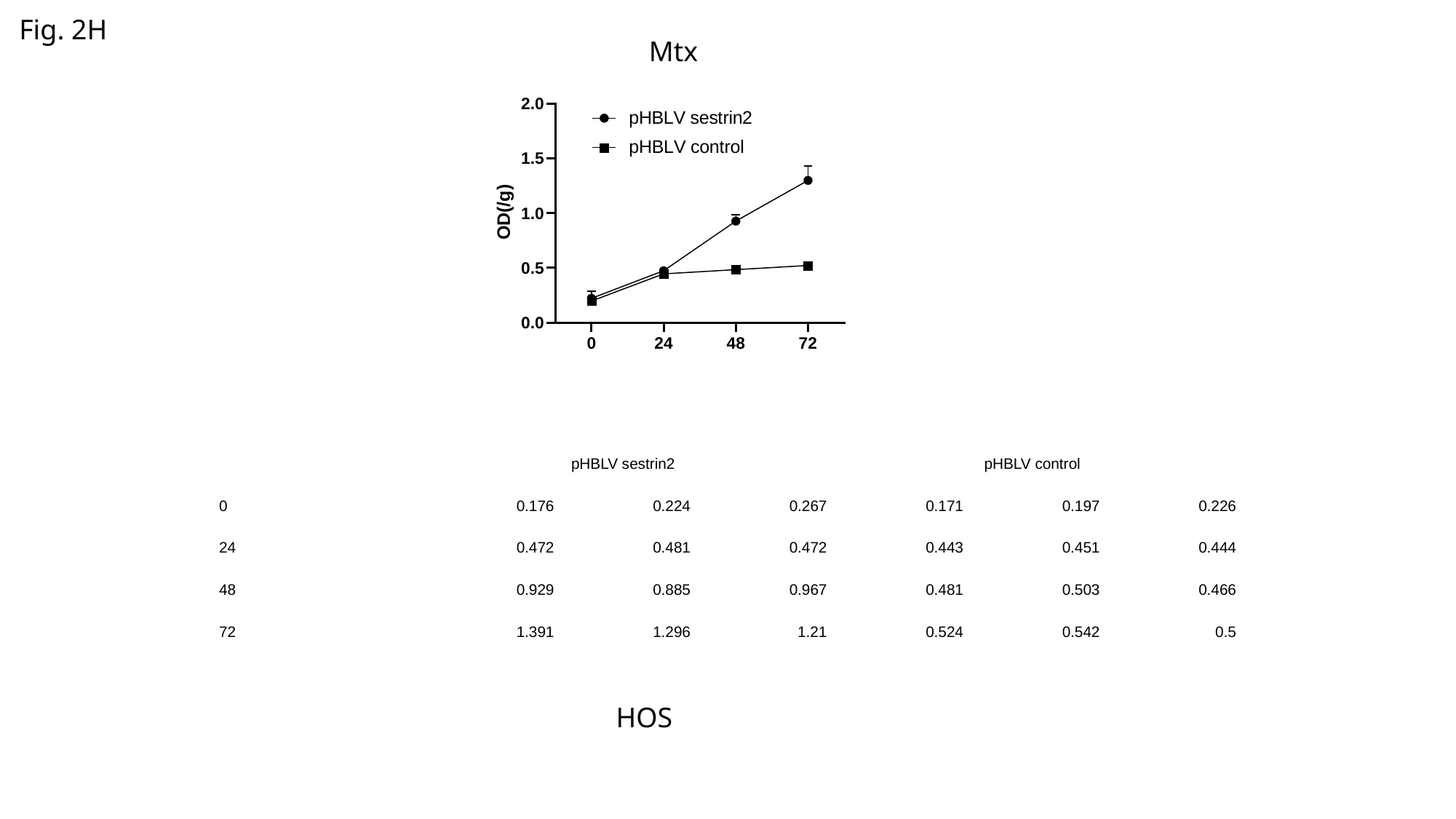

Fig. 2H
Mtx
| | | pHBLV sestrin2 | | | pHBLV control | | |
| --- | --- | --- | --- | --- | --- | --- | --- |
| 0 | | 0.176 | 0.224 | 0.267 | 0.171 | 0.197 | 0.226 |
| 24 | | 0.472 | 0.481 | 0.472 | 0.443 | 0.451 | 0.444 |
| 48 | | 0.929 | 0.885 | 0.967 | 0.481 | 0.503 | 0.466 |
| 72 | | 1.391 | 1.296 | 1.21 | 0.524 | 0.542 | 0.5 |
HOS

## Slide 13
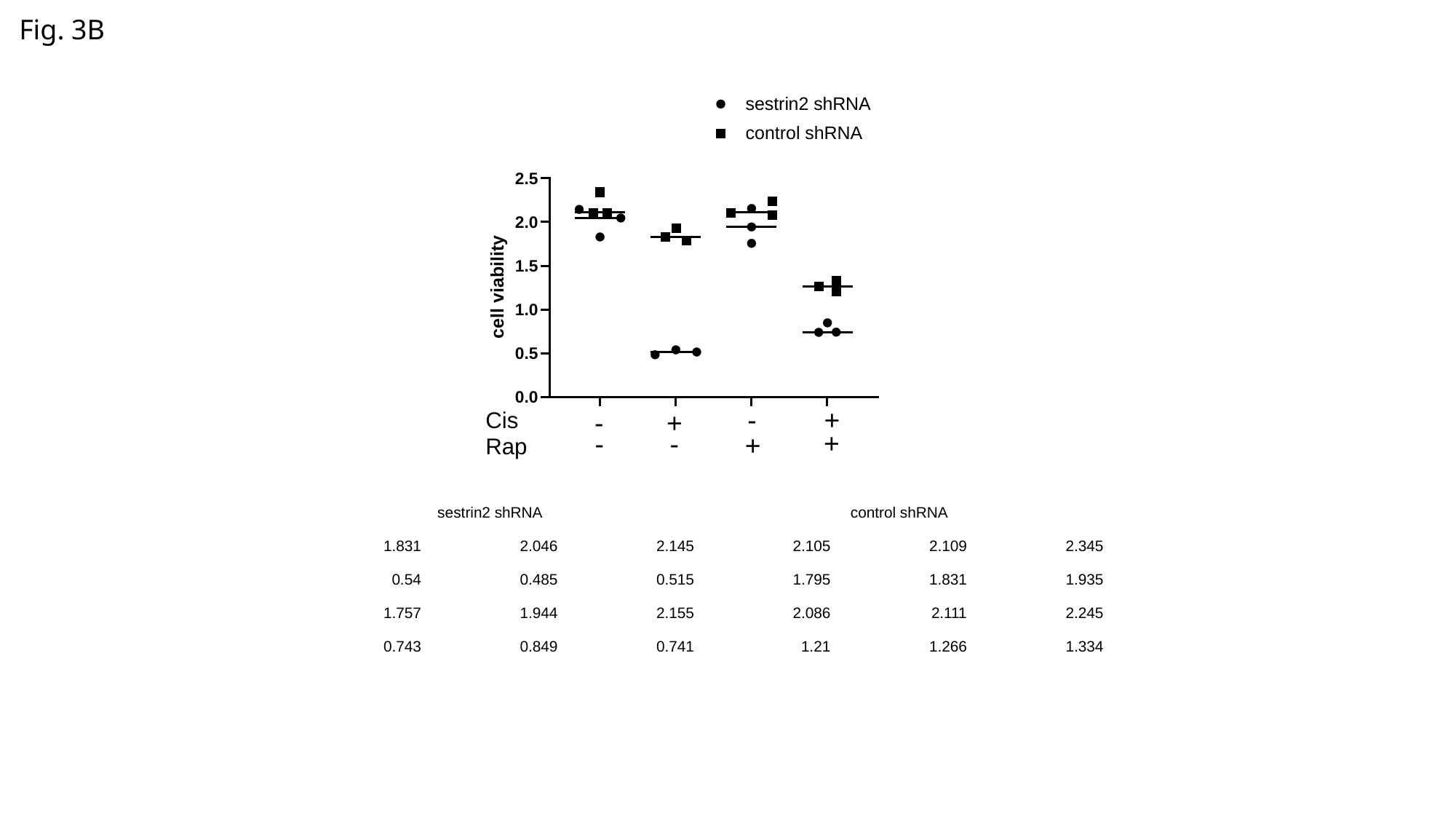

Fig. 3B
-
+
Cis
-
+
+
-
-
+
Rap
| sestrin2 shRNA | | | control shRNA | | |
| --- | --- | --- | --- | --- | --- |
| 1.831 | 2.046 | 2.145 | 2.105 | 2.109 | 2.345 |
| 0.54 | 0.485 | 0.515 | 1.795 | 1.831 | 1.935 |
| 1.757 | 1.944 | 2.155 | 2.086 | 2.111 | 2.245 |
| 0.743 | 0.849 | 0.741 | 1.21 | 1.266 | 1.334 |

## Slide 14
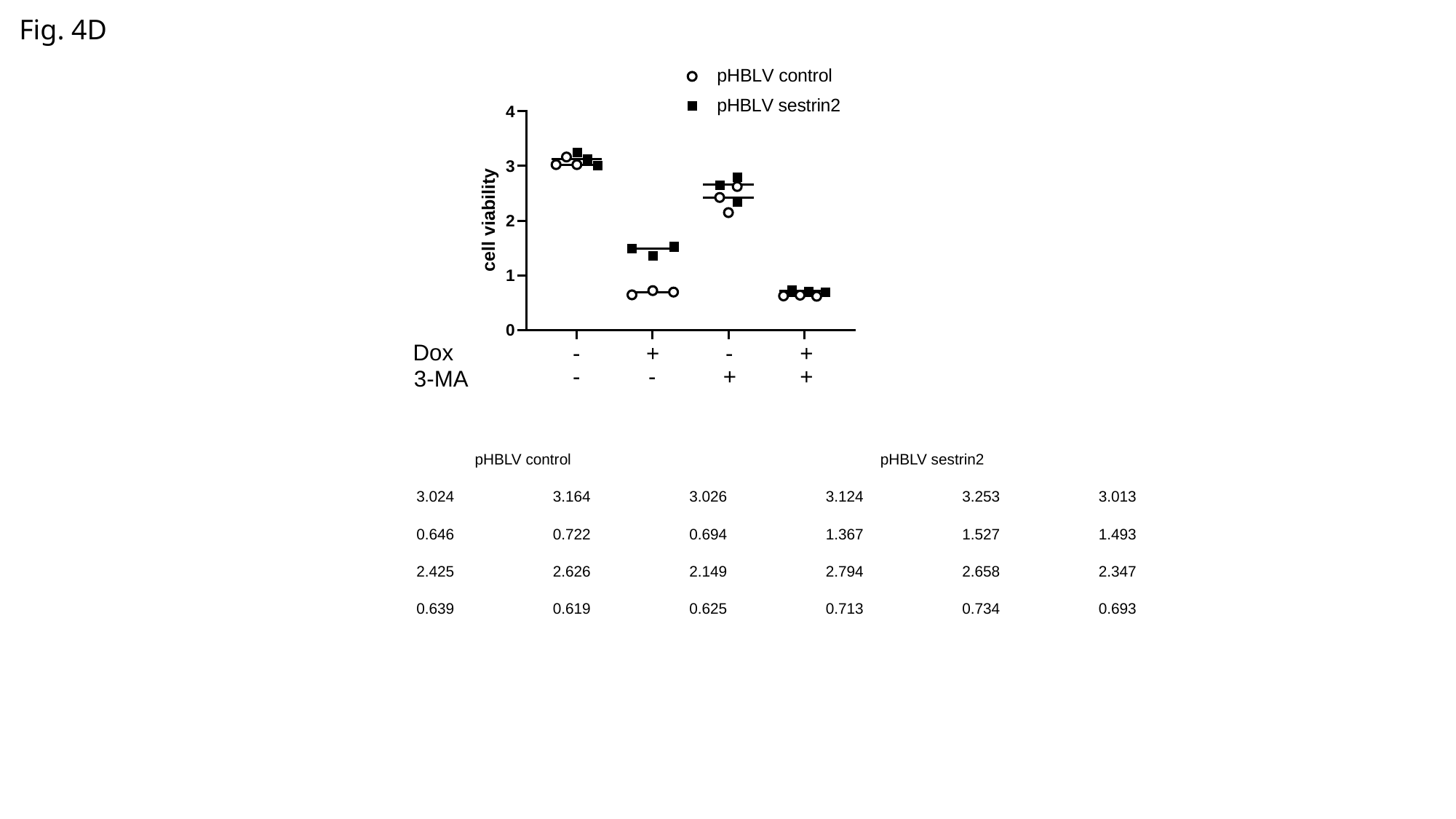

Fig. 4D
Dox
+
-
-
+
+
-
-
+
3-MA
| pHBLV control | | | pHBLV sestrin2 | | |
| --- | --- | --- | --- | --- | --- |
| 3.024 | 3.164 | 3.026 | 3.124 | 3.253 | 3.013 |
| 0.646 | 0.722 | 0.694 | 1.367 | 1.527 | 1.493 |
| 2.425 | 2.626 | 2.149 | 2.794 | 2.658 | 2.347 |
| 0.639 | 0.619 | 0.625 | 0.713 | 0.734 | 0.693 |
