# Supplementary material for: Sestrin2-Mediated Autophagy Contributes to Drug Resistance via Endoplasmic Reticulum Stress in Human Osteosarcoma
Source: Front Cell Dev Biol. 2021 Sep 27;9:722960. doi: 10.3389/fcell.2021.722960 (PMC8502982; doi:10.3389/fcell.2021.722960)
Supplement: Supplementary file 6 [file Data_Sheet_7.ZIP › Raw data of colony formation assay/Raw data of colony formation assay.pptx]

## Slide 1
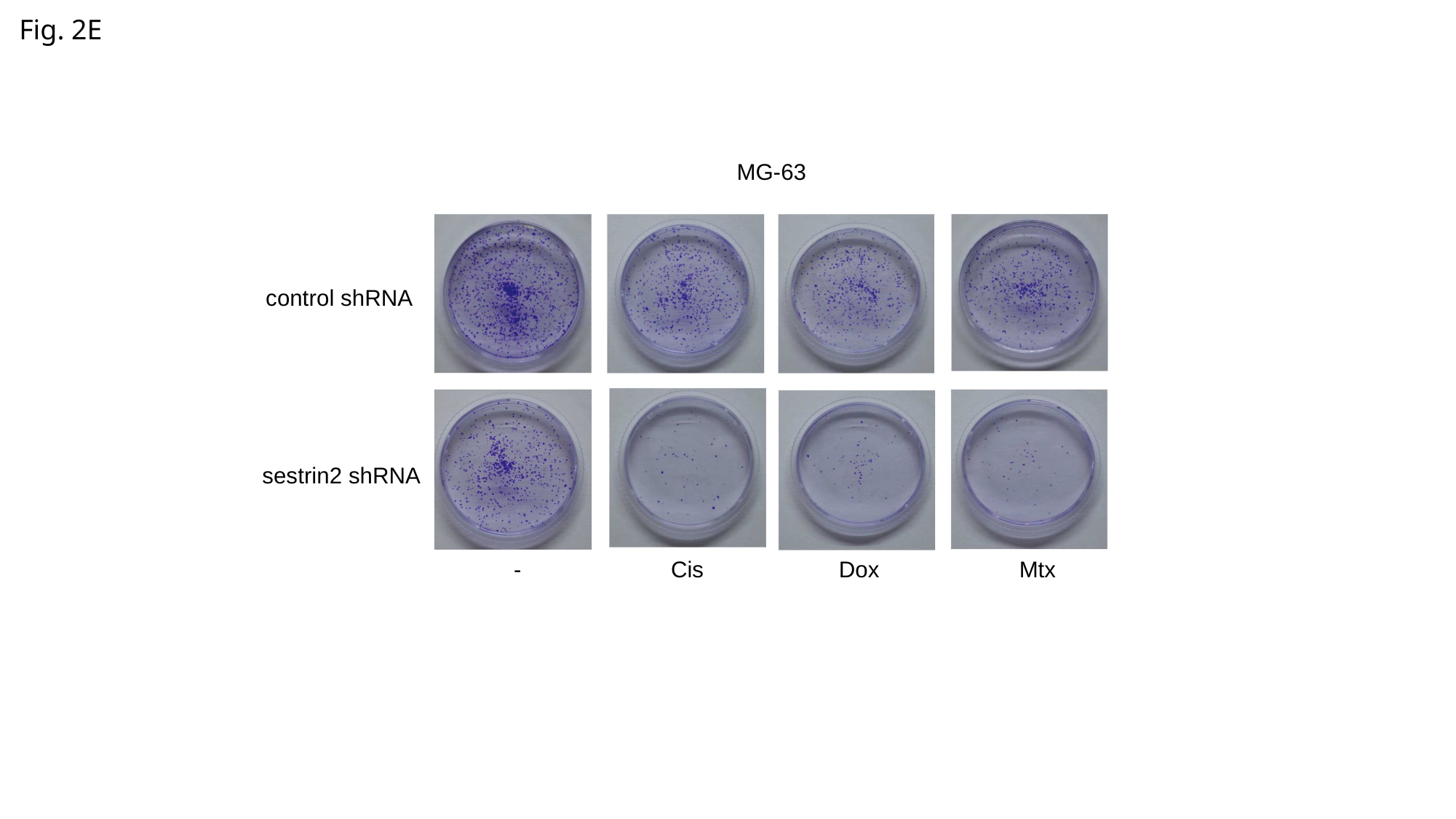

Fig. 2E
MG-63
control shRNA
sestrin2 shRNA
-
Dox
Mtx
Cis

## Slide 2
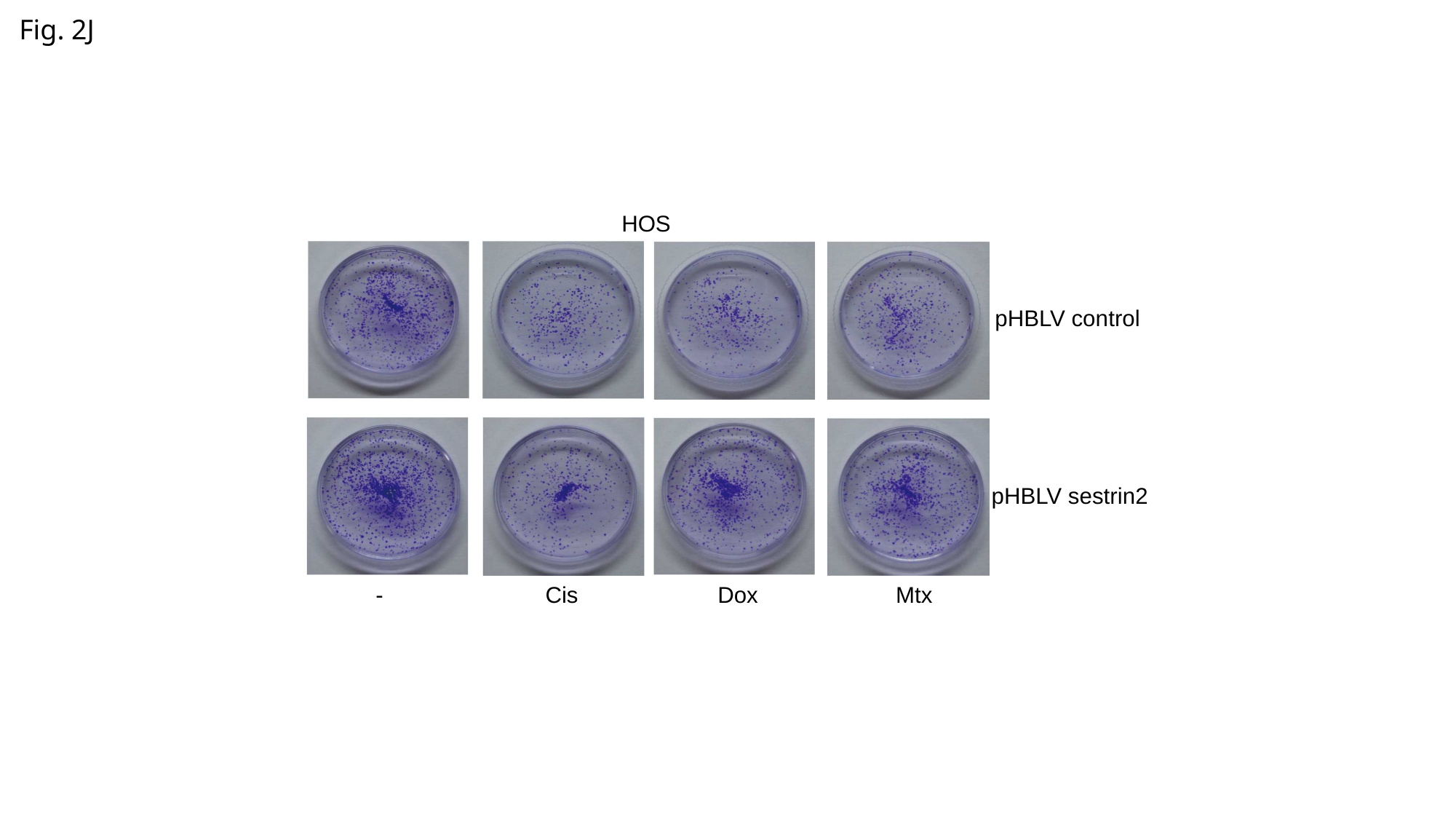

Fig. 2J
HOS
pHBLV control
pHBLV sestrin2
-
Mtx
Cis
Dox
