# Supplementary material for: Sestrin2-Mediated Autophagy Contributes to Drug Resistance via Endoplasmic Reticulum Stress in Human Osteosarcoma
Source: Front Cell Dev Biol. 2021 Sep 27;9:722960. doi: 10.3389/fcell.2021.722960 (PMC8502982; doi:10.3389/fcell.2021.722960)
Supplement: Supplementary file 7 [file Data_Sheet_8.ZIP › Raw data of flow cytometry/Raw data of flow cytometry.pptx]

## Slide 1
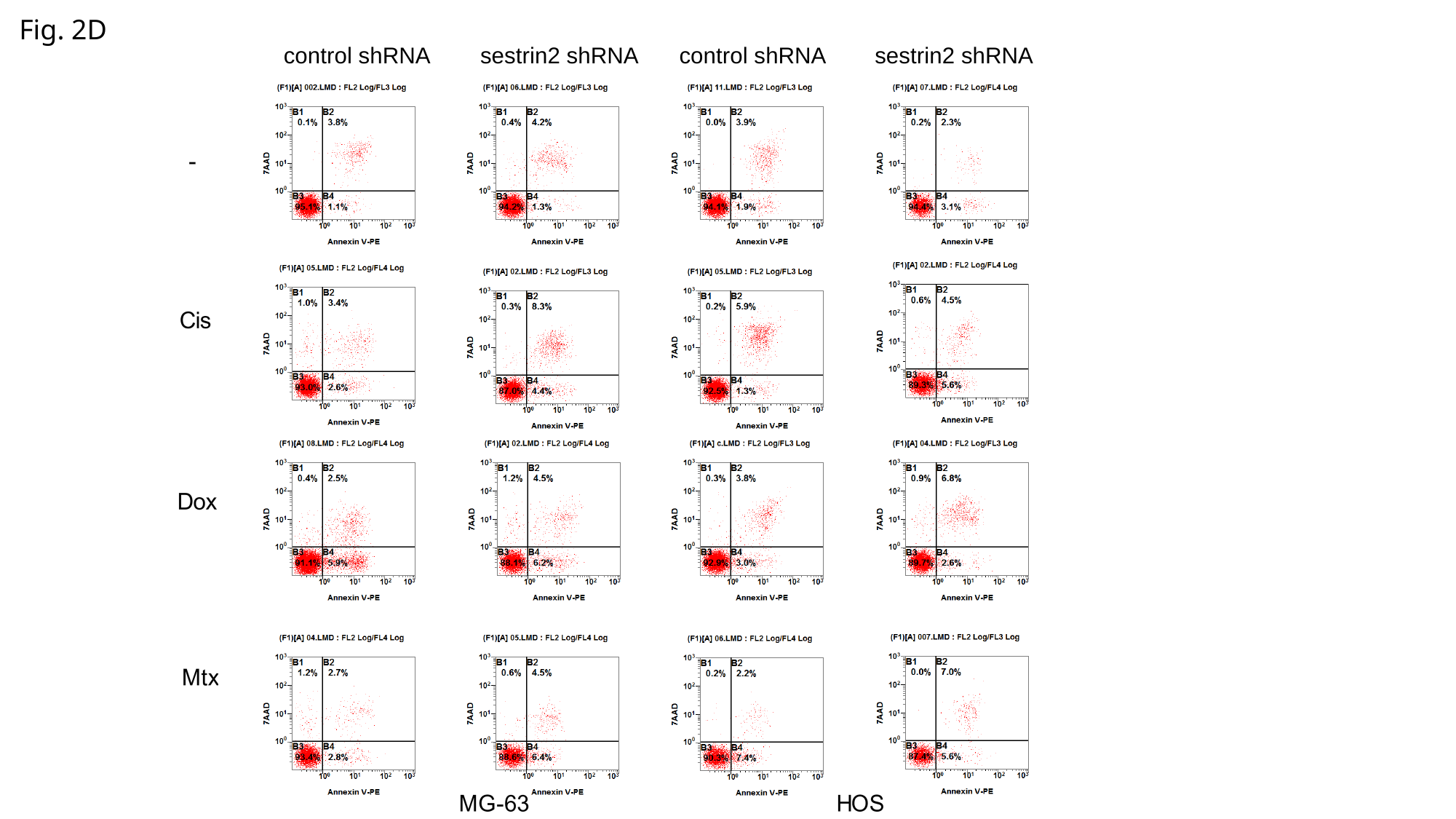

Fig. 2D
control shRNA
sestrin2 shRNA
control shRNA
sestrin2 shRNA

## Slide 2
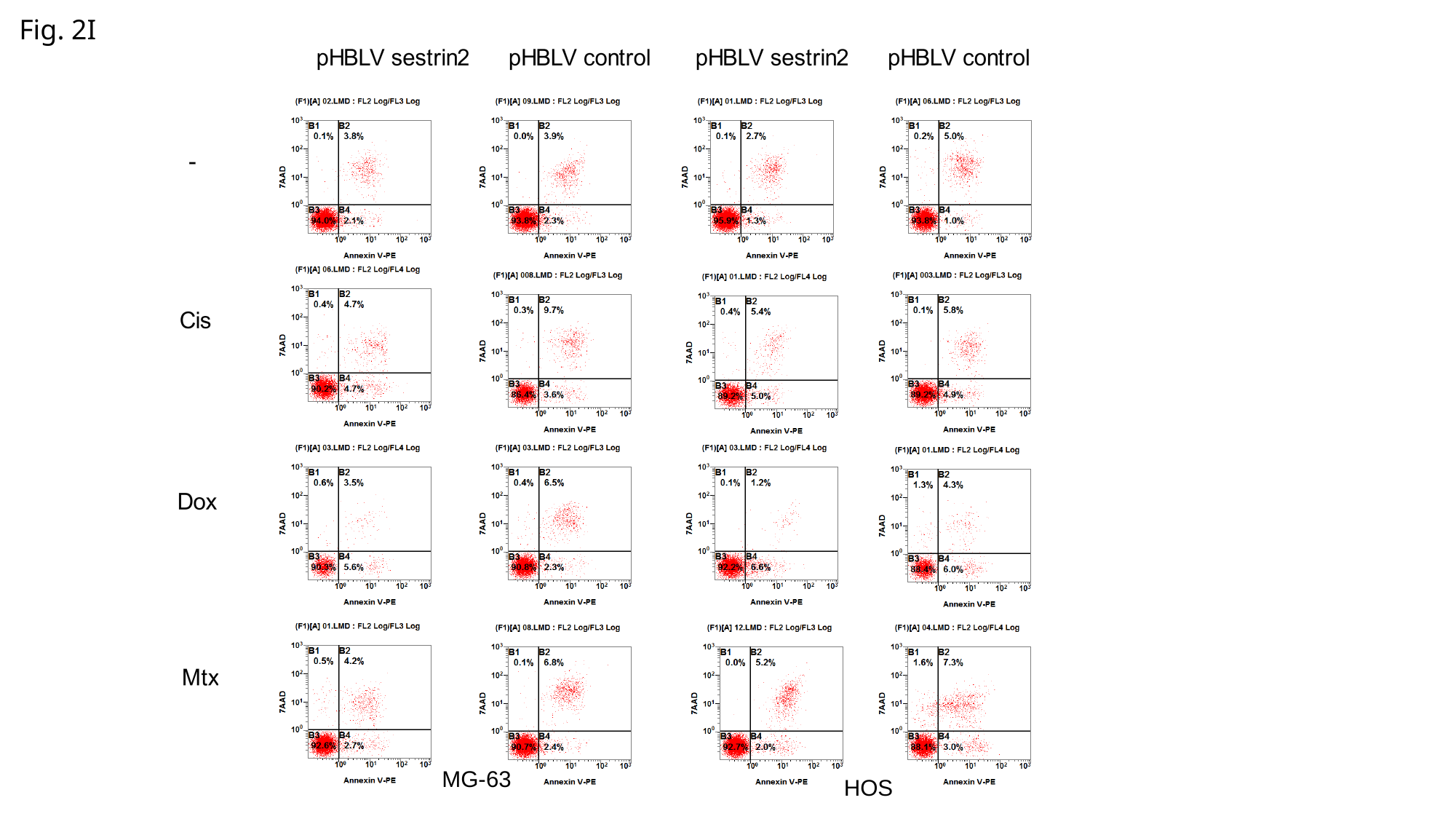

Fig. 2I
MG-63
HOS

## Slide 3
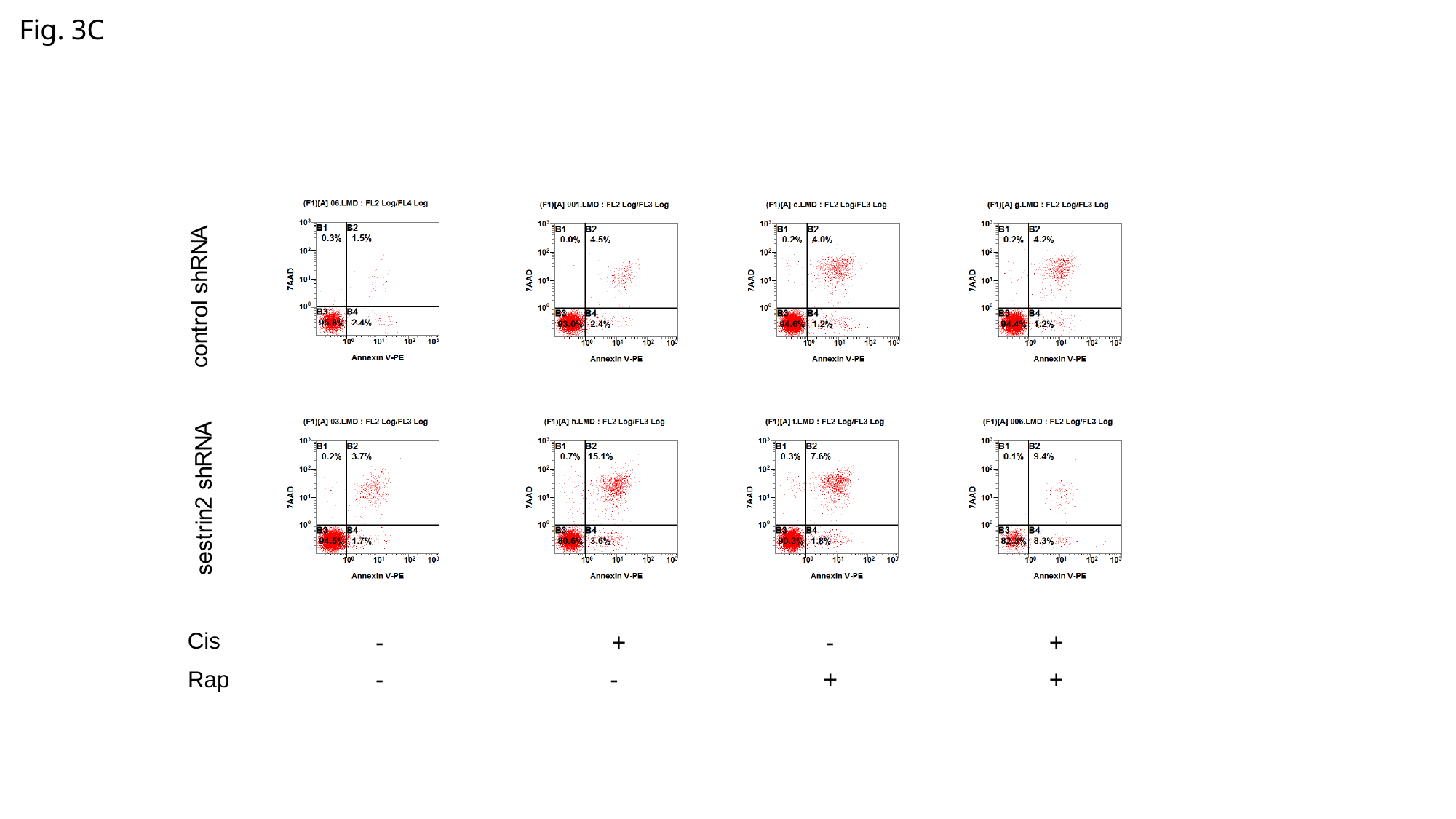

Fig. 3C
+
+
Cis
-
-
-
+
+
-
Rap

## Slide 4
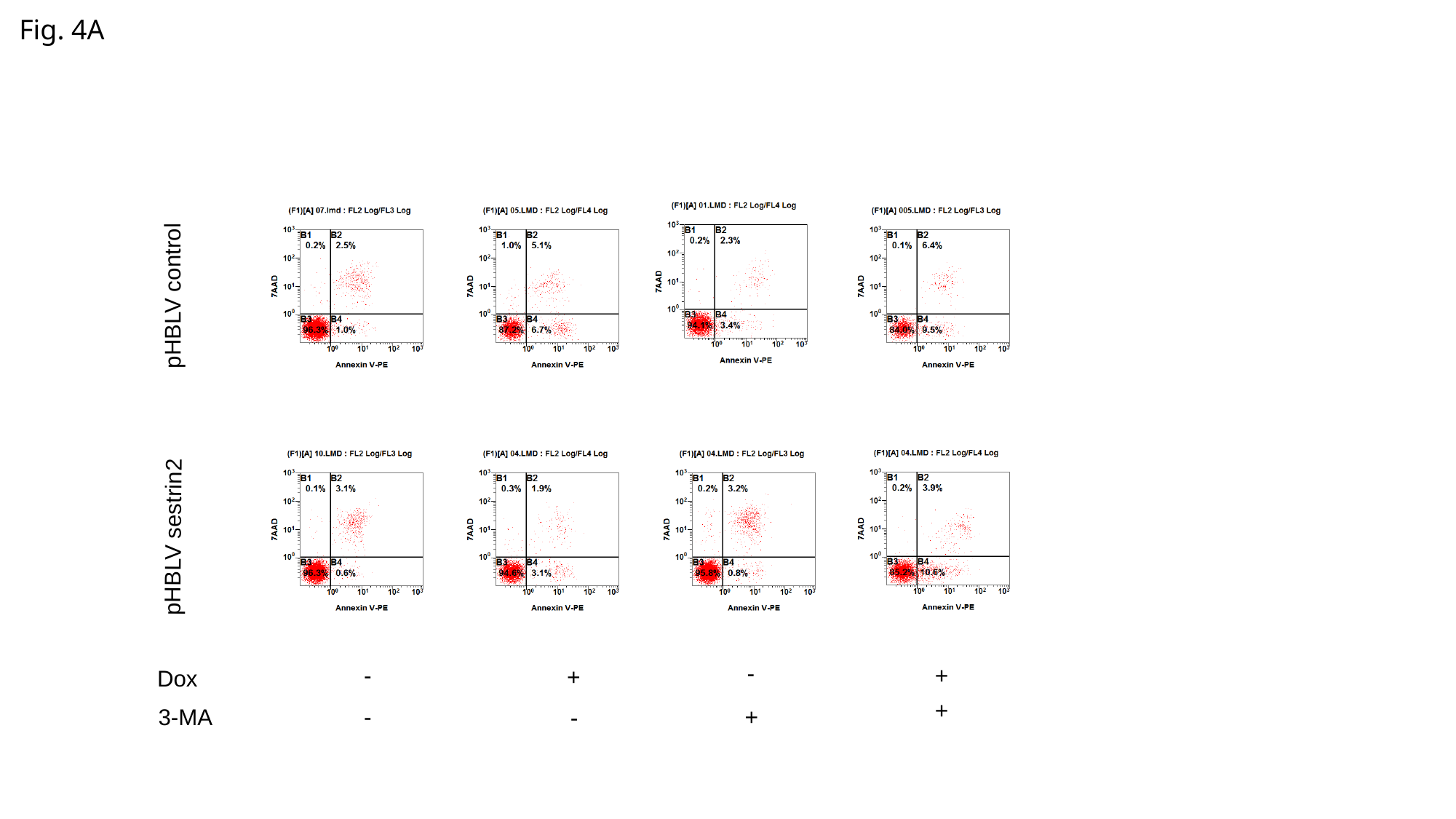

Fig. 4A
pHBLV control
pHBLV sestrin2
-
-
+
+
Dox
+
+
-
3-MA
-

## Slide 5
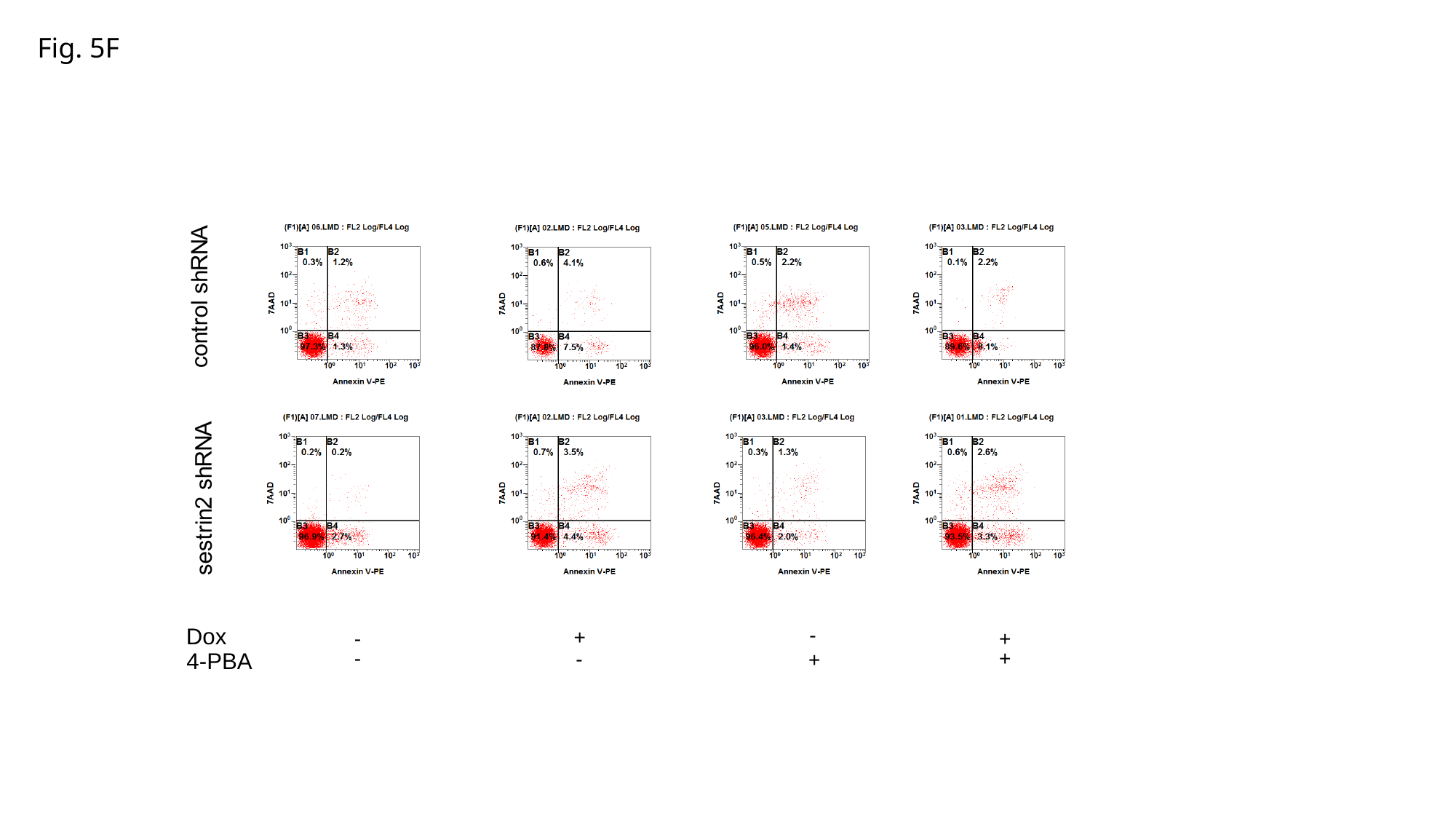

Fig. 5F
Dox
-
+
-
+
-
+
-
4-PBA
+
