# Supplementary material for: Sestrin2-Mediated Autophagy Contributes to Drug Resistance via Endoplasmic Reticulum Stress in Human Osteosarcoma
Source: Front Cell Dev Biol. 2021 Sep 27;9:722960. doi: 10.3389/fcell.2021.722960 (PMC8502982; doi:10.3389/fcell.2021.722960)
Supplement: Supplementary file 8 [file Data_Sheet_9.ZIP › Raw data of immunofluorescence in vivo/Raw data of immunofluorescence in vivo.pptx]

## Slide 1
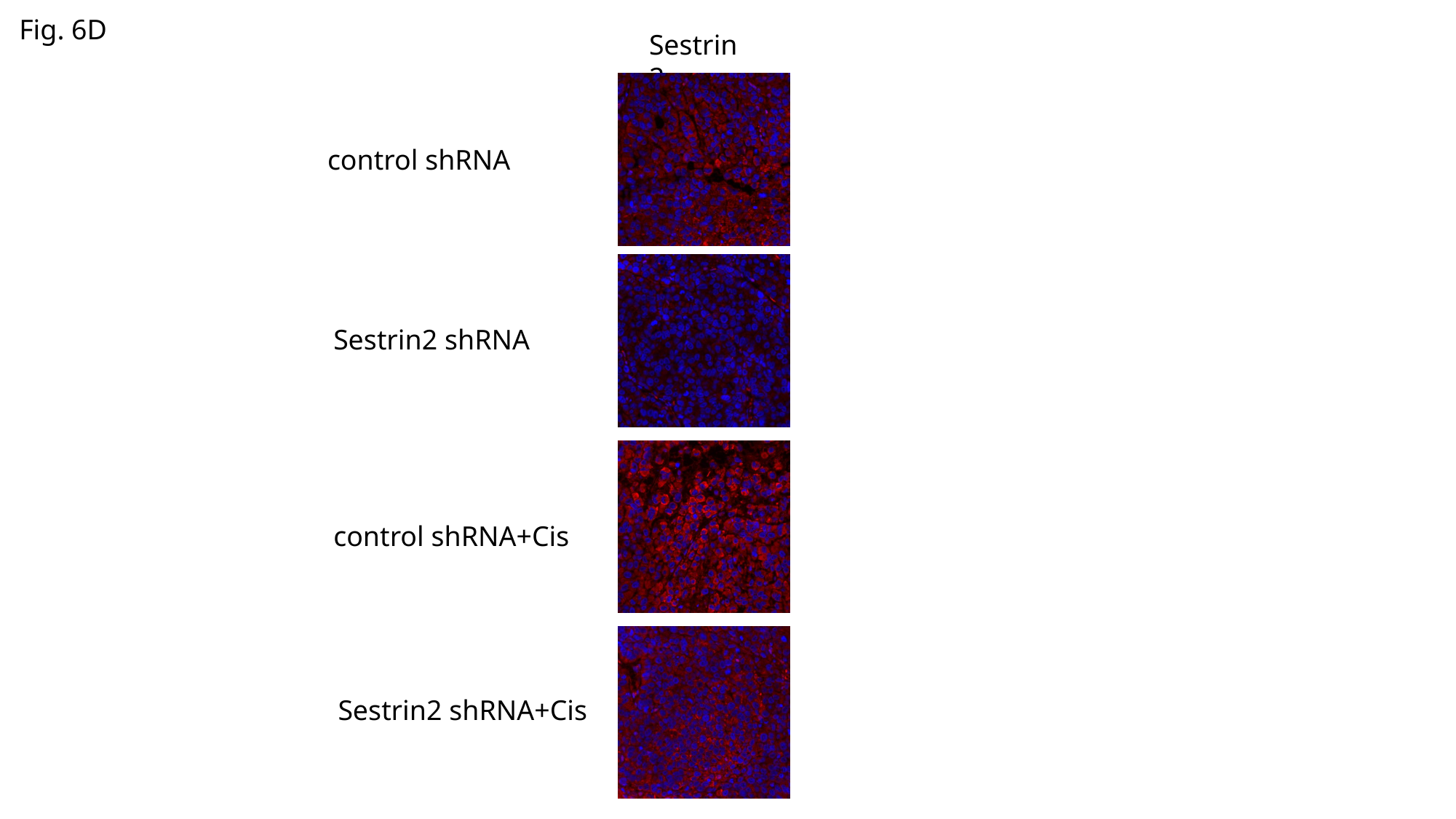

Fig. 6D
Sestrin 2
control shRNA
Sestrin2 shRNA
control shRNA+Cis
Sestrin2 shRNA+Cis

## Slide 2
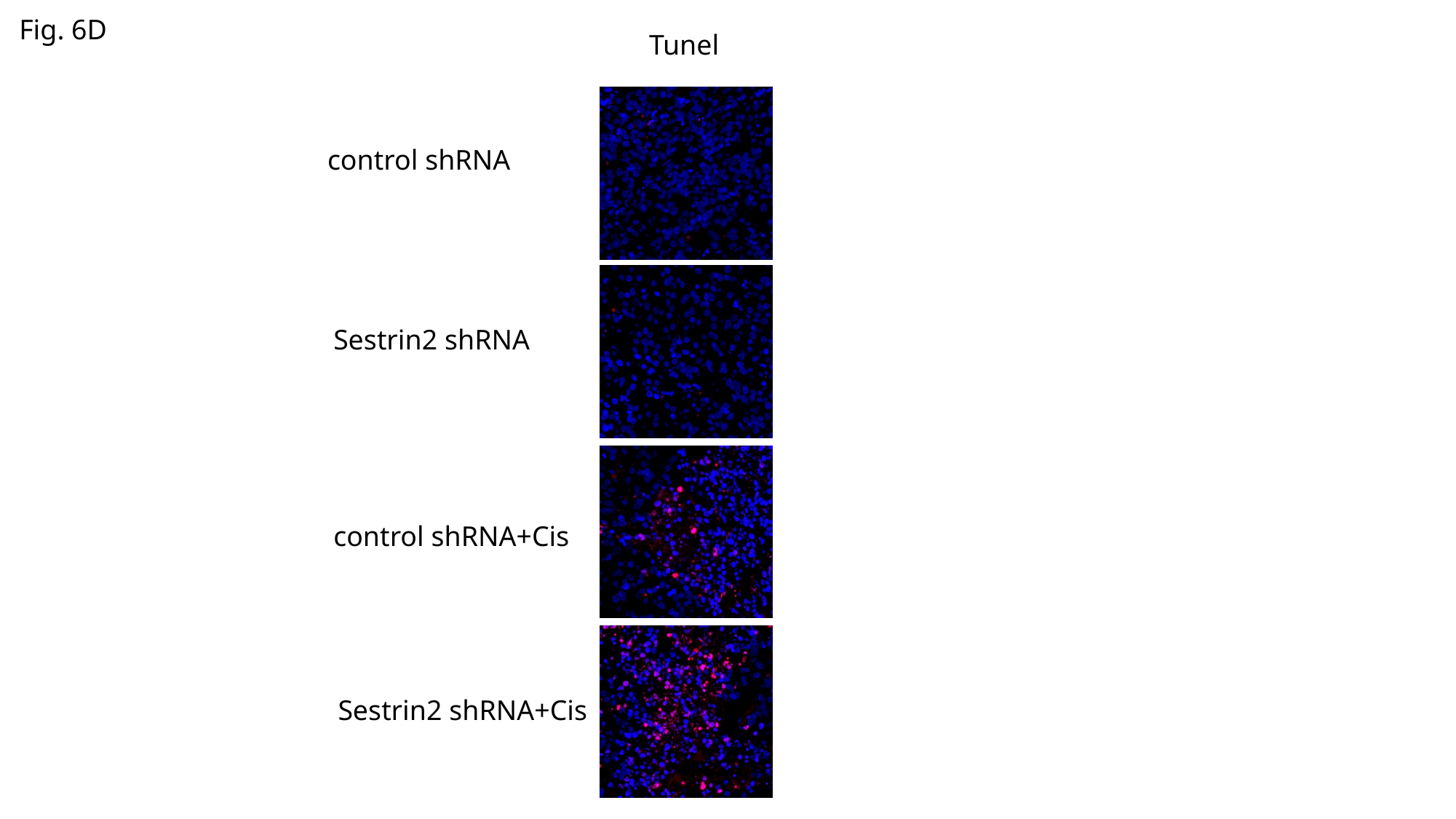

Fig. 6D
Tunel
control shRNA
Sestrin2 shRNA
control shRNA+Cis
Sestrin2 shRNA+Cis

## Slide 3
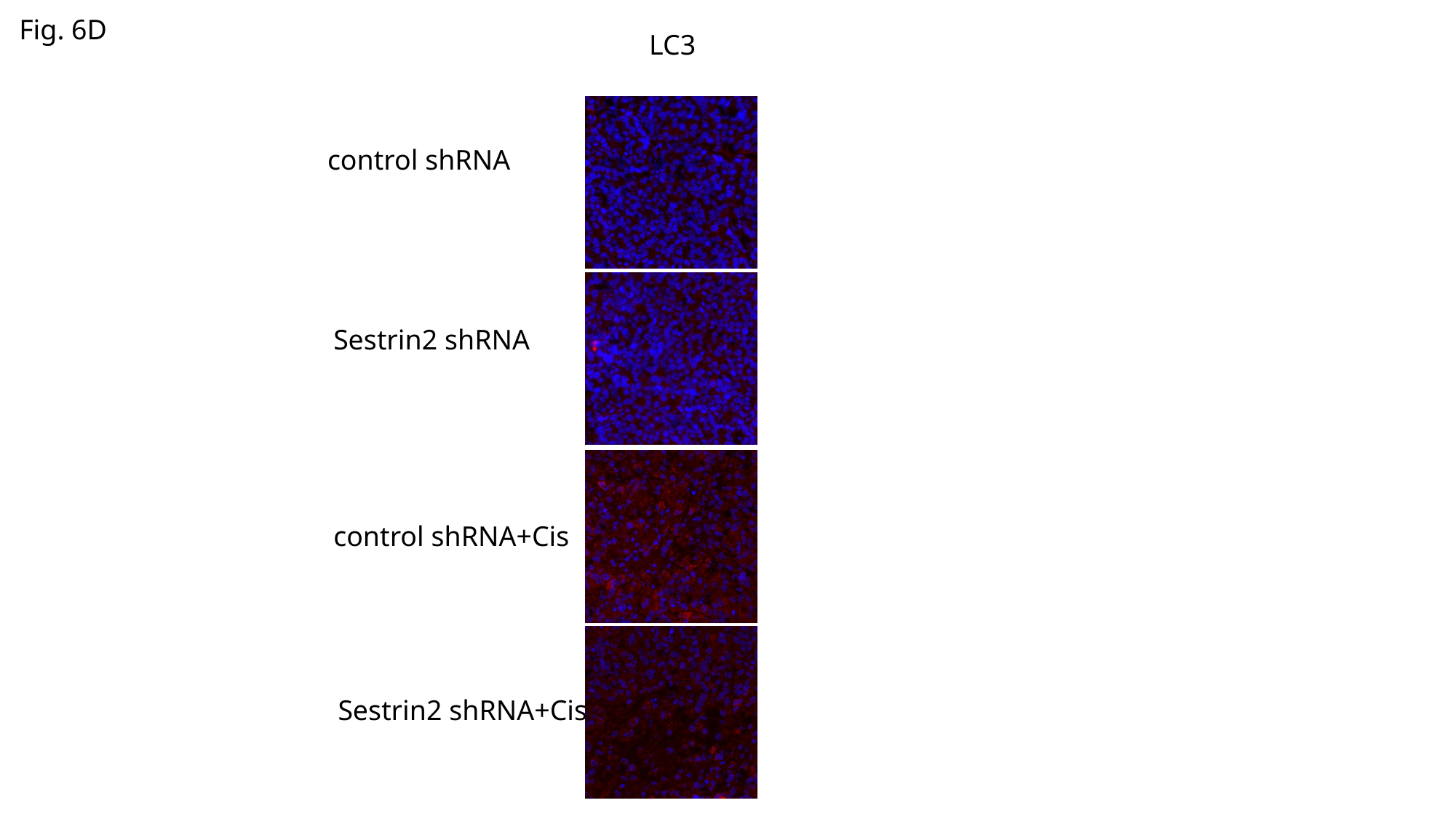

Fig. 6D
LC3
control shRNA
Sestrin2 shRNA
control shRNA+Cis
Sestrin2 shRNA+Cis

## Slide 4
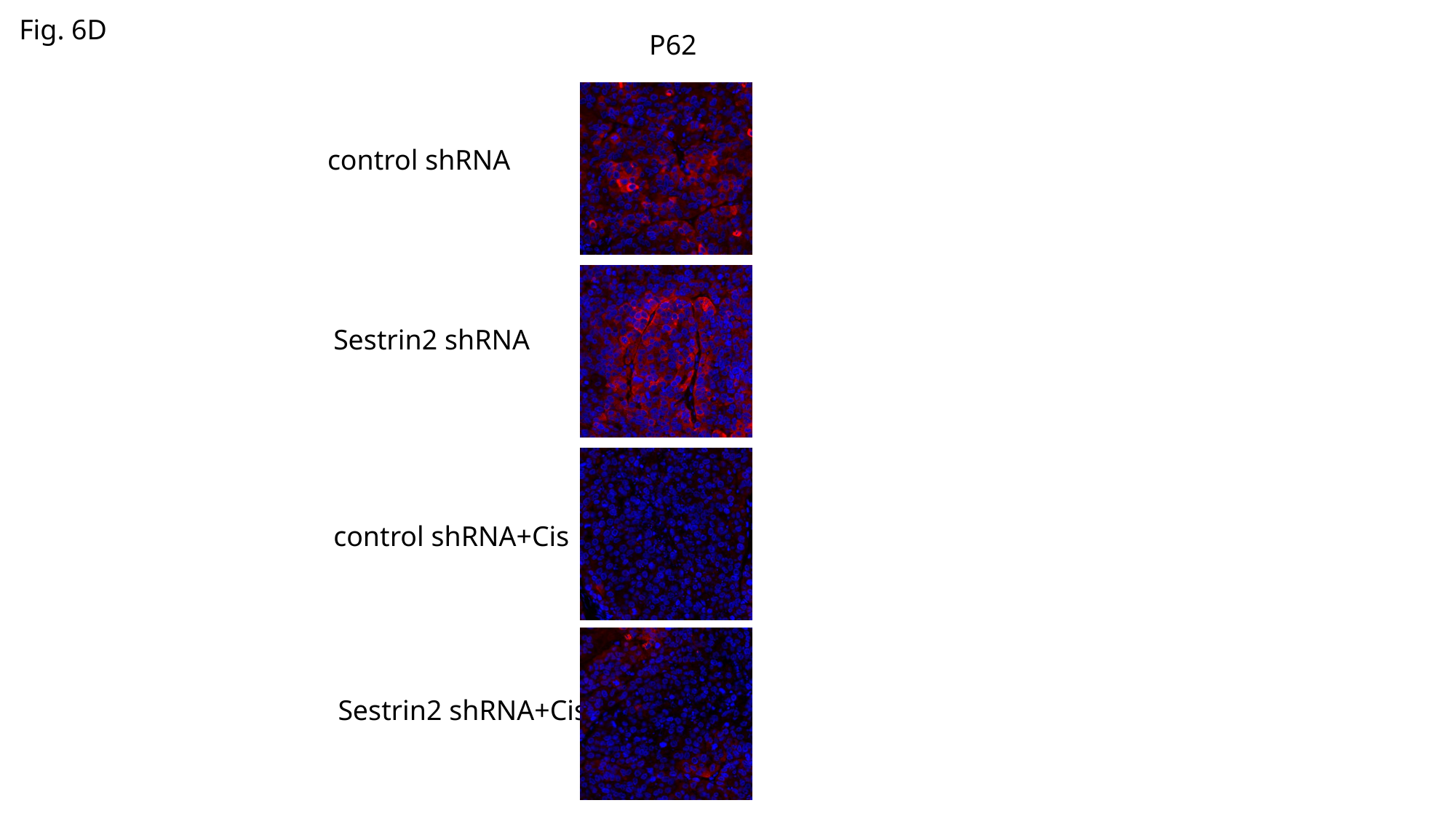

Fig. 6D
P62
control shRNA
Sestrin2 shRNA
control shRNA+Cis
Sestrin2 shRNA+Cis

## Slide 5
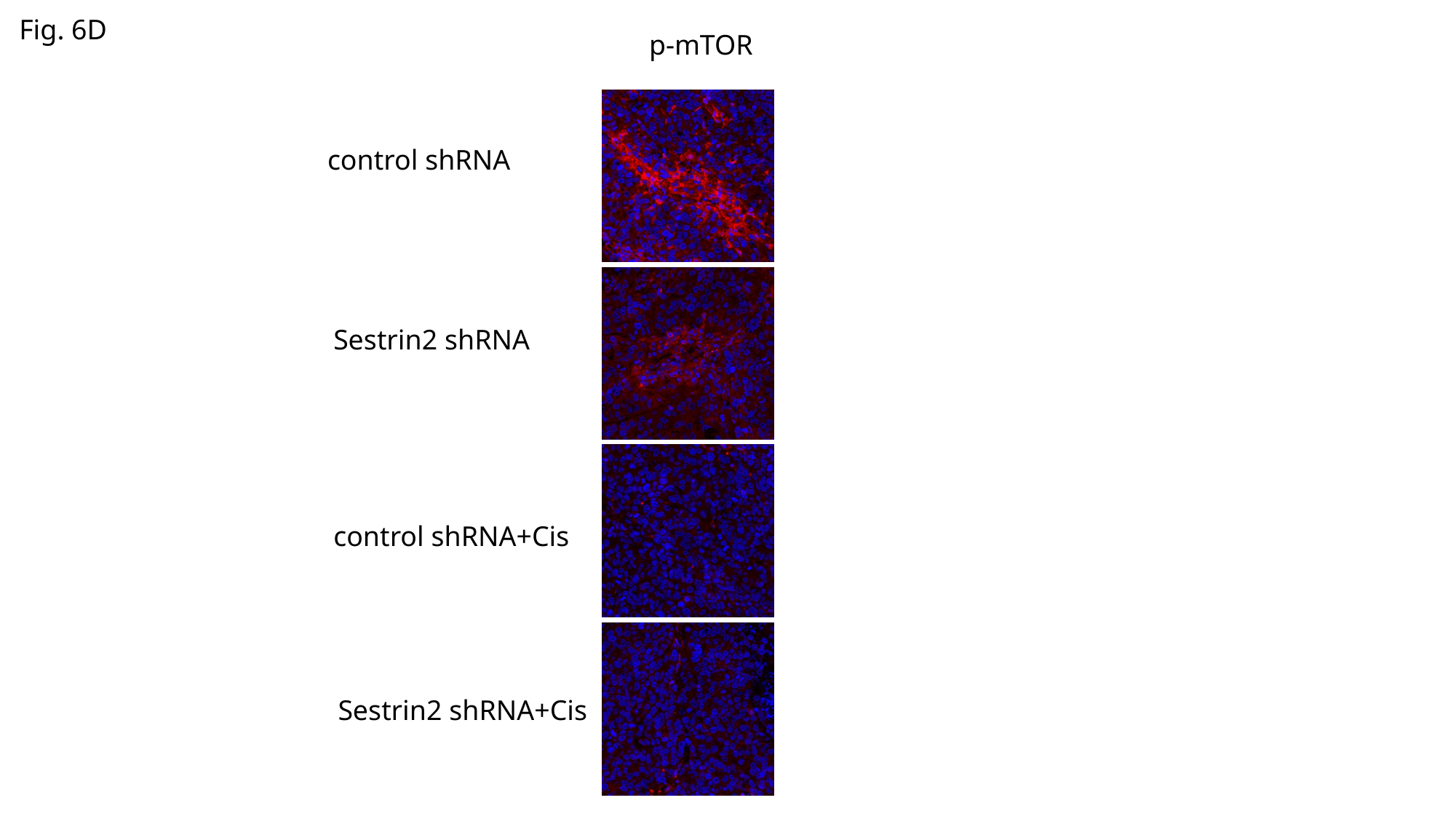

Fig. 6D
p-mTOR
control shRNA
Sestrin2 shRNA
control shRNA+Cis
Sestrin2 shRNA+Cis

## Slide 6
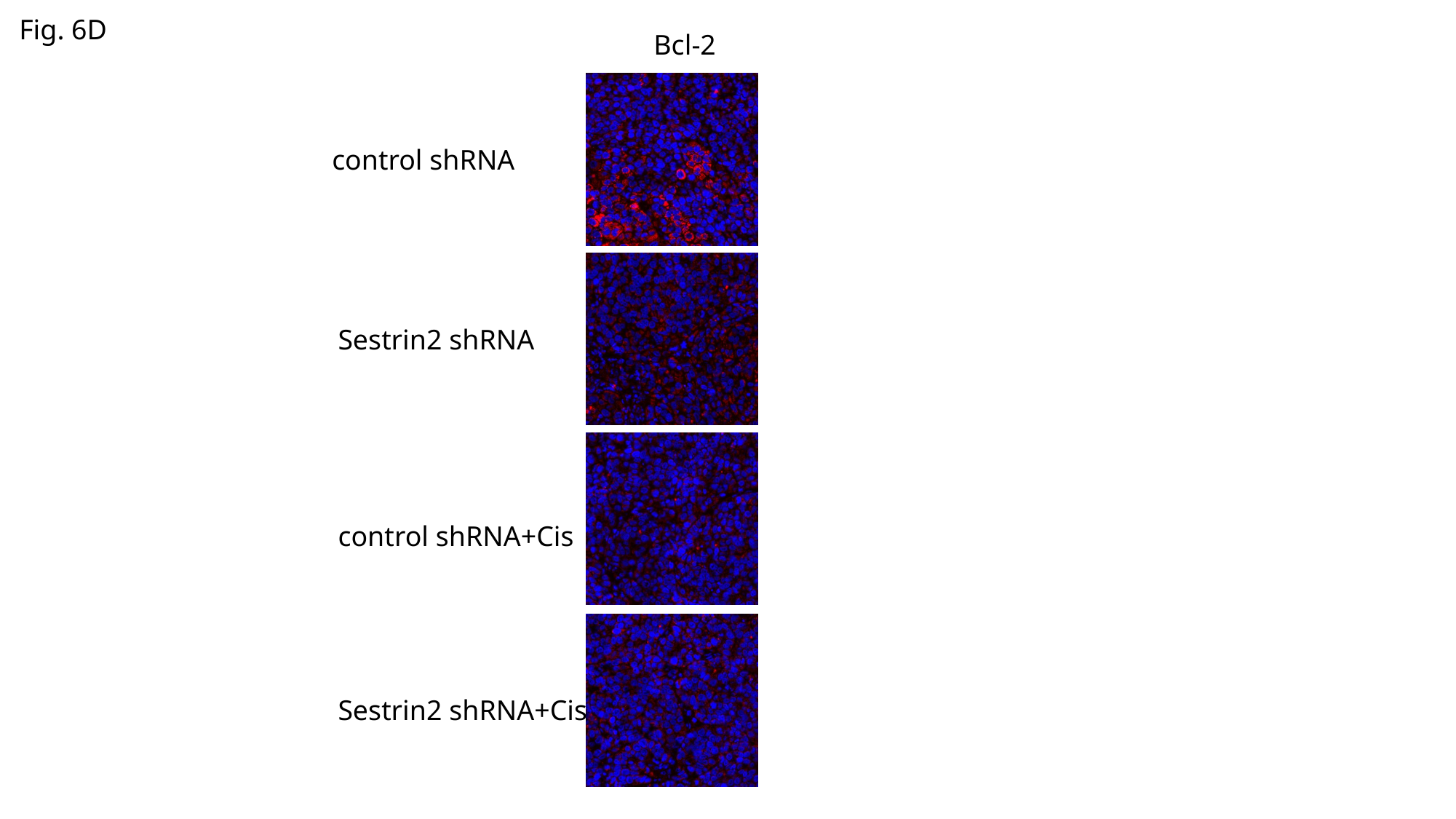

Fig. 6D
Bcl-2
control shRNA
Sestrin2 shRNA
control shRNA+Cis
Sestrin2 shRNA+Cis

## Slide 7
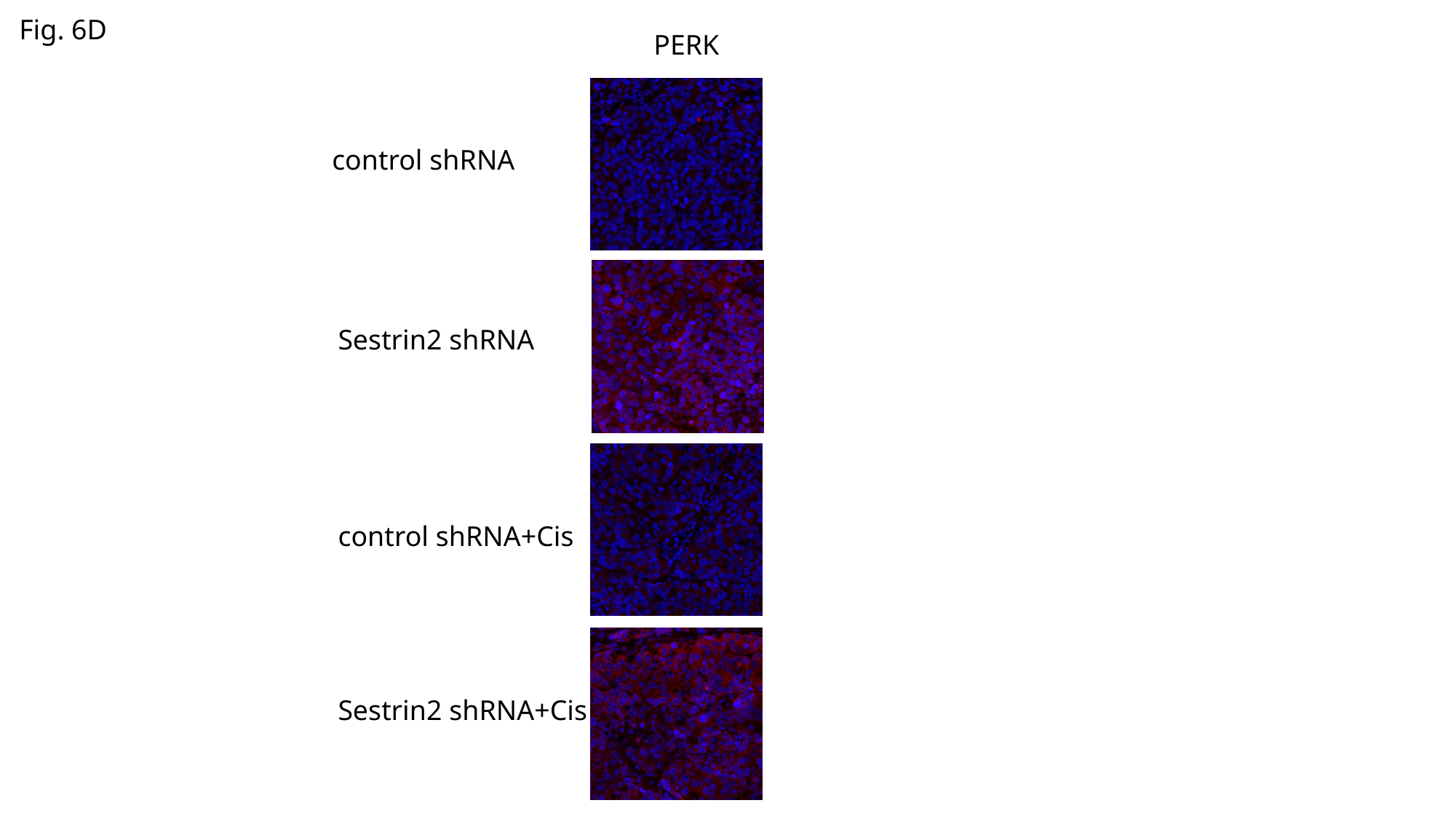

Fig. 6D
PERK
control shRNA
Sestrin2 shRNA
control shRNA+Cis
Sestrin2 shRNA+Cis

## Slide 8
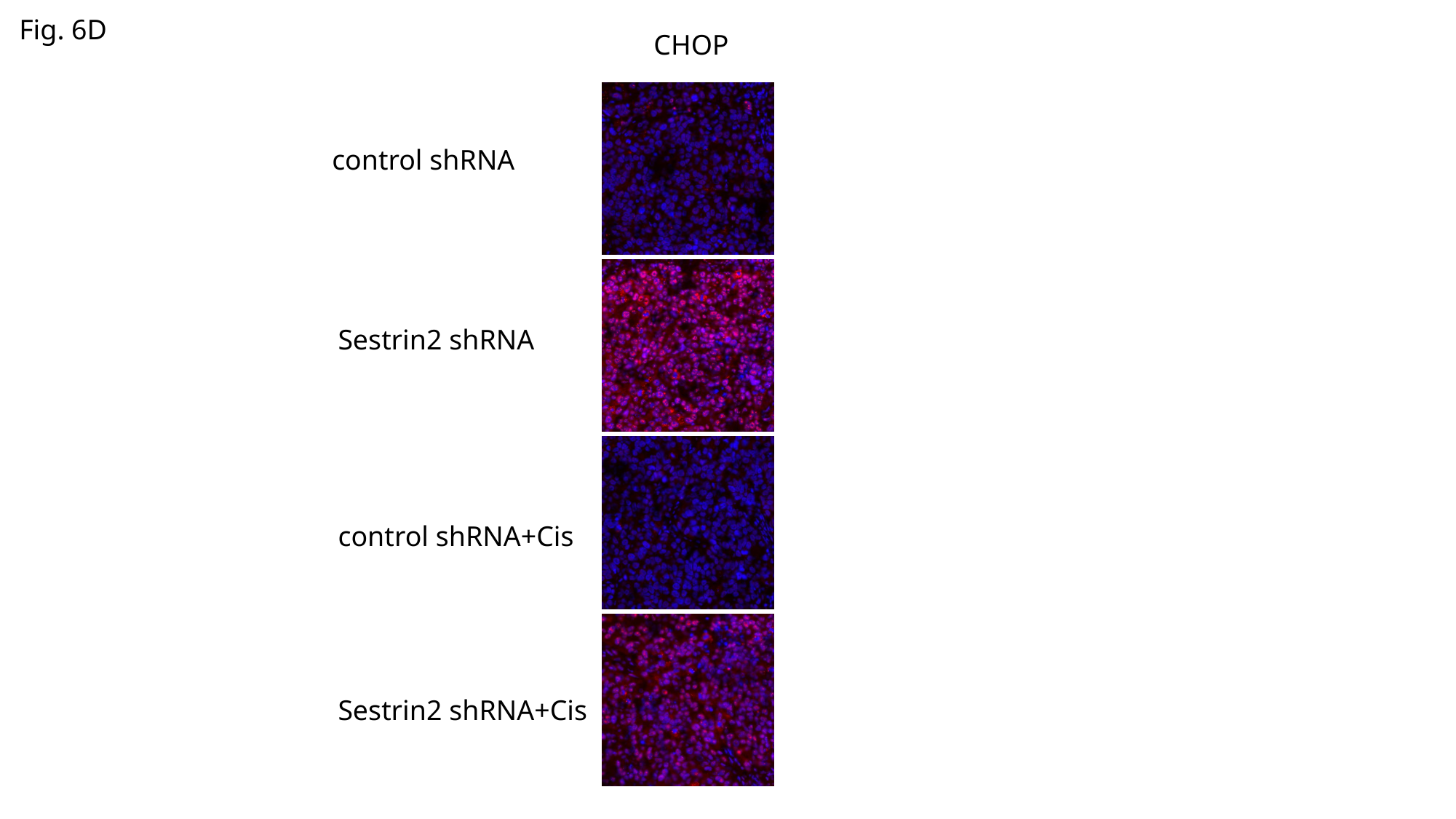

Fig. 6D
CHOP
control shRNA
Sestrin2 shRNA
control shRNA+Cis
Sestrin2 shRNA+Cis

## Slide 9
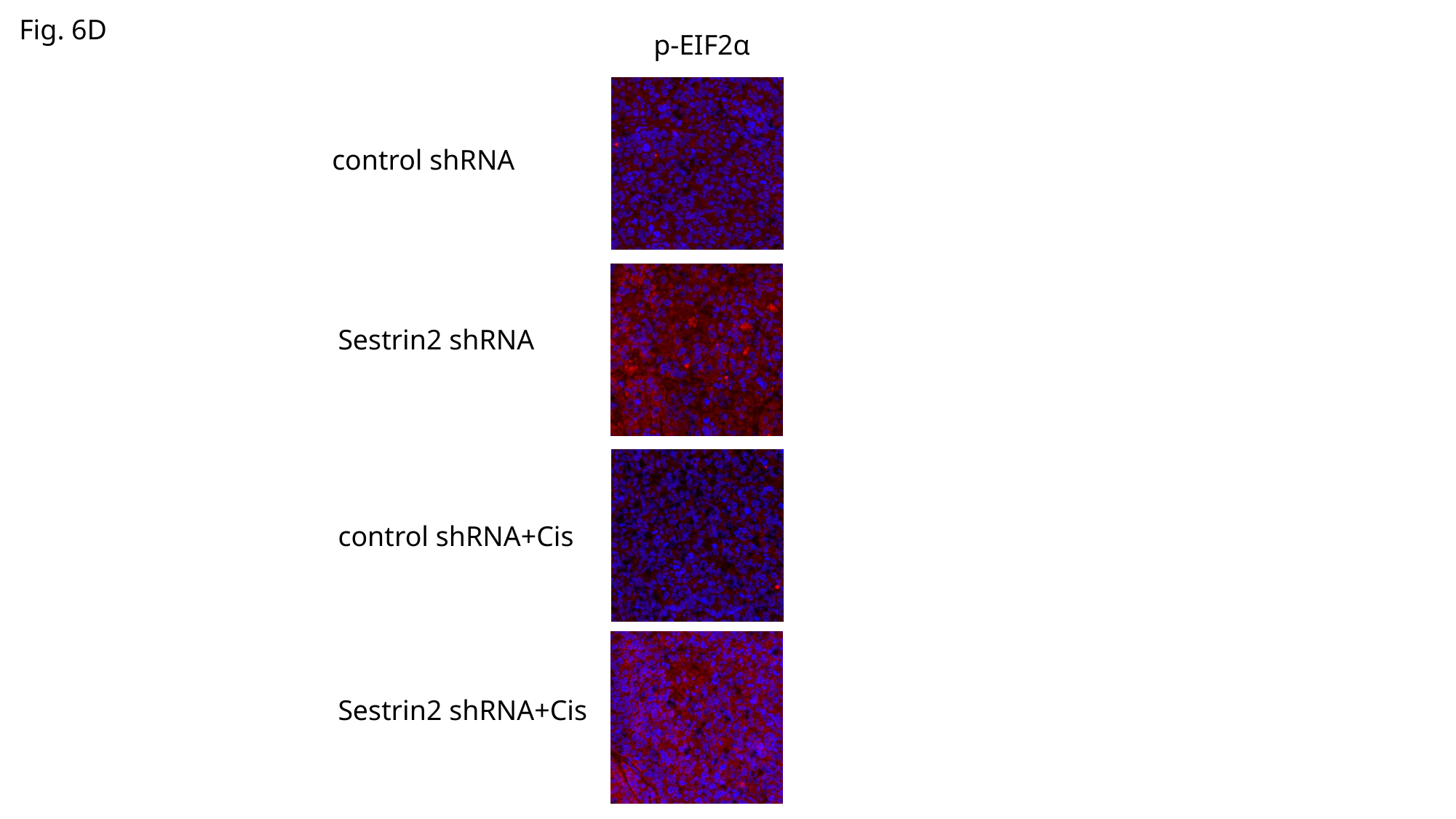

Fig. 6D
p-EIF2α
control shRNA
Sestrin2 shRNA
control shRNA+Cis
Sestrin2 shRNA+Cis
