# Supplementary material for: Sestrin2-Mediated Autophagy Contributes to Drug Resistance via Endoplasmic Reticulum Stress in Human Osteosarcoma
Source: Front Cell Dev Biol. 2021 Sep 27;9:722960. doi: 10.3389/fcell.2021.722960 (PMC8502982; doi:10.3389/fcell.2021.722960)
Supplement: Supplementary file 10 [file Data_Sheet_11.ZIP › Raw data of transmission electron microscopy/Raw data of transmission electron microscopy.pptx]

## Slide 1
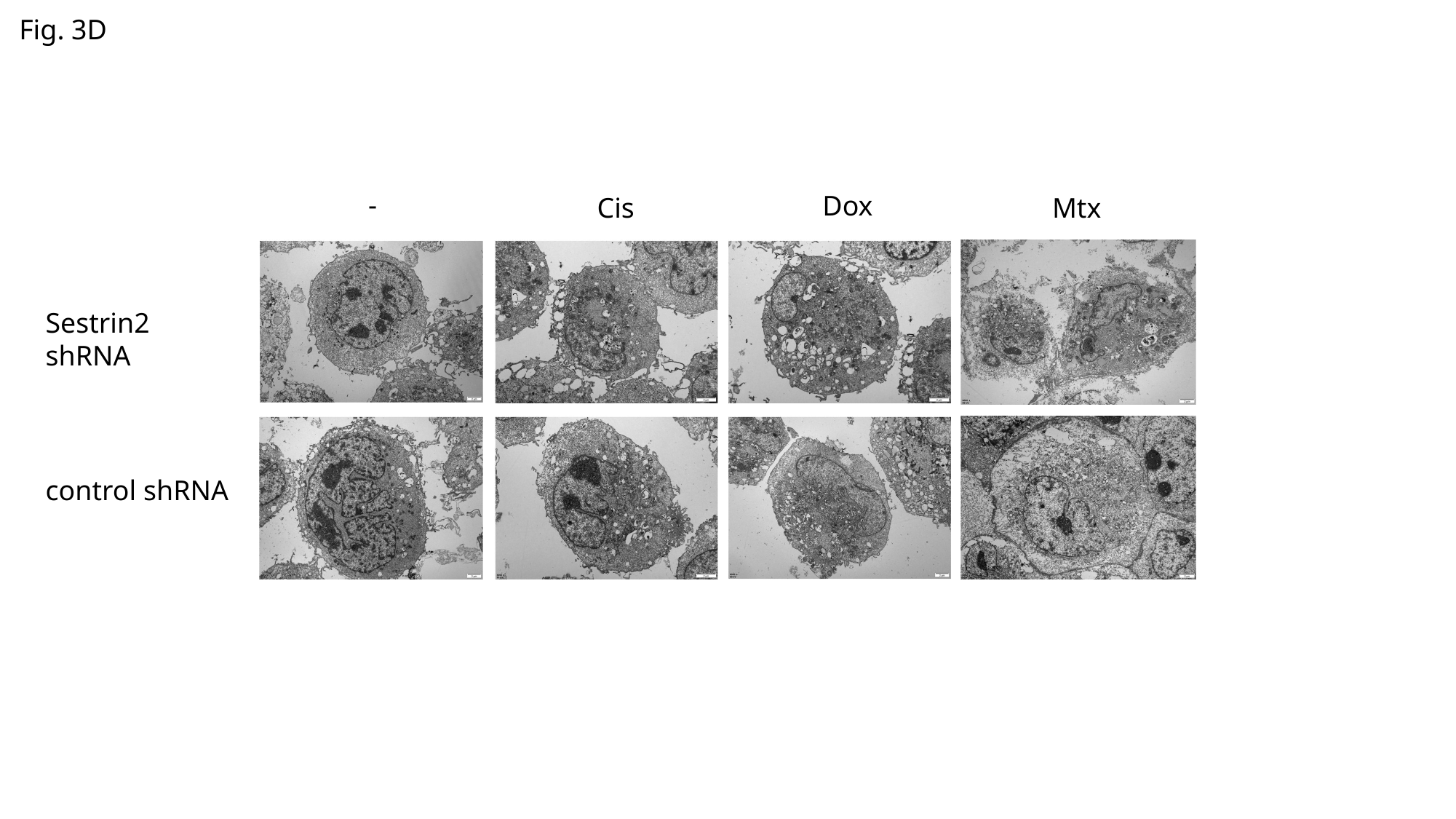

Fig. 3D
-
Dox
Cis
Mtx
Sestrin2 shRNA
control shRNA

## Slide 2
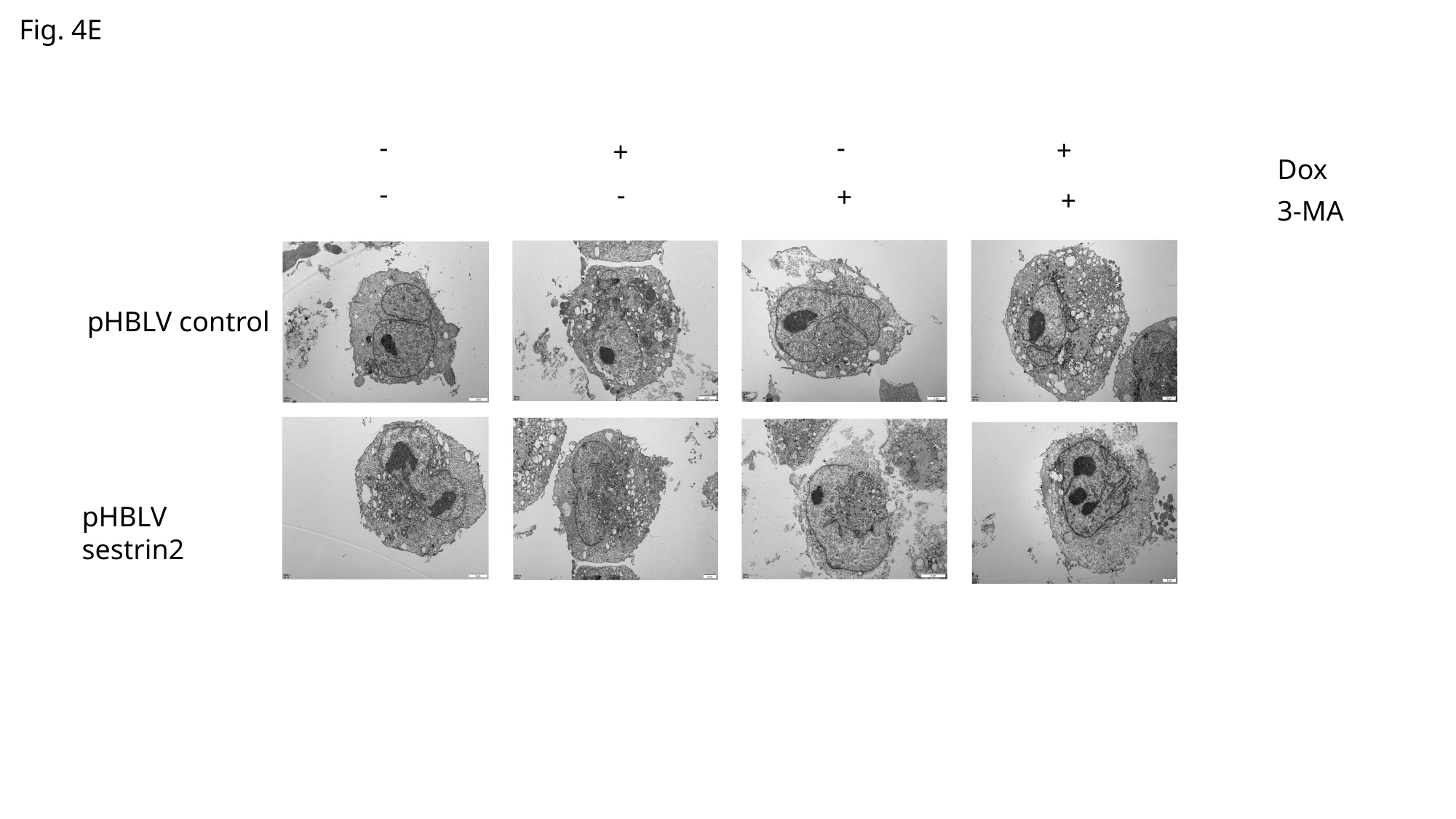

Fig. 4E
-
-
+
+
Dox
-
-
+
+
3-MA
pHBLV control
pHBLV sestrin2

## Slide 3
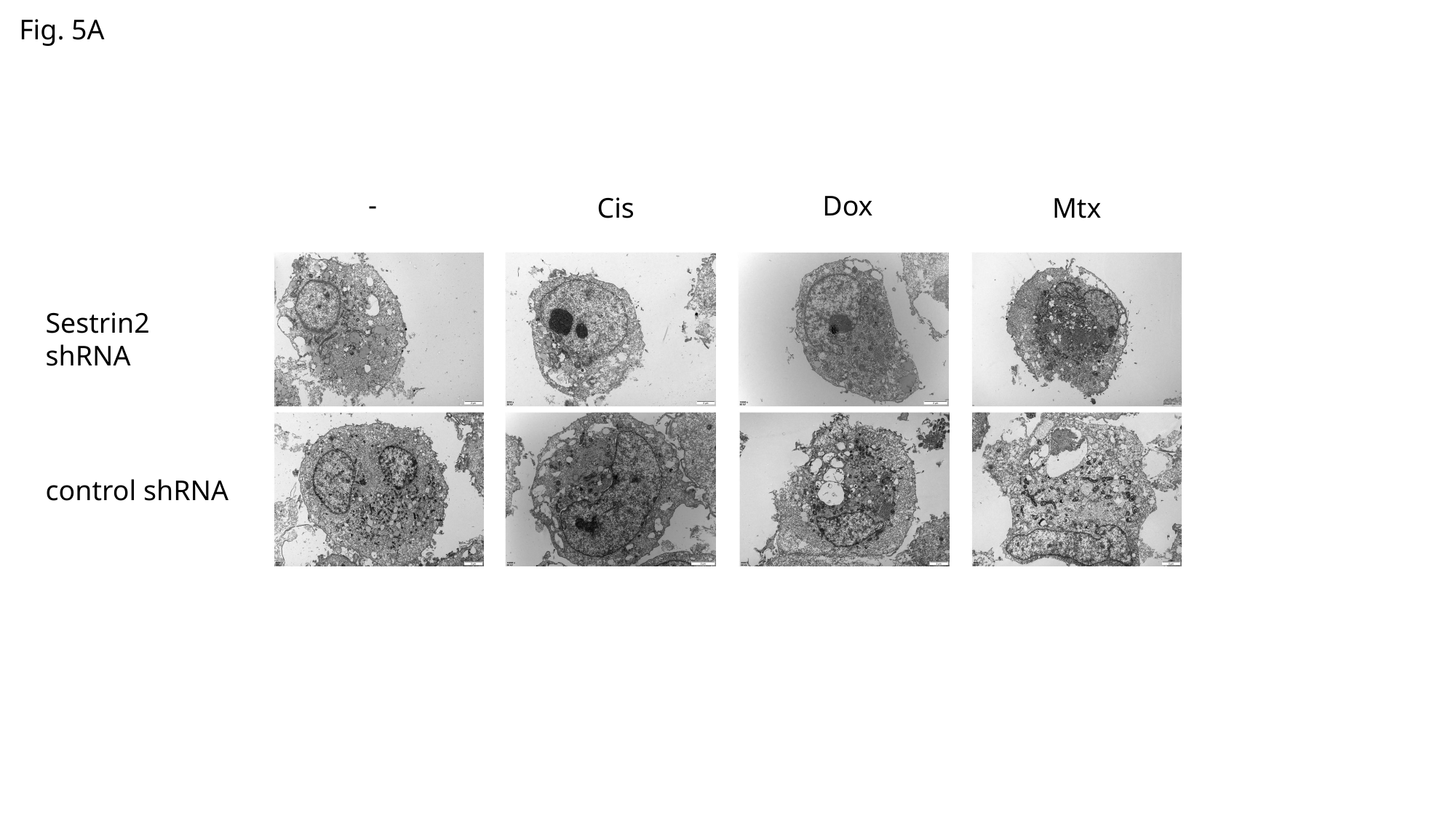

Fig. 5A
-
Dox
Cis
Mtx
Sestrin2 shRNA
control shRNA
