# Supplementary material for: Sestrin2-Mediated Autophagy Contributes to Drug Resistance via Endoplasmic Reticulum Stress in Human Osteosarcoma
Source: Front Cell Dev Biol. 2021 Sep 27;9:722960. doi: 10.3389/fcell.2021.722960 (PMC8502982; doi:10.3389/fcell.2021.722960)
Supplement: Supplementary file 11 [file Data_Sheet_12.ZIP › Raw data of tumour volume and weight/Raw data of tumour volume and weight.pptx]

## Slide 1
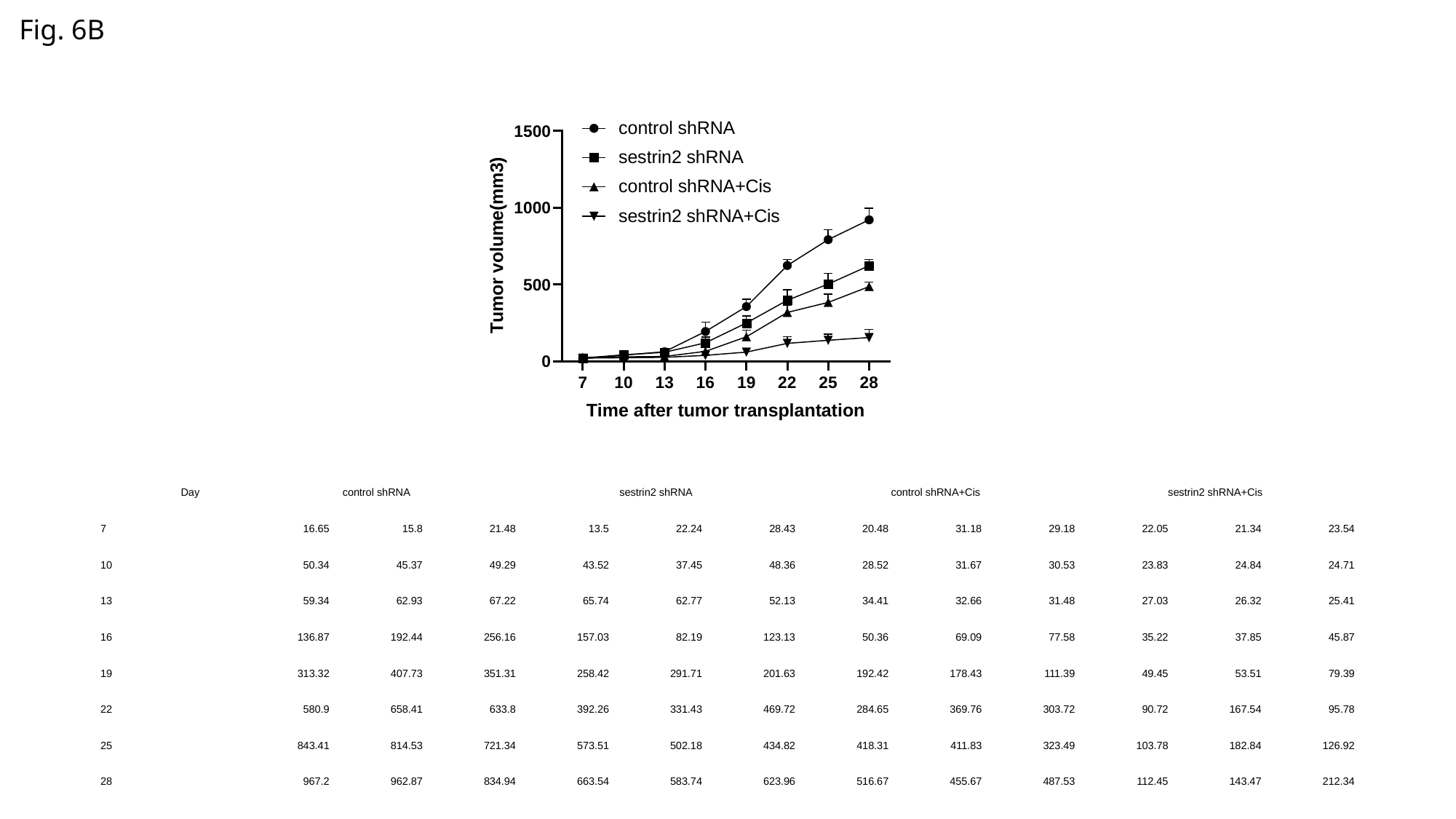

Fig. 6B
| | Day | control shRNA | | | sestrin2 shRNA | | | control shRNA+Cis | | | sestrin2 shRNA+Cis | | |
| --- | --- | --- | --- | --- | --- | --- | --- | --- | --- | --- | --- | --- | --- |
| 7 | | 16.65 | 15.8 | 21.48 | 13.5 | 22.24 | 28.43 | 20.48 | 31.18 | 29.18 | 22.05 | 21.34 | 23.54 |
| 10 | | 50.34 | 45.37 | 49.29 | 43.52 | 37.45 | 48.36 | 28.52 | 31.67 | 30.53 | 23.83 | 24.84 | 24.71 |
| 13 | | 59.34 | 62.93 | 67.22 | 65.74 | 62.77 | 52.13 | 34.41 | 32.66 | 31.48 | 27.03 | 26.32 | 25.41 |
| 16 | | 136.87 | 192.44 | 256.16 | 157.03 | 82.19 | 123.13 | 50.36 | 69.09 | 77.58 | 35.22 | 37.85 | 45.87 |
| 19 | | 313.32 | 407.73 | 351.31 | 258.42 | 291.71 | 201.63 | 192.42 | 178.43 | 111.39 | 49.45 | 53.51 | 79.39 |
| 22 | | 580.9 | 658.41 | 633.8 | 392.26 | 331.43 | 469.72 | 284.65 | 369.76 | 303.72 | 90.72 | 167.54 | 95.78 |
| 25 | | 843.41 | 814.53 | 721.34 | 573.51 | 502.18 | 434.82 | 418.31 | 411.83 | 323.49 | 103.78 | 182.84 | 126.92 |
| 28 | | 967.2 | 962.87 | 834.94 | 663.54 | 583.74 | 623.96 | 516.67 | 455.67 | 487.53 | 112.45 | 143.47 | 212.34 |

## Slide 2
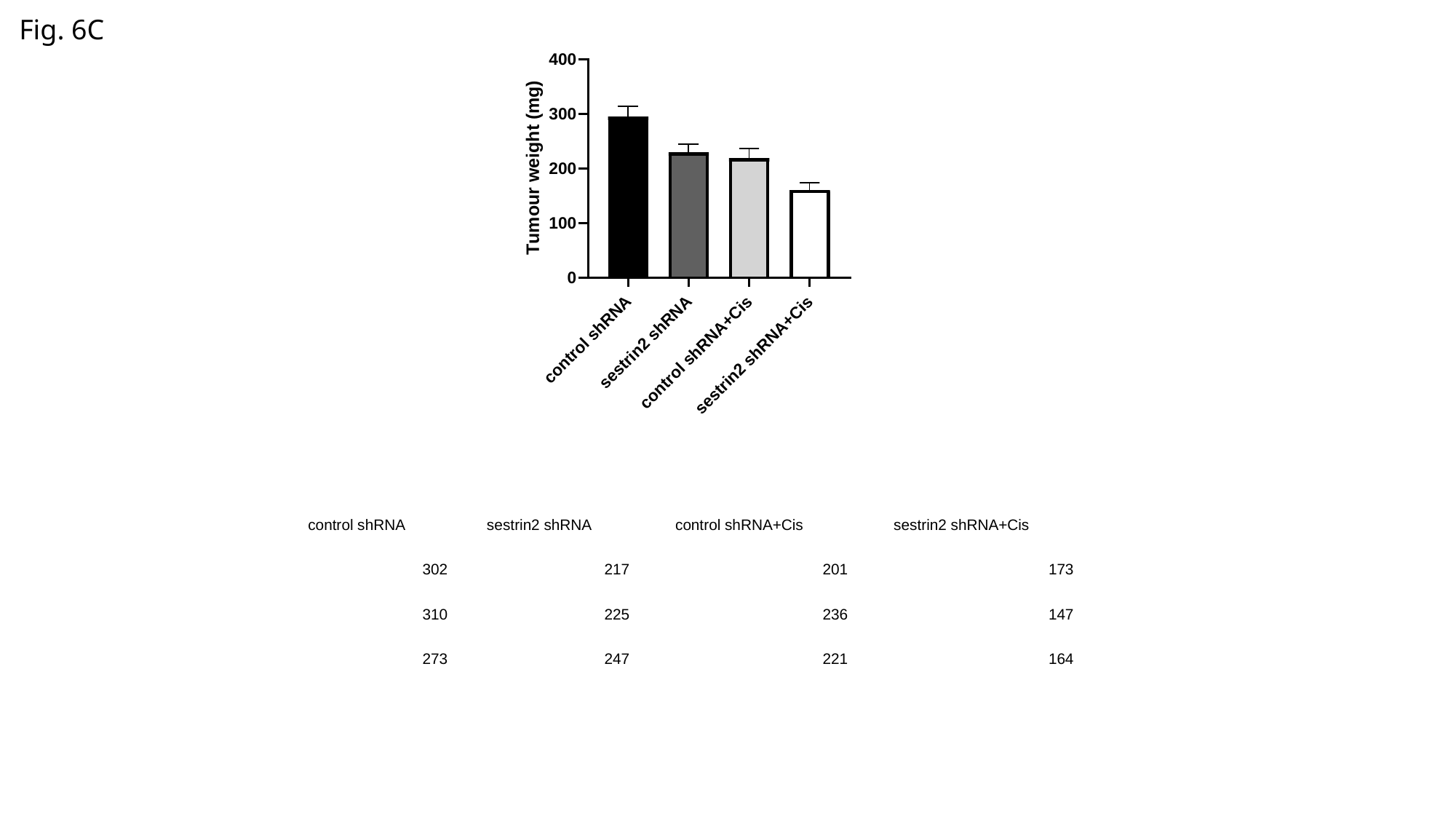

Fig. 6C
| control shRNA | sestrin2 shRNA | control shRNA+Cis | sestrin2 shRNA+Cis |
| --- | --- | --- | --- |
| 302 | 217 | 201 | 173 |
| 310 | 225 | 236 | 147 |
| 273 | 247 | 221 | 164 |
