# Supplementary material for: Sestrin2-Mediated Autophagy Contributes to Drug Resistance via Endoplasmic Reticulum Stress in Human Osteosarcoma
Source: Front Cell Dev Biol. 2021 Sep 27;9:722960. doi: 10.3389/fcell.2021.722960 (PMC8502982; doi:10.3389/fcell.2021.722960)
Supplement: Supplementary file 12 [file Data_Sheet_13.ZIP › Raw data of western blot-1/Raw data of western blot-1.pptx]

## Slide 1
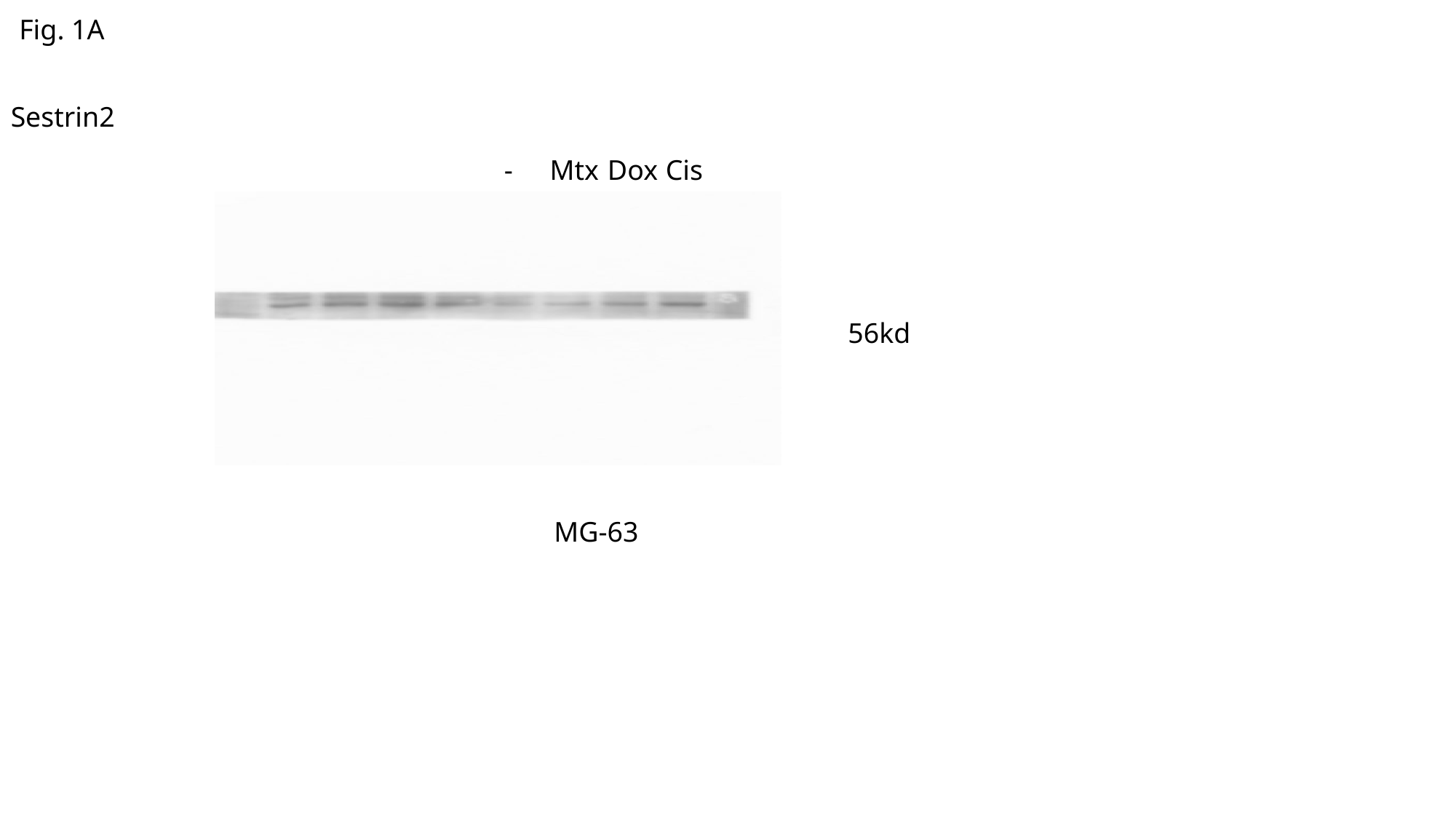

Fig. 1A
Sestrin2
-
Mtx
Dox
Cis
56kd
MG-63

## Slide 2
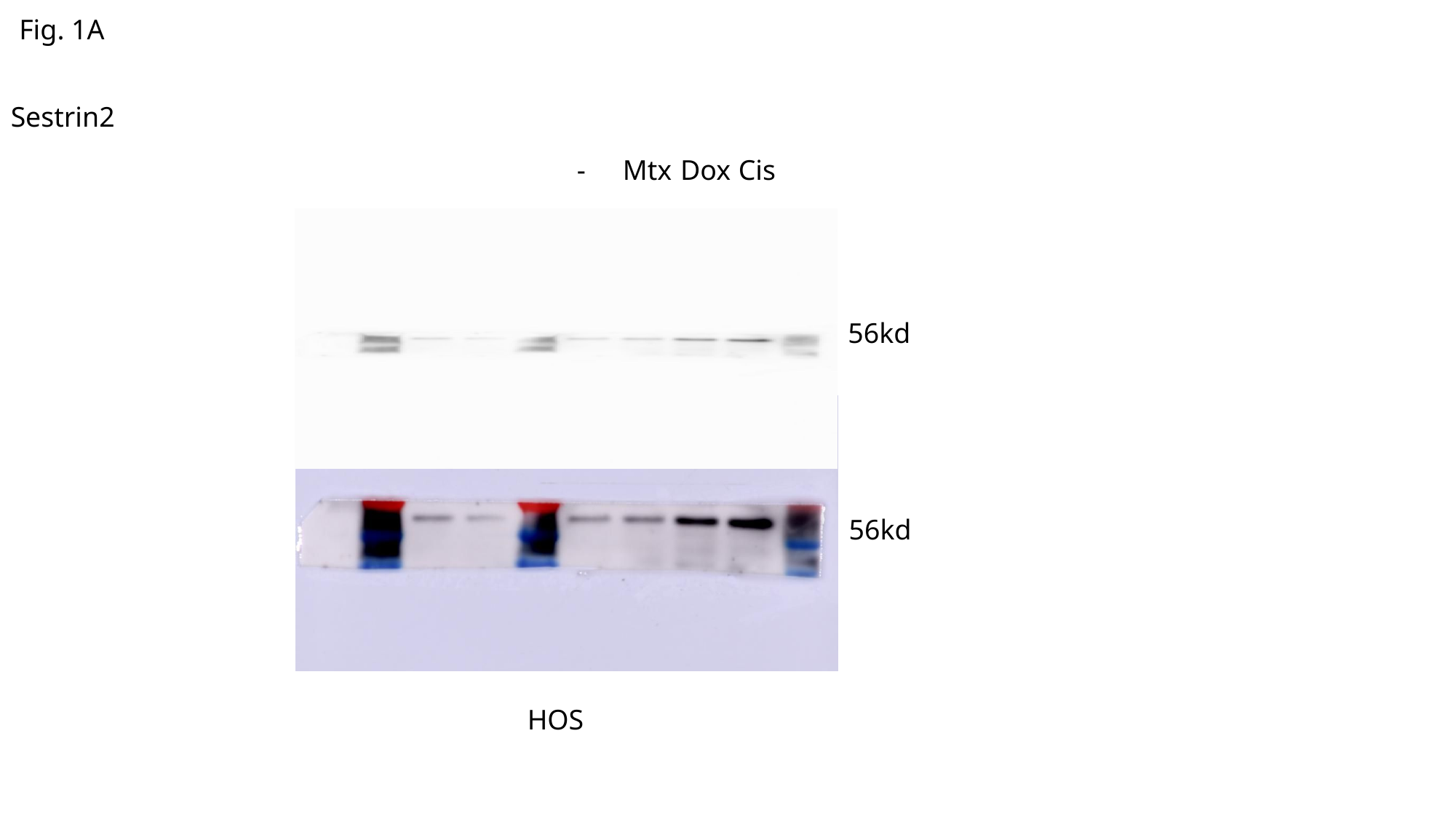

Fig. 1A
Sestrin2
-
Mtx
Dox
Cis
56kd
56kd
HOS

## Slide 3
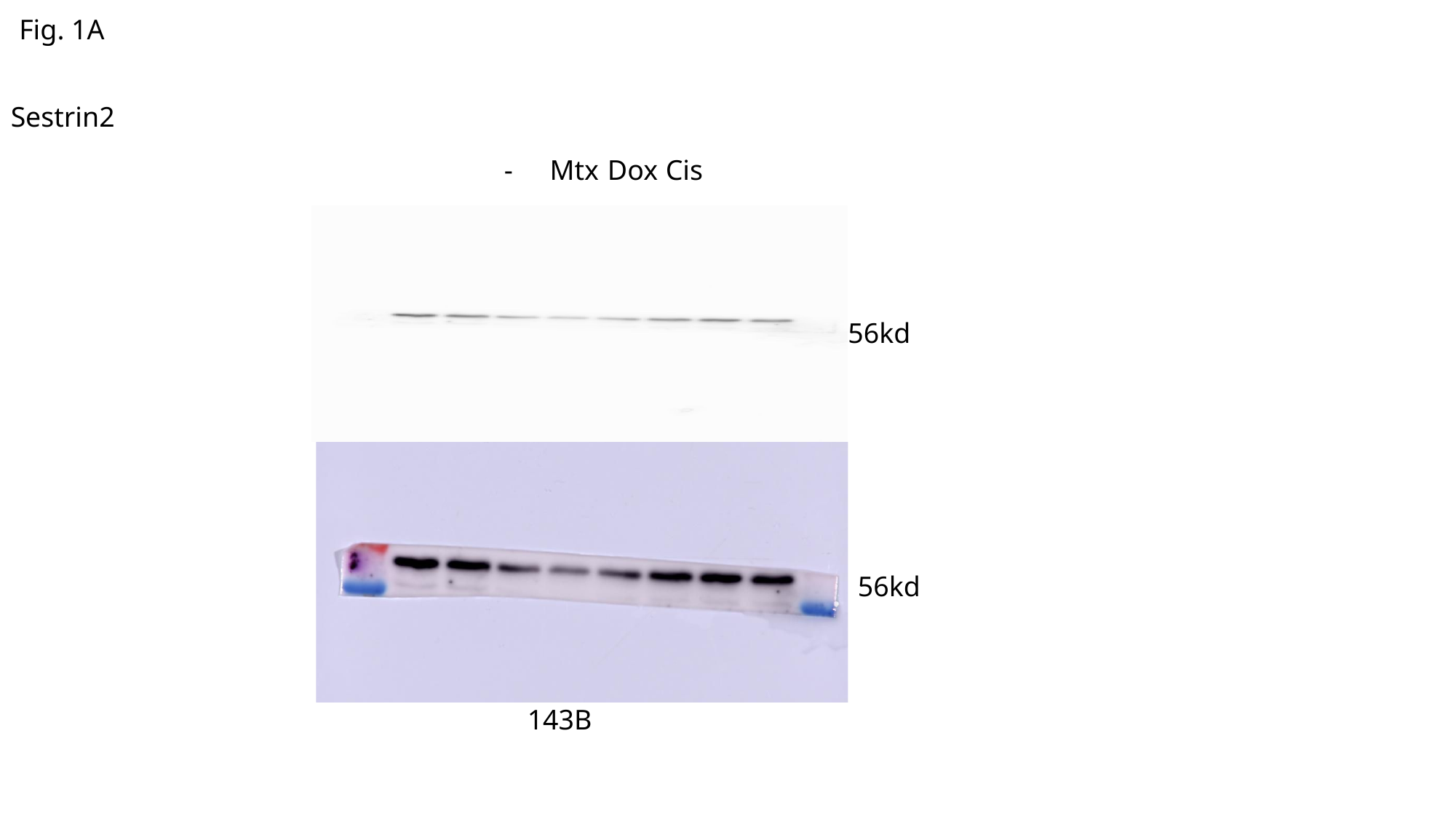

Fig. 1A
Sestrin2
-
Mtx
Dox
Cis
56kd
56kd
143B

## Slide 4
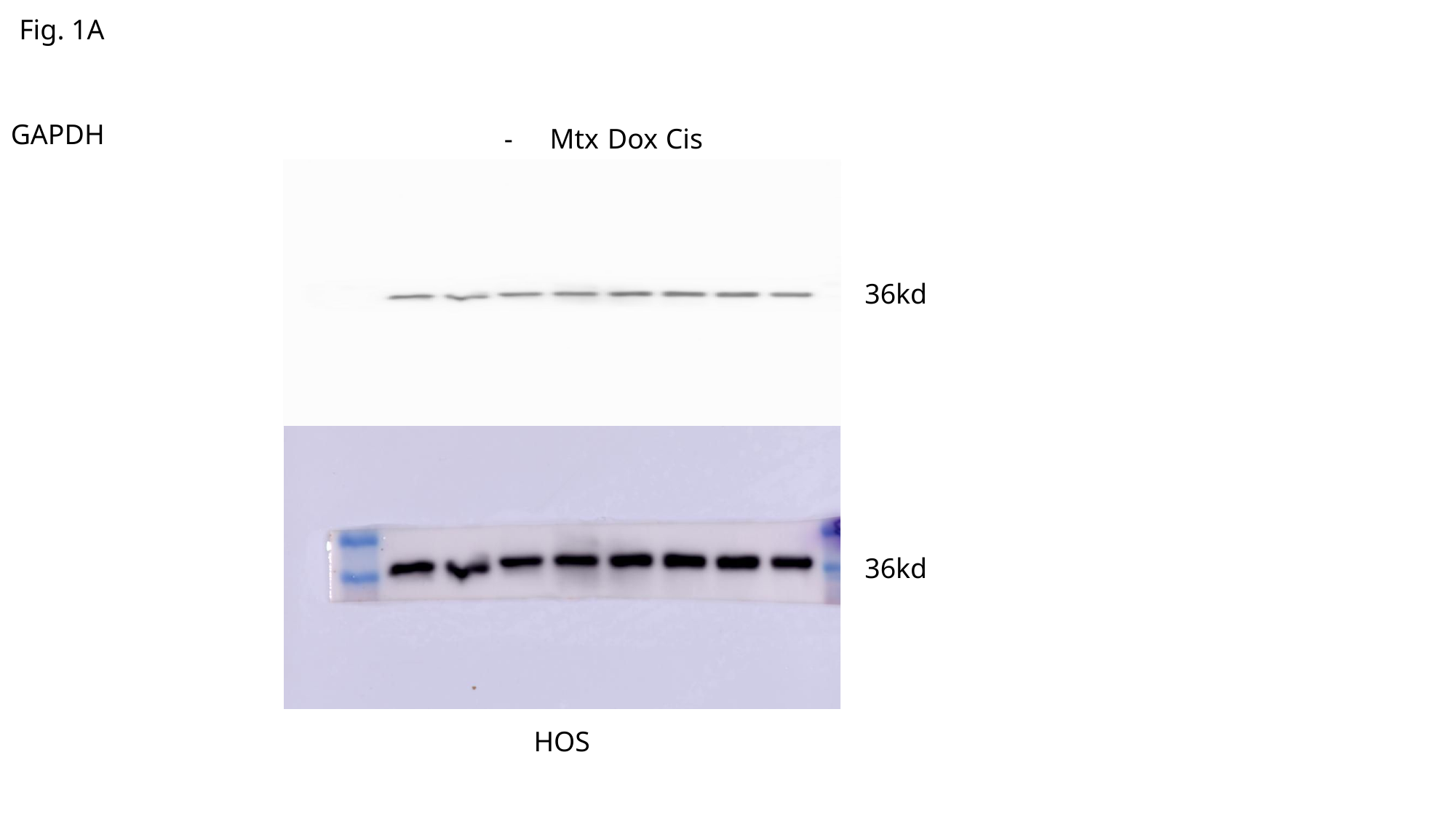

Fig. 1A
GAPDH
-
Mtx
Dox
Cis
36kd
36kd
HOS

## Slide 5
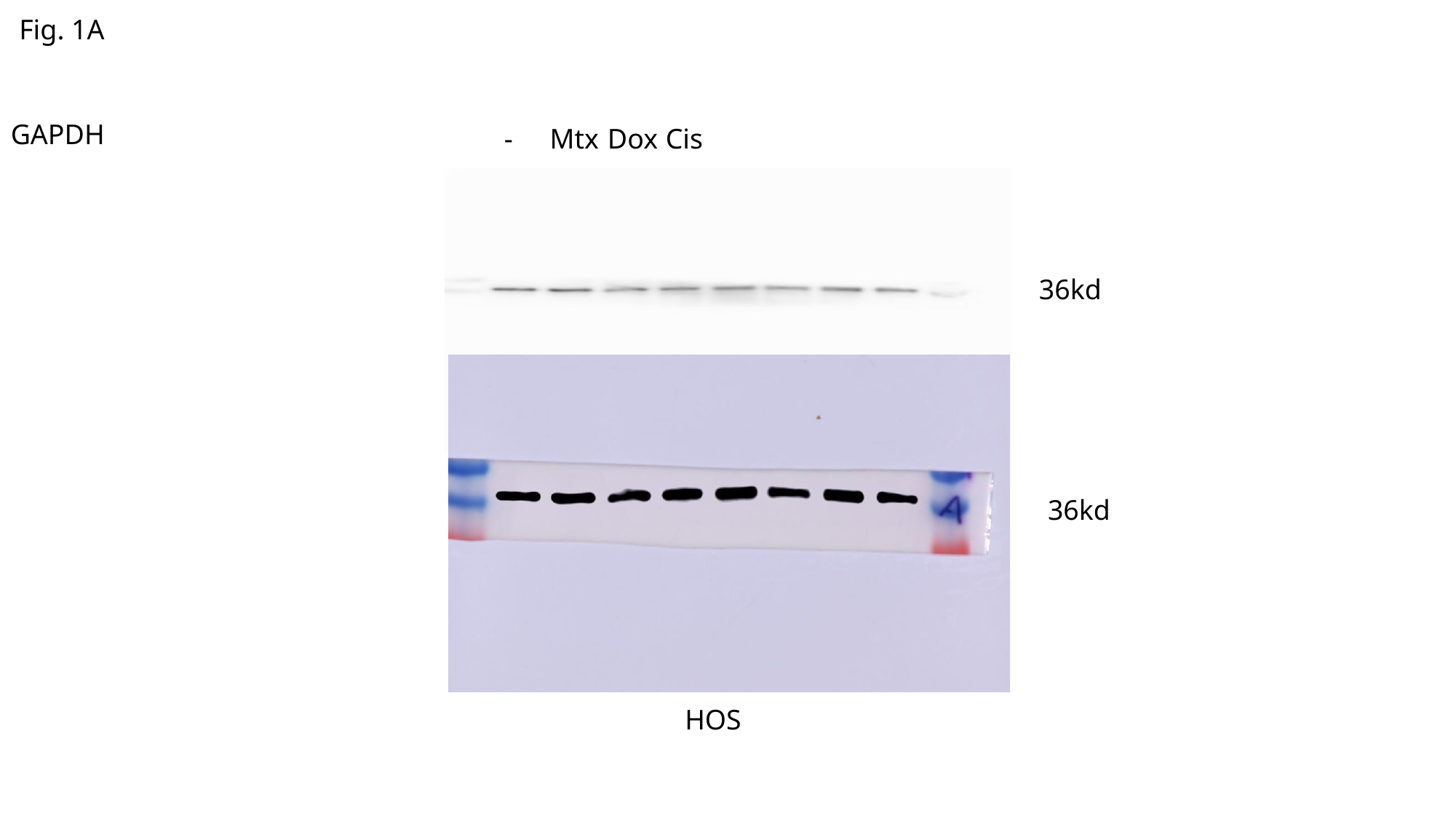

Fig. 1A
GAPDH
-
Mtx
Dox
Cis
36kd
36kd
HOS

## Slide 6
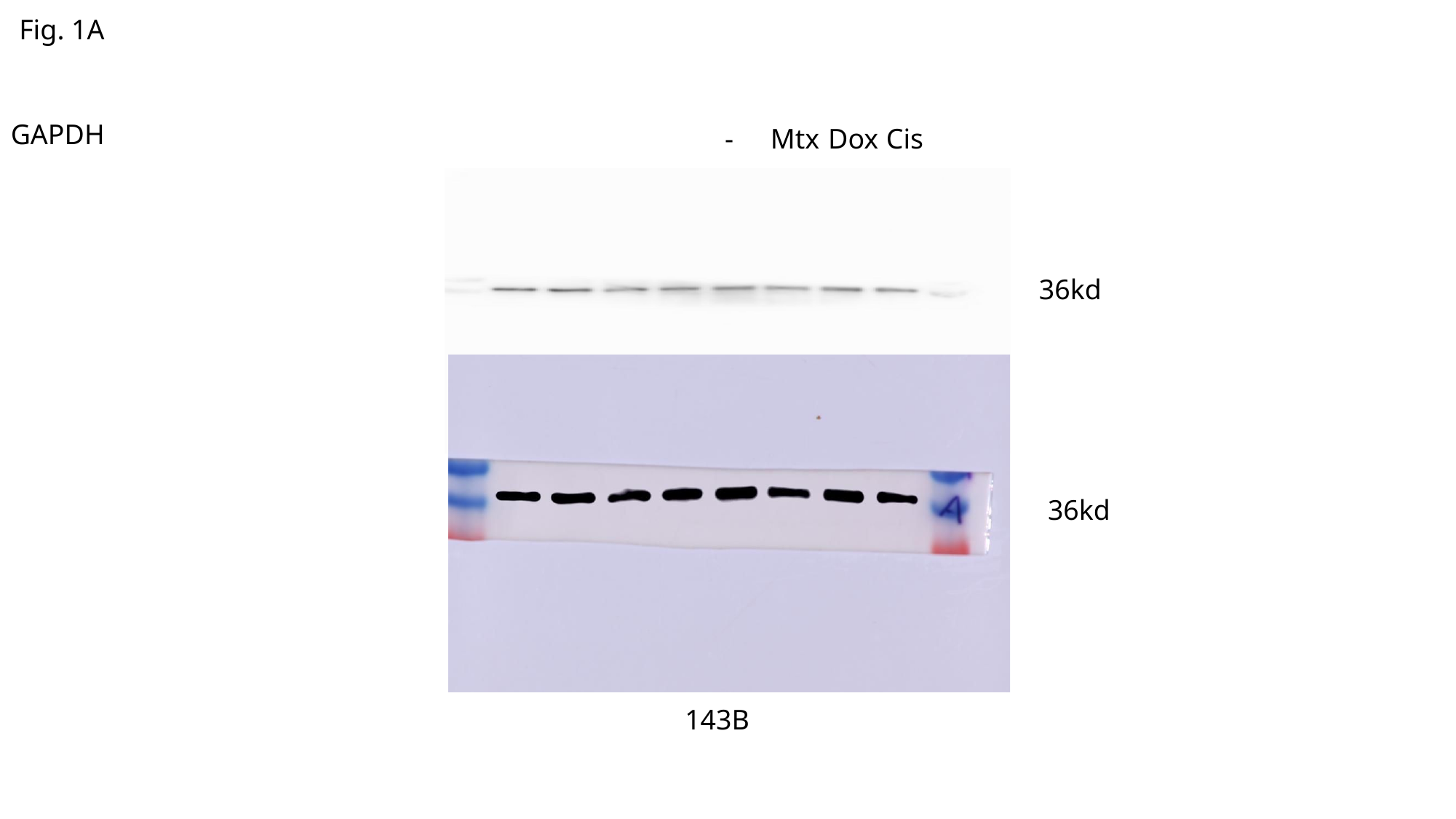

Fig. 1A
GAPDH
-
Mtx
Dox
Cis
36kd
36kd
143B

## Slide 7
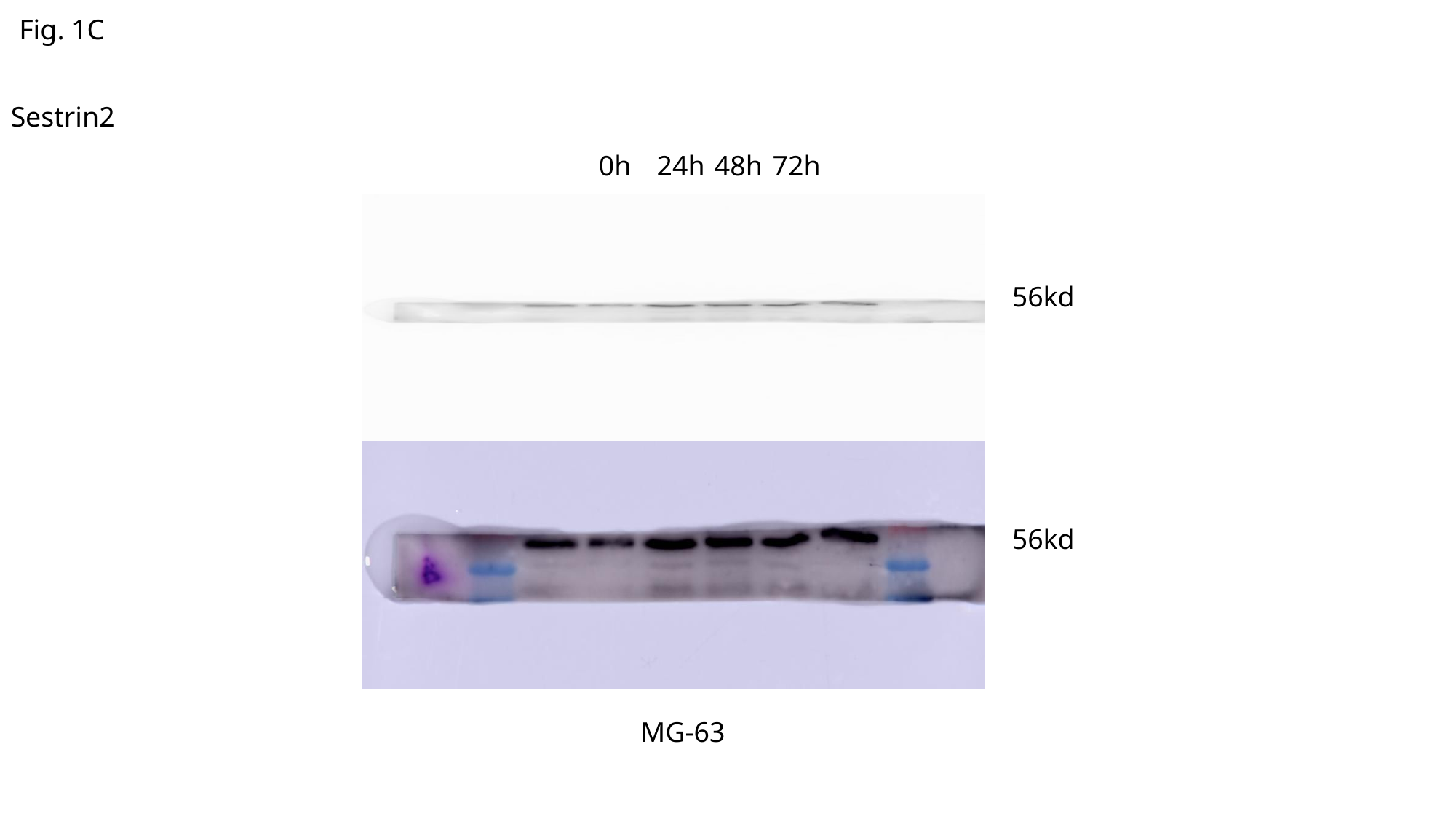

Fig. 1C
Sestrin2
0h
24h
48h
72h
56kd
56kd
MG-63

## Slide 8
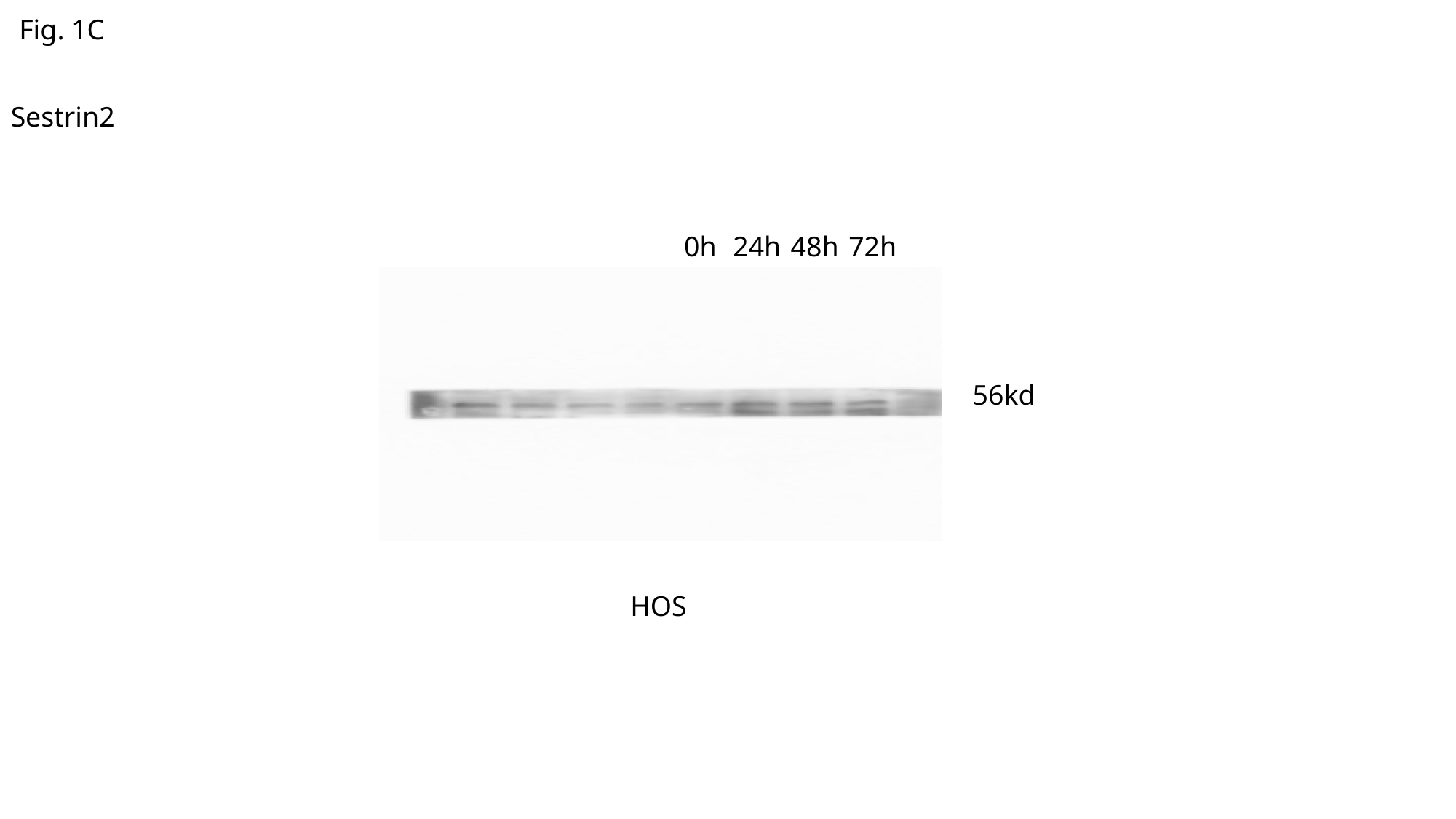

Fig. 1C
Sestrin2
0h
24h
48h
72h
56kd
HOS

## Slide 9
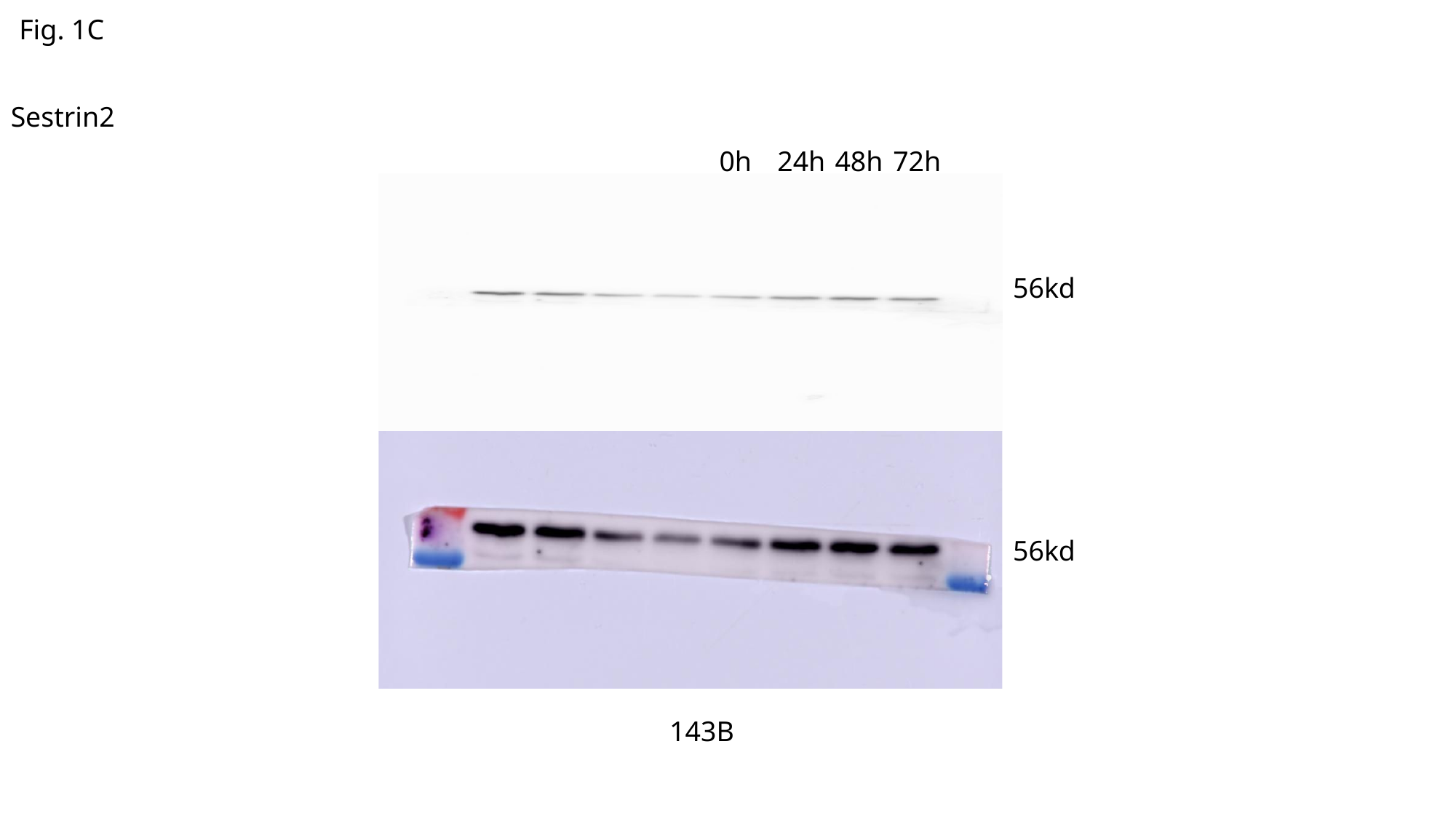

Fig. 1C
Sestrin2
0h
24h
48h
72h
56kd
56kd
143B

## Slide 10
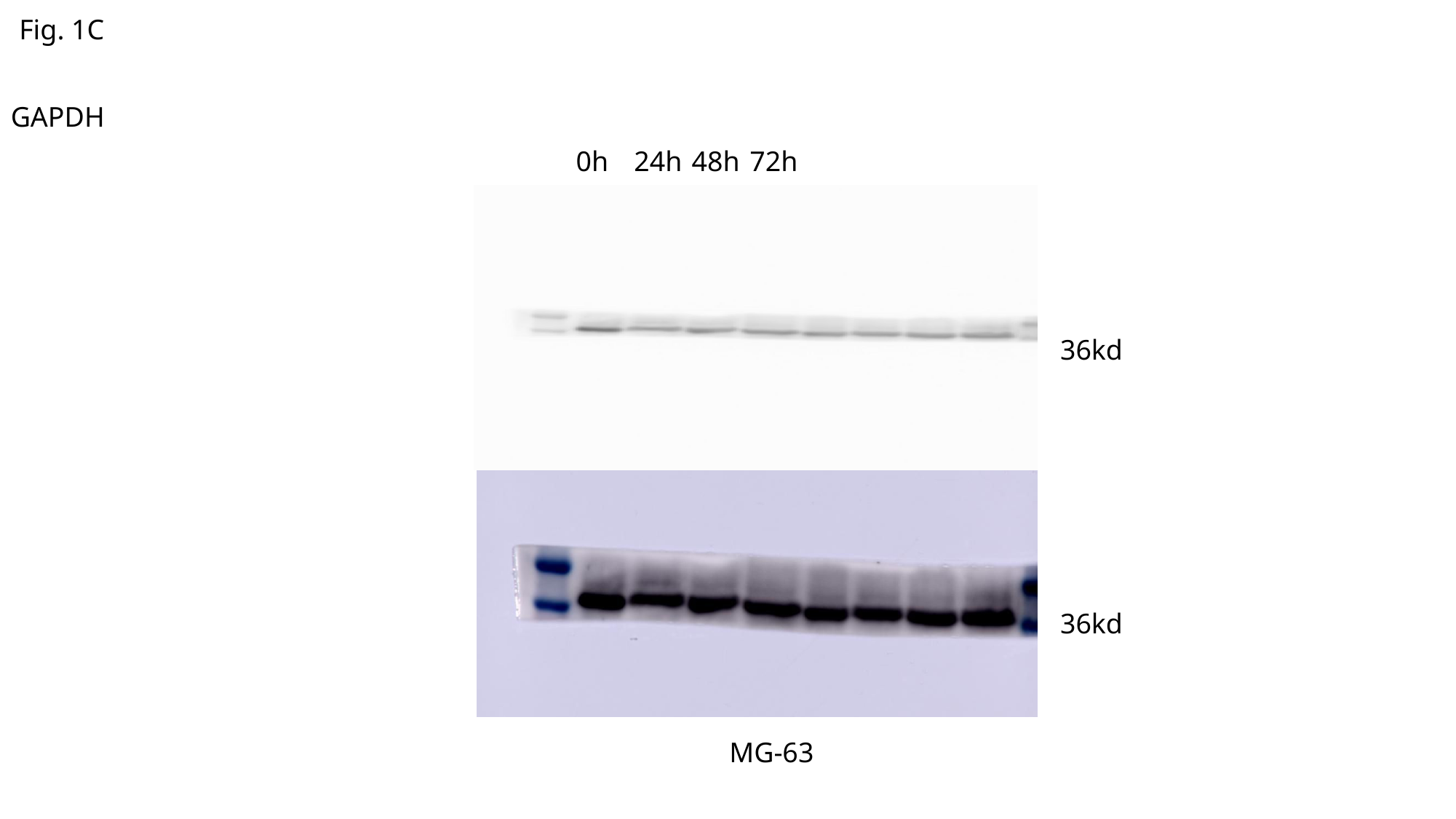

Fig. 1C
GAPDH
0h
24h
48h
72h
36kd
36kd
MG-63

## Slide 11
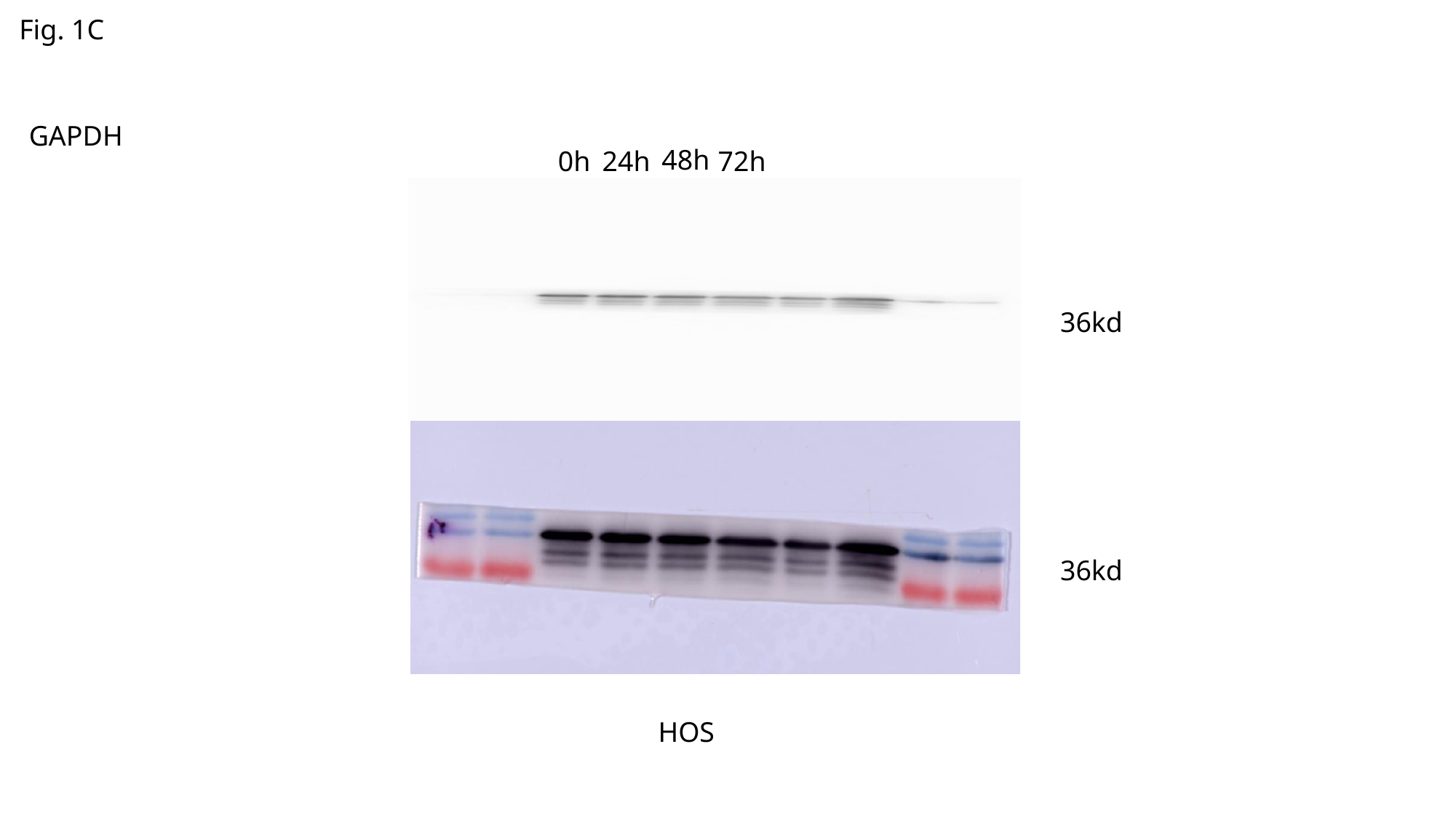

Fig. 1C
GAPDH
48h
0h
24h
72h
36kd
36kd
HOS

## Slide 12
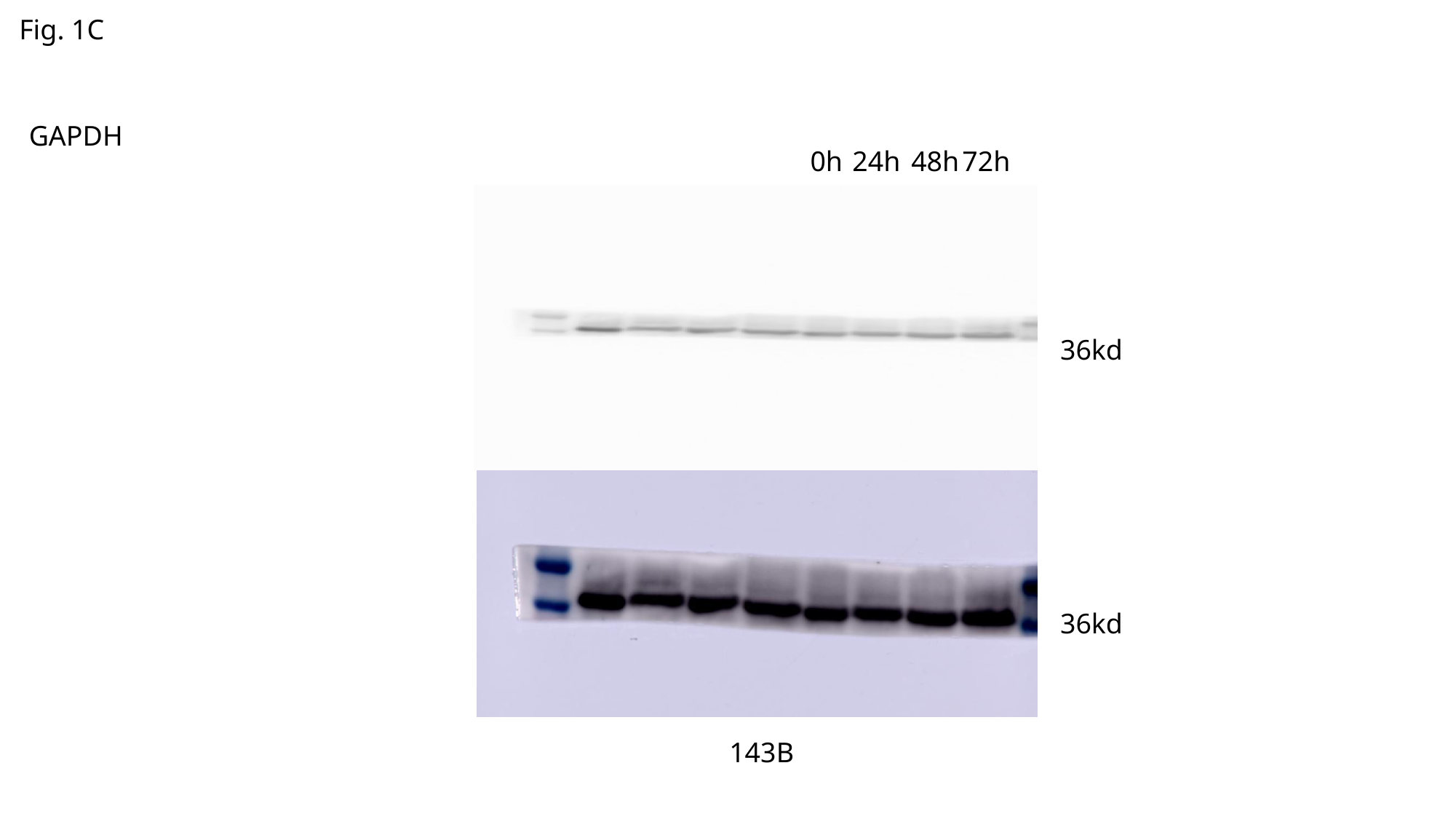

Fig. 1C
GAPDH
0h
24h
48h
72h
36kd
36kd
143B

## Slide 13
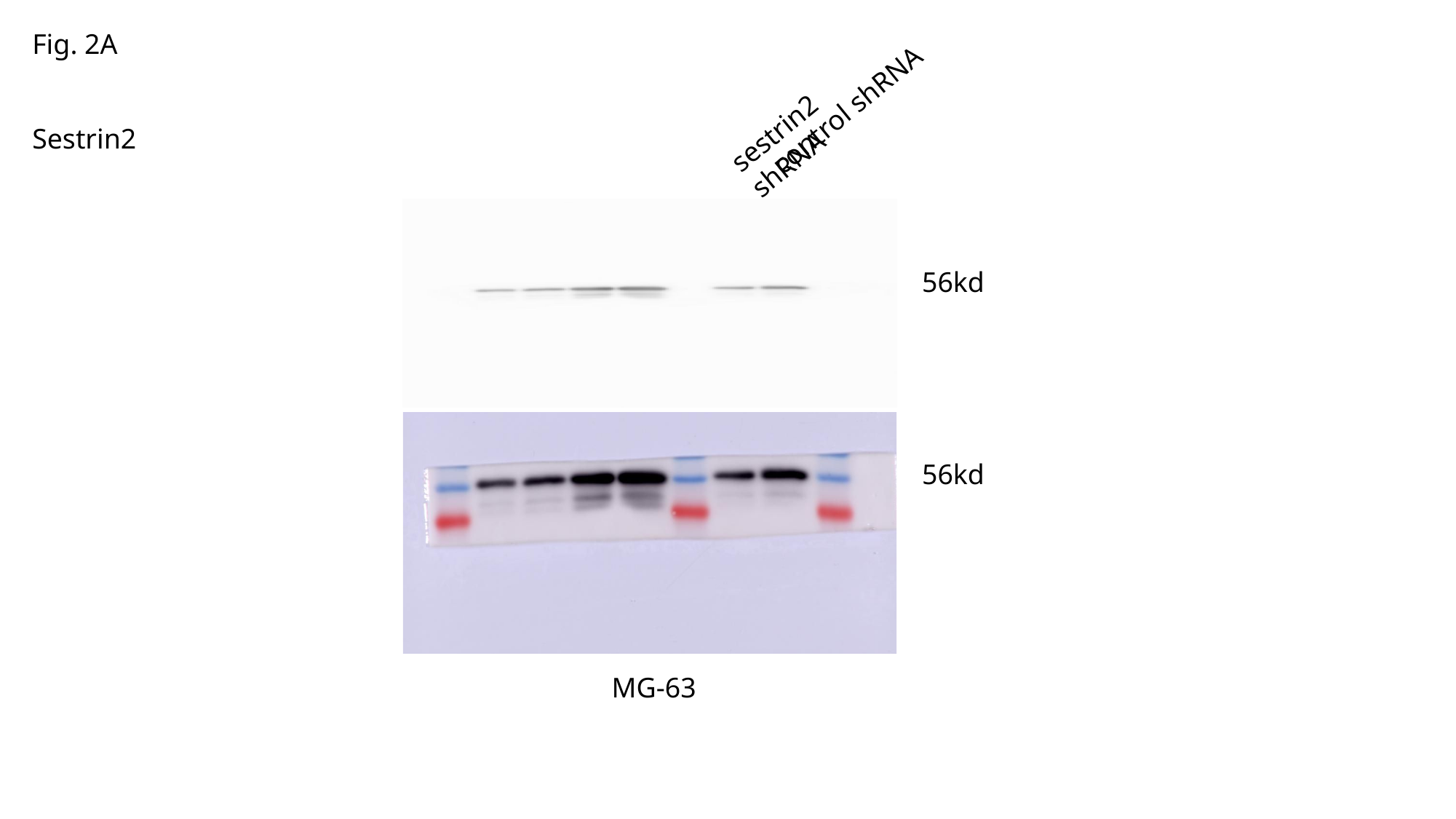

Fig. 2A
sestrin2 shRNA
control shRNA
Sestrin2
56kd
56kd
MG-63

## Slide 14
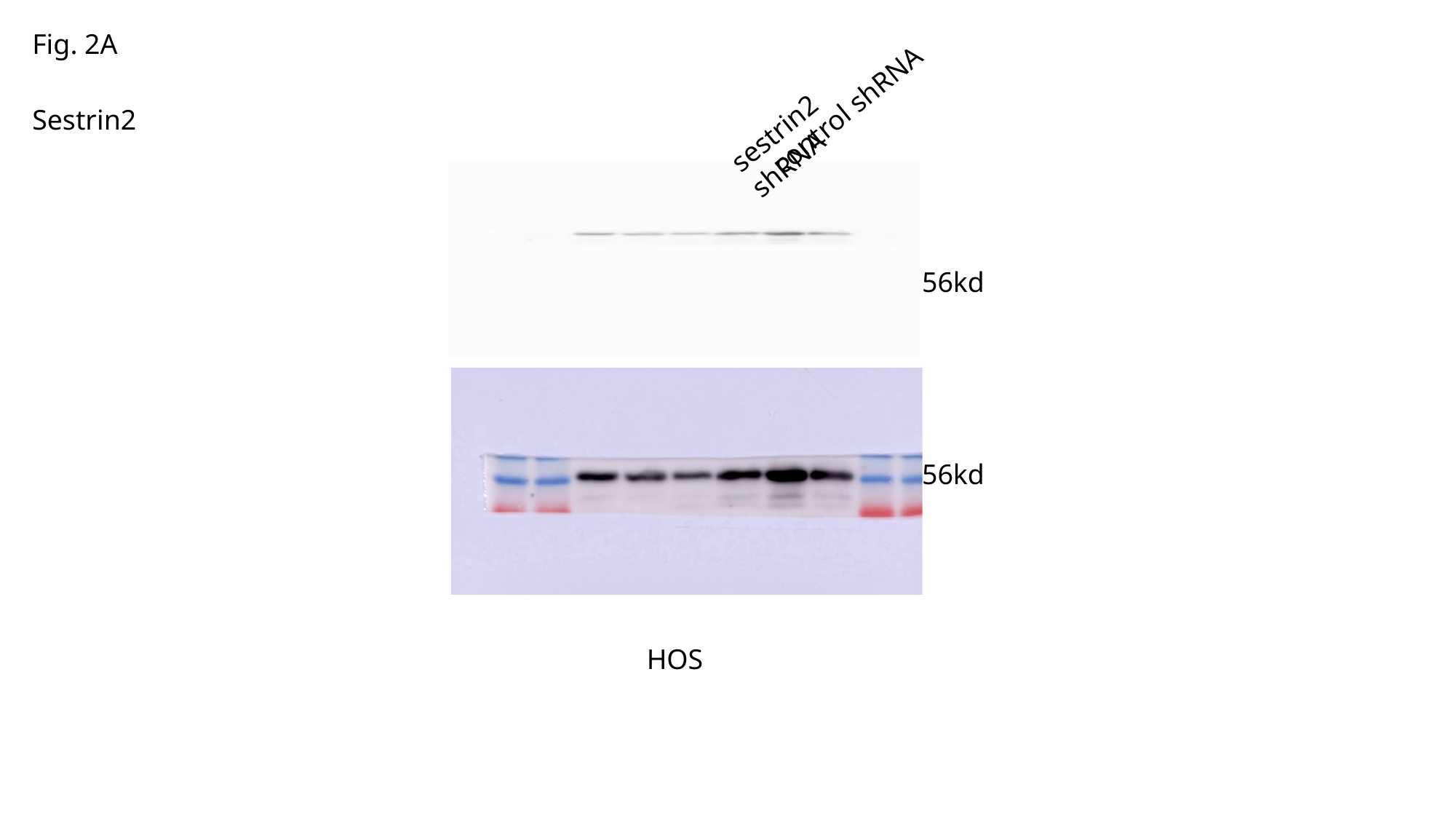

Fig. 2A
sestrin2 shRNA
control shRNA
Sestrin2
56kd
56kd
HOS

## Slide 15
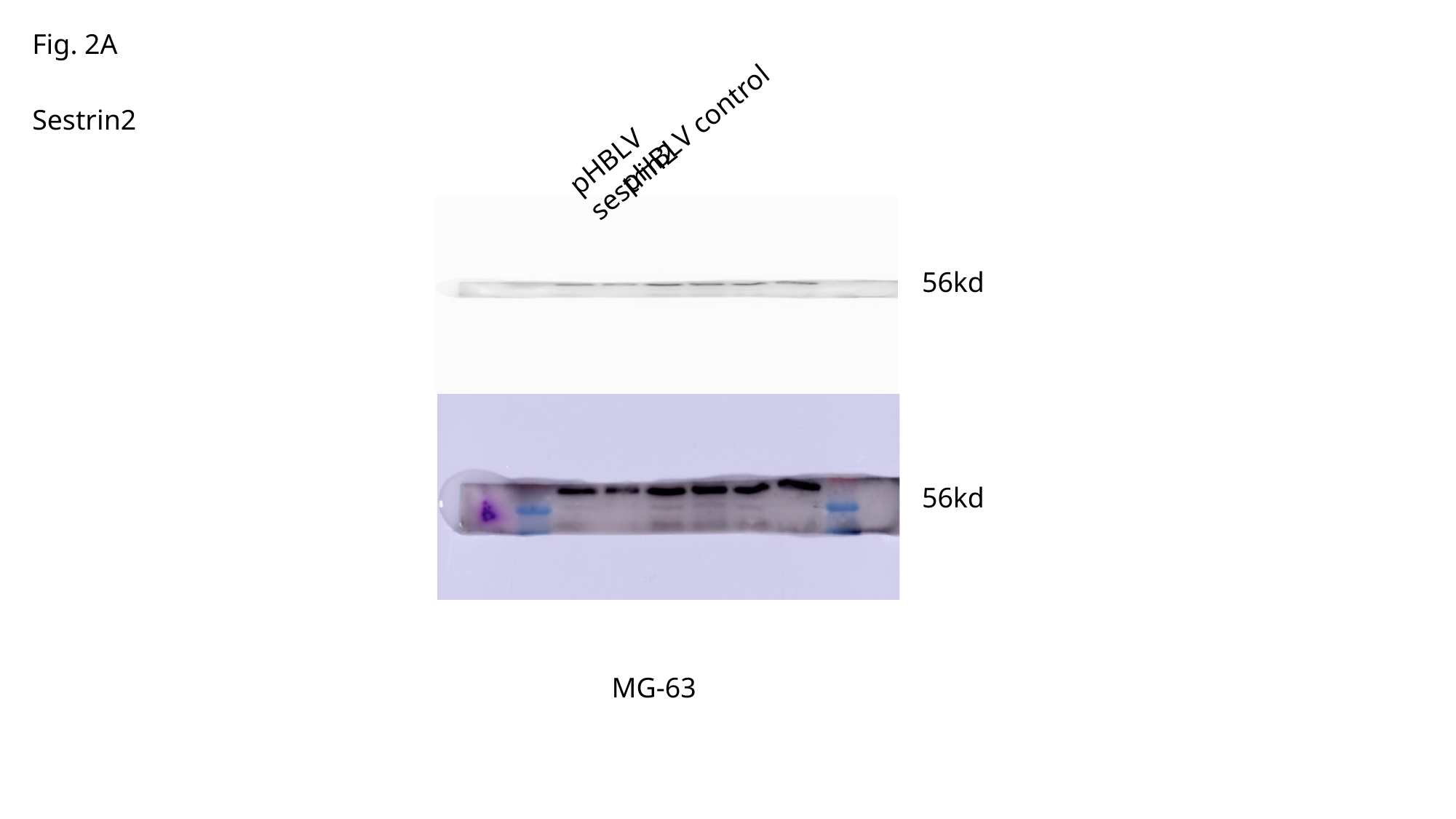

Fig. 2A
Sestrin2
pHBLV control
pHBLV sestrin2
56kd
56kd
MG-63

## Slide 16
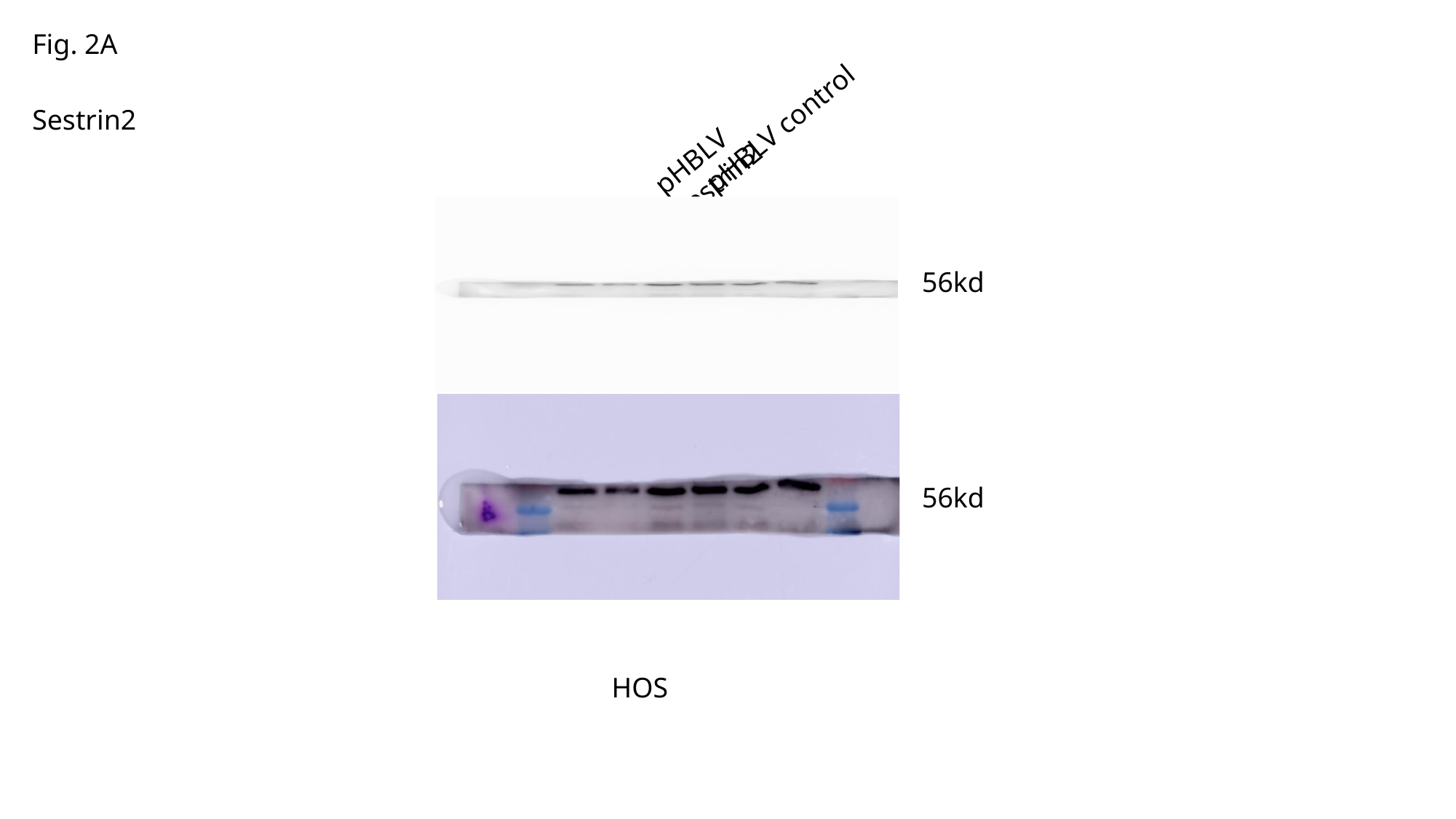

Fig. 2A
Sestrin2
pHBLV control
pHBLV sestrin2
56kd
56kd
HOS

## Slide 17
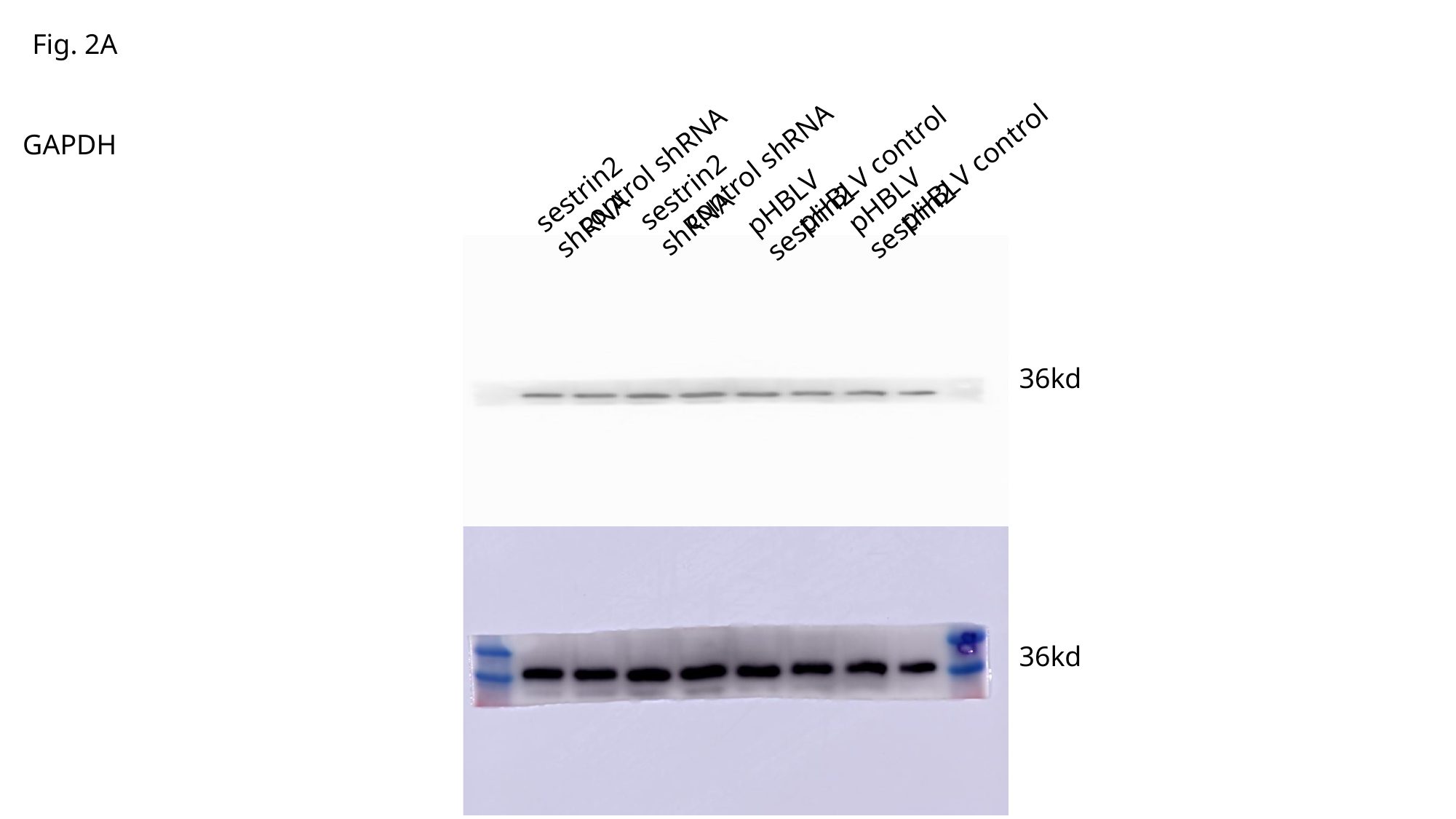

Fig. 2A
GAPDH
sestrin2 shRNA
control shRNA
pHBLV control
sestrin2 shRNA
pHBLV sestrin2
pHBLV control
control shRNA
pHBLV sestrin2
36kd
36kd

## Slide 18
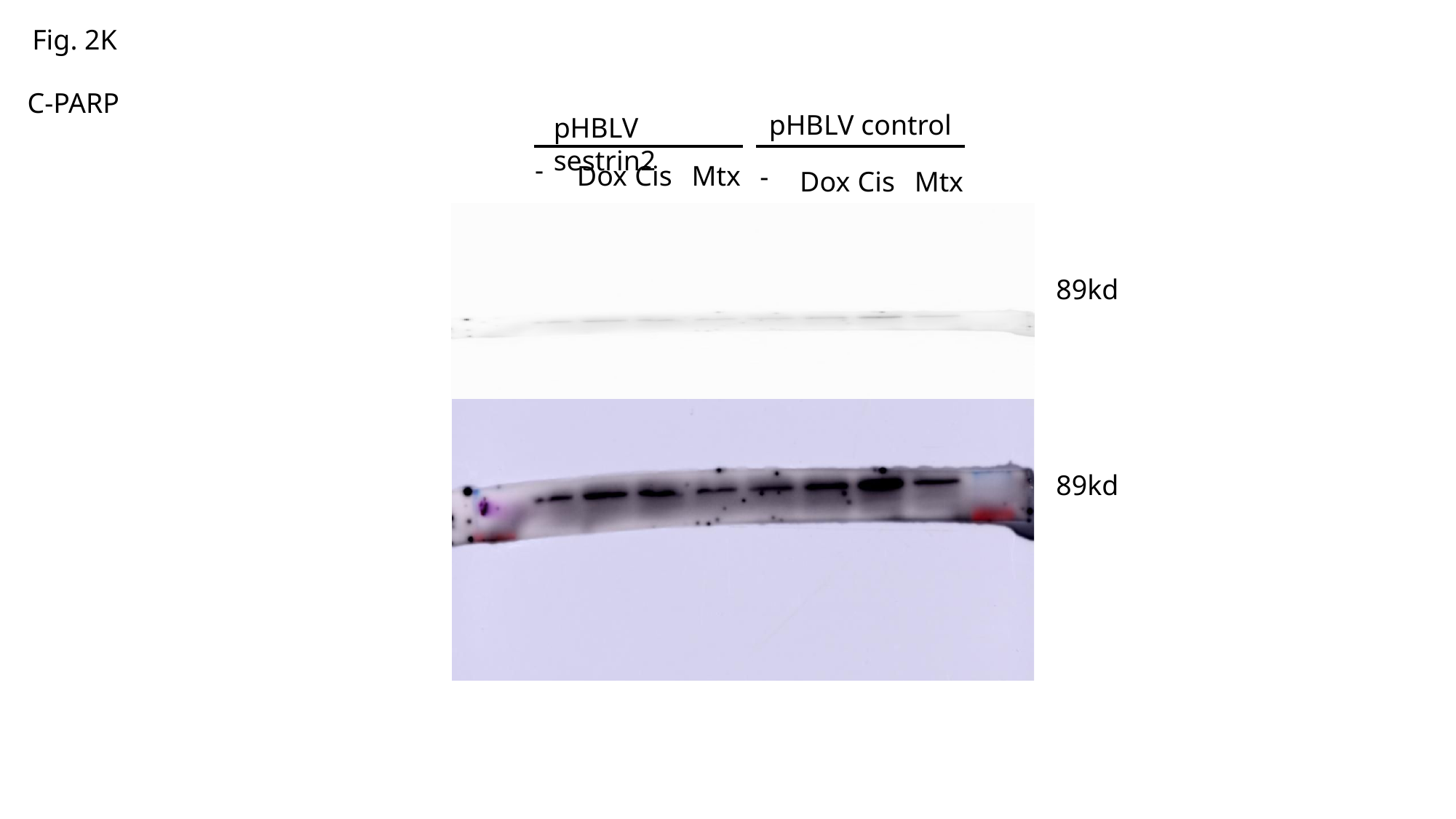

Fig. 2K
C-PARP
pHBLV control
pHBLV sestrin2
-
Dox
Cis
Mtx
-
Dox
Cis
Mtx
89kd
89kd

## Slide 19
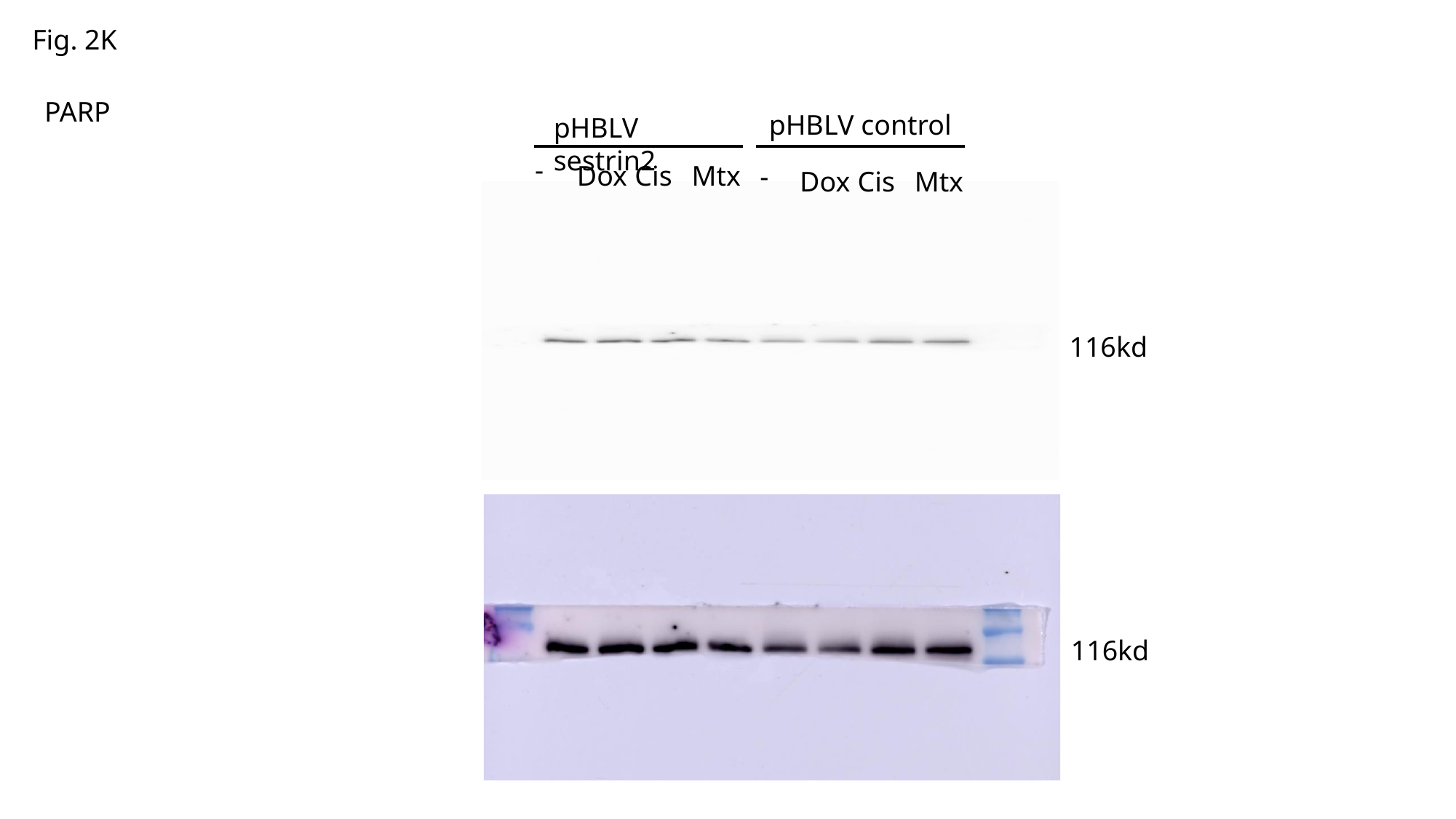

Fig. 2K
PARP
pHBLV control
pHBLV sestrin2
-
Dox
Cis
Mtx
-
Dox
Cis
Mtx
116kd
116kd

## Slide 20
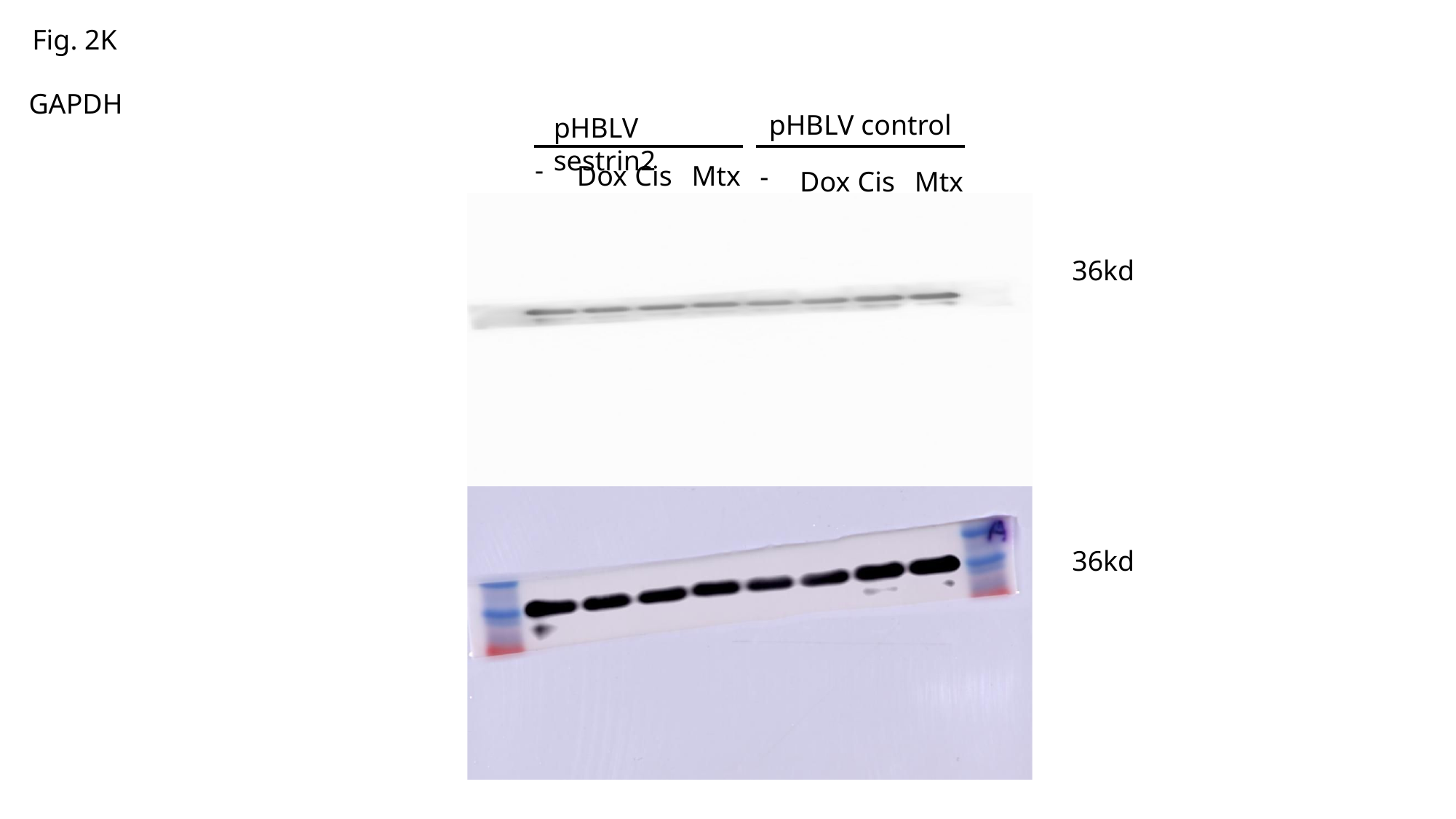

Fig. 2K
GAPDH
pHBLV control
pHBLV sestrin2
-
Dox
Cis
Mtx
-
Dox
Cis
Mtx
36kd
36kd

## Slide 21
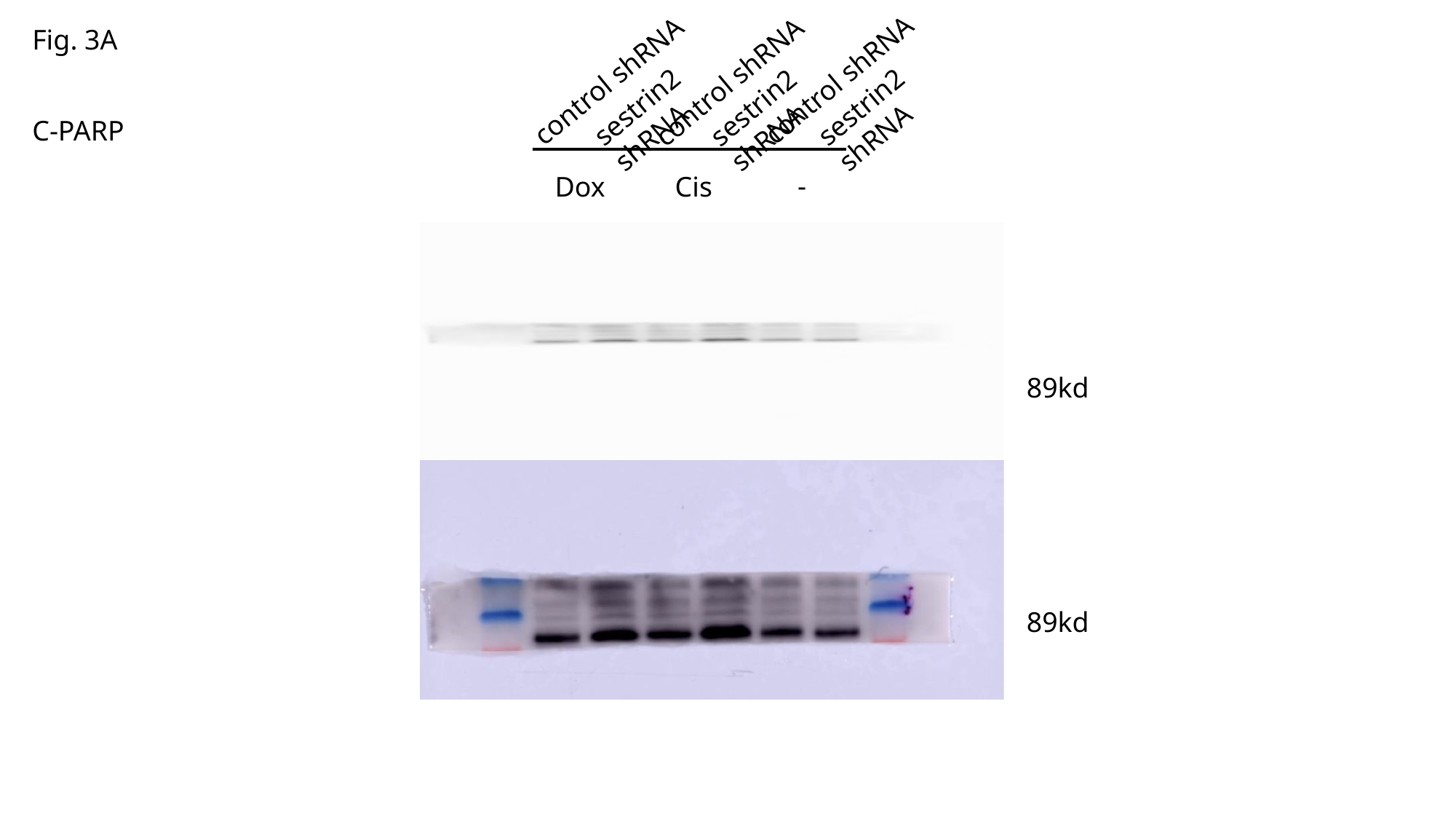

Fig. 3A
control shRNA
sestrin2 shRNA
control shRNA
sestrin2 shRNA
sestrin2 shRNA
control shRNA
C-PARP
-
Dox
Cis
89kd
89kd

## Slide 22
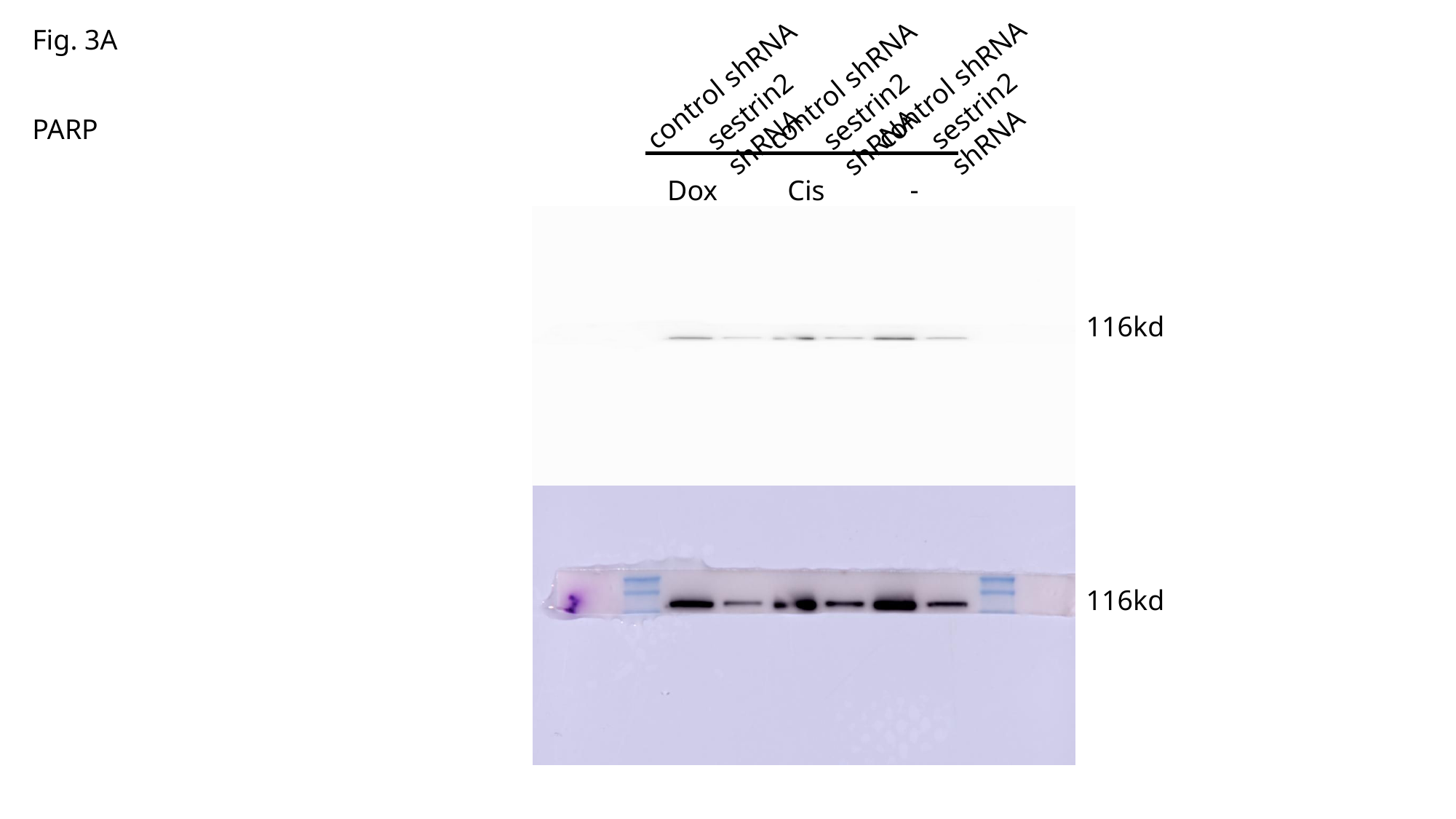

Fig. 3A
control shRNA
sestrin2 shRNA
control shRNA
sestrin2 shRNA
sestrin2 shRNA
control shRNA
PARP
-
Dox
Cis
116kd
116kd

## Slide 23
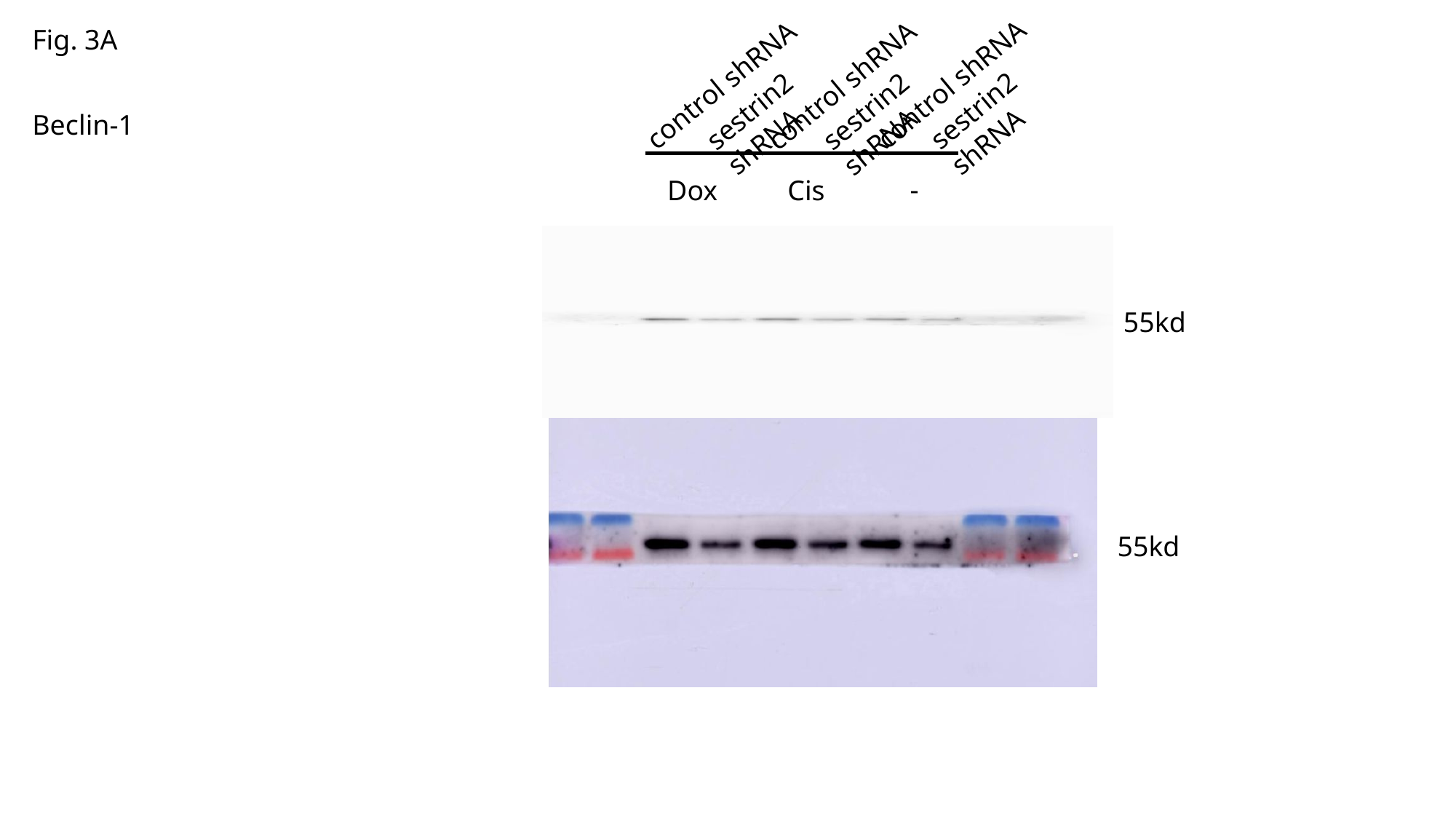

Fig. 3A
control shRNA
sestrin2 shRNA
control shRNA
sestrin2 shRNA
sestrin2 shRNA
control shRNA
Beclin-1
-
Dox
Cis
55kd
55kd

## Slide 24
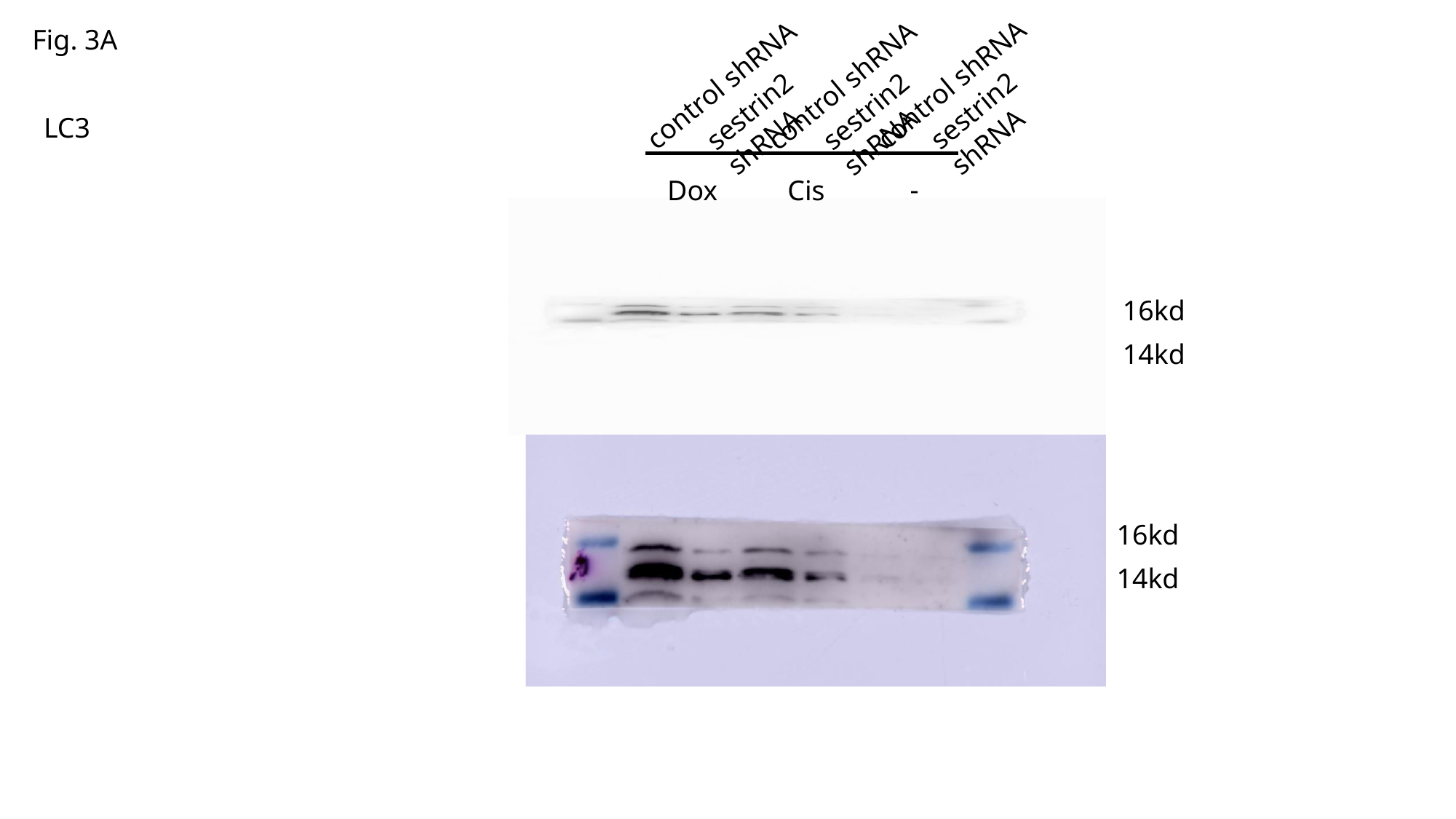

Fig. 3A
control shRNA
sestrin2 shRNA
control shRNA
sestrin2 shRNA
sestrin2 shRNA
control shRNA
LC3
-
Dox
Cis
16kd
14kd
16kd
14kd

## Slide 25
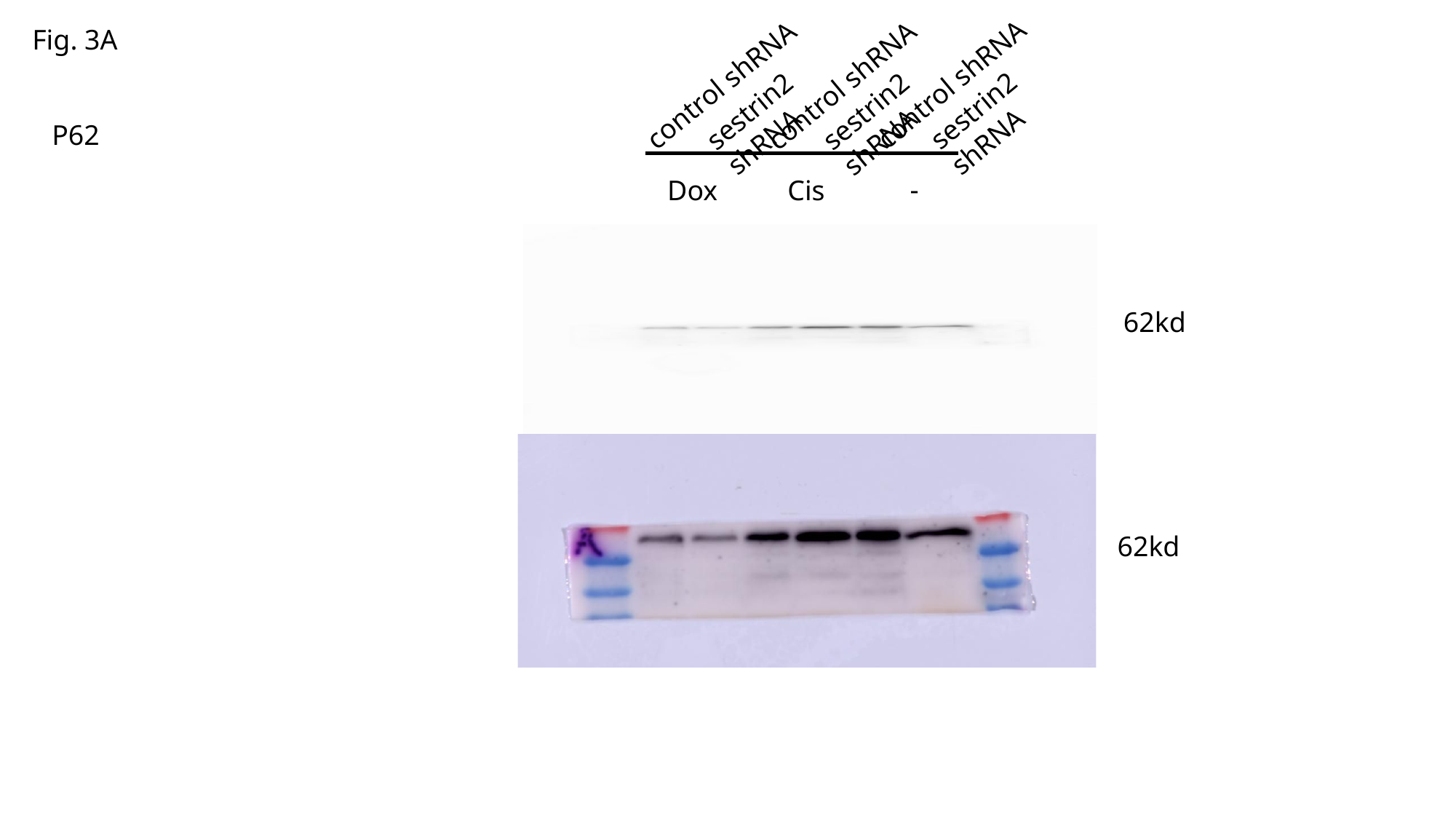

Fig. 3A
control shRNA
sestrin2 shRNA
control shRNA
sestrin2 shRNA
sestrin2 shRNA
control shRNA
P62
-
Dox
Cis
62kd
62kd

## Slide 26
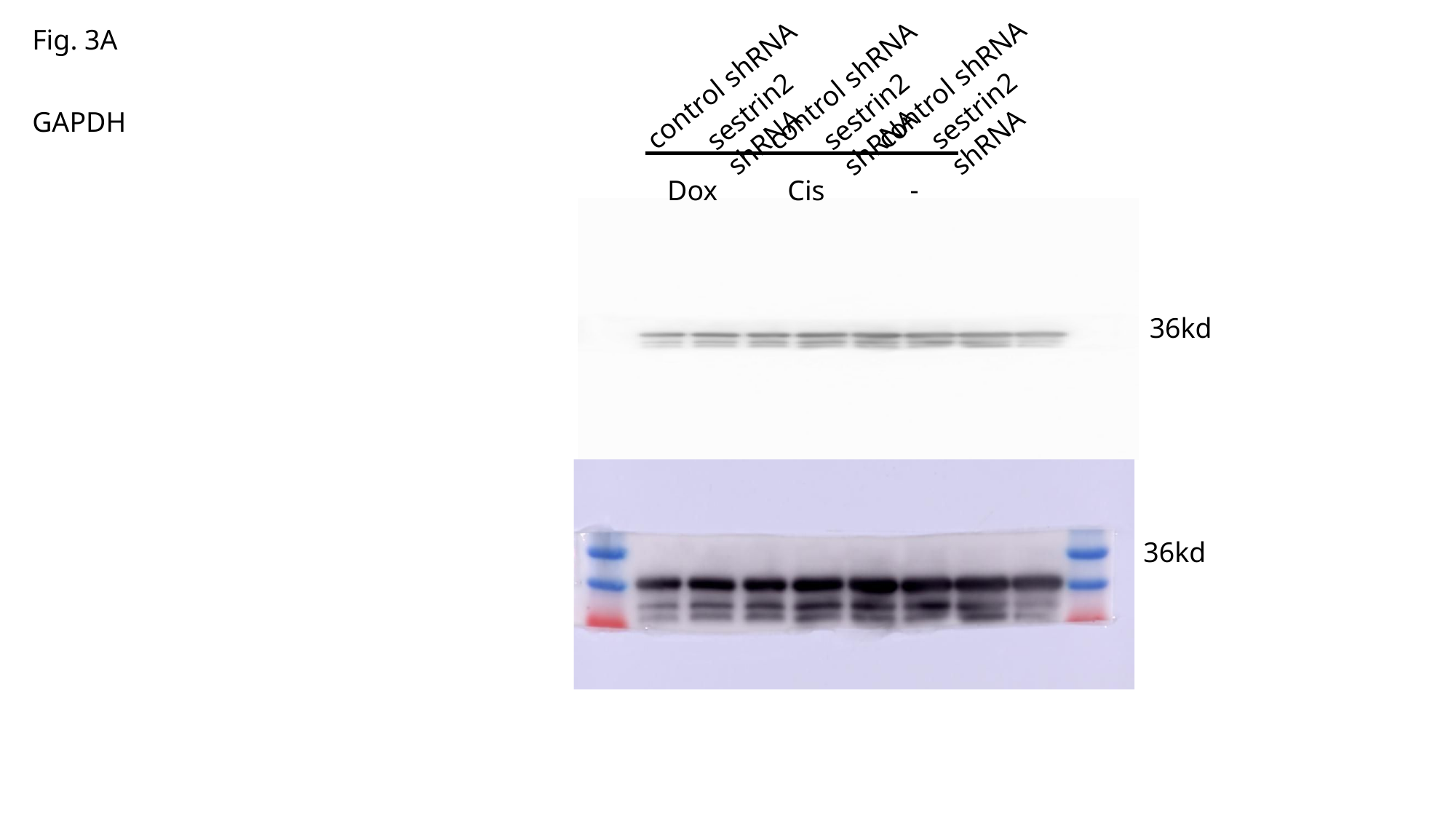

Fig. 3A
control shRNA
sestrin2 shRNA
control shRNA
sestrin2 shRNA
sestrin2 shRNA
control shRNA
GAPDH
-
Dox
Cis
36kd
36kd
