# Supplementary material for: Sestrin2-Mediated Autophagy Contributes to Drug Resistance via Endoplasmic Reticulum Stress in Human Osteosarcoma
Source: Front Cell Dev Biol. 2021 Sep 27;9:722960. doi: 10.3389/fcell.2021.722960 (PMC8502982; doi:10.3389/fcell.2021.722960)
Supplement: Supplementary file 13 [file Data_Sheet_14.ZIP › Raw data of western blot-2/Raw data of western blot-2.pptx]

## Slide 1
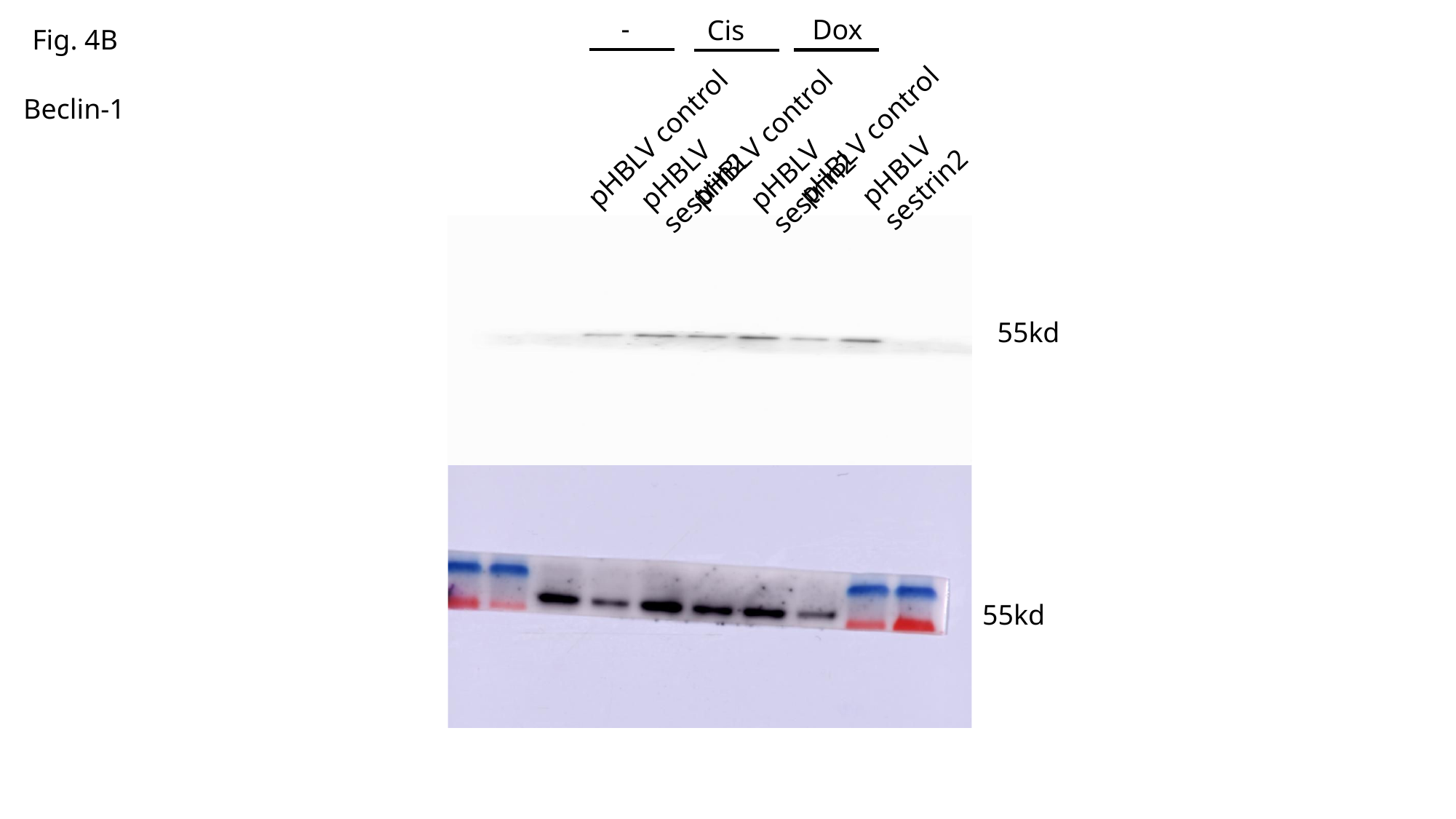

-
Dox
Cis
Fig. 4B
Beclin-1
pHBLV control
pHBLV sestrin2
pHBLV control
pHBLV control
pHBLV sestrin2
pHBLV sestrin2
55kd
55kd

## Slide 2
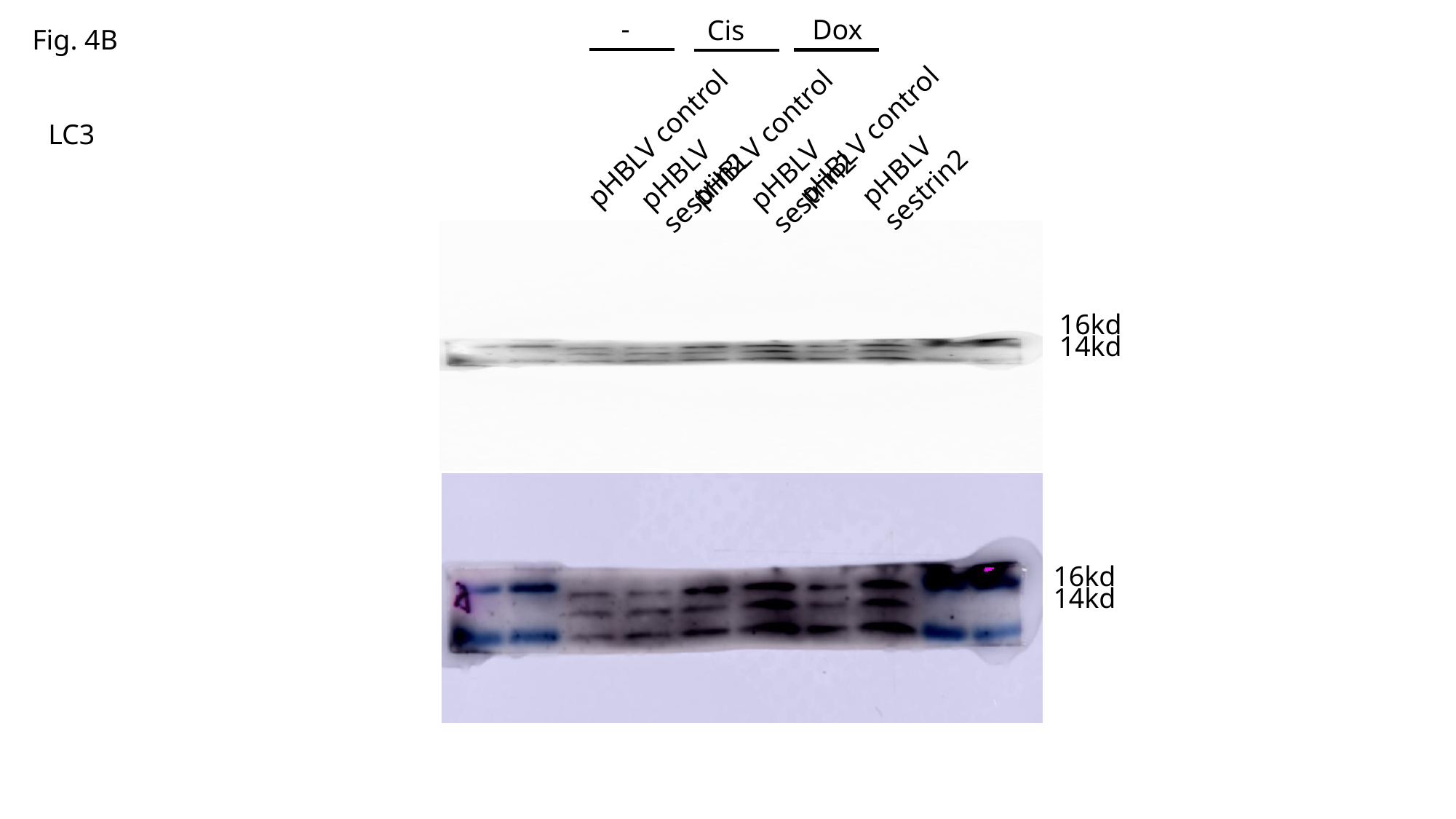

-
Dox
Cis
Fig. 4B
pHBLV control
LC3
pHBLV sestrin2
pHBLV control
pHBLV control
pHBLV sestrin2
pHBLV sestrin2
16kd
14kd
16kd
14kd

## Slide 3
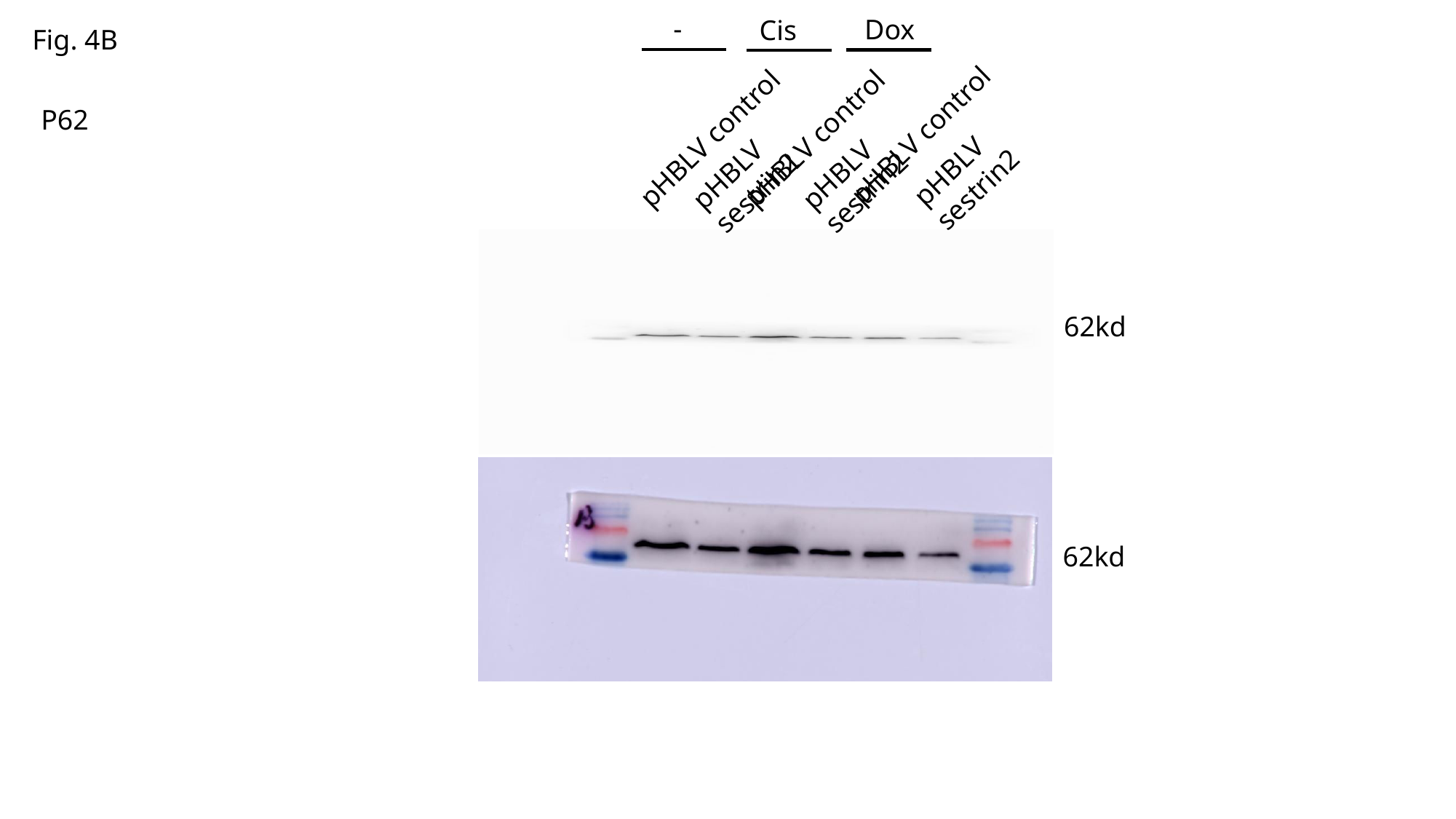

-
Dox
Cis
Fig. 4B
P62
pHBLV control
pHBLV sestrin2
pHBLV control
pHBLV control
pHBLV sestrin2
pHBLV sestrin2
62kd
62kd

## Slide 4
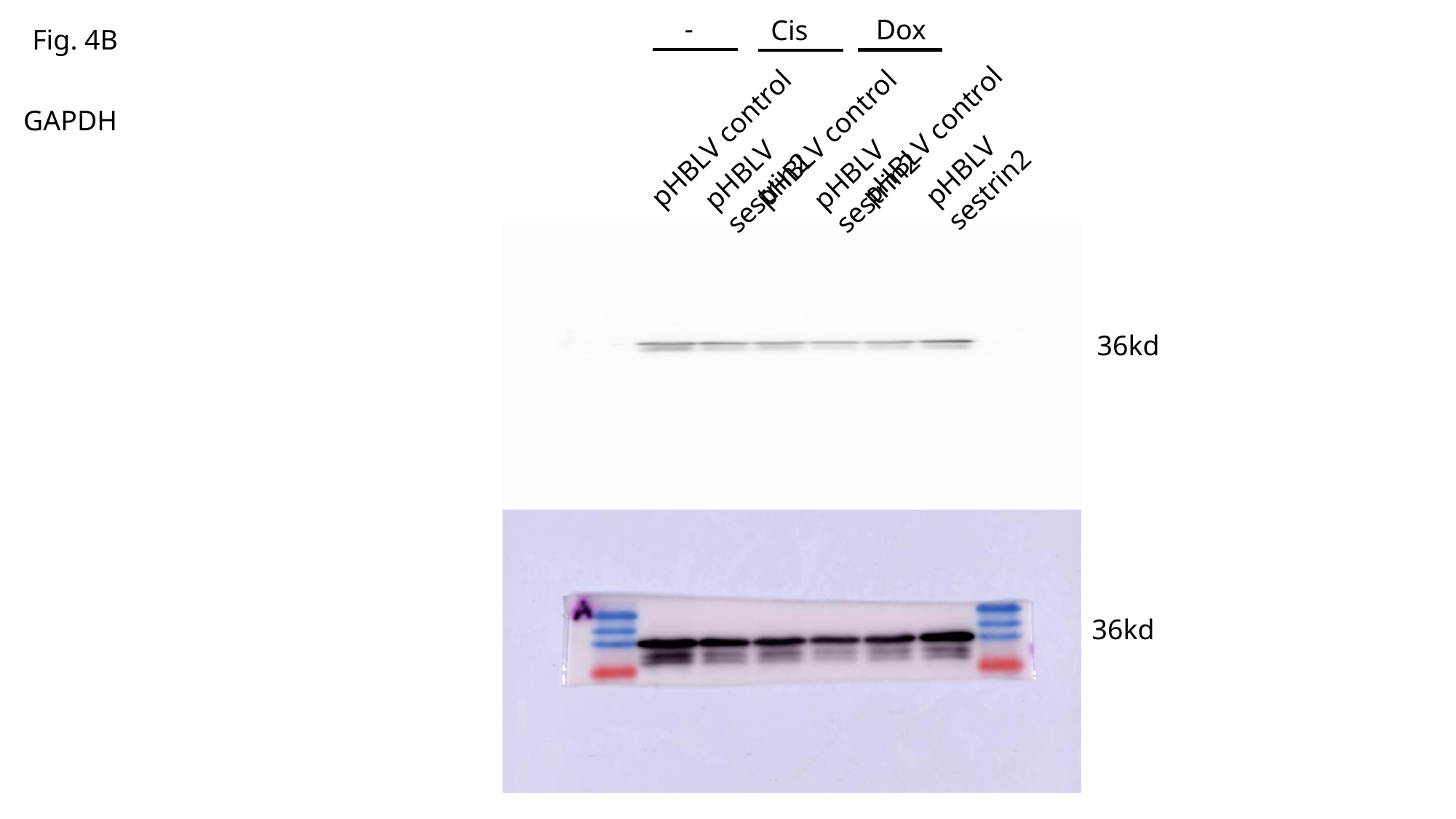

-
Dox
Cis
Fig. 4B
GAPDH
pHBLV control
pHBLV sestrin2
pHBLV control
pHBLV control
pHBLV sestrin2
pHBLV sestrin2
36kd
36kd

## Slide 5
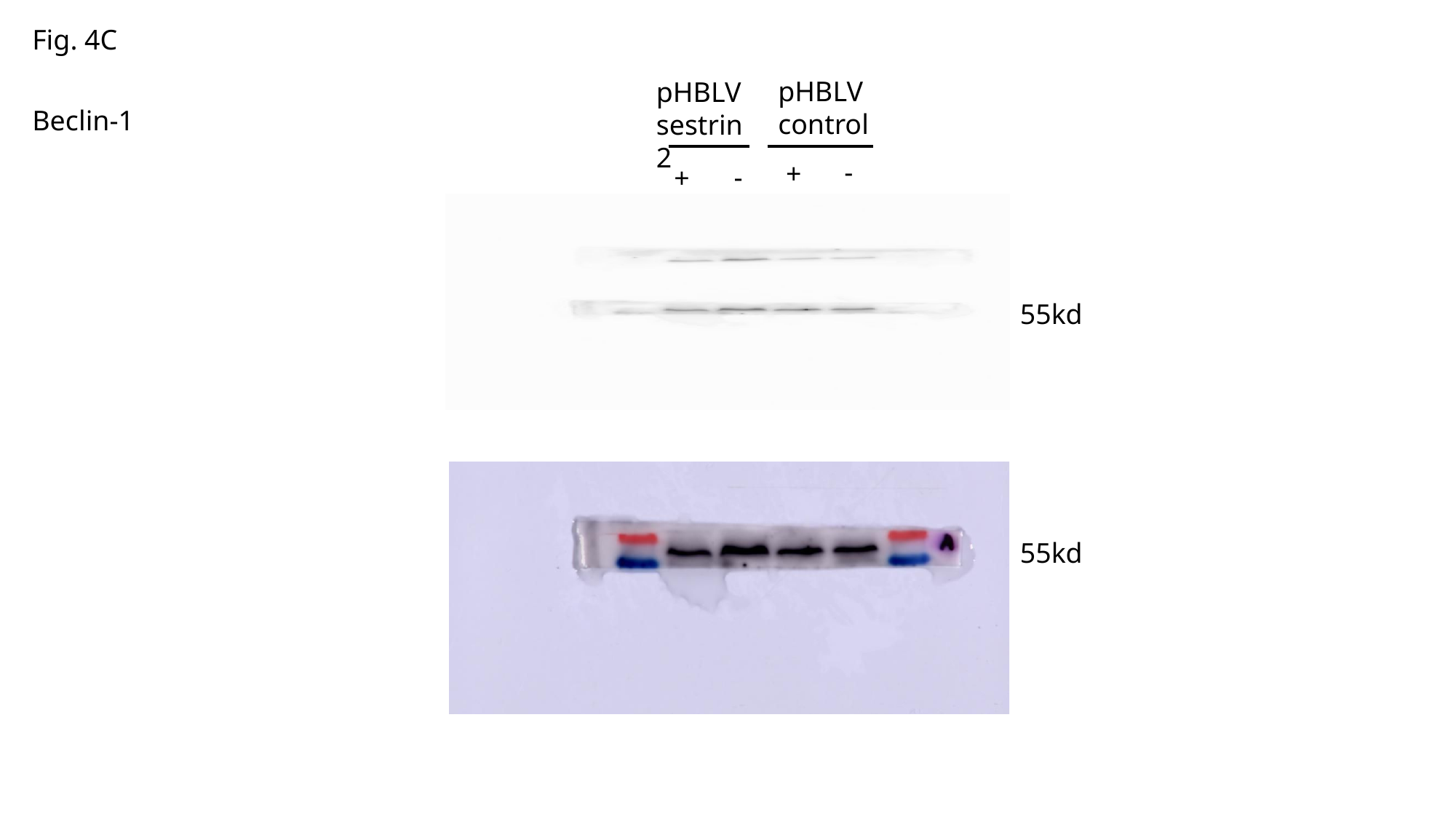

Fig. 4C
pHBLV
control
pHBLV
sestrin2
Beclin-1
-
+
-
+
55kd
55kd

## Slide 6
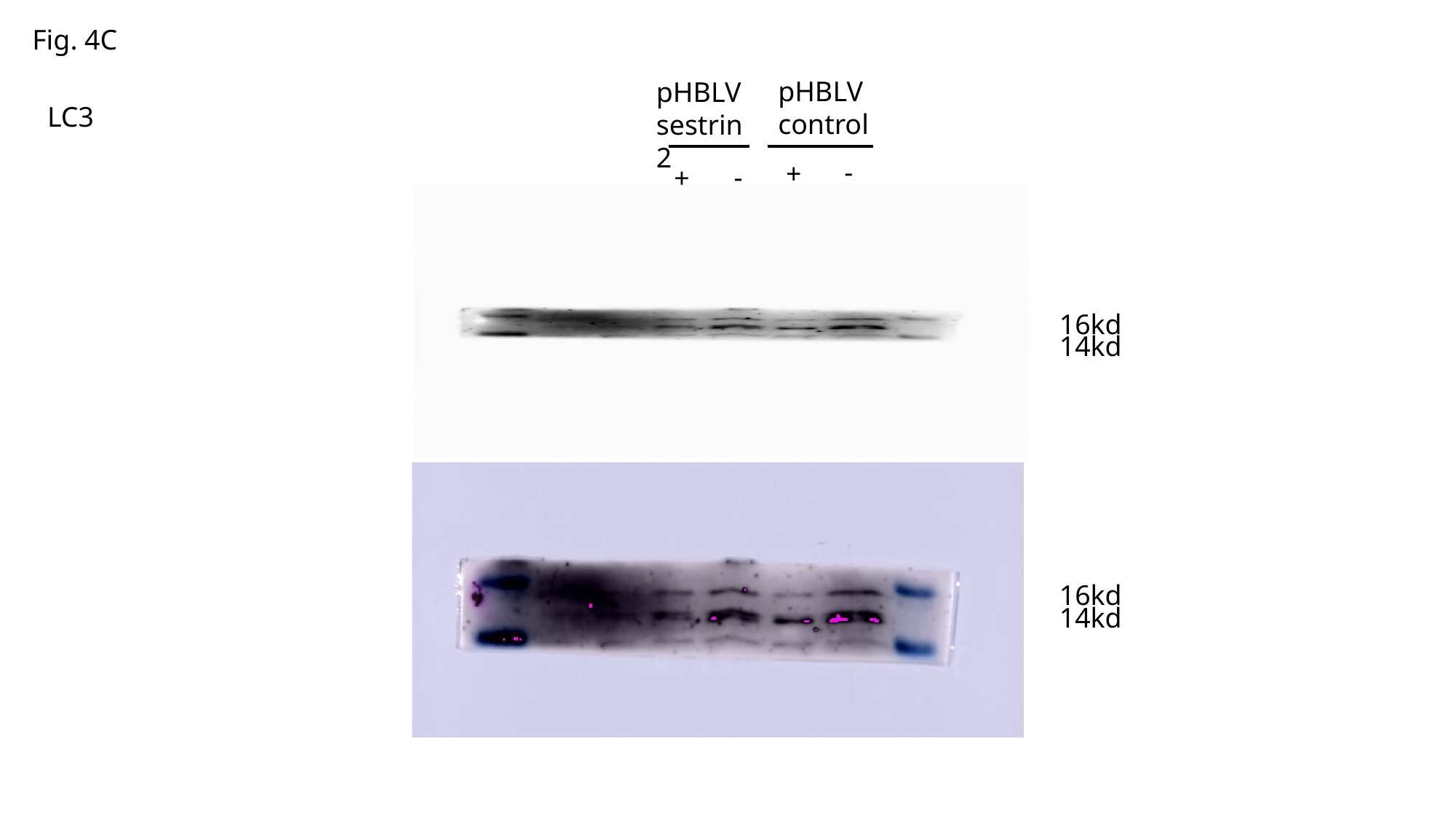

Fig. 4C
pHBLV
control
pHBLV
sestrin2
LC3
-
+
-
+
16kd
14kd
16kd
14kd

## Slide 7
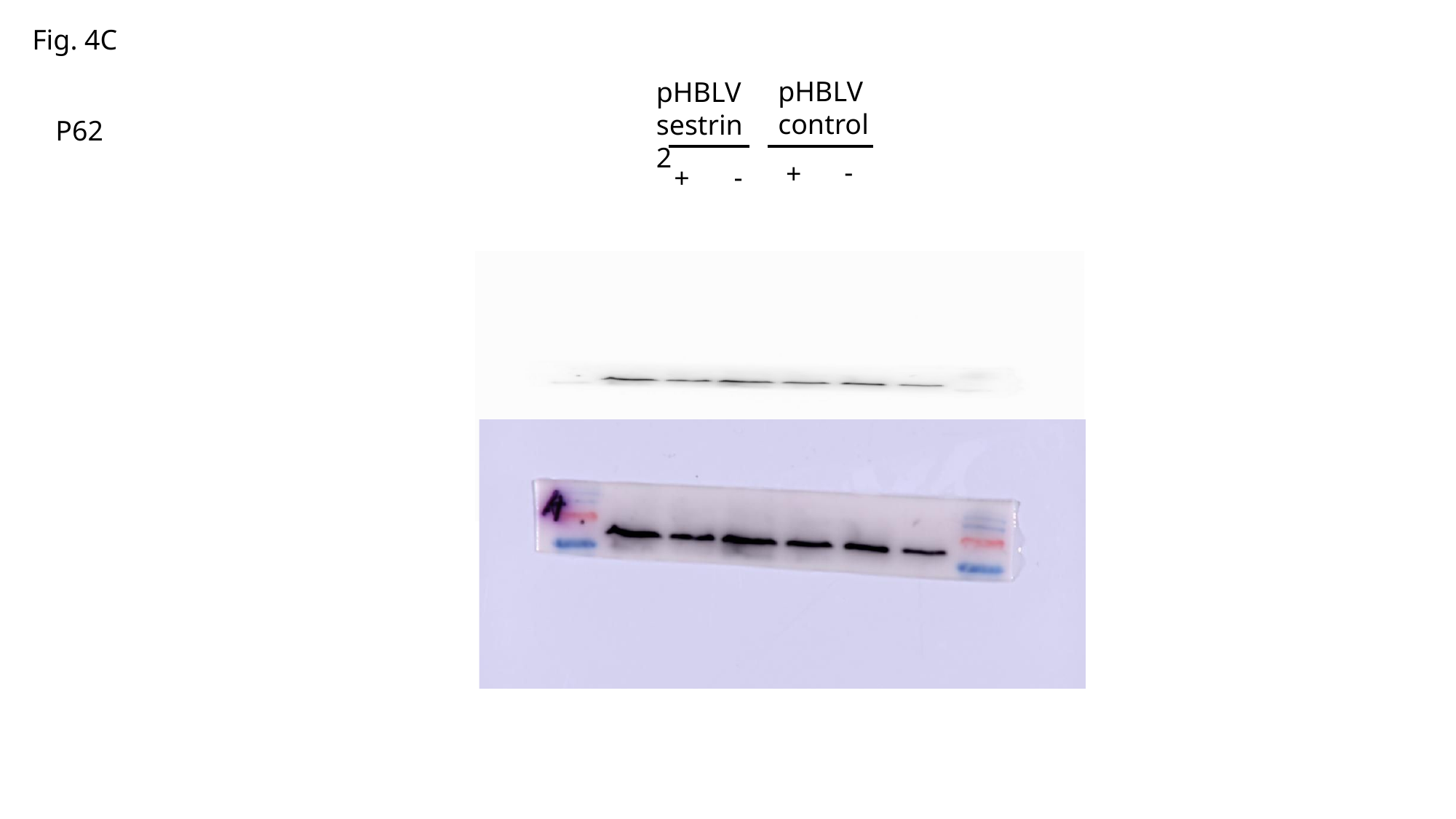

Fig. 4C
pHBLV
control
pHBLV
sestrin2
P62
-
+
-
+

## Slide 8
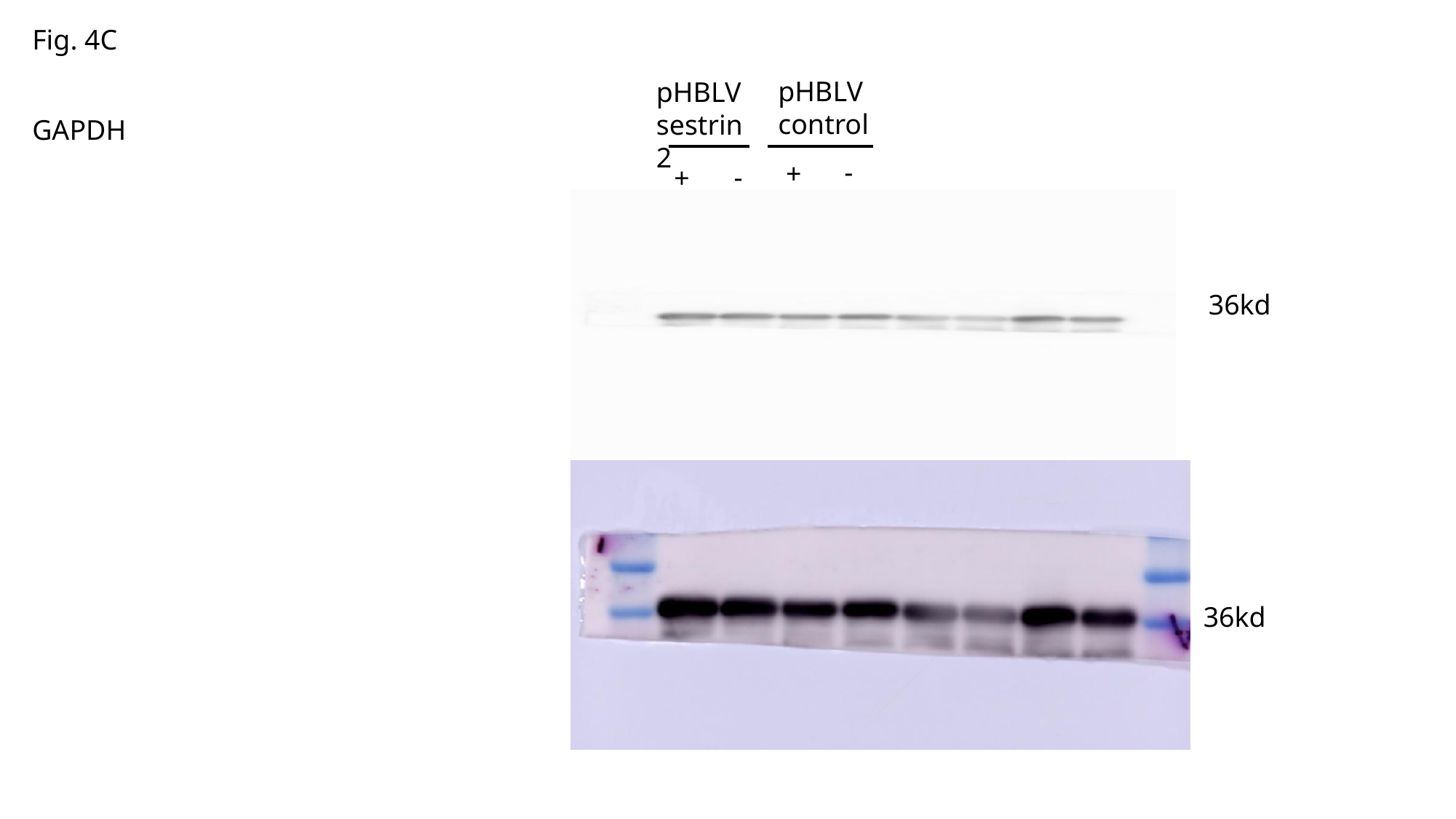

Fig. 4C
pHBLV
control
pHBLV
sestrin2
GAPDH
-
+
-
+
36kd
36kd

## Slide 9
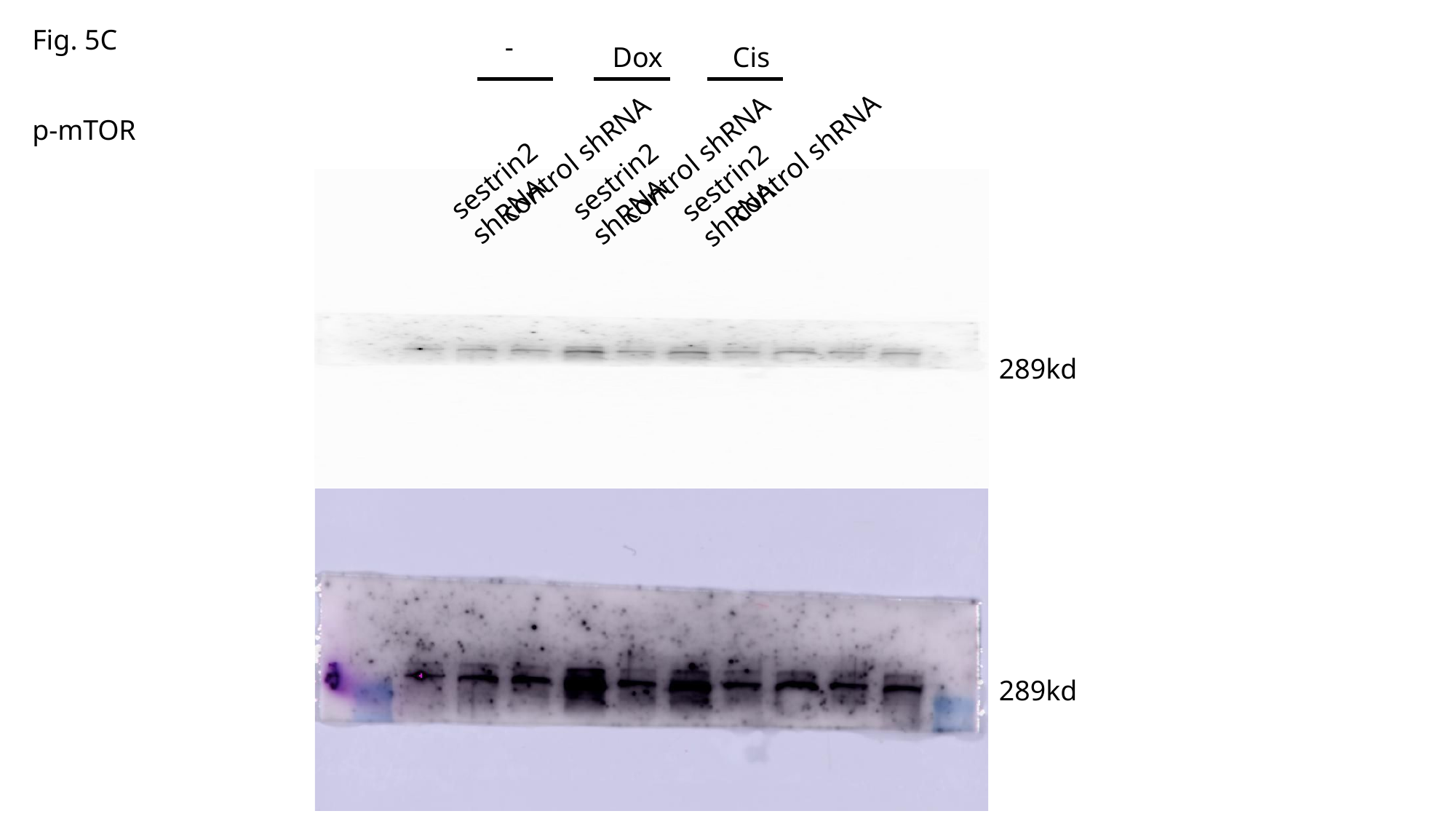

Fig. 5C
-
Dox
Cis
p-mTOR
sestrin2 shRNA
sestrin2 shRNA
sestrin2 shRNA
control shRNA
control shRNA
control shRNA
289kd
289kd

## Slide 10
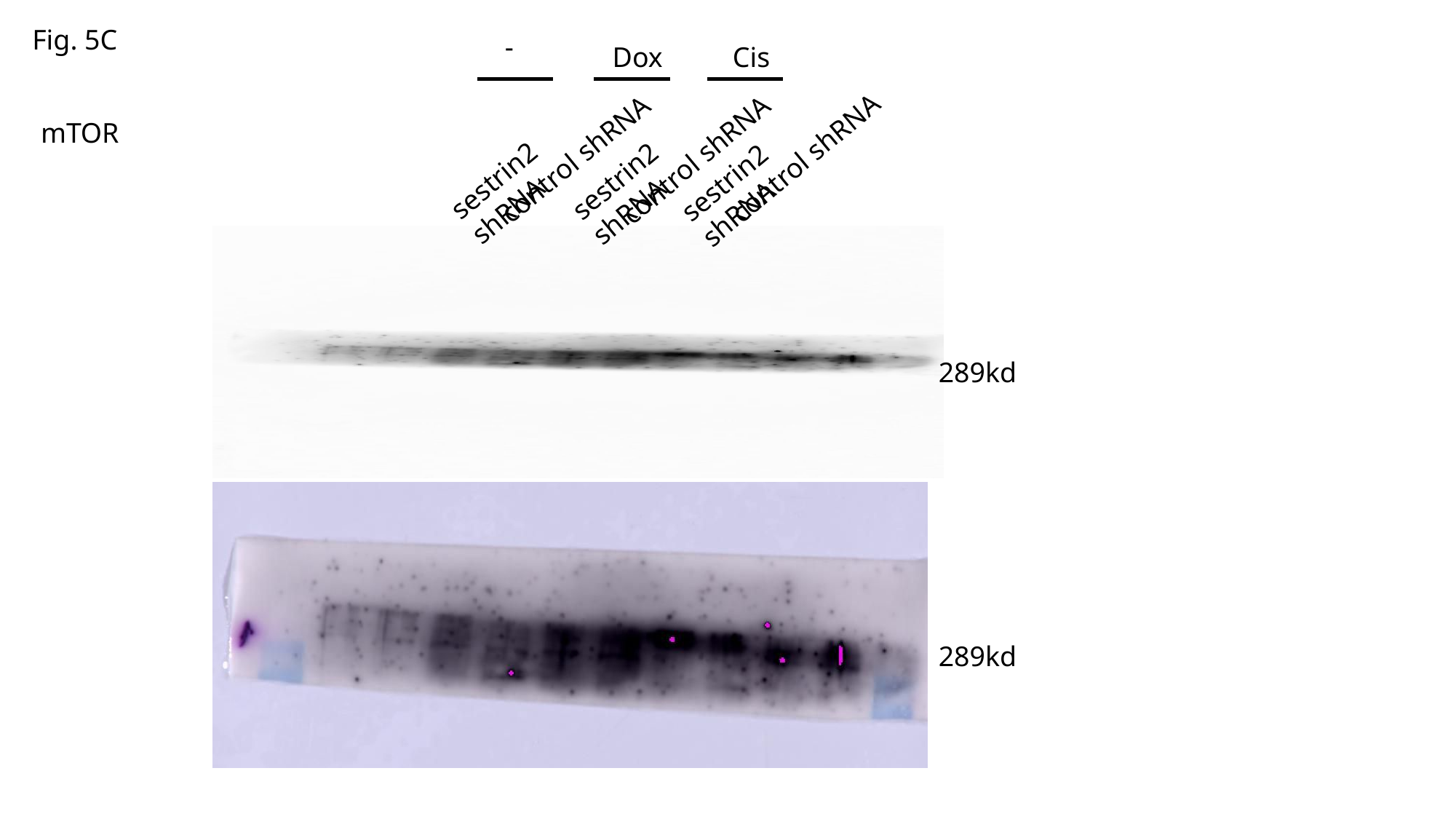

Fig. 5C
-
Dox
Cis
mTOR
sestrin2 shRNA
sestrin2 shRNA
sestrin2 shRNA
control shRNA
control shRNA
control shRNA
289kd
289kd

## Slide 11
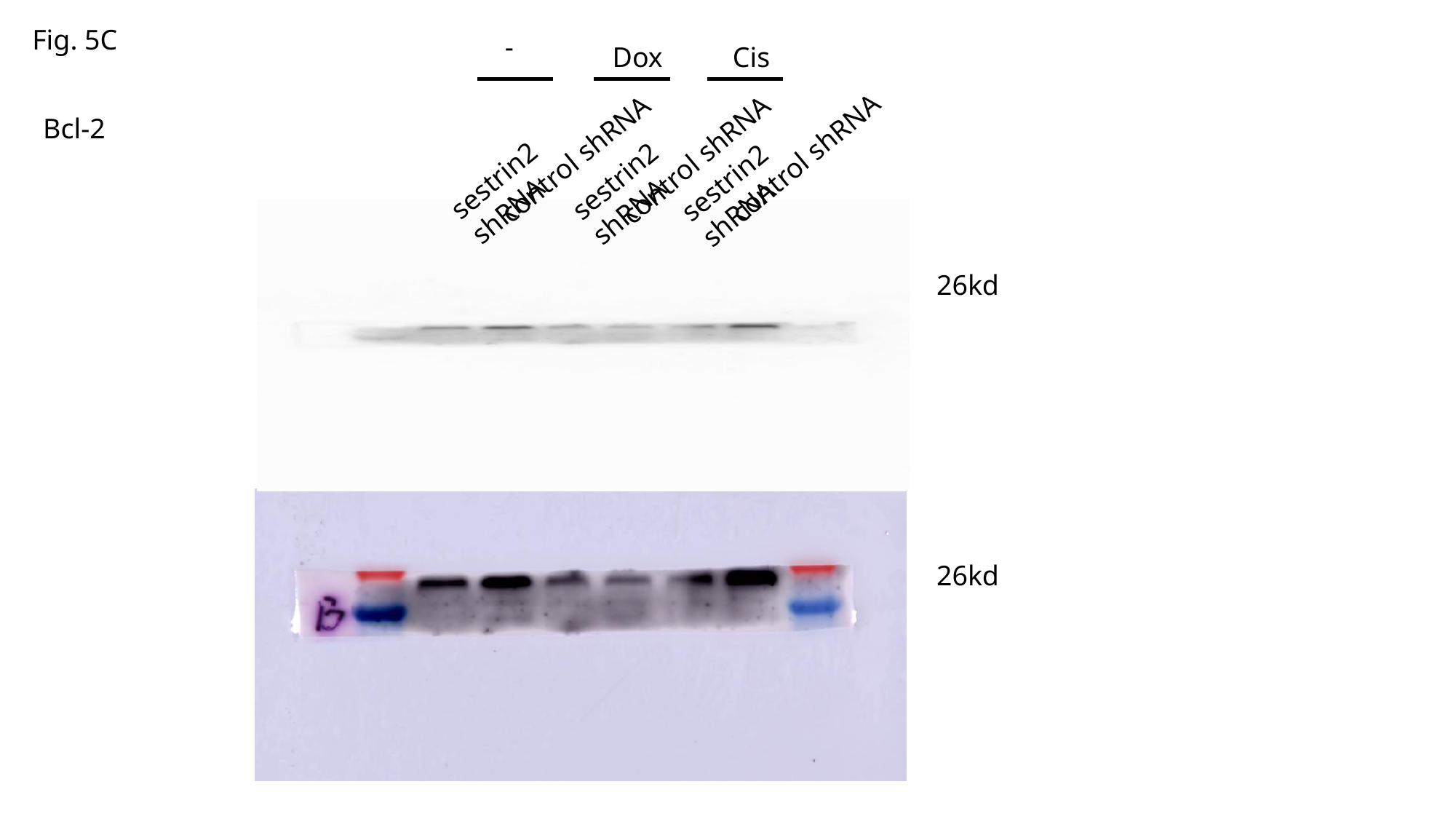

Fig. 5C
-
Dox
Cis
Bcl-2
sestrin2 shRNA
sestrin2 shRNA
sestrin2 shRNA
control shRNA
control shRNA
control shRNA
26kd
26kd

## Slide 12
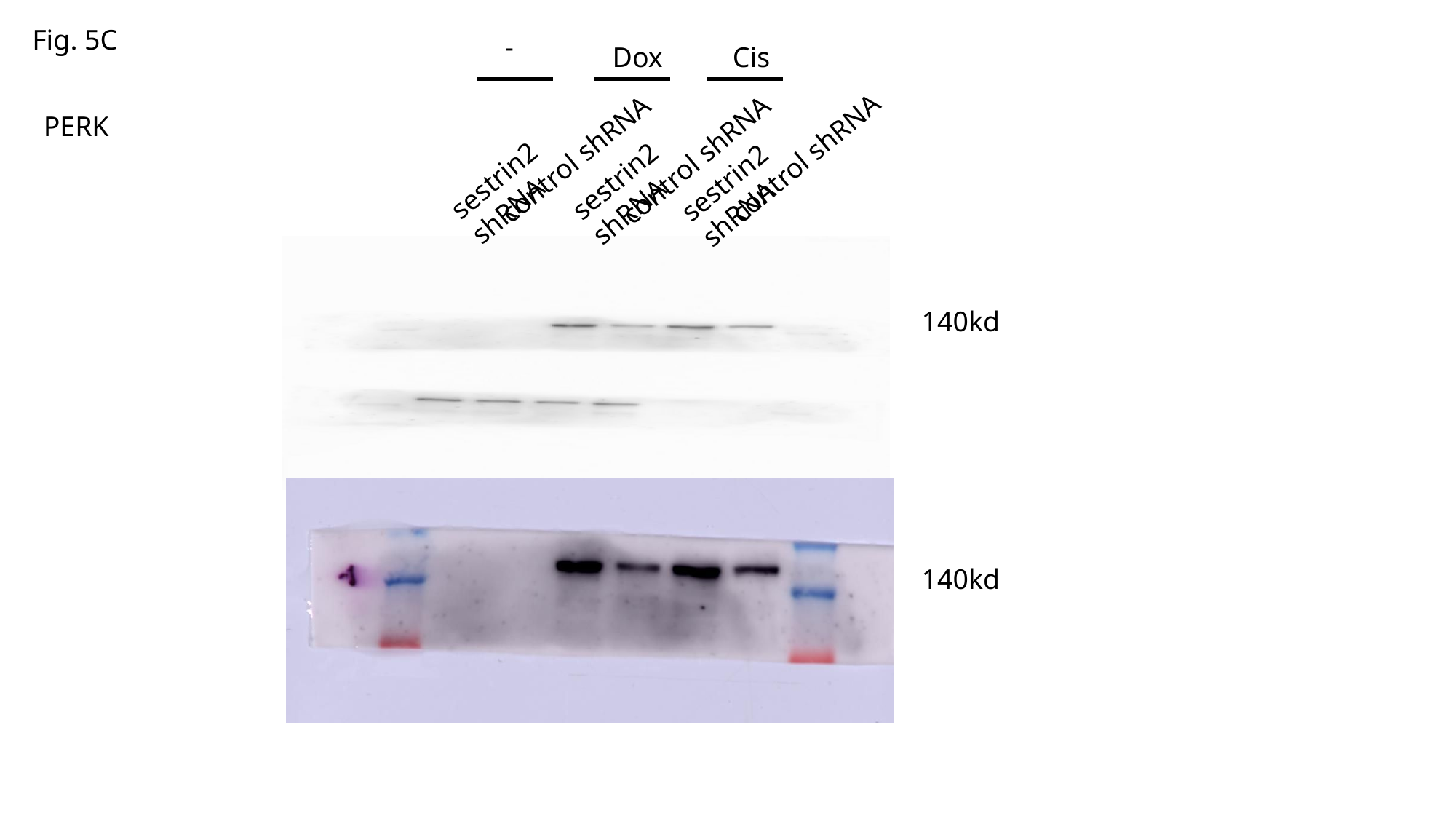

Fig. 5C
-
Dox
Cis
PERK
sestrin2 shRNA
sestrin2 shRNA
sestrin2 shRNA
control shRNA
control shRNA
control shRNA
140kd
140kd

## Slide 13
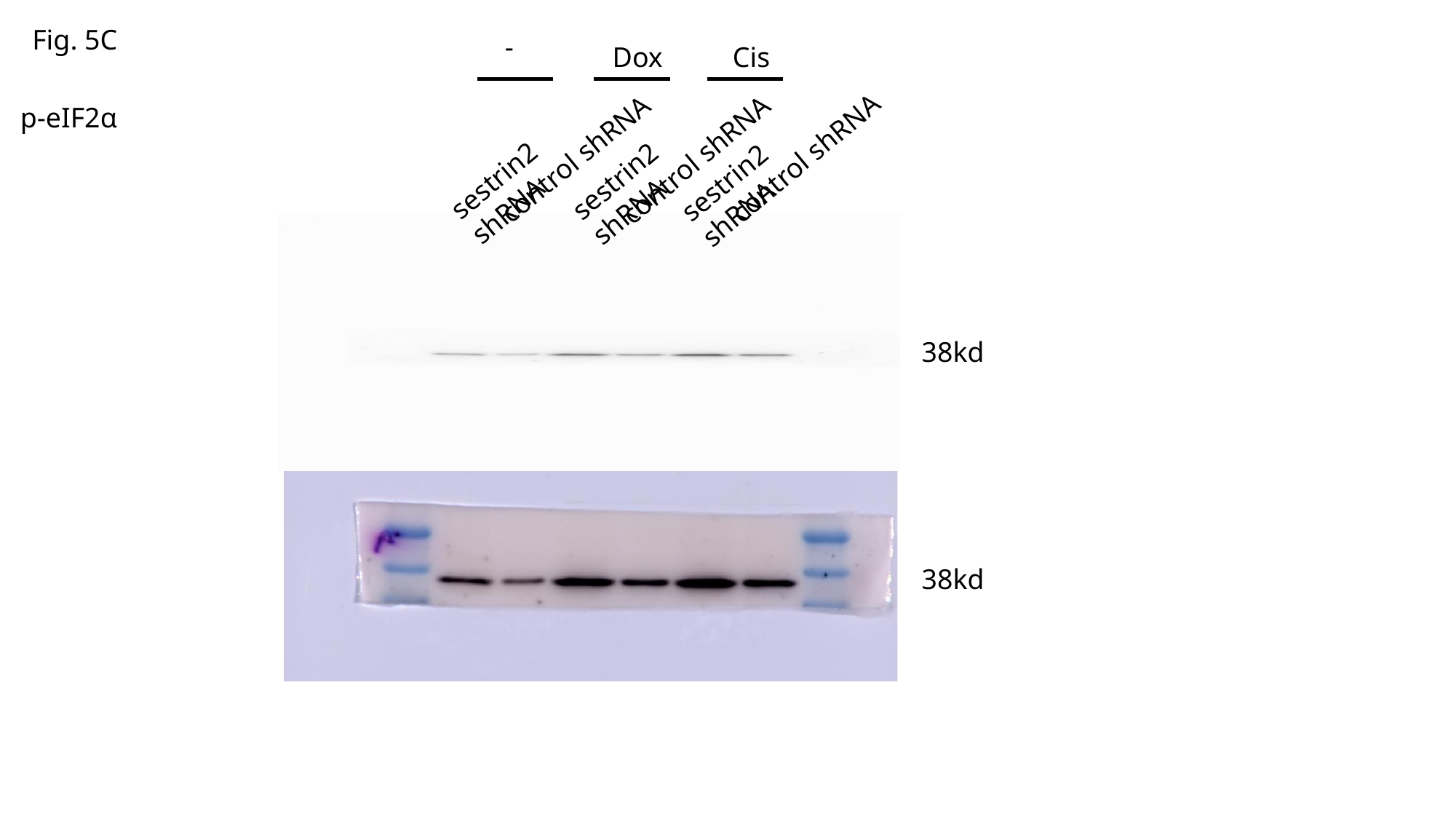

Fig. 5C
-
Dox
Cis
p-eIF2α
sestrin2 shRNA
sestrin2 shRNA
sestrin2 shRNA
control shRNA
control shRNA
control shRNA
38kd
38kd

## Slide 14
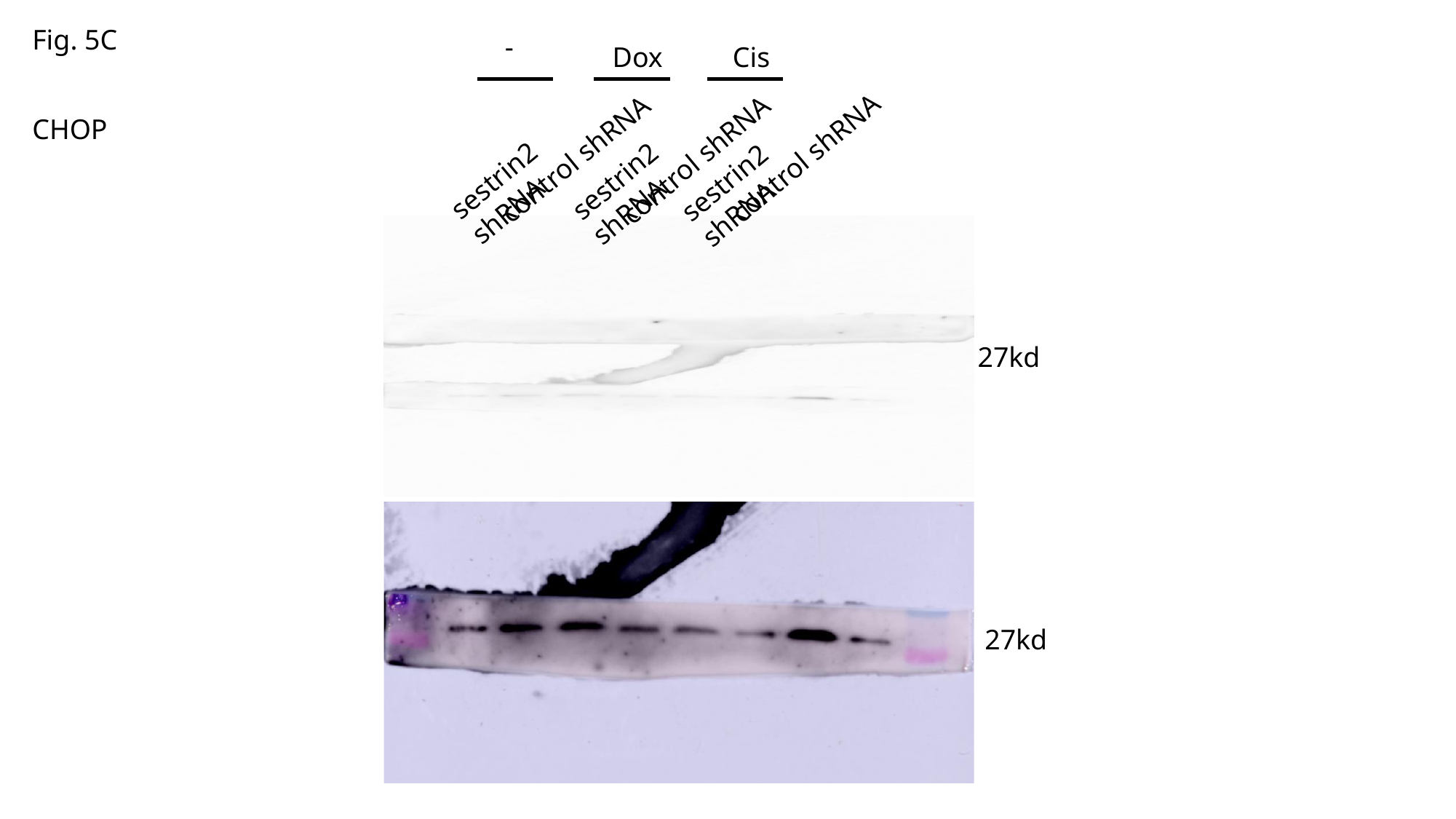

Fig. 5C
-
Dox
Cis
CHOP
sestrin2 shRNA
sestrin2 shRNA
sestrin2 shRNA
control shRNA
control shRNA
control shRNA
27kd
27kd

## Slide 15
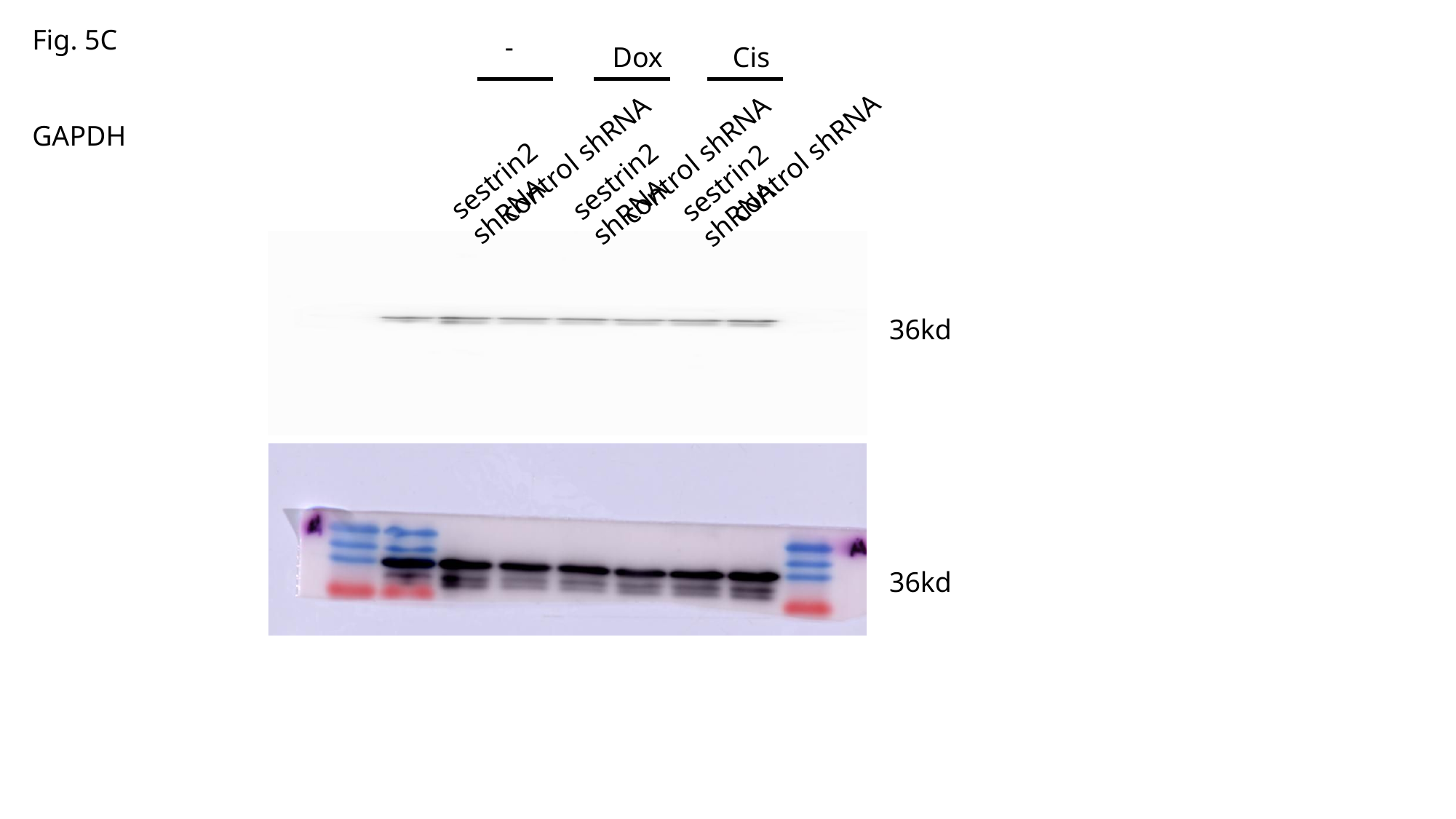

Fig. 5C
-
Dox
Cis
GAPDH
sestrin2 shRNA
sestrin2 shRNA
sestrin2 shRNA
control shRNA
control shRNA
control shRNA
36kd
36kd

## Slide 16
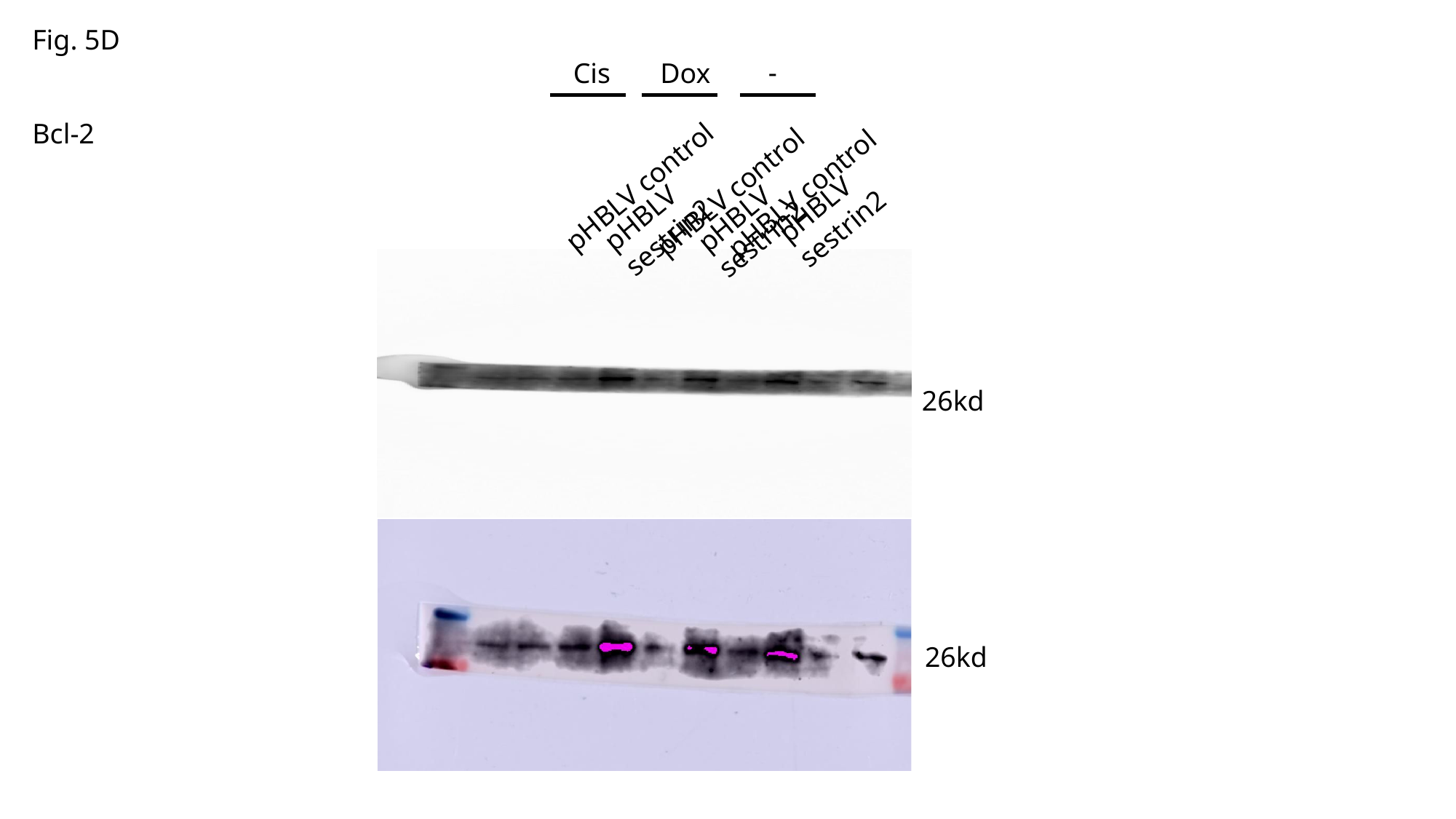

Fig. 5D
-
Cis
Dox
Bcl-2
pHBLV sestrin2
pHBLV sestrin2
pHBLV control
pHBLV sestrin2
pHBLV control
pHBLV control
26kd
26kd

## Slide 17
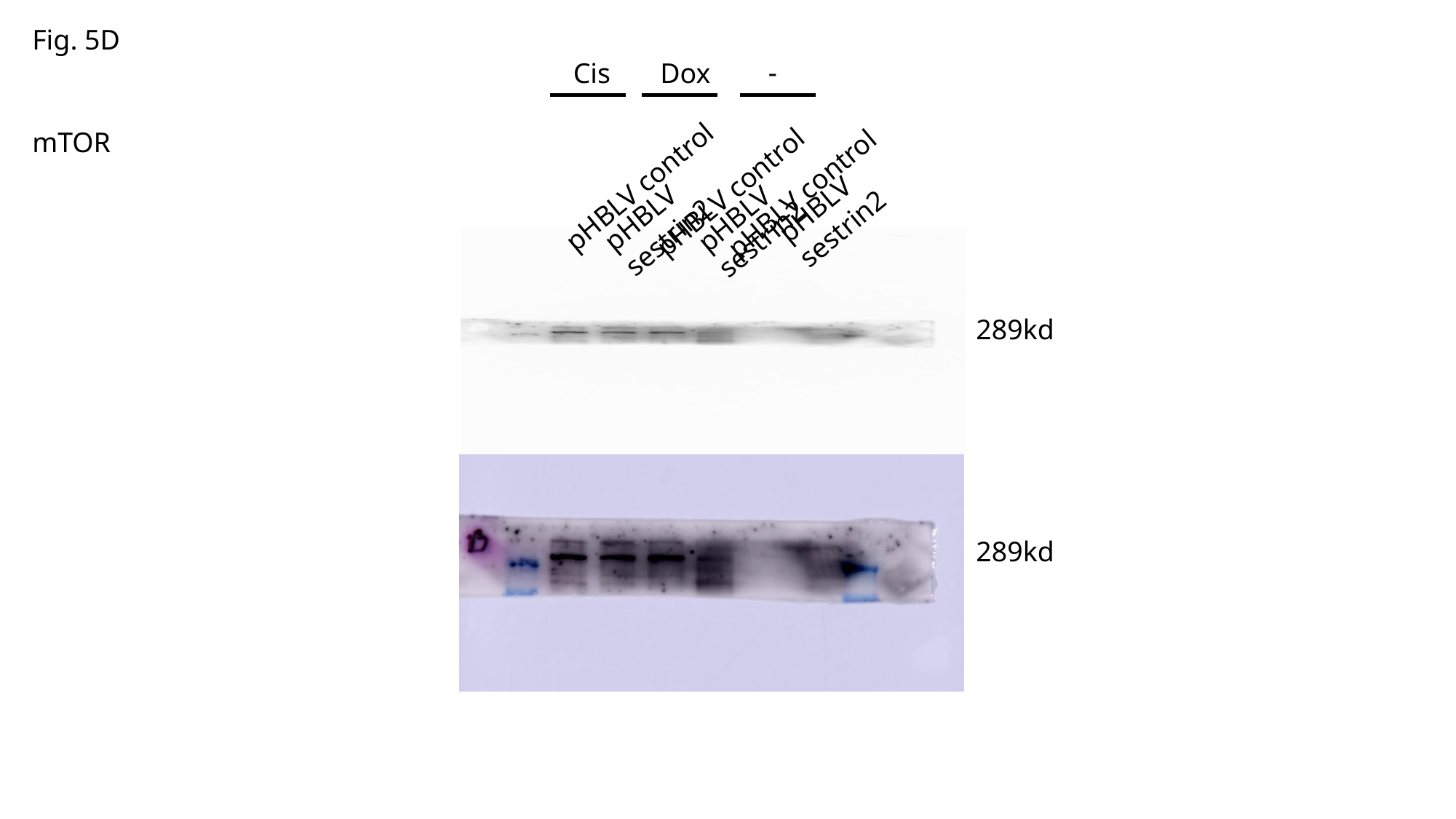

Fig. 5D
-
Cis
Dox
mTOR
pHBLV sestrin2
pHBLV sestrin2
pHBLV control
pHBLV sestrin2
pHBLV control
pHBLV control
289kd
289kd

## Slide 18
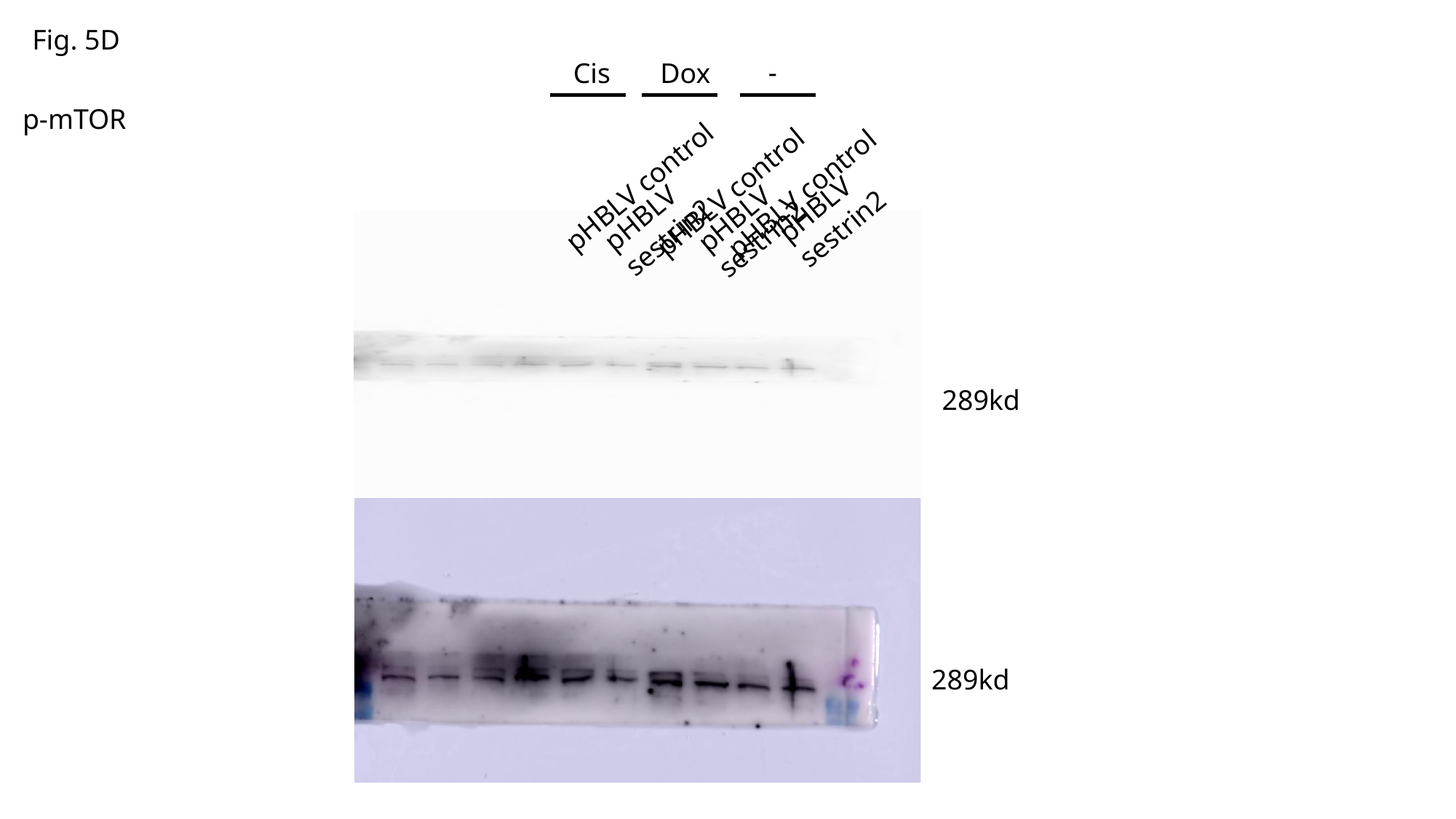

Fig. 5D
-
Cis
Dox
p-mTOR
pHBLV sestrin2
pHBLV sestrin2
pHBLV control
pHBLV sestrin2
pHBLV control
pHBLV control
289kd
289kd

## Slide 19
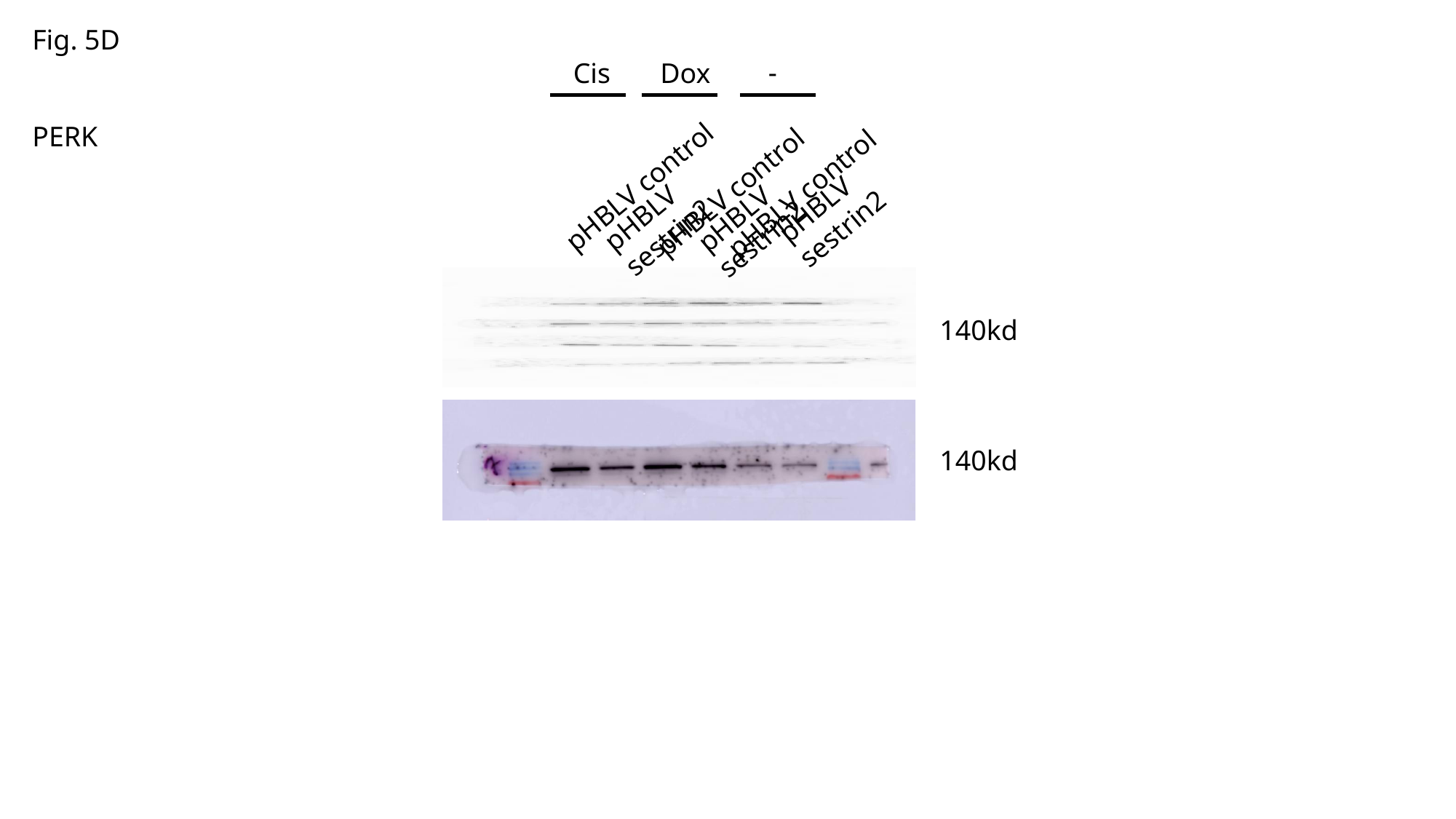

Fig. 5D
-
Cis
Dox
PERK
pHBLV sestrin2
pHBLV sestrin2
pHBLV control
pHBLV sestrin2
pHBLV control
pHBLV control
140kd
140kd

## Slide 20
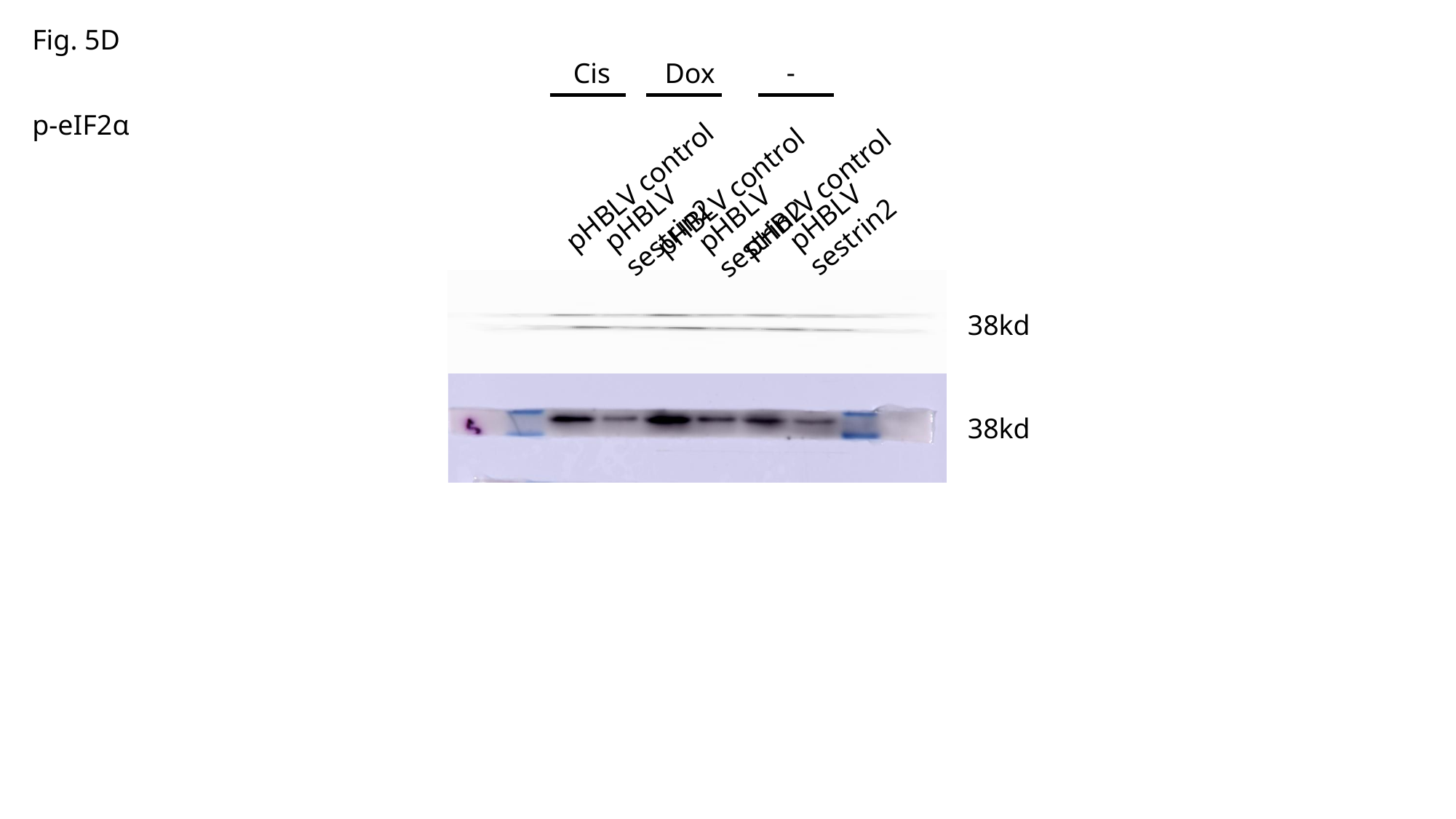

Fig. 5D
-
Cis
Dox
p-eIF2α
pHBLV sestrin2
pHBLV sestrin2
pHBLV control
pHBLV sestrin2
pHBLV control
pHBLV control
38kd
38kd

## Slide 21
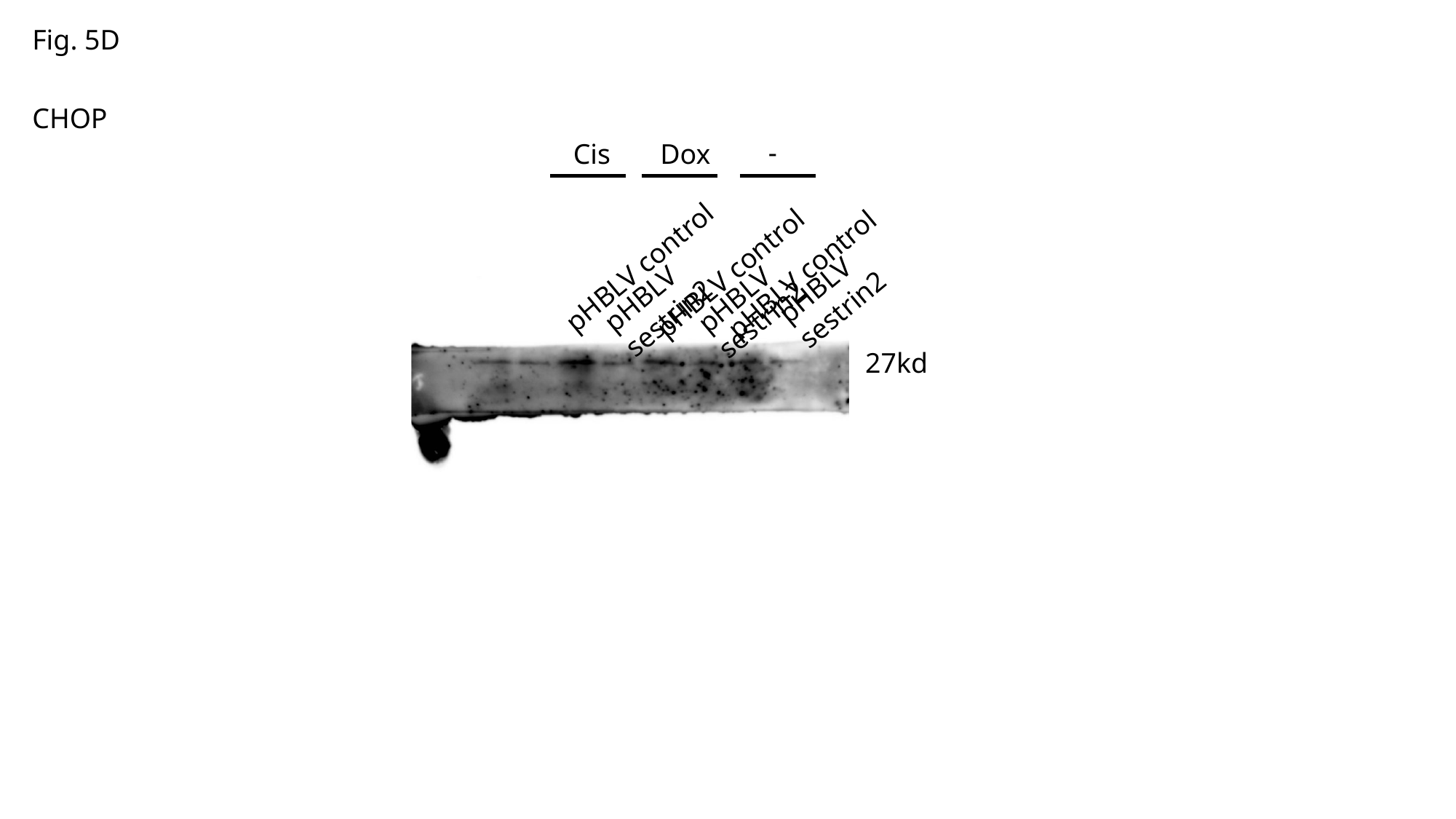

Fig. 5D
CHOP
-
Cis
Dox
pHBLV sestrin2
pHBLV sestrin2
pHBLV control
pHBLV sestrin2
pHBLV control
pHBLV control
27kd

## Slide 22
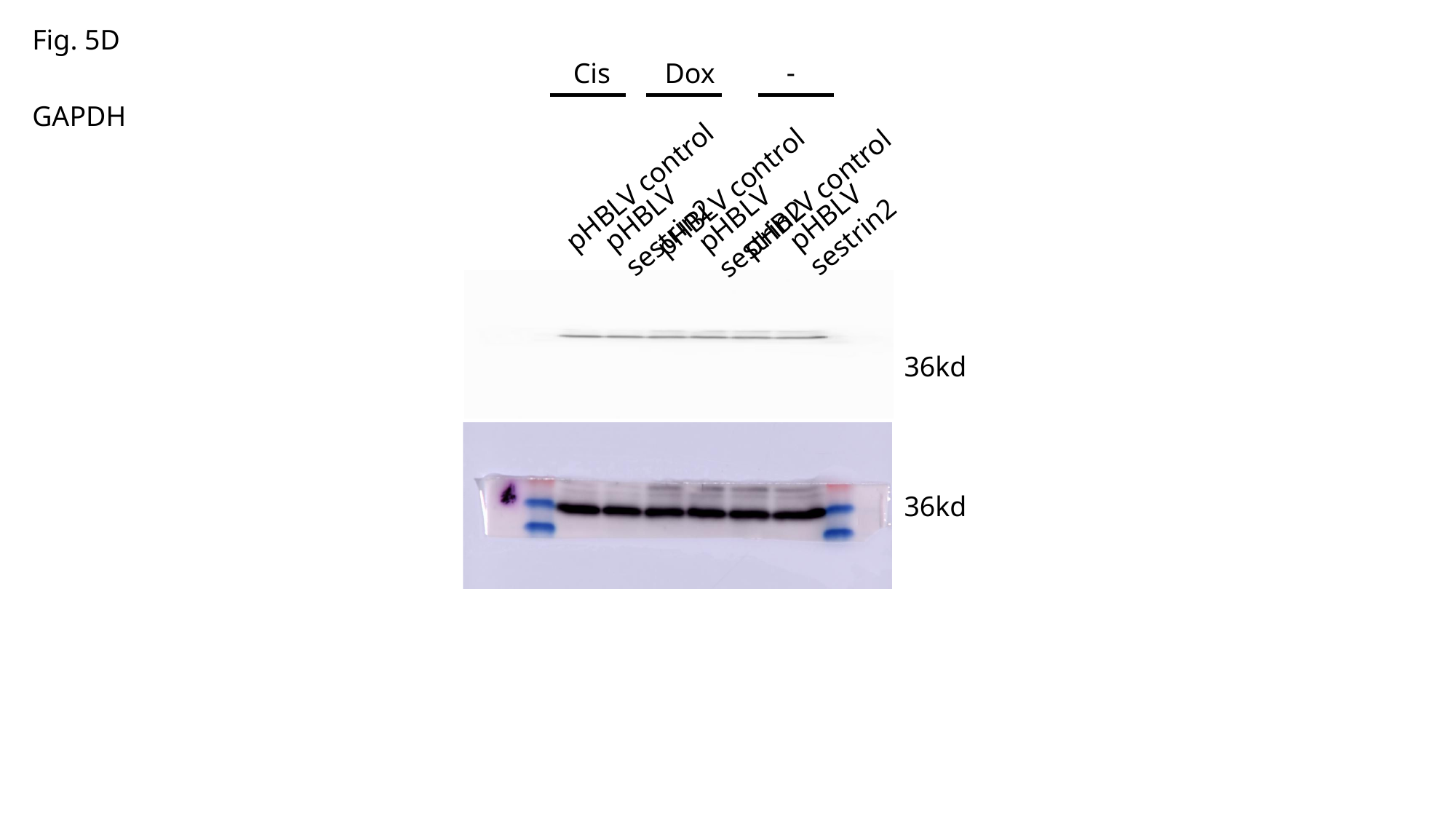

Fig. 5D
-
Cis
Dox
GAPDH
pHBLV sestrin2
pHBLV sestrin2
pHBLV control
pHBLV sestrin2
pHBLV control
pHBLV control
36kd
36kd
